# Estimation of the size, density, and demographic distribution of the UK pet dog population in 2019

**DOI:** 10.1038/s41598-024-82358-y

**Published:** 2024-12-30

**Authors:** Kirsten M. McMillan, Xavier A. Harrison, David C. Wong, Melissa M. Upjohn, Robert M. Christley, Rachel A. Casey

**Affiliations:** 1 Dogs Trust, London, UK; 2 University of Exeter, Exeter, UK; 3 University of Leeds, Leeds, UK

**Keywords:** Biogeography, Ecological modelling, Population dynamics

## Abstract

There is limited knowledge about the size of the UK dog population. This makes it difficult to reliably monitor population dynamics and management. A repeatable method of measuring the UK dog population, including owned and unowned dogs i.e., those housed long term by animal welfare charities, would help inform interventions to reduce risks to dog welfare such as poor breeding practices and the illegal import of young or pregnant dogs. However, estimating the total dog population in the UK is not straightforward. Although several estimates of dog numbers have been previously suggested, differing methodologies and limited public access to data sources makes comparison and replication difficult. In this study, we compiled a comprehensive dataset of UK pet dogs in 2019, from multiple sources, including a breed registry, veterinary corporations, pet insurance companies, animal welfare charities and an academic institution. Using a hierarchical Bayesian N-mixture model, we estimate 12.64 million (mean, 95% CI 8.54–15.16 million; median, 13.03 million, 95% CI 8.51–15.24 million) owned and unowned pet dogs within the UK in 2019. Estimates were modelled at the postcode area scale, allowing for aggregation to region or country level, as well as providing associated metrics of uncertainty. Furthermore, we provide spatial demographic estimates, regarding age, breed, cephalic index, and body size. Establishing a population baseline offers significant analytical benefits to welfare, veterinary, epidemiological, and business stakeholders alike: as it provides the spatial data required to underpin robust canine welfare strategies and campaigns. We plan to repeat the described process, in order to provide ongoing estimates: allowing for the interrogation of changes to the UK pet dog population over space and time.

**Supplementary Information:**

The online version contains supplementary material available at 10.1038/s41598-024-82358-y.

## Introduction

Dogs are the most popular pet within the United Kingdom (UK), with 31% of households owning at least one dog^1^. Accurate population estimates and knowledge of geographic variation, are crucial for understanding the canine ‘market size’, along with the demand for dogs/puppies over space and time. Despite this, we have limited knowledge regarding the total canine population size, incorporating both owned and unowned dogs i.e., those housed long term by animal welfare charities. Within the UK, there appears to be no consistent population of free-living unowned dogs, either as supported ‘strays’ (i.e., receiving food, even irregularly, and/or occasional veterinary support), or as completely self-sustaining feral packs^2^. Instead, strays are recycled back into the owned sub-population via reunification with their previous owner^3^ or care of an animal welfare charity^2,4^. Therefore, this study does not specifically address ‘stray’ dogs but includes both owned and unowned dogs in all subsequent analyses.

Increasing consumer demand for dogs^5,6^ and the associated financial benefits for those selling puppies^7,8^, has led to several practices that have negative impacts on dog welfare. These include: the sale of puppies bred in large-scale establishments with unsuitable environments with regards to health and behavioural development; legal and illegal international transportation of puppies with associated welfare and disease transmission risks; and sales through online advertising. The latter provides an ideal platform for sellers to prosper from impulse and/or poorly planned purchases, whilst simultaneously accommodating two-way anonymity^2,7,9–14^. These factors, coupled with a naïve consumer market, has led to experts’ warning of a canine welfare crisis^15,16^. To understand the UK pet dog ‘market’, including factors that influence supply, the first step is to reliably quantify the UK pet dog population.

Estimating the total population size of dogs within any geographical context is not straightforward, in part due to a lack of comparable and accessible datasets^17^. Though several UK estimates have been put forward in recent years (discussed below), details of methodological approach and data sources generally have not been made publicly available. This makes comparisons across studies difficult, as well as limiting reproducibility of results. UK Pet Food (previously Pet Food Manufacturing Association) have provided annual rolling population totals from 2011 to 2023. Their 2019–2020 estimate was 9.0 million^18^, whilst estimates for 2021, 2022, and 2023 were reported as 12.0, 13.0 and 12.0 million, respectively. However, methodological changes prevent direct comparison between estimates pre and post 2021. Surveys from 2011 to 2020 were conducted face-to-face with a sample size of 4,203-8,353 households per year, whilst 2021–2023 surveys were online, with a sample size of approximately 9,000 households^1,18^. Furthermore, evaluating the accuracy of UK Pet Food estimates is difficult, as they lack associated metrics of uncertainty, and exact methodologies are not made publicly available (e.g., sampling, geographic coverage, and estimations). Given the historical knowledge gap, this estimate has been hugely valuable for many industries. Nonetheless, the methods remain limited in scope. For example, the Office for National Statistics estimated 27.8 million UK households in 2019^19^, which equates to a UK Pet Food sampling rate of ~ 0.03% of the UK’s total households ($\:n$ = 8,000), or ~ 0.01% per year.

Based on public surveys, previous estimates have varied between 10.5 and 11.5 million^20,21^. However, as surveys are costly initiatives, reliant on active marketing strategies, participant numbers and/or geographic coverage, there may be financial constraints. The limited absolute and spatially explicit sample sizes may have led to these surveys producing unreliable population estimates. In 2011, Asher et al.^22^ approached this challenge from a fresh perspective: by identifying and including three external data sources that enriched public survey data, providing four distinct estimates based on varying combinations of data sources. Despite broadening the data pool, the authors suggested that the most reliable estimate was based on public survey data alone, at 9.4 million. This judgement was based on the potential under- and over-estimate of the population, when incorporating a combination of public survey and insurance records, or public survey and veterinary surveys, respectively^22^. Since then, no further research has built upon this important study (however, see MacDonald et al.^23^ regarding UK feline population dynamics).

To provide an independent and more robust estimate of total dog population size (including both owned and unowned dogs), along with details of spatial demographics, we developed a research infrastructure of 18 project participants, including a breed registry, veterinary corporations, pet insurance companies, animal welfare charities and an academic institution (further detail outlined in Methods *‘Data: sources*,* cleaning*,* and deduplication’*). Data were combined to generate a robust and distinct estimate of the UK pet dog population in 2019, across multiple spatial scales: facilitating greatest applicability for researchers/stakeholders. The year 2019 was selected as the year of study, for the following reasons (1) project was initiated in 2020; (2) results serve as proof of concept for ongoing UK dog population estimates. Specifically, in order to analyse spatiotemporal changes to the UK pet dog population, we intend to replicate the process detailed in this paper to provide ongoing estimates. This approach will help researchers and stakeholders understand evolving patterns in dog ownership following specific events. Consequently, delay between project initiation and publication may be attributed to developing reproducible pipelines to expedite future estimates.

We applied a hierarchical Bayesian N-mixture model to estimate population sizes in a closed mark–recapture framework, using human population density data to improve precision of our estimates (as pet dogs are inherently located with owners). The advantage of this approach is that we model population size at the scale of postcode area (i.e., initial characters of the alphanumeric UK postcode), which can then be compiled to provide regional or country level population estimates, as well as associated metrics of uncertainty. Furthermore, spatial demographic details, regarding age, breed, cephalic index (brachycephalic, mesocephalic, and dolichocephalic) and body size (large, medium, and small), can be calculated by extracting proportions from the raw data and used to partition the regional and country level population estimates.

Establishing a UK pet dog population baseline offers significant analytical benefits to welfare, veterinary, epidemiological, and business stakeholders alike: as it provides the spatial data required to underpin robust canine welfare strategies and campaigns.

## Results

Dogs Trust data were combined with datasets sourced from 17 external project participants. Data sources included a breed registry (45.0%: The Kennel Club (KC), UK), veterinary corporations (26.5%: PDSA; Medivet; Vets4Pets), pet insurance companies (17.1%: The Insurance Emporium (The Equine and Livestock Insurance Company Limited); NCI Insurance; Cardif Pinnacle; Agria Pet Insurance; Direct Line), animal welfare charities (5.9%: Battersea Dogs and Cats Home; Blue Cross; SSPCA; Raystede; Wood Green, The Animals Charity; Edinburgh Dog and Cat Home; Mayhew) and an academic institution (5.5%: SAVSNET - Small Animal Veterinary Surveillance Network, University of Liverpool). Prior to removal of duplicate individuals and those aged > 18.3 years^24^ (see *Methods* for data cleaning and deduplication details), raw data included 12,348,414 dogs for the year 2019, representing a 1.04:1 male skew ($\:{n}_{\text{♂}}$ = 2,224,732; $\:{n}_{\text{♀}}$= 2,139,057), and a 6:1 ratio of pure ($\:{n}_{E}$ = 3,694,017) to crossbred individuals ($\:{n}_{X}$ = 613,079).

Following data cleaning and deduplication, dataset included 4,375,861 dogs for the year 2019: suggesting that, on average, individual dogs were sampled 2.8x across data sources. Deduplication consisted of a four-phase process, whereby duplicate individuals were limited to one entry (outlined within Supplementary Note 1). During Phase 1 of deduplication, 28.7% of the raw data were identified as exact duplicates (i.e., exact matches for any cases with more than 10/18 variables), with 6.2% of remaining cases unable to be identified as duplicates during Phases 2, 3 and 4, due to missing key variables (i.e., at least one of the following variables were absent: breed (free text), sex (M/F/unknown), date of birth (DOB; MM/YYYY), first three characters of dog name (common, not pedigree name). Cleaned data represented 332 different purebreds^25,26^ and 1,071 crossbreds, incorporating: ‘Mix Breed’ i.e., lineage unknown or > 2 parental breeds; ‘Breed $\:{\rm\:X}$ x Breed $\:{\rm\:Y}$’; or ‘Breed $\:{\rm\:X}$ Cross/Type’. Microchip numbers were reported for 50.3% of dogs ($\:n$ = 2,201,805), and 0.02% of dogs had > 1 microchip numbers attached to their record ($\:n$ = 860).

### Population estimate

Using a hierarchical Bayesian approach accounting for imperfect detection probability, we estimated the posterior mean for the UK 2019 owned and unowned pet dog population as 12.64 million (95% CI 8.54–15.16 million) and median of 13.03 million (95% CI 8.51–15.24 million). Our estimates were compared to two existing datasets: (A) dog population estimate per postcode area reported by Aegerter et al.^3^ and (B) human population estimate per postcode area^27^ (Supplementary Fig. 1). Modelled estimates per postcode area agreed with the previous empirical estimate (Pearson’s cor = 0.83, df = 122, *p* < 0.001), with estimates both above and below modelled densities, suggesting an absence of bias in the predictions at extreme scales (i.e., small and large populations). This advocates for reliable regional and/or country level estimates, as these represent aggregated small-scale observations (Supplementary Fig. 1A). An asymptotic exponential function was expected and evident between dogs per postcode area and human population density (Supplementary Fig. 1B).

**Fig. 1:**
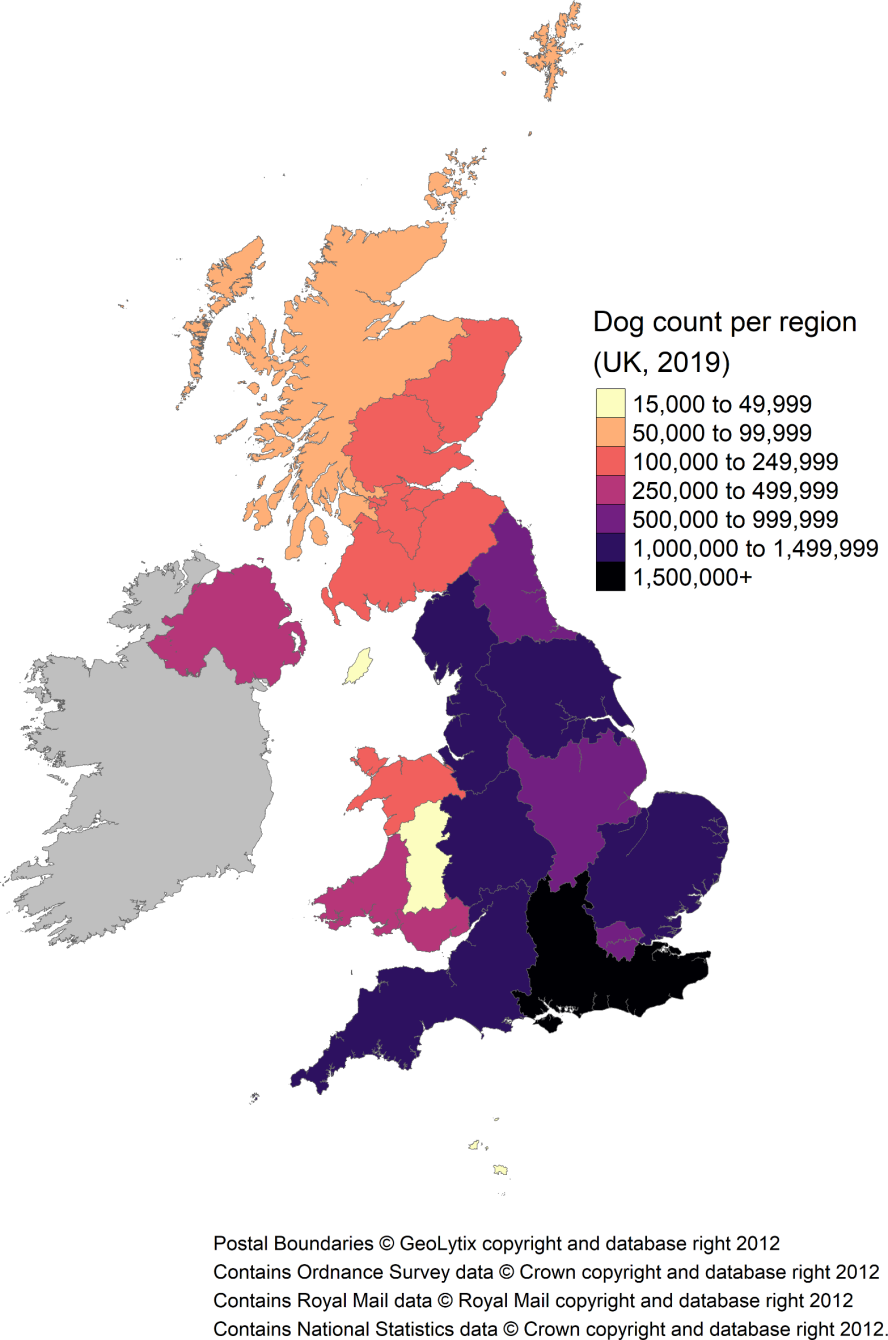
UK 2019 estimated mean dog population (i.e., marginal posterior distribution estimate) per region. Includes full population ($\:N$ = 12.64 million), i.e., all categories within: age; breed (pure and crossbred); cephalic index; and body size. See Table 1 for further detail.

**Fig. 2:**
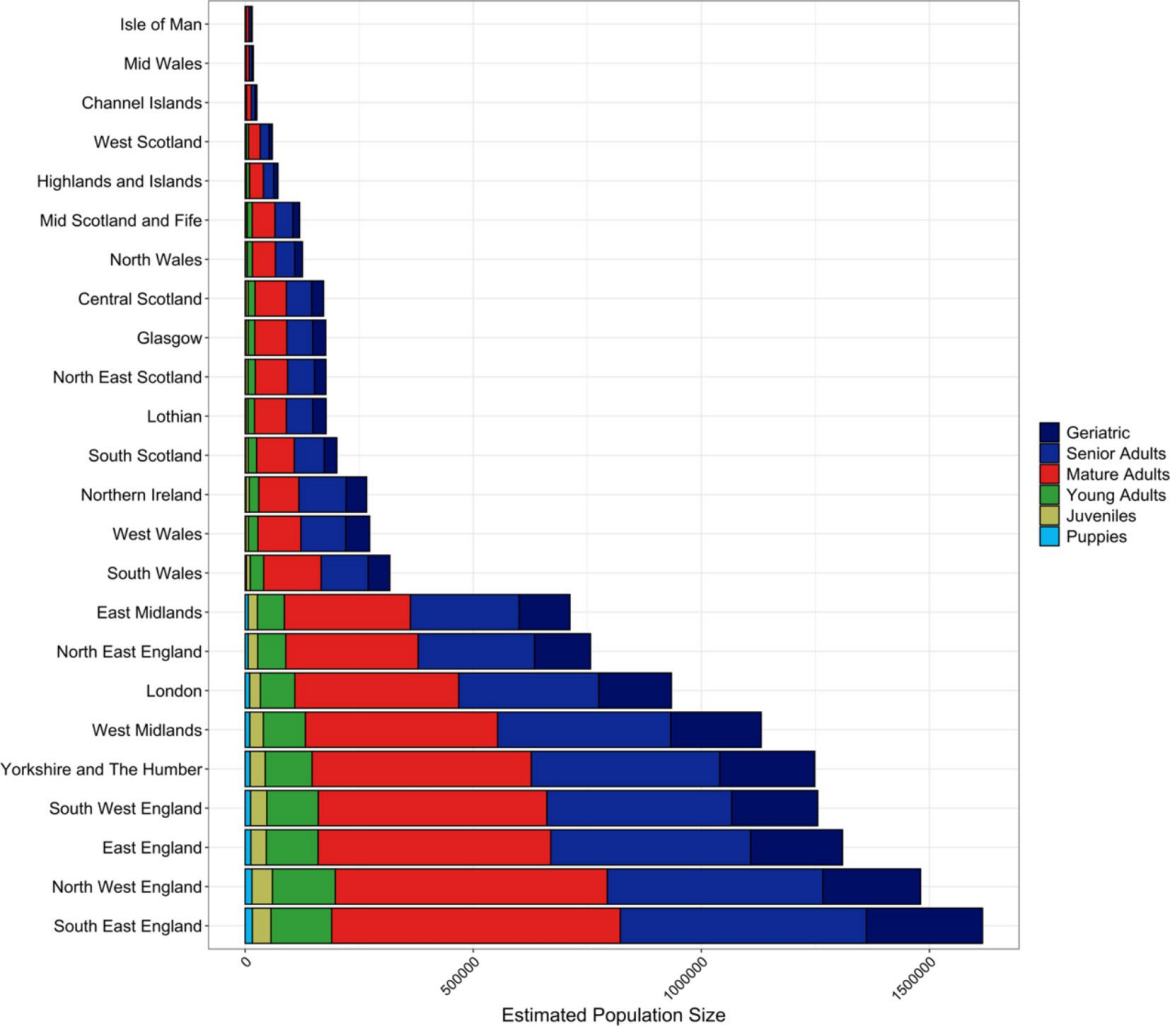
UK 2019 estimated dog population per region, with associated regional age demographics. See Table 1 and Supplementary Table 3 for further regional detail, and Table 2 for country-level proportions.

**Fig. 3:**
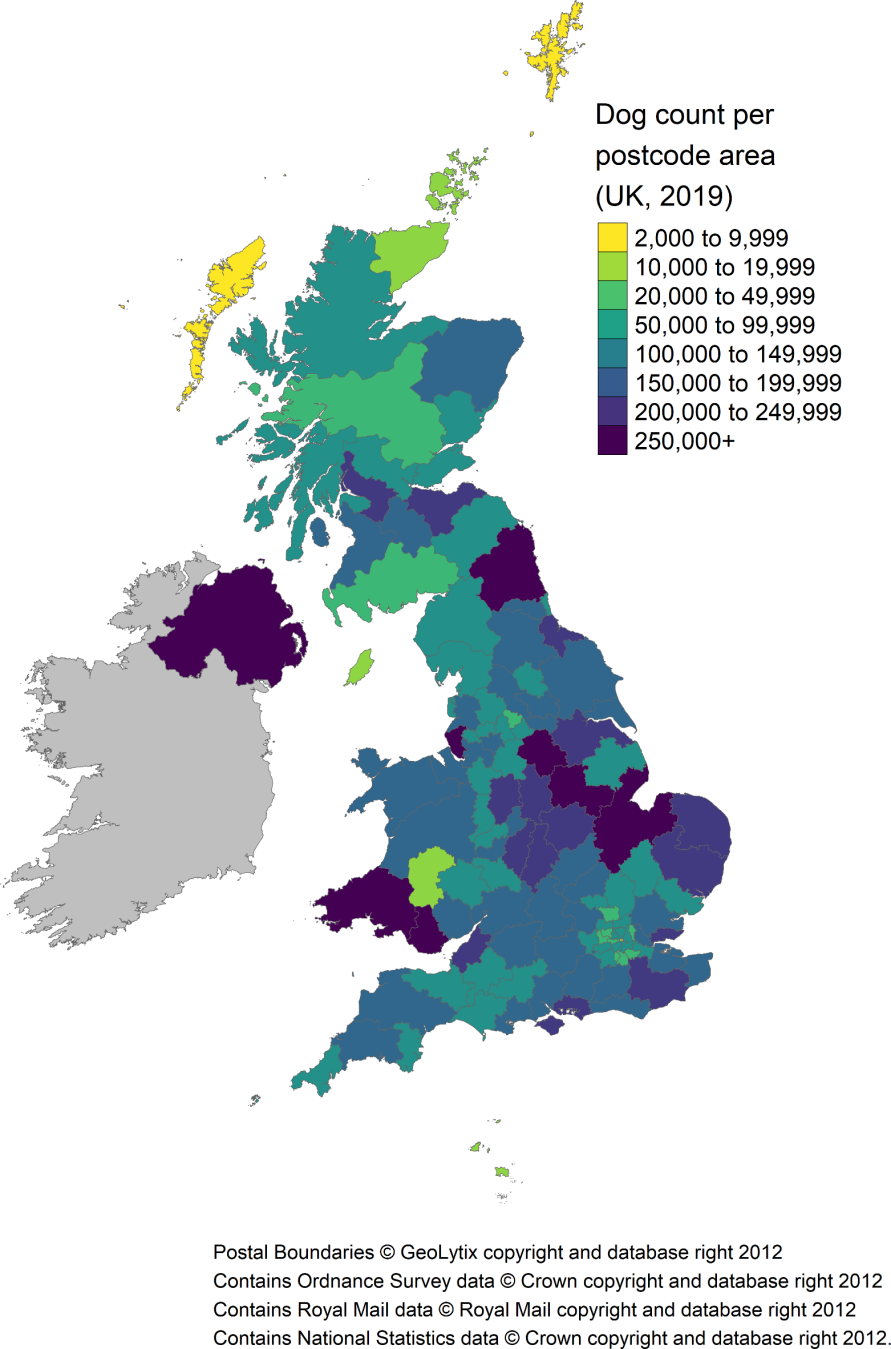
UK 2019 estimated mean dog population (i.e., marginal posterior distribution estimate) per postcode area. Includes full population ($\:N$ = 12.64 million), i.e., all categories within: age; breed (pure and crossbred); cephalic index; and body size. See Supplementary Table 1 and Supplementary Figs. 1 - 3 for further detail.

The estimated posterior mean of 12.64 million was used for all subsequent descriptive statistics. Within this estimate, 83.0% ($\:n$ = 10,486,868) were reported to reside in England, 2.1% in Northern Ireland ($\:n$ = 266,367), 9.1% within Scotland ($\:n$ = 1,155,625) and 5.8% within Wales ($\:n$ = 733,714). Regional population estimates are listed in Table 1 and visualized in Figs. 1 and 2. Over half of the UK dog population were located within the following 5/24 regions: South East England, North West England, East England, South West England and Yorkshire and The Humber. Predicted densities per postcode area are visualized in Fig. 3 (see Supplementary Table 1 and Supplementary Figs. 1 and 2 for predicted values, and Supplementary Fig. 3 for estimated dog population postcode specific detection probabilities).

**Table 1:** UK 2019 estimated dog population per region, ranked by increasing proportion of population (%).

Country	Region	Population estimate	Proportion of UK population (%)
England	Isle of Man	15520.46	0.1
Wales	Mid Wales	17965.07	0.1
England	Channel Islands	25684.64	0.2
Scotland	West Scotland	59596.50	0.5
Scotland	Highlands and Islands	71937.63	0.6
Scotland	Mid Scotland and Fife	119420.58	0.9
Wales	North Wales	125664.53	1.0
Scotland	Central Scotland	171967.22	1.4
Scotland	Glasgow	176846.42	1.4
Scotland	North East Scotland	177275.57	1.4
Scotland	Lothian	177538.97	1.4
Scotland	South Scotland	201042.12	1.6
Northern Ireland	Northern Ireland	266366.69	2.1
Wales	West Wales	272934.76	2.2
Wales	South Wales	317149.43	2.5
England	East Midlands	712073.71	5.6
England	North East England	757136.28	6.0
England	London	934592.18	7.4
England	West Midlands	1131374.62	8.9
England	Yorkshire and The Humber	1248692.27	9.9
England	South West England	1255197.70	9.9
England	East England	1309538.56	10.4
England	North West England	1480628.38	11.7
England	South East England	1616429.67	12.8

Includes full population ($\:N$ = 12.64 million), i.e., all categories within: age; breed (pure and crossbred); cephalic index; and body size. See Figs. 1 and 2 for further detail.

**Table 2:** UK 2019 country-level estimates for proportional age demographics (%); includes full population ($\:N$ = 12.64 million). Age group (AG) population estimate, per country, and associated proportional age group demographics both within (($\:N$_AG, country_/$\:N$_country_)*100) and between countries (($\:N$_AG, country_/$\:N$_total AG_)*100). Example: 15.9% of England’s population is within the geriatric development period (‘within country’) and 83.3% of the UK geriatric population can be found within England (‘between countries’).

Country	Age group	Population estimate	Proportion within country (%)	Proportion between countries (%)
England	Geriatric	1666510.22	15.9	83.3
Northern Ireland	Geriatric	45087.37	16.9	2.3
Scotland	Geriatric	169554.96	14.7	8.5
Wales	Geriatric	119948.62	16.3	6.0
England	Senior Adults	3464050.08	33.0	82.6
Northern Ireland	Senior Adults	103501.47	38.9	2.5
Scotland	Senior Adults	376283.1	32.6	9.0
Wales	Senior Adults	249816.03	34.0	6.0
England	Mature Adults	4082599.09	38.9	83.1
Northern Ireland	Mature Adults	88025.55	33.0	1.8
Scotland	Mature Adults	466961.86	40.4	9.5
Wales	Mature Adults	277568.4	37.8	5.6
England	Young Adults	890976.62	8.5	82.8
Northern Ireland	Young Adults	20910.21	7.9	1.9
Scotland	Young Adults	101189.27	8.8	9.4
Wales	Young Adults	63228.96	8.6	5.9
England	Juveniles	283332.9	2.7	83.5
Northern Ireland	Juveniles	6908.73	2.6	2.0
Scotland	Juveniles	31094.75	2.7	9.2
Wales	Juveniles	18107.53	2.5	5.3
England	Puppies	99399.54	0.9	85.0
Northern Ireland	Puppies	1933.35	0.7	1.7
Scotland	Puppies	10541.06	0.9	9.0
Wales	Puppies	5044.26	0.7	4.3

**Fig. 4:**
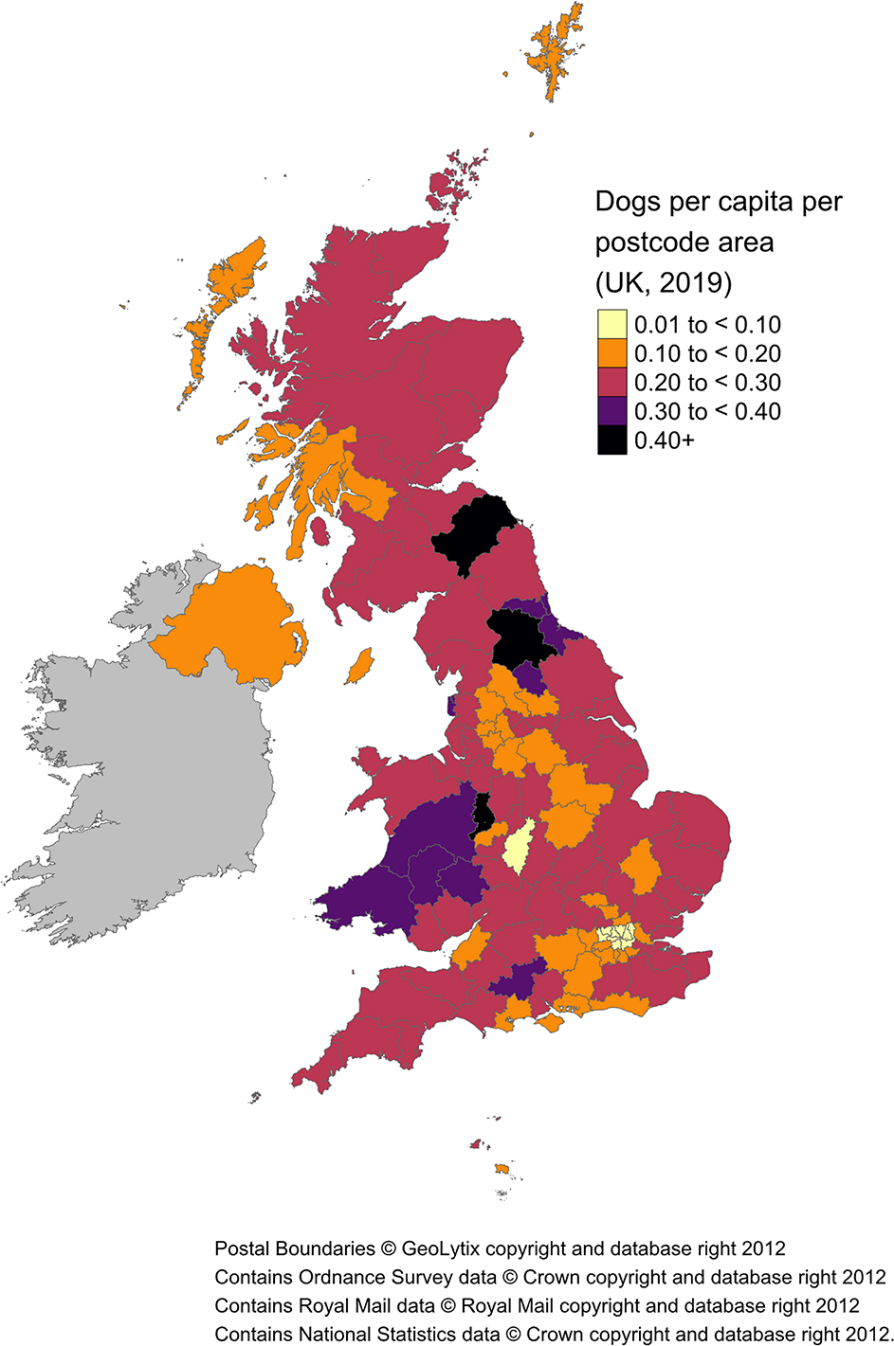
UK 2019 dogs per capita, per postcode area. See Supplementary Table 2 and Supplementary Fig. 4 for further detail. N.B. The estimate for the TD postcode area (i.e., Galashiels, Scotland) dog population is an outlier i.e., relatively high compared to the human population (posterior mean = 2.94). We advise interpreting the TD postcode area estimate with caution.

Mean dogs per capita, per postcode area, are presented in Fig. 4, with values and 95% credible intervals (CI) presented within Supplementary Table 2 and Supplementary Fig. 4. The greatest density of dogs per capita are located within: Telford, Darlington, Swansea, Harrogate, Llandrindod Wells and Sunderland. The lowest densities are located within 6 areas of London (London East, London Western Central, London Northern, London West and Paddington, London South Western and London Southall). The estimated dog population for TD postcode area (Galashiels, Scotland) is an outlier i.e., relatively high compared to the human population. We are confident that this is due to a ‘data in’ issue i.e., one of the eighteen raw data sources has a strong correction factor and a low customer base for TD: leading to an overcorrection of the final output. The 95% CI for the dog population within TD: 35,975 − 65,236 (Supplementary Table 1) incorporates a previously published estimate of 46,992^3^. Despite this, we advise interpreting this estimate with caution.

## Age demographics

In 2019, the UK canine age distribution was as follows: geriatrics 15.8% ($\:n$ = 1,998,180; ≥12 years), senior adults 33.2% ($\:n$ = 4,193,073; 7 to < 12 years), mature adults 38.9% ($\:n$ = 4,917,441; 2 to < 7 years), young adults 8.5% ($\:n$ = 1,077,512; 12 to < 24 months), juveniles 2.7% ($\:n$ = 339,548; 6 to < 12 months), and puppies 0.9% ($\:n$ = 116,821; 0 to < 6 months). Thus, 49% of the UK pet dog population was estimated to be within their senior or geriatric developmental period. Age categories sourced from Harvey^28^.

Proportional estimates for age demographics both within and between countries, are listed in Table 2. The greatest national proportion of geriatric and senior adult dogs was observed within Northern Ireland, contributing 16.9% and 38.9% of their total population, respectively. Scotland had the greatest national proportion of mature adult dogs at 40.4%. There was little within-country variation regarding proportional demographics of juveniles (range: 2.5–2.7%), puppies (range: 0.7–0.9%) and young adults (range: 7.9–8.8%). Between countries, England was found to home 82.6–85% of individuals within all age groups, followed by 8.5–9.5% in Scotland, 4.3-6.0% in Wales and 1.7–2.5% in Northern Ireland (Table 2).

Variation in age demographics were evident within and between regions (Fig. 2; Supplementary Table 3). Regional age demographic proportions varied most for mature adults (range: 33.0-42.8%), with Northern Ireland representing the lowest proportion and West Scotland representing the greatest. Geriatric and senior adult regional proportions also varied, ranging from 11.7% in West Scotland to 19.2% in West Wales, and 31.7% in the Highlands and Islands to 38.9% in Northern Ireland, respectively. Regional proportions of younger age groups did not vary as widely: young adults 7.6–9.9%; juveniles 2.1-3.0%, and puppies 0.4–1.1%. Between regions, South East England was found to home 12.0-13.5% of individuals within all age groups, followed by 10.7–13.2% in North West England, 9.9–10.7% in East England, 9.4–10.5% in South West England and 9.3–10.4% in Yorkshire and The Humber. The remaining 19 regions did not home > 10% of any age group (Supplementary Table 3).

## Breed popularity

An estimated 85.8% of the UK 2019 dog population were listed as purebred ($\:{n}_{E}$ = 10,843,010) represented by 331 recognised breeds^25,26^, whilst 14.2% were listed as crossbred ($\:{n}_{X}$ = 1,799,564), represented by 1,070 crossbreds. UK proportional breed demographics for purebreds and crossbreds are listed in Supplementary Tables 4 and 5, respectively. England exhibited the greatest breed diversity, representing 98.5% ($\:n$ = 326) of the total purebred variation available within the data, and 99% ($\:n$ = 1,059) of the total crossbred variation. In comparison, Northern Ireland, Scotland, and Wales represented 67.4% ($\:n$ = 223), 79.2% ($\:n$ = 262) and 74.9% ($\:n$ = 248) of the purebred variation; and 17% ($\:n$ = 182), 23.7% ($\:n$ = 254) and 23.1% ($\:n$ = 248) of crossbred variation, respectively (Supplementary Table 6). Estimated ratios between pure and crossbred dogs varied between countries: England = 6.04:1 ($\:{n}_{E}$ = 8,997,180; $\:{n}_{X}$ = 1,489,689); Northern Ireland = 8.3:1 ($\:{n}_{E}$ = 237,680; $\:{n}_{X}$ = 28,687); Scotland = 7.8:1 ($\:{n}_{E}$ = 1,024,710; $\:{n}_{X}$ = 130,915); and Wales = 8.1:1 ($\:{n}_{E}$ = 653,360; $\:{n}_{X}$ = 80,354).

The UK top 15 purebreds include: Labrador Retriever (10.2%), Cocker Spaniel (6.9%), Staffordshire Bull Terrier (4.7%), English Springer Spaniel (4.3%), German Shepherd (3.5%), French Bulldog (3.4%), Golden Retriever (2.7%), Pug (2.7%), Border Terrier (2.5%), Shih Tzu (2.4%), Cavalier King Charles Spaniel (2.4%), Jack Russell Terrier (2.4%), Bulldog (2.4%), West Highland White Terrier (2.1%) and Boxer (2.1%) (Supplementary Table 4). However, purebred popularity varied between countries (Supplementary Table 6). Labrador Retriever was the most popular breed in all countries, expect Northern Ireland where Miniature Schnauzer was the top choice. Additionally, Cocker Spaniel ranked second in England and Scotland, but ranked below Yorkshire Terrier and French Bulldog in Northern Ireland and Wales, respectively (Supplementary Table 6).

**Fig. 5:**
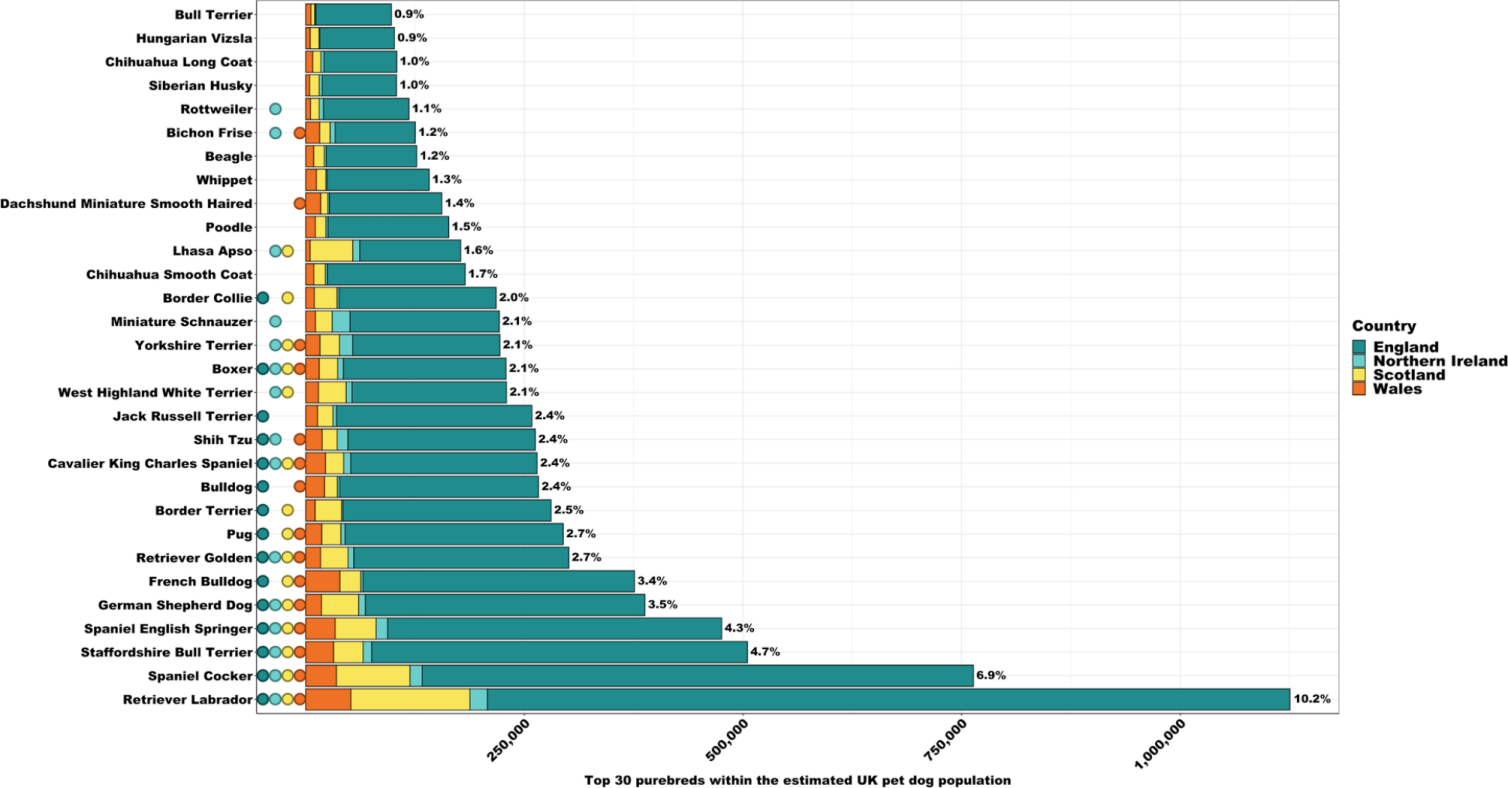
UK 2019 dog population estimates for the top 30 purebreds, subdivided by country. Percentage (%) relates to proportion of UK population represented by purebred. Circles represent presence of purebred within country’s (England, Northern Ireland, Scotland, and Wales) top 15 rankings. Notable variation in popularity across countries is evident, e.g., Jack Russell Terrier only apparent within England’s rankings, West Highland White Terrier apparent within Northern Ireland’s and Scotland’s rankings only, Miniature Schnauzer and Rottweiler within Northern Ireland’s ranking, and Dachshund Miniature Smooth Haired only present within Wales’s rankings. See Supplementary Tables 4 and 6 for further detail.

Population estimates for the top 30 purebreds within the UK, and across countries, are presented in Fig. 5. Within this top 30, 16.7% were classified as large breeds, 20% were medium sized, 60% were small breeds and one breed (‘Poodle’) could not be classified due to the combination of multiple sizes into one breed category. Furthermore, 30% of this top 30 were of brachycephalic type, 16.7% dolichocephalic, and 53.3% mesocephalic. This top 30 purebred rankings were in accordance with KC 2019^29^ breed registrations: 80% ($\:n$ = 24) of those listed within their top 30 were also apparent within our top 30. Those missing from the KC list, but apparent within ours included: Jack Russel Terrier (ranked 12/30), Yorkshire Terrier (ranked 16/30), Bichon Frise (ranked 25/30) and Siberian Husky (ranked 27/30). Additions to the KC list, but missing from our top 30, included: Pomeranian, Dobermann, Dogue de Bordeaux and Dachshund Miniature Long Haired^29^. These were ranked 35, 34, 31 and 51 (out of 332), within our data, respectively. Our purebred rankings were also similar to Pets4Homes^30^ popularity rankings: 69% ($\:n$ = 11) of those listed within their top 16 were also present within our top 16. Those excluded from the Pets4Homes top 16 list, but apparent within ours, included: Golden Retriever (ranked 7/16), Pug (ranked 8/30), Border Terrier (ranked 9/16), West Highland White Terrier (ranked 14/16), and Boxer (ranked 15/16). Additions to the Pets4Homes list, but excluded from our top 16, included: Dachshund Smooth Haired, Border Collie, Dachshund Miniature Smooth Haired, Pomeranian and Chihuahua Smooth Coat^30^. These were ranked 61, 18, 22, 35 and 19 (out of 331), within our data, respectively.

**Fig. 6:**
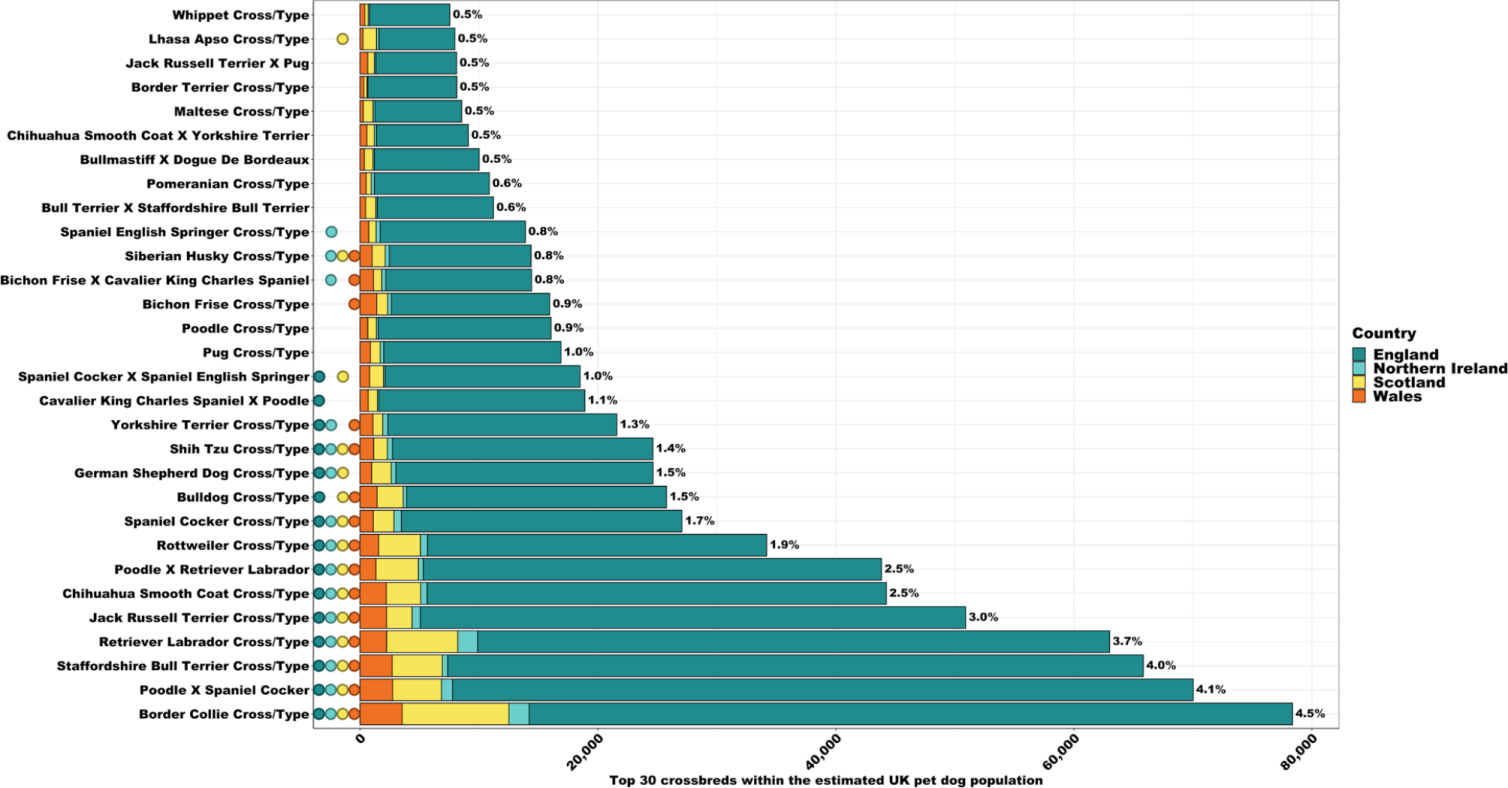
UK 2019 dog population estimates for the top 30 crossbreds, subdivided by country. ‘Mix breeds’ was omitted due to unknown lineage (represents 39.3% of the total crossbred population). Percentage (%) relates to proportion of UK population represented by crossbred. Circles represent presence of crossbred within country’s (England, Northern Ireland, Scotland, and Wales) top 15 rankings. Notable variation in popularity across countries is evident e.g., Cavapoo (i.e., Cavalier King Charles Spaniel x Poodle) only apparent within England’s rankings, Sprocker (i.e., Cocker Spaniel x English Springer Spaniel) apparent within England’s and Scotland’s rankings only, Cavachon (i.e., Bichon Frise x Cavalier King Charles Spaniel) within Northern Ireland’s and Wales’s rankings, English Springer Spaniel cross/type only present within Northern Ireland’s ranking, and Lhasa Apso cross/type only present within Scotland’s rankings. See Supplementary Tables 5 and 7 for further detail.

Within the crossbred population, 39.3% were compiled from ‘Mix Breeds’ i.e., lineage unknown or > 2 parental breeds. Excluding ‘Mix Breeds’, the subsequent top 15 crossbreds included: Border Collie cross/type (4.5%), Cockerpoo (i.e., Cocker Spaniel X Poodle; 4.1%), Staffordshire Bull Terrier cross/type (4.0%), Labrador Retriever cross/type (3.7%), Jack Russell Terrier cross/type (3.0%), Labradoodle (i.e., Labrador Retriever x Poodle; 2.5%), Chihuahua Smooth Coat cross/type (2.5%), Rottweiler cross/type (1.9%), Cocker Spaniel cross/type (1.7%), Bulldog cross/type (1.5%), German Shepherd cross/type (1.5%), Shih Tzu cross/type (1.4%), Yorkshire Terrier cross/type (1.3%), Cavapoo (i.e., Cavalier King Charles Spaniel X Poodle; 1.1%) and Sprocker (i.e., Cocker Spaniel x English Springer Spaniel; 1.0%, Supplementary Table 5). However, variation in crossbred popularity was evident between countries (Supplementary Table 7). Excluding ‘Mix Breeds’, Border Collie cross/type held top rank in all countries, followed by Labrador Retriever cross/type in Northern Ireland and Scotland, and Cockerpoo (i.e., Cocker Spaniel X Poodle; 4.1%) in England and Wales (Supplementary Table 7). Population estimates for the top 30 crossbreds (excluding ‘Mix Breeds’) within the UK, and across countries, are presented in Fig. 6.

## Cephalic index

An estimated 22.5% of the UK 2019 purebred population were listed as brachycephalic breeds ($\:n$ = 2,843,714), 61.8% mesocephalic ($\:n$ = 7,813,435) and 15.7% dolichocephalic ($\:n$ = 1,985,426). Cephalic index categories were sourced from O’Neill et al.^31^. However, variation in proportional cephalic demographics were evident within and between countries (Table 3). Whilst the majority of all country-level populations consisted of mesocephalic breeds (range: 56.6–66.0%), Wales represented the greatest proportion of within-country brachycephalic and dolichocephalic populations at 26.9% and 16.5% respectively, in comparison with England (22.4%, 16.0%), Northern Ireland (23.1%, 11.7%) and Scotland (20.5%, 13.5%). Between countries, England was found to home 82.6–84.5% of individuals within all cephalic index groups, followed by 7.8–9.8% in Scotland, 5.3–6.9% in Wales and 1.6–2.2% in Northern Ireland (Table 3).

**Table 3:** UK 2019 country-level estimates for proportional cephalic index (CI) demographics (%); includes full population ($\:N$ = 12.64 million).

Country	Cephalic index	Population estimate	Proportion within country (%)	Proportion between countries (%)
England	Brachycephalic	2347705.98	22.4	82.6
Northern Ireland	Brachycephalic	61600.04	23.1	2.2
Scotland	Brachycephalic	237087.93	20.5	8.3
Wales	Brachycephalic	197319.59	26.9	6.9
England	Mesocephalic	6461684.41	61.6	82.7
Northern Ireland	Mesocephalic	173648.6	65.2	2.2
Scotland	Mesocephalic	763099.61	66.0	9.8
Wales	Mesocephalic	415002.07	56.6	5.3
England	Dolichocephalic	1677478.07	16.0	84.5
Northern Ireland	Dolichocephalic	31118.05	11.7	1.6
Scotland	Dolichocephalic	155437.46	13.5	7.8
Wales	Dolichocephalic	121392.13	16.5	6.1

Cephalic index population estimate, per country, and associated proportional cephalic index demographics both within (($\:N$_CI, country_/$\:N$_country_)*100) and between countries (($\:N$_CI, country_/$\:N$_total CI_)*100). Example: 22.4% of England’s population are listed as brachycephlic (‘within country’) and 82.6% of the UK brachycephalic population can be found within England (‘between countries’).

**Fig. 7:**
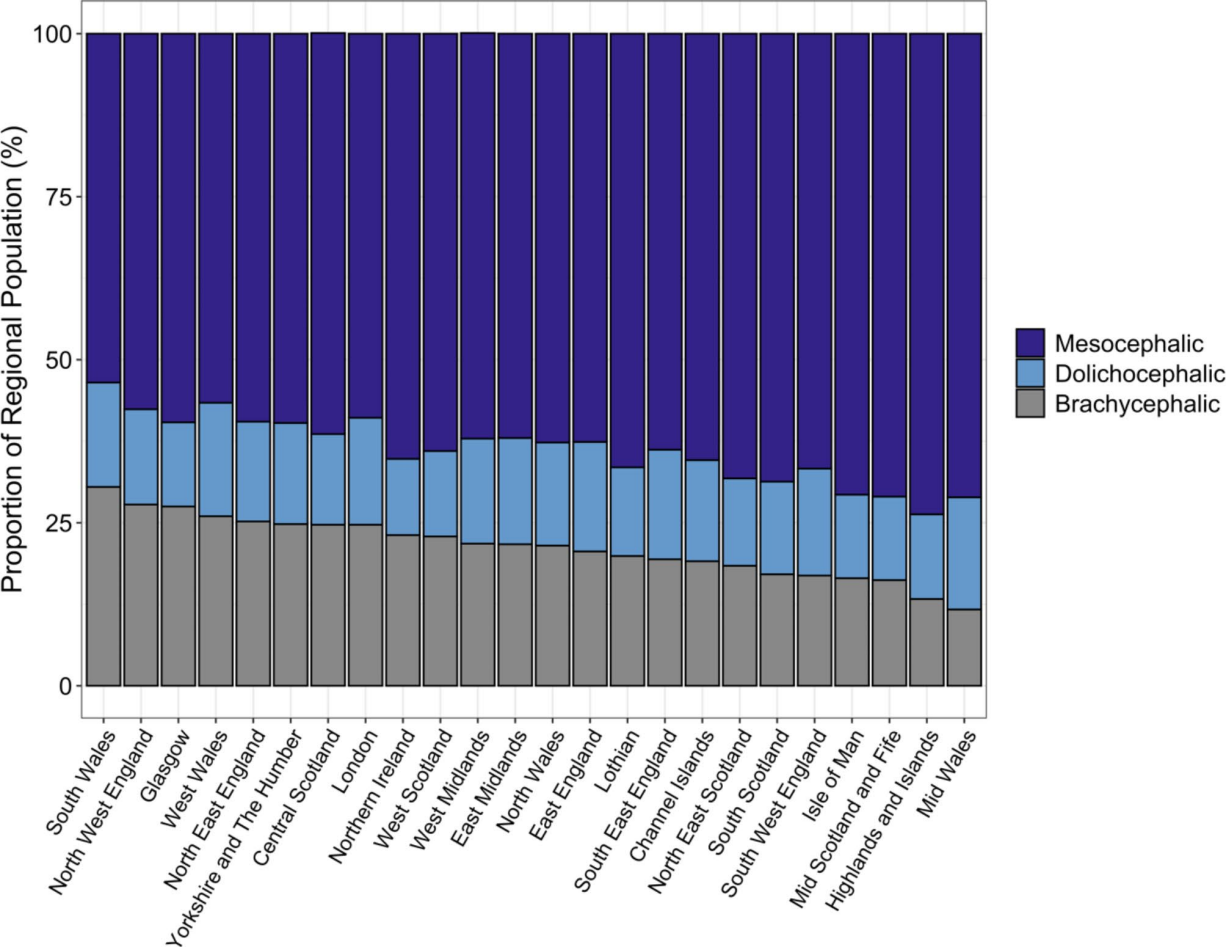
UK 2019 regional proportional demographics of each cephalic index: mesocephalic, dolichocephalic, and brachycephalic. See Supplementary Table 8 for further regional detail, and Table 3 for country-level proportions.

Cephalic demographics also varied amongst regions (Fig. 7; Supplementary Table 8). Mesocephalic proportions ranged from 53.5 to 73.7%, with South Wales representing the lowest proportion and Highlands and Islands representing the greatest. Regional brachycephalic proportions ranged from 11.7 to 30.5%, with Mid Wales representing the lowest and South Wales representing the highest, whilst Dolichocephalic proportions did not vary as broadly, ranging from 11.7 to 17.4% (Fig. 7; Supplementary Table 8).

## Body size

In 2019, 52.5% of the UK canine population were estimated to be small in body size ($\:n$ = 6,633,421), with 29.3% ($\:n$ = 3,711,087) listed as large and the remaining 18.2% medium sized ($\:n$ = 2,298,066). Body size categories were sourced from KC^25^ and Fédération Cynologique Internationale^26^. Whilst this pattern remained nationally consistent, proportional estimates for size demographics varied within and between countries (Table 4). Scotland presented the lowest national proportion of small dogs, at 51.1% of the total population, in comparison with England = 52.2%, Northern Ireland = 59.2%, and Wales = 56.6%. Scotland also presented the greatest proportion of large dogs, contributing 32.2% of the total population, in comparison to England = 29.4%, Northern Ireland = 26.4% and Wales = 25.3%. England exhibited the greatest proportion of medium sized dogs at 18.4%, followed by Wales = 18.1%, Scotland = 16.7% and Northern Ireland = 14.3% (Table 4). Between countries, England was found to home 82.5–84.2% of individuals within all body size groups, followed by 8.4–10.0% in Scotland, 5.0-6.3% in Wales and 1.7–2.4% in Northern Ireland (Table 4).

**Table 4:** UK 2019 country-level estimates for proportional body size (BS) demographics (%); includes full population ($\:N$ = 12.64 million).

Country	Body size	Population estimate	Proportion within country (%)	Proportion between countries (%)
England	Large	3083122.4	29.4	83.1
Northern Ireland	Large	70419.97	26.4	1.9
Scotland	Large	371951.04	32.2	10.0
Wales	Large	185593.49	25.3	5.0
England	Medium	1934250.91	18.4	84.2
Northern Ireland	Medium	38184.53	14.3	1.7
Scotland	Medium	193014.73	16.7	8.4
Wales	Medium	132616.16	18.1	5.8
England	Small	5469495.16	52.2	82.5
Northern Ireland	Small	157762.19	59.2	2.4
Scotland	Small	590659.24	51.1	8.9
Wales	Small	415504.14	56.6	6.3

**Fig. 8:**
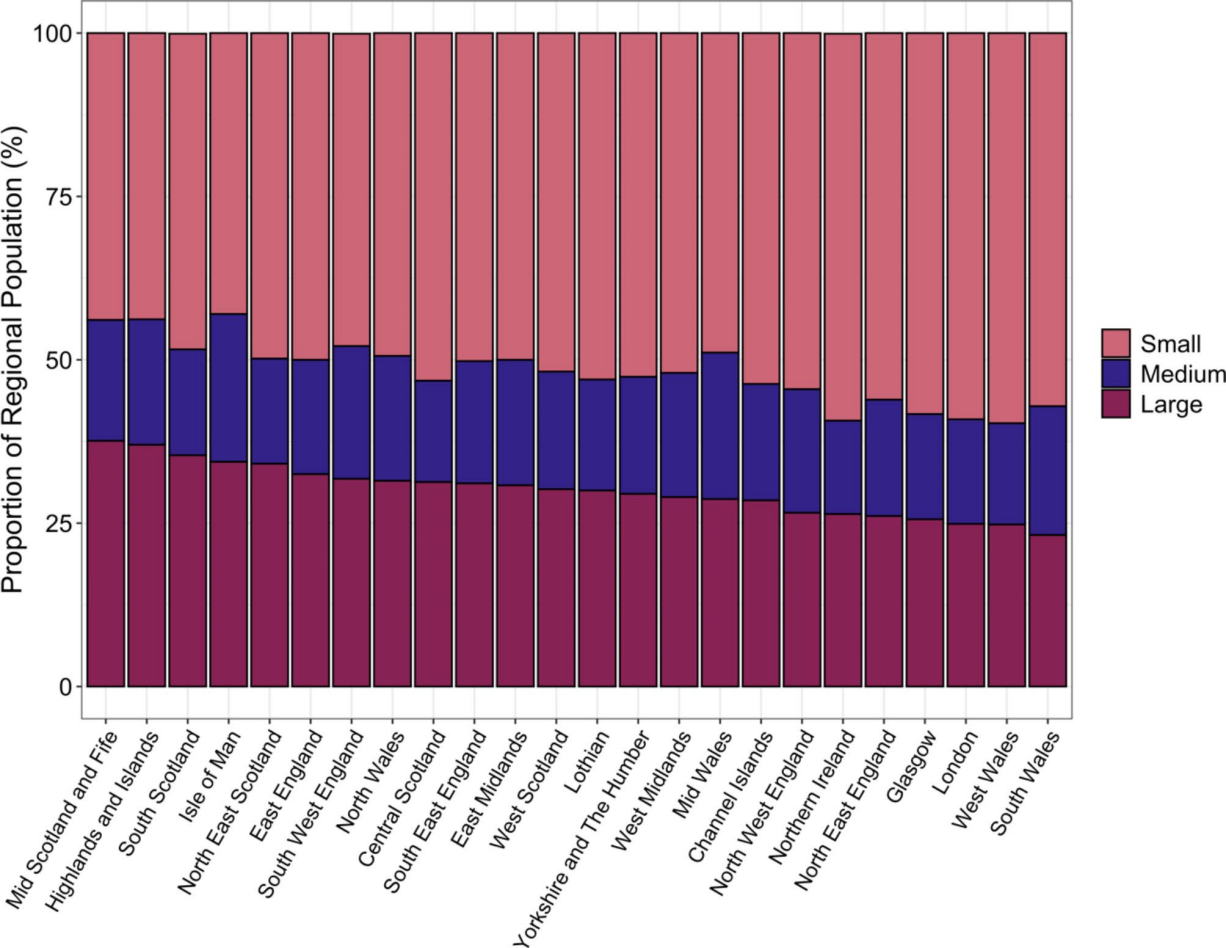
UK 2019 regional proportional demographics of each body size: small, medium, and large. See Supplementary Table 9 for further regional detail, and Table 4 for country-level proportions.

Body size population estimate, per country, and associated proportional body size demographics both within (($\:N$_BS, country_/ $\:N$_country_)*100) and between countries (($\:N$_BS, country_/ $\:N$_total BS_)*100). Example: 29.4% of England’s population are listed as large breeds (‘within country’) and 83.1% of the UK large-breed population can be found within England (‘between countries’). See Fig. 8 for further detail.

Variation in size demographics were also evident amongst regions (Fig. 8; Supplementary Table 9). Regional demographic proportions varied most for the small sized population, ranging from 43.0 to 59.7%, with Isle of Man representing the lowest and West Wales representing the highest proportions. Large sized populations also varied between regions, ranging from 23.2% (South Wales) to 37.6% (Mid Scotland and Fife). Medium sized populations did not range as widely, ranging from 14.3% in Northern Ireland to 22.6% in Isle of Man (Fig. 8; Supplementary Table 9).

## Discussion

We provide an estimate and national description of the density and distribution of owned and unowned pet dogs (i.e., those housed long term by animal welfare charities) in 2019, at varying spatial scales across the UK. We estimate that there were 12.64 million (mean, 95% CI 8.54–15.16 million; median, 13.03 million, 95% CI 8.51–15.24 million) pet dogs, with 85.8% of this total represented by purebreds. For comparison, previous estimates for 2019 have been suggested at 9.0^18^ and 9.9 million^32^. We provide details regarding spatial descriptions of demographic factors that directly influence population dynamics, such as breed, age, cephalic index, and body size^23,33,34^. As far as the authors are aware, we have generated a population estimate from the most comprehensive UK pet dog dataset to date, via a collaborative network including a breed registry, veterinary corporations, pet insurance companies, animal welfare charities and an academic institution.

It is important to note, that the 12.64 million (95% CI 8.54–15.16 million; median, 13.03 million, 95% CI 8.51–15.24 million) estimate is temporally explicit, i.e., assumes no net migration, death, birth etc. Estimates with comparable time points include UK Pet Food 2018–2019 and 2019–2020 statistic of 9.0 million for both periods^18^, and PDSA 2019 statistic of 9.9 million^32^. UK Pet Food also released estimates for 2021, 2022 and 2023, at 12.0, 13.0 and 12.0 million, respectively^1,18^; whilst PDSA suggested 9.6, 10.2 and 11.0 million, respectively^35–37^. These previous statistics remain lower, or equivalent to, our 2019 estimate of 12.64 million, suggesting that the scientific community may have been underestimating the UK dog population. The most prominent consequence is that inaccuracies in these estimates are then propagated forward into extrapolated statistics, such as regional densities, annual population growth and proportional demographics. For example, based on prior population and survival estimates^18,38–41^, it was previously suggested that the UK required 750,000-850,000 puppies be born each year, to maintain the population size^13,42^. Applying our population estimate and assuming an unchanged UK median mortality of 12.0^43^ or 12.5^44^ years, we suggest the number of dogs needed to maintain the population size as 1.01–1.05 million dogs annually. This amplified figure highlights the pressure on the existing puppy ‘supply chain’.

Variations in popularity of dog breeds are often evident as large fluctuations, that arise from fashions and fads^45,46^. An acute increase in breed demand and impulse buying^5,6^ have resulted in puppies becoming lucrative commodities in an industry driven by profitability, often at the expense of canine welfare^7^. Here, we list UK breed popularity overall, and within England, Northern Ireland, Scotland, and Wales. Our popularity rankings were broadly in accordance with the KC 2019 breed registration statistics^29^ and Pets4Homes popularity rankings^30^, indicating that our data reliably represents UK breed demographics. However, differences in breed popularity were observed among the three rankings. Small terriers e.g., Border Terriers, Jack Russell Terriers, West Highland White Terriers, and Yorkshire Terriers were more popular within our data, while Dachshunds, Pomeranians, and Chihuahuas proved more popular in both external rankings (KC^29^ and Pets4Homes^30^). As both external lists are linked to the market supply of dogs via breed registry and online sales, they likely include a higher proportion of younger age groups (young adults, juveniles, and puppies)^14^. Consequently, the discrepancy between our UK breed popularity rankings and the external lists may suggest a potential decline in demand for small terriers and a surge in popularity for Dachshunds, Pomeranians, and Chihuahuas. Future research should monitor ongoing breed demographic changes and consider how rapid population growth may impact canine welfare.

The popularity of brachycephalic (flat-faced) breeds has been increasing internationally, despite the scientific evidence highlighting significant health and welfare challenges associated with this conformation^24,47,48^. Fashion (social influence) has been suggested to be more important than function in determining the popularity of dog breeds^49^. Whilst regional and country level variation in brachycephalic popularity is evident within our data: overall, nine of the top thirty UK popular breeds are considered brachycephalic (French Bulldog, Bulldog, Pug, Cavalier King Charles Spaniel, Shih Tzu, Boxer, Chihuahua Smooth Coat, Chihuahua Long Coat and Lhasa Apso^31^). Consequently, it is imperative that we continue to monitor breed popularity patterns^50^, in order to ensure the welfare of “fashionable” breeds^51^ are not compromised by unscrupulous breeders who are capitalizing on consumer demand^7^. There needs to be a fundamental shift in the way dogs are selected for breeding, refocusing on canine health, welfare, functionality, and behaviour^52,53^ and disconnecting from selection pressure towards phenotypic exaggeration to achieve breed standards.

The UK recorded a rapid increase in puppy purchasing during the COVID-19 pandemic^2,54–56^ with many deciding to purchase a puppy for the first time^57^. Sharp increases in web interest regarding the adoption and sale of dogs was reported^5,58^, with online pet supply companies reporting increased sales^59^ and dramatic price increases, e.g., 131% average increase for dogs in 2020 versus 2019^30^. There is increasing concern for the welfare of these ‘pandemic puppies’ following changes in the management of COVID-19: restricting movement and reducing time-sensitive exposure to key environmental and social stimuli during their critical developmental period^60,61^. Pet owners were found to experience unique hardships related to changes in everyday life from the COVID-19 pandemic^58,62^. A reported 3.4 million UK households have confirmed the relinquishment of a pet since the start of the pandemic, with 60% of that figure represented by pet dogs^63^. Furthermore, 26% of UK dog owners stated the development of at least one behavioural problem as a result of lockdown measures^61^, prompting fears of a surge in relinquishment, abandonments and euthanasia. These factors may have altered the density and distribution the UK pet dog population. Accordingly, as previously stated, UK Pet Food have reported an increase in the population from 9.0 million in 2019–2020, to 12.0, 13.0 and 12.0 million in 2021, 2022 and 2023, respectively^1,64^. In contrast, PDSA have suggested that there has not been a statistically significant increase in the estimated pet population size^35^. In order to interrogate differences in the UK pet dog population over time, we plan to repeat the process described in this paper and provide an ongoing estimate. This will allow researchers and stakeholders to better understand any changed patterns in dog ownership due to specific events, such as the introduction of new regulations, legislature, or the onset of a global pandemic. Furthermore, future estimates will allow us to validate the process described in this paper, via multi-year assessment.

It is vital that we continue to accurately quantify the UK pet dog population size, as the analytical benefits of these outcomes are far reaching, with respect to both human and canine health and welfare. These results provide analytical value to veterinary and epidemiological research, disease control and contingency response, ecological and environmental impact, policy development and implementation, and public health. Furthermore, previous research has attempted to produce national estimates of pet population size by incorporating human-related factors that may influence pet ownership, such as owner age, household size, education/profession, income, rural location etc^20,21,65–67^. Here, we provide the spatiotemporal dataset required to reverse this approach and elucidate socioeconomic factors that influence pet dog population density and breed popularity, including pure versus crossbred lineage, cephalic index, and body size.

The data presented in this paper may contain knowledge gaps or biases, due to human socioeconomic demographics, human reporting error, or poor representation of subpopulations. For example, due to the participants involved, data were unlikely to include laboratory dogs and/or racing greyhounds. Additionally, 49% of the UK pet dog population were estimated to be within their Senior or Geriatric developmental period (≥7 years), with only 0.9%, 2.7% and 8.5% of the population represented by Puppies (0 to < 6 months), Juveniles (6 to < 12 months) and Young Adults (12 to < 24 months), respectively^28^. This suggests a potential under-representation of younger age groups, likely due to delayed registration of young dogs on the compiled data sources. Future studies should consider sourcing datasets which predominantly represent younger age groups, e.g., online advertisements for puppies and registered puppy training classes. It has been estimated that 77% of the UK pet dog population are registered with a veterinary practice, 42–46% of UK pet owners have health insurance for their animals, and 29% are KC registered^22^. Thus, our dataset potentially captures a large proportion of the UK pet dog population. However, for a dog to appear within these datasets, a financial contribution is normally required from the owner (in most cases), which some cannot afford. In the hope of representing the remaining subpopulation, the authors incorporated data from animal welfare charities, i.e., ‘unowned’ dogs. Furthermore, as previously mentioned, there appears to be an absence of free-living unowned dogs in the UK i.e., ‘stray’ dogs. Instead, strays are reintegrated into the owned dog population through reunification with their previous owners^3^ or through the care and support of animal welfare organizations^2,4^. As such, we believe the dataset provides a comprehensive proxy for the density and distribution of dogs within the UK.

Quantitative model validation was carried out by comparing the population estimates reported here, with dog population estimates presented by Aegerter et al.^3^ (Supplementary Fig. 1A). This aspect of the study may be considered a limitation for two reasons. First, the model by Aegerter et al. is based on pet population size estimates from data collected in 2011^21^, leading to an 8-year gap between the provided population estimate and the model validation. Second, the accuracy of the Aegerter et al. estimates are not optimal, as they are derived from surveys and extrapolation (spatially and categorically subdivided across ownership classes)^3^. Despite these limitations, the data from Aegerter et al. remains the most appropriate spatial dataset for validating the model, and therefore, we advocate for its inclusion in the study.

As human population density is included within the N-mixture model as a predictor of detection probability and therefore dog population size, readers must consider the per capita output with caution. Furthermore, we do not provide details regarding proportion of households with dogs or the number of dogs per owner. However, as Asher et al.^22^ described geographical variation in both variables, future studies may wish to consider incorporating these measures as potential priors. We recommend that future studies focus on a national-scale overview regarding UK pet dog population dynamics: allowing for the development of a stochastic transitionary state model. By including parameterised compartments and sub-populations, e.g., market sources, emigration/immigration, and birth/death rates, we can start to identify and evaluate unrepresented sub-populations; gaps in data coverage; substandard regulations; and importantly, the potential impact of legislative change.

## Conclusion

The UK puppy market is vast: we estimated the number of puppies required just to replace the existing ageing population to be around 1 million in 2019. The combined sales of puppies in England, Scotland and Wales are estimated to be worth ~£130 million per annum^68^. The estimated 12.64 million dogs in the UK in 2019 are of huge importance in terms of societal influence as well as the economy. Understanding the spatial and temporal distribution of dogs across the UK therefore has numerous benefits, such as informing dog related service and charitable provision.

## Methods

### Data: sources, cleaning, and deduplication

Dogs Trust data were merged with datasets from 17 project participants, totaling 18 data sources. Participants represented various industries, including a breed registry (The Kennel Club (KC) - UK), veterinary corporations (PDSA; Medivet; Vets4Pets), pet insurance companies (The Insurance Emporium; NCI Insurance; Cardif Pinnacle; Agria Pet Insurance Ltd; Direct Line), animal welfare charities (Battersea Dogs and Cats Home; Blue Cross; SSPCA; Raystede; Wood Green, The Animals Charity; Edinburgh Dog and Cat Home; PDSA; Mayhew), and an academic institution (SAVSNET - Small Animal Veterinary Surveillance Network, University of Liverpool). Data requested included all individuals (owned and unowned, where applicable) recorded up to and including December 31, 2019. All data were obtained from original registries and not through surveys. This data established the ‘closed’ population for the mark–recapture modelling framework. The year 2019 was selected as year of study, as the project was initiated in 2020, and these results serve as proof of concept for ongoing UK dog population estimates. Consequently, delay between project initiation and publication may be attributed to developing reproducible pipelines related to data access, structure, cleaning, and modeling.

Variables requested from project participants included: breed (free text), crossbred (Y/N/unknown), sex (M/F/unknown), date of birth (DOB; MM/YYYY), postcode area (i.e., first one or two characters), first three characters of dog name (common, not pedigree name), last six characters of microchip number, last six characters of additional microchip number (if more than one known), status (alive/dead) and termination date (death or end of policy due to death; DD/MM/YY). Not all canine-centric variables were sent by project participants. Longevity (age of dog in decimal years) reflects the period between the DOB and termination date (if deceased), or date data were received (if assumed alive). Within data sources, under-sampled postcode areas do occur i.e., some data sources record no or few dogs at a location where others record many. This among-survey variation at the postcode area level is corrected by the detection probability model (discussed below), which uses information on the human population size to inform likely dog population size.

Data cleaning included the removal of non-canids, classifying all individuals into (1) The Kennel Club (KC)^25^ and/or Fédération Cynologique Internationale (FCI)^26^ recognised ‘Purebred’ breeds i.e., parental lineage = 1 breed, or (2) ‘Crossbred’ breeds i.e., parental lineage ≥ 2 breeds, which included ‘Mix Breed’ i.e., lineage unknown or > 2 parental breeds, ‘Breed $\:{\rm\:X}$ x Breed $\:{\rm\:Y}$’, ‘Breed $\:{\rm\:X}$ Cross/Type’. Purebreds within the dataset may be included in one or both breed reference lists^25,26^. Due to variation in reporting, alternative breed names were collapsed into one breed category based on known ancestry and/or breed popularity, i.e., historical breed registration statistics^29^. These changes, listed in Supplementary Table 10, were established via majority agreement by canine behaviour and research experts at Dogs Trust. Individuals were grouped into one of six age groups: ‘Puppies’ aged 0 to < 6 months, ‘Juveniles’ aged 6 to < 12 months, ‘Young Adults’ aged 12 to < 24 months, ‘Mature Adults’ aged 2 to < 7 years, ‘Senior’ aged 7 to < 12 years and ‘Geriatric’ aged ≥12 years. These groupings were developed by Harvey^28^ to capture age-related developmental trajectories for the majority of dog breeds. Body size per purebred, i.e., small, medium and large, were obtained from KC^25^ and FCI^26^ grey literature and cephalic index i.e., the percentage of skull breadth to length, per purebred, were obtained from O’Neill et al.^31^ and include brachycephalic (flat-faced), mesocephalic or dolichocephalic (long-faced; Supplementary Table 10).

Postcode area was assigned to one of the following twenty-four regions, determined by the UK National Statistics Postcode Directory^69^: Highlands and Islands, North East Scotland, Central Scotland, Glasgow, Lothian, Mid Scotland and Fife, South Scotland, West Scotland, Northern Ireland, North East England, North West England, Yorkshire and The Humber, East England, East Midlands, West Midlands, London, South East England, South West England, Isle of Man, Channel Islands, North Wales, Mid Wales, West Wales, South Wales (Supplementary Table 11).

As data were obtained from multiple sources, duplication of an individual across data sources was probable. Deduplication consisted of a four-phase process outlined within Supplementary Note 1: whereby duplicate individuals were limited to one entry (prior to downstream analyses). Full implementation of the deduplication workflow is available as Source Code 1 (10.6084/m9.figshare.24534151.v2). For the purposes of this study, deduplicated data were then subset to rows where the following variables were complete: crossbred (Y/N); status (alive/dead) and sex (M/F). Due to the expansive nature of the dataset, and the absence of a mandate to report a dog’s death, data were limited to dogs aged ≤ 18.3 years, i.e., the age at which 95% of the UK’s pure and crossbred pet dog population were found to be deceased^24^. This measure was taken to ensure that the data only included dogs that were alive in 2019, thereby preventing artificial inflation of the resulting population estimate caused by the inadvertent inclusion of deceased dogs.

Data did not include accompanying information regarding the owner. However, data did include human population density, as pet dogs are inherently where owners are present. The UK 2011 human population census data were obtained from the Office for National Statistics^27^, which incorporates censuses undertaken by the ONS in England and Wales^70^, National Records of Scotland^71^, and the Northern Ireland Statistics and Research Agency^72^. All downstream analyses utilised human population data from the 2011 census, as 2019 devolved census data was not available for all nations at time of analysis. Despite there being previous dog estimates^3,20–22^, we did not include these data. However, we applied Aegerter et al.^3^ dog population per postcode area estimates for model checking, along with human population estimates per postcode area^27^. Rankings regarding breed popularity were extracted from: (1) Pets4Homes^30^, which details numbers of buyers per puppy, regarding the sale of dogs from their online marketplace; and (2) KC 2019 breed registry statistics^29^.

### Statistical analyses

All analyses were conducted using the statistical programming software R version 4.0.4 (2021-02-15)^73^. We used a hierarchical Bayesian N-mixture model^74^ to estimate postcode area specific population size, in a closed mark–recapture framework, using data from all 18 sources. N-mixture models allow estimation of population sizes whilst accounting for imperfect detection, i.e., the fact that the dataset will not include all dogs present in the UK and therefore likely be an underestimate. Here we treat each of the 18 data sources are separate ‘sampling occasions’. This allows us to estimate the true (unobserved) population size at postcode area as a function of the total number of dogs recorded in the dataset corrected for differential detection probability across surveys at each postcode. First, we assumed that our deduplicated estimates of number of dogs as postcode *i* (N_i_) were drawn from a Poisson distribution with latent abundance *λ*.

$$\:{N}_{i}\:\sim\:\:Poisson\left(\lambda\:\right)$$

Second, we assumed that our observed counts at each combination of *i* postcodes and *j* replicate data sources (y_i, j_) arise with a detection probability *p* described by the Binomial distribution

$$\:{y\:}_{i,j}|\:{N}_{i}\:\sim\:\:Binomial({N}_{i},\:{p}_{i,j})$$

Here we modelled the detection probability at each postcode-survey combination as a function of human population size at that postcode area, using random intercepts and slopes for each survey. That is, we expect lower detection probability in more populous postcodes, and that each data source will vary in its ability to correctly enumerate then number of dogs in a postcode. The latter could occur for several reasons, including market share for commercial data sources, alongside variation in coverage or completeness of records.

$$\:logit\left({p}_{ij}\right)=\:{\alpha\:}_{j}+\:{\beta\:}_{j}*{human\:population}_{i}$$

We initially modelled random intercepts and slopes as correlated, arising from a multivariate Normal distribution. However, this led to convergence issues, and so the final model included uncorrelated intercepts and slopes. Uncorrelated random effects make the assumption that there is no relationship between the intercept value and slope value at a given postcode. For example, larger means (intercept) would not automatically be associated with larger/more positive slopes (for a positive correlation), and so these two quantities are free to vary independently.

This model included a 124 row (postcode area) x 18 column (data sources) matrix as a response, representing the individual deduplicated population size estimates for all postcode area-data source combinations. Models were run for 100,000 iterations with a thinning interval of 50 following a burnin of 10,000^75^. Flat uniform prior distributions with support from zero to one were chosen for detection probabilities, across the multiple data sources (i.e., a breed registry, veterinary corporations, pet insurance companies, animal welfare charities and an academic institution). This was run in program JAGS^76^ via the *R2jags* package^77^. Convergence was assessed by running two parallel chains and calculating the Gelman–Rubin statistic, which was below 1.05 for parameters, indicating convergence. Results are presented as posterior means and 95% credible intervals for all parameters. Full implementation of the model is available as Source Code 2 (10.6084/m9.figshare.24534151.v2).

Posterior estimates of dogs per postcode area were extracted from the model and transposed to allow vectorisation with human population data. Each posterior draw was divided by the human population size for that postcode area, to allow the calculation of dogs per capita mean and associated 95% credible intervals. To obtain a population estimate per region and country, population estimates per postcode area were aggregated to the regional and country level scale, respectively. Estimates for all proportional demographics, including age, parental lineage, breed, cephalic index, and body size, both within and between regions and countries, were calculated by extracting proportions from the raw data, which were then used to partition population estimates. For mapping purposes i.e., visualising geospatial data, postcode district boundaries were obtained from the University of Edinburgh’s DataShare Service^78^. Pearson correlation coefficient was used to determine the relationship between our dog population estimate per postcode area and (A) previous dog population per postcode area estimate^3^ and (B) human population per postcode area extracted from the 2011 census^27^.

## Electronic supplementary material

Below is the link to the electronic supplementary material.

Supplementary Material 1

## Data Availability

Raw datasets include commercially sensitive and potentially identifiable information. Consequently, the raw data will not be made available. However, de-duplicated and source/dog anonymized raw data are published on Figshare (https://doi.org/10.6084/m9.figshare.24534151.v2), alongside the source code for deduplication workflow (‘Source code 1’) and model simulations (‘Source code 2’).

## References

[1] UK Pet Food. *UK Pet Population, 2023.* https://www.ukpetfood.org/information-centre/statistics/uk-pet-population.html (2023).

[2] Dogs Trust. *Puppy Smuggling: Puppies Still Paying as Government Delays*. https://www.dogstrust.org.uk/downloads/2020%20Puppy%20smuggling%20report.pdf (2020).

[3] Aegerter, J., Fouracre, D. & Smith, G. C. A first estimate of the structure and density of the populations of pet cats and dogs across Great Britain. *PLOS ONE ***12**, e0174709 (2017). 28403172 10.1371/journal.pone.0174709 PMC5389805

[4] Dogs Trust. *Stray Dog Survey Report 2021–2022*. https://www.dogstrust.org.uk/downloads/Dogs-Trust%20-Stray-Dog-Survey-Report-2021-2022.pdf (2022).

[5] Morgan, L. et al. Human–dog relationships during the COVID-19 pandemic: Booming dog adoption during social isolation. *Humanit. Soc. Sci. Commun.* **7**, 1–11 (2020).

[6] Ho, J., Hussain, S. & Sparagano, O. Did the COVID-19 pandemic spark a public interest in pet adoption? *Front. Veterinary Sci.* **8**, (2021). 10.3389/fvets.2021.647308 PMC8145284 34046443

[7] Maher, J. & Wyatt, T. European illegal puppy trade and organised crime. *Trends Organ. Crim*. **24**, 506–525 (2021). 10.1007/s12117-021-09429-8 PMC8382934 34456550

[8] Yeates, J. & Bowles, D. Breeding and selling of Companion animals. In *The Palgrave International Handbook of Animal Abuse Studies* (eds Maher, J., Pierpoint, H. & Beirne, P.) 15–38 (Palgrave Macmillan UK, London). 10.1057/978-1-137-43183-7_2 (2017).

[9] IFAW (International Fund for Animal Welfare). *How Much Is That Doggie on My Browser: The Truth Behind Online Puppy Sales*. https://s3.amazonaws.com/ifaw-pantheon/sites/default/files/legacy/ifaw-report-how-much-is-that-doggie-on-my-browser.pdf (2012).

[10] Dogs Trust. *Puppy Smuggling: When Will This Cruel Trade End?* https://www.dogandcatwelfare.eu/media/publicationtemp/111018_puppy_smuggling_2018_final.pdf (2018).

[11] RSPCA. *Do puppies have secret powers? Understanding the irrational behaviour of the puppy-buying public.* https://slideplayer.com/slide/5993731/ (2012).

[12] FOUR PAWS International. *Puppy Trade in Europe. Research on the Impact of Illegal Businesses on the Market, Onconsumers, on the One-Health Concept and on Aminal Welfare.* https://www.stop-puppy-mills.org/media/REPORT-EUROPEAN-PUPPY-TRADE.pdf (2013).

[13] Norman, C., Stavisky, J. & Westgarth, C. Importing rescue dogs into the UK: Reasons, methods and welfare considerations. *Vet. Rec.* **186**, 248–248 (2020). 31932354 10.1136/vr.105380 PMC7057815

[14] Ross, K. E., Langford, F., Pearce, D. & McMillan, K. M. What patterns in online classified puppy advertisements can tell us about the current UK puppy trade. *Animals* **13**, 1682 (2023). 37238112 10.3390/ani13101682 PMC10215115

[15] Gray, A. Impending dog behaviour crisis following Covid-19 lockdown. *Vet. Rec.* **187**, e56–e56 (2020). 10.1136/vr.m3892 33024011

[16] RSPCA. *Fears recession and ‘lockdown puppies’ could spark dog welfare crisis.* https://www.rspca.org.uk/-/news-fears-recession-and-lockdown-puppies-could-spark-dog-welfare-crisis (2020).

[17] More, S. J. et al. Understanding the dog population in the Republic of Ireland: Insight from existing data sources? *Ir. Veterinary J.* **75**, 16 (2022). 10.1186/s13620-022-00223-8 PMC9281166 35836251

[18] UK Pet Food. *Historical Pet Data, 2022.* https://www.ukpetfood.org/information-centre/statistics/historical-pet-data.html (2022).

[19] ONS (Office for National Statistics). *Families and households in the UK - Office for National Statistics.* https://www.ons.gov.uk/peoplepopulationandcommunity/birthsdeathsandmarriages/families/bulletins/familiesandhouseholds/2019 (2019).

[20] Murray, J. K., Browne, W. J., Roberts, M. A., Whitmarsh, A. & Gruffydd-Jones, T. J. Number and ownership profiles of cats and dogs in the UK. *Vet. Rec.* **166**, 163–168 (2010). 20139379 10.1136/vr.b4712

[21] Murray, J. K., Gruffydd-Jones, T. J., Roberts, M. A. & Browne, W. J. Assessing changes in the UK Pet cat and dog populations: Numbers and household ownership. *Vet. Rec.* **177**, 259–259 (2015). 26350589 10.1136/vr.103223

[22] Asher, L. et al. Estimation of the number and demographics of companion dogs in the UK. *BMC Vet. Res.* **7**, 74 (2011). 22112367 10.1186/1746-6148-7-74 PMC3305510

[23] McDonald, J., Finka, L., Foreman-Worsley, R., Skillings, E. & Hodgson, D. Cat: Empirical modelling of Felis catus population dynamics in the UK. *PLOS ONE* **18**, e0287841 (2023). 37437091 10.1371/journal.pone.0287841 PMC10337951

[24] McMillan, K. M. et al. Longevity of companion dog breeds: Those at risk from early death. *Sci. Rep.* **14**, 531 (2024). 38302530 10.1038/s41598-023-50458-w PMC10834484

[25] The Kennel Club. *Breeds A to Z.* https://www.thekennelclub.org.uk/search/breeds-a-to-z/ (2021).

[26] Fédération Cynologique Internationale. *FCI: The largest canine organisation of the world.* https://www.fci.be/en/FCI-the-largest-canine-organisation-of-the-world-90.html (2021).

[27] ONS (Office for National Statistics). *Population Estimates for the United Kingdom, March 2011.* https://www.ons.gov.uk/peoplepopulationandcommunity/populationandmigration/populationestimates/bulletins/2011censuspopulationestimatesfortheunitedkingdom/2012-12-17 (2011).

[28] Harvey, N. D. How old is my dog? Identification of rational Age groupings in Pet Dogs based upon normative age-linked processes. *Front. Veterinary Sci.* **8**, (2021). 10.3389/fvets.2021.643085 PMC8110720 33987218

[29] The Kennel Club. *Breed registration statistics, 2019.* https://www.thekennelclub.org.uk/media-centre/breed-registration-statistics (2019).

[30] Pets4Homes. *Pets4Homes**Pandemic Pets: How Covid-19 affected pet sales and pricing in 2020.* https://www.pets4homes.co.uk/pet-advice/pandemic-pets-how-covid-19-affected-pet-sales-and-pricing-in-2020.html (2020).

[31] O’Neill, D. G. et al. Unravelling the health status of brachycephalic dogs in the UK using multivariable analysis. *Sci. Rep.* **10**, 17251 (2020). 33057051 10.1038/s41598-020-73088-y PMC7560694

[32] PDSA. *PAW PDSA Animal Wellbeing Report 2019*. https://www.pdsa.org.uk/media/7420/2019-paw-report_downloadable.pdf (2019).

[33] Egenvall, A., Hedhammar, A., Bonnett, B. N. & Olson, P. Gender, age, breed and distribution of morbidity and mortality in insured dogs in Sweden during 1995 and 1996. *Vet. Rec.* **146**, 519–525 (2000). 11321213 10.1136/vr.146.18.519

[34] Di Nardo, A., Candeloro, L., Budke, C. M. & Slater, M. R. Modeling the effect of sterilization rate on owned dog population size in central Italy. *Prev. Vet. Med.* **82**, 308–313 (2007). 17692414 10.1016/j.prevetmed.2007.06.007

[35] PDSA. *PAW PDSA Animal Wellbeing Report 2021*. https://www.pdsa.org.uk/media/12078/pdsa-paw-report-2021.pdf (2021).

[36] PDSA. *PAW PDSA Animal Wellbeing Report 2022.* https://www.pdsa.org.uk/what-we-do/pdsa-animal-wellbeing-report/paw-report-2022 (2022).

[37] PDSA. *PAW PDSA Animal Wellbeing Report 2023.* https://www.pdsa.org.uk/what-we-do/pdsa-animal-wellbeing-report/paw-report-2023 (2023).

[38] Michell, A. R. Longevity of British breeds of dog and its relationships with sex, size, cardiovascular variables and disease. *Vet. Rec*. **145**, 625–629 (1999). 10619607 10.1136/vr.145.22.625

[39] Proschowsky, H. F., Rugbjerg, H. & Ersbøll, A. K. Mortality of purebred and mixed-breed dogs in Denmark. *Prev. Vet. Med.* **58**, 63–74 (2003). 12628771 10.1016/s0167-5877(03)00010-2

[40] Adams, V. J., Evans, K. M., Sampson, J. & Wood, J. L. N. Methods and mortality results of a health survey of purebred dogs in the UK. *J. Small Anim. Pract.* **51**, 512–524 (2010). 21029096 10.1111/j.1748-5827.2010.00974.x

[41] O’Neill, D. G., Church, D. B., McGreevy, P. D., Thomson, P. C. & Brodbelt, D. C. Longevity and mortality of owned dogs in England. *Vet. J.* **198**, 638–643 (2013). 24206631 10.1016/j.tvjl.2013.09.020

[42] Loeb, J. *Should we legitimise puppy farming?*10.1136/vr.k4955 (2018). 10.1136/vr.k4955 30467246

[43] Teng, K. T., Brodbelt, D. C., Pegram, C., Church, D. B. & O’Neill, D. G. Life tables of annual life expectancy and mortality for companion dogs in the United Kingdom. *Sci. Rep.* **12**, 6415 (2022). 35484374 10.1038/s41598-022-10341-6 PMC9050668

[44] McMillan, K. M. et al. Longevity of companion dog breeds: those at risk from early death. *Sci. Rep. ***14**, 531 (2024). 10.1038/s41598-023-50458-w PMC10834484 38302530

[45] Herzog, H. Forty-two Thousand and one dalmatians: Fads, Social Contagion, and dog breed popularity. *Soc. Anim.* **14**, 383–397 (2006).

[46] Herzog, H. A., Bentley, R. A. & Hahn, M. W. Random drift and large shifts in popularity of dog breeds. *Proc. Royal Soc. London. Series B: Biol. Sci.*** 271**, S353–S356 (2004). 10.1098/rsbl.2004.0185 PMC1810074 15504016

[47] Packer, R. M. A., O’Neill, D. G., Fletcher, F. & Farnworth, M. J. Great expectations, inconvenient truths, and the paradoxes of the dog-owner relationship for owners of brachycephalic dogs. *PLOS ONE* **14**, e0219918 (2019). 31323057 10.1371/journal.pone.0219918 PMC6641206

[48] Packer, R. & O’Neill, D. *Health and Welfare of Brachycephalic (Flat-Faced) Companion Animals: A Complete Guide for Veterinary and Animal Professionals* (CRC, 2021).

[49] Ghirlanda, S., Acerbi, A., Herzog, H. & Serpell, J. A. Fashion vs. function in Cultural Evolution: The case of dog breed popularity. *PLOS ONE* **8**, e74770 (2013). 24040341 10.1371/journal.pone.0074770 PMC3770587

[50] O’Neill, D. G., McMillan, K. M., Church, D. B. & Brodbelt, D. C. Dog breeds and conformations in the UK in 2019: VetCompass canine demography and some consequent welfare implications. *PLOS ONE* **18**, e0288081 (2023). 37494312 10.1371/journal.pone.0288081 PMC10370710

[51] Burnett, E. et al. How much is that doodle in the window? Exploring motivations and behaviours of UK owners acquiring designer crossbreed dogs (2019–2020). *Canine Med. Genet.* **9**, 8 (2022). 35610665 10.1186/s40575-022-00120-x PMC9127489

[52] McGreevy, P. D. & Nicholas, F. W. Some Practical Solutions to Welfare problems in dog breeding. *Anim Welf.* **8**, 329–341 (1999).

[53] Trees, L. et al. Strengthening legislation around dog breeding. *Vet. Rec.* **193**, 116–117 (2023). 37539878 10.1002/vetr.3337

[54] The Kennel Club. *The Covid-19 puppy boom - one in four admit impulse buying a pandemic puppy.* https://www.thekennelclub.org.uk/media-centre/2020/august/the-covid-19-puppy-boom-one-in-four-admit-impulse-buying-a-pandemic-puppy/ (2020).

[55] Mills, G. Puppy prices soar in Covid-19 lockdown. *Vet. Rec.* **187**, 4–5 (2020). 33638547 10.1136/vr.m2755 PMC7456696

[56] Siettou, C. Societal interest in puppies and the Covid-19 pandemic: A Google trends analysis. *Prev. Vet. Med.* **196**, 105496 (2021). 34555632 10.1016/j.prevetmed.2021.105496

[57] Packer, R. M. A. et al. Pandemic puppies: Characterising motivations and behaviours of UK Owners who purchased puppies during the 2020 COVID-19 pandemic. *Animals* **11**, 2500 (2021). 34573466 10.3390/ani11092500 PMC8468924

[58] Holland, K. E. et al. More attention than Usual: A thematic analysis of Dog Ownership experiences in the UK during the First COVID-19 Lockdown. *Animals* **11**, 240 (2021). 33477947 10.3390/ani11010240 PMC7833365

[59] Pets at Home. *Pets at Home Group Plc Annual Report and Accounts 2021*. https://www.annualreports.com/HostedData/AnnualReportArchive/p/LSE_PETS.L_2021.pdf (2021).

[60] Brand, C. L. et al. Pandemic puppies: Demographic characteristics, Health and Early Life experiences of puppies Acquired during the 2020 phase of the COVID-19 pandemic in the UK. *Animals* **12**, 629 (2022). 35268198 10.3390/ani12050629 PMC8909199

[61] Christley, R. M. et al. Impact of the First COVID-19 Lockdown on Management of Pet Dogs in the UK. *Animals* **11**, 5 (2021). 10.3390/ani11010005 PMC7822167 33375158

[62] Applebaum, J. W., Tomlinson, C. A., Matijczak, A., McDonald, S. E. & Zsembik, B. A. The concerns, difficulties, and stressors of caring for pets during COVID-19: Results from a large survey of U.S. Pet Owners. *Animals* **10**, 1882 (2020). 10.3390/ani10101882 PMC7602525 33076475

[63] UK Pet Food. *NEW pet population data highlights pet peak but the number of owners giving up their pet is huge concern.* https://www.ukpetfood.org/resource/new-pfma-pet-population-data-highlights-pet-peak-but-the-number-of-owners-giving-up-their-pet-is-huge-concern.html (2022).

[64] UK Pet Food. Historical Pet Data, 2021. https://www.ukpetfood.org/information-centre/statistics/historical-pet-data.html (2021).

[65] Westgarth, C. et al. Factors associated with cat ownership in a community in the UK. *Vet. Rec.* **166**, 354–357 (2010). 20305290 10.1136/vr.c1606

[66] Murray, J. K. & Gruffydd-Jones, T. J. Proportion of pet cats registered with a veterinary practice and factors influencing registration in the UK. *Vet. J.* **192**, 461–466 (2012). 21963659 10.1016/j.tvjl.2011.08.035

[67] Downes, M. J., Clegg, T. A., Collins, D. M., McGrath, G. & More, S. J. The spatial distribution of pet dogs and pet cats on the island of Ireland. *BMC Vet. Res.* **7**, 28 (2011). 21663606 10.1186/1746-6148-7-28 PMC3141403

[68] Maher, J. & Wyatt, T. Rural-urban dynamics in the UK illegal puppy trade: Trafficking and trade in ‘man’s best friend’. *International J Rural Law Policy* **2**, 1–10 (2019).

[69] ONS (Office for National Statistics). *ONS & Postcode Directory August 2017*. https://geoportal.statistics.gov.uk/datasets/ons-postcode-directory-august-2017-1/about (2017).

[70] ONS (Office for National Statistics). *England & Wales, 2011 Census.* https://www.ons.gov.uk/census/2011census (2011).

[71] NRS (National Records of Scotland). *Scotland’s 2011 census.* https://www.scotlandscensus.gov.uk/ (2011).

[72] NISRA (Northern Ireland Statistics and Research Agency). *2011 Census.* https://www.nisra.gov.uk/statistics/census/2011-census (2011).

[73] R Core Team. *R: A Language and Environment for Statistical Computing* (R Foundation for Statistical Computing, 2021).

[74] Royle, J. A. N-Mixture models for estimating Population size from spatially replicated counts. *Biometrics* **60**, 108–115 (2004). 15032780 10.1111/j.0006-341X.2004.00142.x

[75] Fordyce, J. A., Gompert, Z., Forister, M. L. & Nice, C. C. A hierarchical bayesian approach to ecological count data: A flexible tool for ecologists. *PLOS ONE ***6**, e26785 (2011). 22132077 10.1371/journal.pone.0026785 PMC3221656

[76] Plummer, M. JAGS: A program for analysis of Bayesian graphical models using Gibbs sampling. In *Proceedings of the 3rd international workshop on distributed statistical computing.* Vol. 124, No. 125.10 (2003).

[77] Su, S. Y., Yajima, M. & Su, M. Y. S. & J.A.G.S System Requirements. Package ‘R2jags’. R package version 0.03-08 (2015).

[78] Pope, A. GB Postcode Area, Sector, District, [Dataset]. *University of Edinburgh, Tech. Rep. *10.7488/ds/1947 (2017).

[79] Madsen, L. & Royle, J. A. A review of N‐mixture models. *Wiley Interdisciplinary Reviews: Computational Statistics*, **15**(6), e1625 (2023).

