# Supplementary material for: Estimation of the size, density, and demographic distribution of the UK pet dog population in 2019
Source: Sci Rep. 2024 Dec 30;14:31746. doi: 10.1038/s41598-024-82358-y (PMC11686149; doi:10.1038/s41598-024-82358-y)
Supplement: Supplementary file 1 — Supplementary Material 1 [file 41598_2024_82358_MOESM1_ESM.pdf]

Supplementary Material:

‘Estimation of the size, density, and demographic distribution of the UK pet dog population in 2019’

Kirsten M. McMillan[1*], Xavier A. Harrison[2], David C. Wong[3], Melissa M. Upjohn[1], Robert M. Christley[1], Rachel A. Casey[1]

[1]Dogs Trust, London, UK; [2]University of Exeter, Exeter, UK; [3]University of Leeds, Leeds, UK

***For correspondence:** kirsten.mcmillan@dogstrust.org.uk

This PDF file includes: Supplementary Figures 1 to 4
Supplementary Tables 1 to 11
Supplementary Note 1

Supplementary Figure 1. Model checking against existing datasets: **(A)** Relationship between UK 2019 dog population estimate per postcode area (provided here) and previous estimate for dog population per postcode area extracted from Aegerter et al.[3], along with associated Pearson's correlation coefficient (p-value); **(B)** Relationship between UK 2019 dog population estimate per postcode area (provided here) and human population size per postcode area[27]. Dot represents mean distribution for each postcode area, and thin line represents 95% credible intervals, for both plots. An asymptotic exponential function was evident.

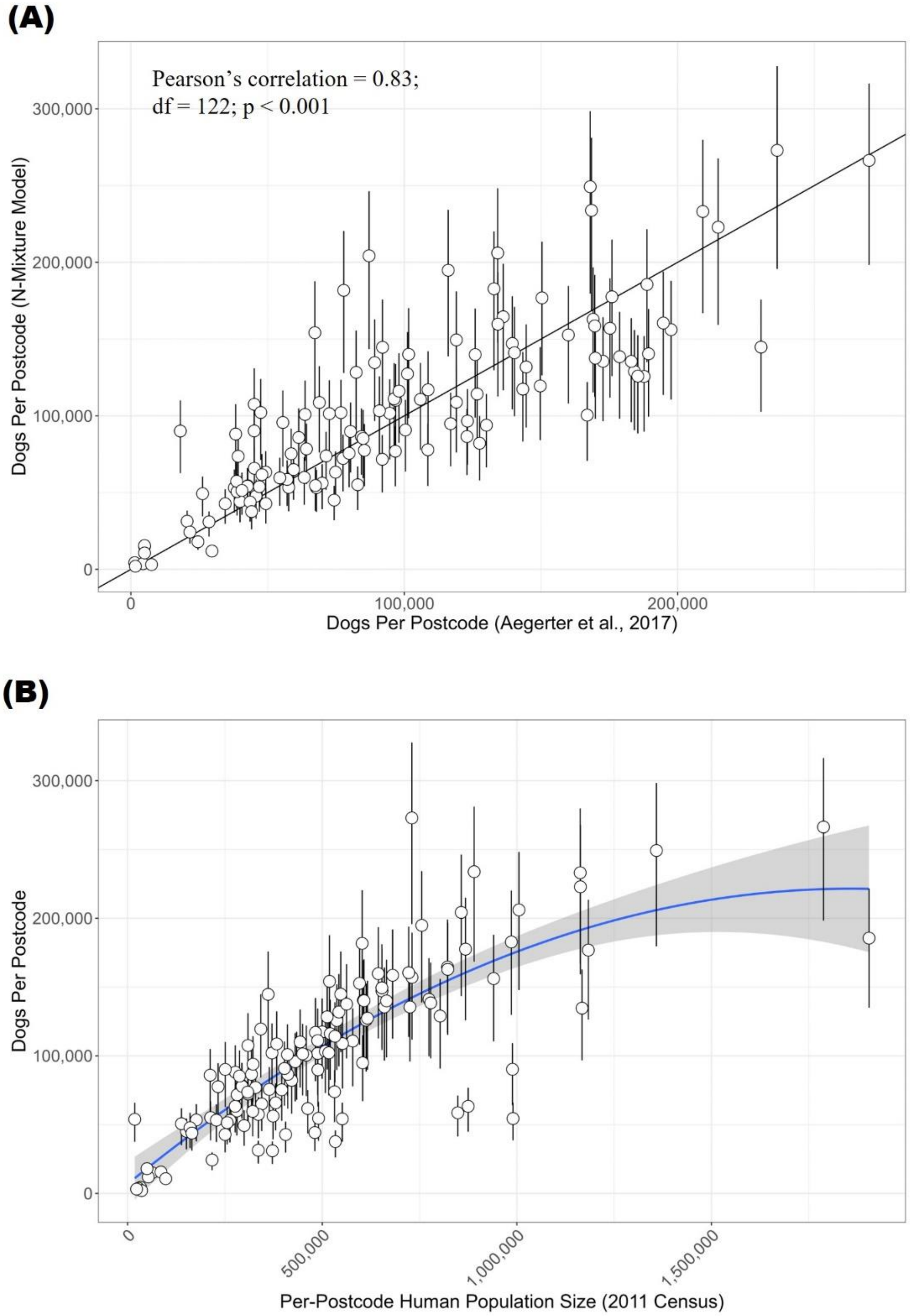

Supplementary Figure 2. Halfeye plot of marginal posterior distribution estimates regarding UK 2019 dog population per postcode area. Dot represents mean of the distribution, the bold line represents 50% of the probability density, the thinner line extends to the 90% density area.

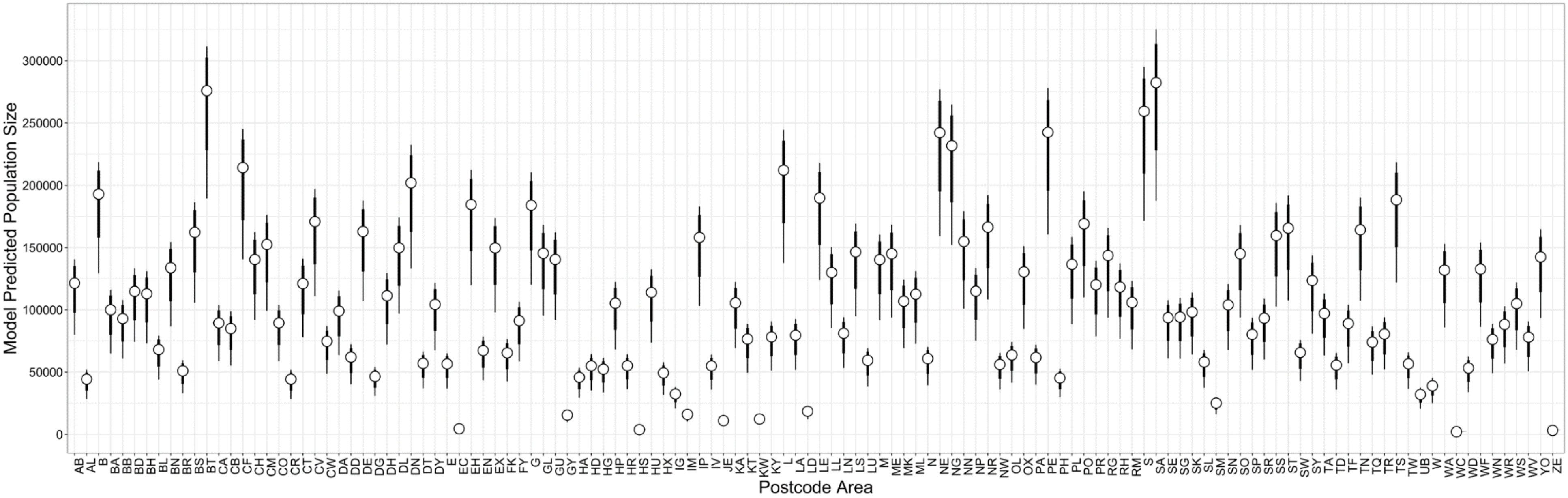

Supplementary Figure 3A. Detection probabilities per postcode as estimated by the N-mixture model. Points are posterior medians, error bars represent 66% (thick) and 95% credible intervals (thin) respectively. Grey density plots represent sampling distribution.

Detection probabilities are low for most postcodes (this figure) and surveys/data sources (Fig 3B), which is not what we would have predicted. These estimates may be depressed as a result of the deduplication process removing vast numbers of repeat-observed dogs from across different surveys, whereas N-mixture models assume that repeat observations within a site (here postcodes) are possible (see Madsen & Royle[79]). In addition, the type of N mixture model we have fitted here assumes closed populations at each site that are not subject to gains and losses, so the deduplication process we have used may violate this assumption in that each replicate survey encounters entirely new individuals at each point.

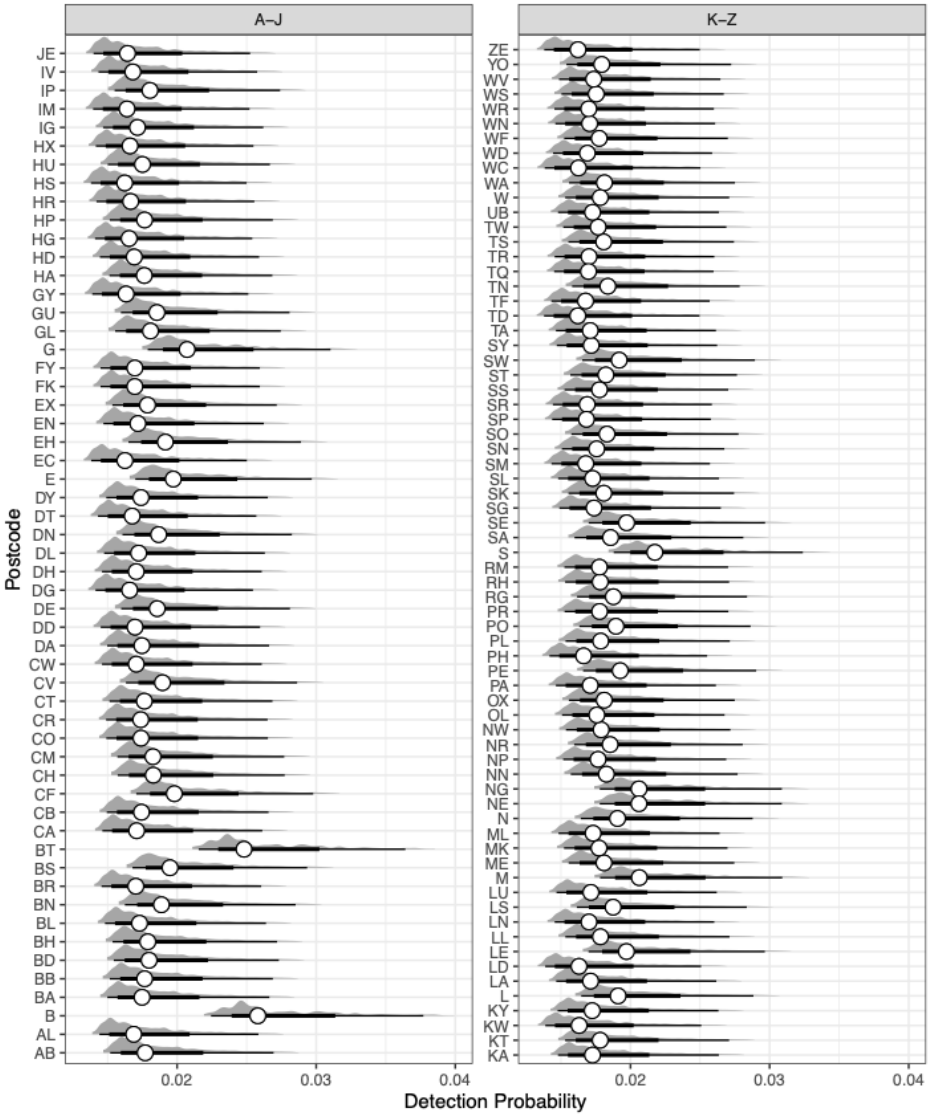

Supplementary Figure 3B. Estimated dog population postcode specific detection probabilities, for anonymized 18 raw data sources (i.e., 'Survey ID').

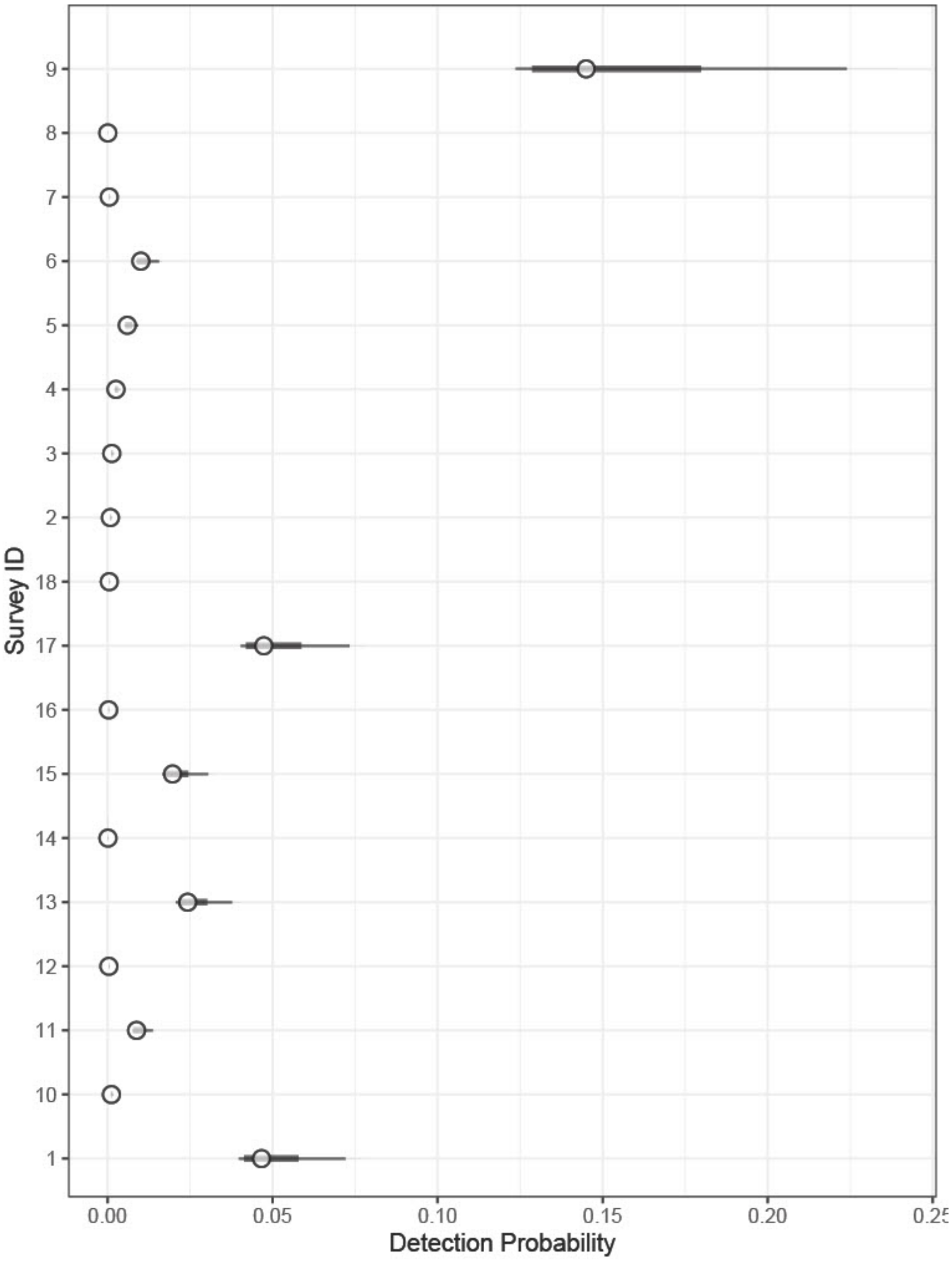

Supplementary Figure 4. Both **(A)** and **(B)** represent relationship between UK 2019 estimated dogs per capita per postcode area, and human population size per postcode area[27]. Dot represents mean of the distribution for each postcode area, and thin line represents 95% credible intervals. **(A)** Includes all UK postcode areas (n = 124), including TD postcode area, which has been identified as an outlier i.e., relatively high compared to the human population (posterior mean = 2.94). We are confident that this is due to a 'data in' issue i.e., one of the eighteen raw data sources has a strong correction factor and a low customer base for TD: leading to an overcorrection of the final output. The 95% CIs for the dog population within TD (Supplementary Table 1) incorporates a previously published estimate[3]. Despite this, we advise interpreting the TD postcode area dog population estimate with caution. **(B)** TD postcode area has been removed from all remaining UK postcode areas (n = 123).

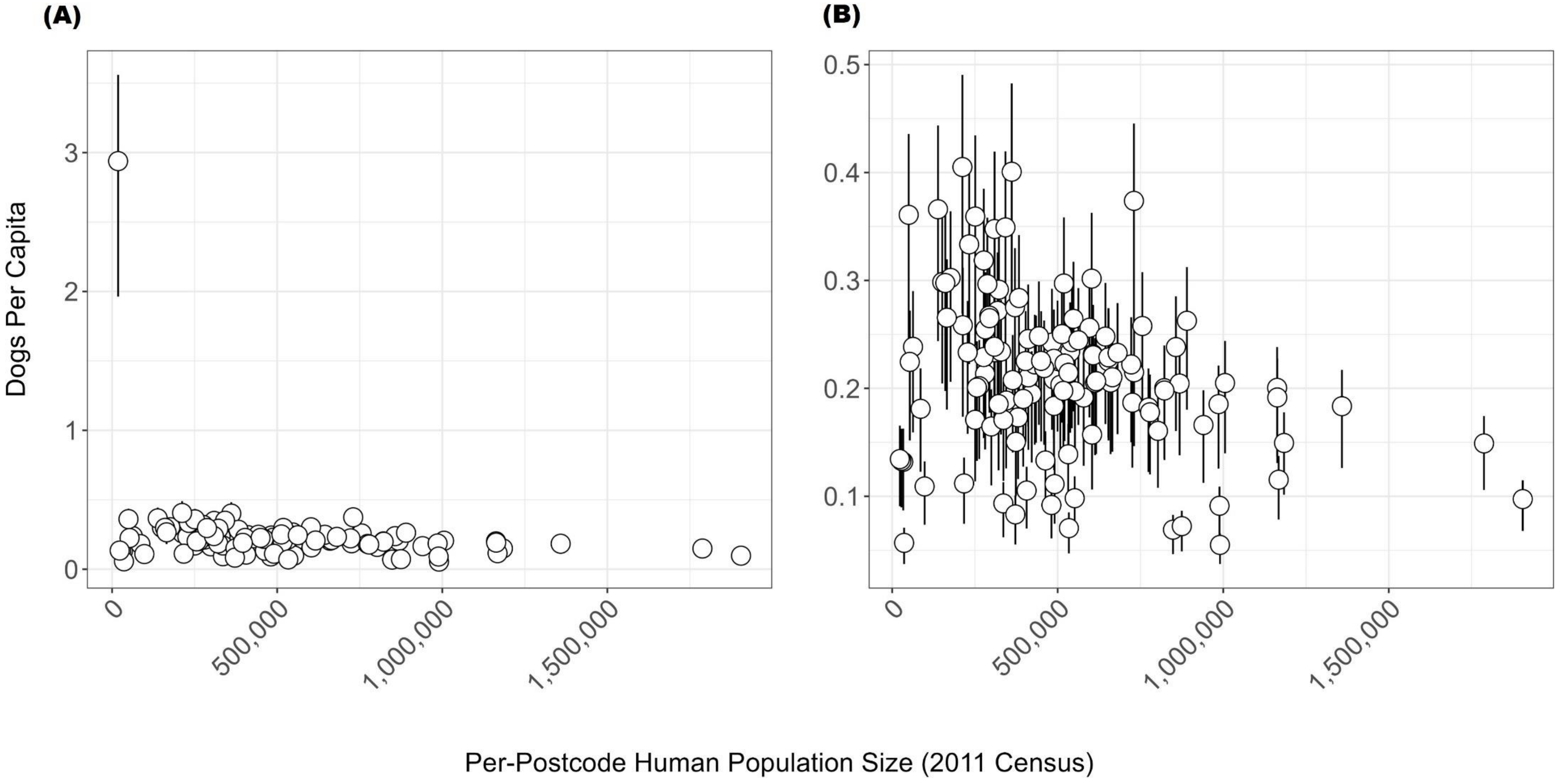

Supplementary Table 1. UK 2019 estimated dog population, standard deviation (SD) and quantiles of the marginal posterior distribution for each postcode area. The range between the 2.5% and 97.5% quantiles is the 95% credible interval for each parameter. The last two columns in the table are convergence diagnostics. Rhat i.e., potential scale reduction statistic, also known as the Gelman-Rubin statistic, is the ratio of the variance of a parameter when the data is pooled across all the chains to the within-chain variance and should ideally have a value very close to 1. It is recommended to only use the sample if R-hat < 1.05, which it is in all cases. n.eff i.e., the effective sample size, is a number smaller than or equal to the number of samples saved from the chains. The higher the autocorrelation in the saved samples, the smaller the effective sample size.

Postcode Area	Population Estimate	SD	2.5%	25%	50%	75%	97.5%	Rhat	n.eff
AB	117383.10	17558.75	79962.50	104597.75	121440.50	131910.00	140567.72	1.002	2800
AL	42802.85	6785.66	28515.85	37865.75	44327.50	48392.25	51864.02	1.003	1700
B	185557.58	25736.96	129343.57	165243.25	192890.50	208269.75	218616.17	1.002	5400
BA	96594.09	14801.67	64978.80	85737.00	100005.00	108768.25	116092.10	1.002	2400
BB	89828.55	13679.02	60774.10	79754.50	92963.00	101046.50	107929.02	1.002	2700
BD	110722.08	17047.48	74193.95	98193.25	114828.00	124800.00	133129.40	1.002	3100
BH	108880.21	16802.75	72986.05	96545.50	112892.50	122727.50	130935.15	1.002	3000
BL	65823.67	10209.31	44109.57	58373.75	68118.50	74255.25	79308.02	1.002	2200
BN	128914.87	19607.13	86662.87	114379.50	133779.00	145354.00	154437.10	1.002	5400
BR	49294.78	7719.65	32948.67	43688.50	50978.00	55650.00	59580.05	1.002	1900
BS	156122.97	23298.05	105782.37	138889.50	162266.50	175725.25	186377.42	1.002	5400
BT	266366.69	35331.36	189359.55	238608.25	275956.00	297706.00	311632.05	1.002	5400
CA	86506.00	12977.13	58988.00	77014.75	89429.50	97216.00	103736.05	1.002	2100
CB	82143.11	12590.03	55341.75	72970.00	84997.00	92541.25	98779.07	1.002	2400
CF	206076.70	30326.40	140568.52	183476.00	214197.50	231663.50	245369.62	1.002	5400
CH	135523.81	20370.01	91776.22	120596.75	140404.00	152557.75	162354.20	1.002	3600
CM	147110.69	22258.18	99160.65	130670.75	152461.50	165550.25	176276.27	1.002	3600
CO	86494.39	12995.38	58868.87	77018.00	89474.00	97087.75	103709.35	1.002	2400
CR	42765.79	6727.95	28497.25	37863.00	44319.00	48332.00	51706.32	1.002	2400
CT	116979.44	18209.68	77989.90	103764.75	121105.50	132006.75	141026.07	1.002	2600
CV	164529.65	24911.49	111015.54	146209.50	170867.00	185371.75	196975.40	1.002	5400
CW	72246.46	11024.98	48726.77	64268.75	74742.50	81265.00	86886.02	1.002	2000
DA	95675.72	14953.93	63624.42	84743.50	99061.50	108031.00	115447.20	1.002	2200
DD	59892.47	9257.59	40237.12	53127.25	62052.00	67516.00	72235.25	1.002	2000
DE	157028.55	23378.01	106991.87	139840.50	162909.50	176543.75	187680.15	1.002	4400
DG	45119.00	6705.32	30915.25	40298.00	46533.50	50619.00	54099.02	1.003	1500
DH	107508.40	16753.46	72069.07	95277.25	111336.50	121220.25	129695.22	1.002	2000
DL	144663.48	22395.20	96934.12	128427.75	149777.00	163057.25	174191.55	1.002	2100
DN	194832.71	28755.54	133062.50	173701.00	201988.00	218882.50	232502.07	1.002	4800
DT	55118.43	8523.10	37020.87	48941.75	56982.00	62130.50	66488.17	1.003	1600
DY	100889.01	15680.07	67543.22	89339.50	104368.00	113733.25	121634.10	1.002	2400
E	54401.92	8139.95	36876.85	48356.25	56590.00	61339.50	64963.02	1.002	5400
EC	4381.42	716.54	2887.97	3860.00	4524.50	4965.00	5401.00	1.003	1200
EH	177538.97	26866.43	119714.82	157643.25	184542.00	200037.00	212481.50	1.002	5400
EN	65005.95	10181.08	43344.87	57568.25	67332.50	73345.50	78547.10	1.002	2100
EX	144787.23	22006.35	97846.32	128768.25	149701.50	162876.00	173736.85	1.002	3000

FK	63296.03	9792.01	42524.90	56238.75	65449.00	71356.75	76287.07	1.002	1900
FY	88112.41	13875.60	58556.55	78041.25	91284.50	99504.25	106509.05	1.002	1800
G	176846.42	26126.03	120122.07	157377.50	183932.00	199431.00	210455.17	1.002	5400
GL	140401.08	21065.37	95349.30	125066.75	145418.00	157870.50	168089.12	1.002	3400
GU	135478.95	20403.68	91790.82	120482.00	140444.00	152498.00	162173.17	1.002	4100
GY	15004.62	2375.96	10018.00	13271.50	15502.00	16928.25	18256.12	1.003	1300
HA	44212.06	6978.36	29342.00	39119.50	45845.50	49975.25	53455.00	1.002	2600
HD	53107.47	8411.72	35354.17	46967.00	54984.50	60045.00	64331.10	1.003	1800
HG	50631.16	8031.61	33738.47	44809.75	52377.50	57213.50	61360.12	1.003	1500
HP	101700.58	15690.75	68185.80	90306.25	105330.50	114636.75	122359.05	1.002	2600
HR	53365.17	8085.55	36374.95	47447.50	55115.50	59959.50	64257.00	1.003	1600
HS	3676.04	576.32	2482.95	3263.00	3790.00	4143.00	4497.05	1.003	1400
HU	110063.96	17056.46	73664.55	97700.50	114009.00	124185.75	132562.40	1.002	2400
HX	47738.40	7624.43	31688.92	42206.75	49360.00	53976.00	57922.12	1.003	1500
IG	31356.98	4947.13	20814.90	27776.00	32460.50	35436.25	37977.07	1.002	2100
IM	15520.46	2371.17	10520.95	13827.75	16025.00	17491.25	18730.02	1.003	1400
IP	152694.01	23089.82	103170.80	135692.50	158114.50	171758.75	183002.02	1.002	3300
IV	53226.62	8156.63	35997.65	47310.75	54959.50	59921.50	64091.47	1.003	1600
JE	10680.02	1656.71	7209.77	9473.75	11014.00	12041.00	12951.05	1.003	1600
KA	102074.01	15389.50	69304.07	90859.25	105588.50	114615.25	122382.15	1.002	2100
KT	73867.20	11354.24	49583.95	65557.00	76569.00	83309.00	88839.07	1.002	3000
KW	11930.50	1837.97	8062.00	10582.75	12319.00	13426.00	14454.00	1.003	1200
KY	75579.18	11515.54	51149.82	67175.50	78203.50	85081.50	90784.22	1.002	2200
L	204244.43	30924.43	137587.07	181395.50	212132.50	230194.00	244504.07	1.002	5400
LA	76956.72	11800.44	51719.52	68365.50	79589.00	86592.75	92652.05	1.002	2100
LD	17965.07	2752.02	12187.90	15950.25	18532.00	20247.00	21697.00	1.003	1300
LE	182752.30	27270.69	123934.22	162376.00	189794.00	205868.25	217961.50	1.002	5400
LL	125664.53	18773.36	85389.97	111911.50	129957.00	141110.00	150364.12	1.002	2900
LN	78454.21	11844.75	53322.20	69834.75	81165.00	88156.75	94150.15	1.003	1800
LS	141095.74	21504.46	94905.67	125322.00	146501.00	159130.00	169127.07	1.002	5200
LU	57394.45	8933.67	38379.67	50870.00	59430.00	64779.25	69254.02	1.002	2100
M	134661.03	19886.29	91621.47	119858.25	140230.50	151706.00	160305.12	1.002	5400
ME	140045.01	21550.26	93916.77	124260.00	145059.50	157893.00	168344.75	1.002	3500
MK	103274.93	15855.90	69275.45	91706.50	106893.00	116414.25	124184.25	1.002	2800
ML	108671.19	16878.31	72736.62	96414.00	112584.00	122468.75	130996.00	1.002	2100
N	58589.87	8905.03	39400.95	51990.00	60859.00	66098.25	70215.00	1.002	5400
NE	233101.27	34098.75	159269.97	207730.75	242222.50	262338.00	277096.65	1.002	5400
NG	222858.60	32614.21	152111.12	198546.75	231776.50	250814.00	264906.67	1.002	5400
NN	149434.26	22690.42	100892.97	132863.50	154863.00	168263.00	179176.37	1.002	3600
NP	111072.72	16838.99	75165.80	98787.25	114954.50	124986.50	133329.02	1.002	2700
NR	160435.81	24192.78	108368.70	142527.75	166356.50	180679.50	192014.00	1.002	4100
NW	54164.62	8478.55	36059.45	48039.50	56103.00	61245.25	65355.20	1.002	2900
OL	61659.88	9441.20	41479.65	54722.00	63755.00	69420.00	74156.10	1.002	2600
OX	125862.69	19277.50	84649.20	111817.50	130445.50	141814.50	151162.10	1.002	3600

PA	59596.50	9326.22	39867.00	52842.75	61730.00	67289.00	71985.02	1.002	1900
PE	233828.18	34006.69	160468.82	208609.00	242553.50	262261.50	278085.37	1.002	5400
PH	43841.40	6673.77	29795.87	38932.25	45292.50	49304.25	52765.02	1.003	1500
PL	131827.50	20234.65	88494.87	116989.25	136525.00	148584.25	158512.60	1.002	2900
PO	162854.84	24642.54	109957.47	144620.50	169099.00	183559.25	195085.15	1.002	5400
PR	116099.48	17567.19	78702.50	103178.25	120166.50	130662.50	139318.12	1.002	2700
RG	138484.61	20908.02	93703.02	123069.25	143657.00	155851.50	165735.10	1.002	5100
RH	114205.64	17537.34	76785.95	101344.75	118277.50	128645.25	137308.65	1.002	3000
RM	102179.85	15882.88	68326.75	90543.00	105908.00	115209.25	123102.17	1.002	2900
S	249256.89	35744.18	171460.32	222362.50	259423.00	280706.00	295016.70	1.002	5400
SA	272934.76	39874.06	187484.97	243444.25	282410.50	306029.00	325265.07	1.002	4400
SE	90181.03	13577.95	60903.82	80068.75	93658.00	101668.50	107755.02	1.002	5400
SG	90779.96	14124.17	60700.85	80461.75	94122.00	102447.00	109451.12	1.002	2300
SK	94888.30	14406.49	64146.40	84391.25	98245.50	106866.00	113752.12	1.002	3200
SL	56193.60	8796.52	37502.42	49786.00	58097.50	63448.00	67882.10	1.002	2300
SM	24291.61	3863.10	16175.70	21462.25	25102.00	27474.25	29496.00	1.003	1700
SN	100442.85	15349.38	67789.97	89265.75	103962.00	113082.75	120685.32	1.002	2500
SO	139899.73	21406.51	93934.77	124177.25	144994.00	157691.25	167930.67	1.002	3900
SP	77537.26	12150.73	51755.92	68752.00	80260.00	87487.50	93673.02	1.002	1800
SR	90107.89	14186.64	60071.52	79796.25	93277.00	101705.00	108970.47	1.003	1800
SS	154154.54	24106.56	102742.07	136519.75	159626.50	174076.25	185918.17	1.002	2800
ST	159730.78	24396.22	107539.77	141848.00	165568.50	179989.00	191753.02	1.002	3900
SW	63278.33	9520.69	42848.97	56271.50	65741.50	71282.00	75728.15	1.002	5400
SY	119472.18	18230.90	80829.45	106270.00	123530.00	134366.00	143569.05	1.002	2000
TA	93910.04	14416.12	63302.97	83384.00	97132.00	105776.00	113054.47	1.002	2000
TD	53849.11	8483.16	35975.07	47740.00	55634.50	60763.00	65236.05	1.003	1300
TF	85902.76	13619.72	57104.80	76088.25	89024.50	97023.00	104025.37	1.003	1700
TN	158559.65	23920.56	107274.70	141075.50	164232.50	178387.75	189975.10	1.002	3800
TQ	71675.88	11189.16	47992.27	63495.50	74062.50	80845.75	86567.35	1.003	1800
TR	77900.16	12130.71	52148.77	69043.50	80565.00	87796.25	94072.20	1.002	2000
TS	181755.25	27951.57	121930.27	161214.75	188324.50	204861.50	218473.05	1.002	3400
TW	54593.72	8490.55	36583.47	48385.50	56625.00	61615.25	65841.20	1.002	2500
UB	30946.30	4890.50	20590.97	27375.25	32061.00	34991.50	37459.05	1.002	2300
W	37634.73	5862.90	25160.77	33278.75	39003.00	42493.50	45402.12	1.002	2700
WA	127351.09	19466.63	85773.77	113115.75	131915.00	143456.25	153008.15	1.002	3500
WC	2046.09	340.77	1340.00	1798.00	2112.50	2316.25	2554.02	1.003	1500
WD	51399.94	8210.58	34005.55	45462.75	53277.50	58154.00	62342.00	1.002	1900
WF	128321.00	19658.30	86227.80	113963.00	132738.50	144495.75	154142.40	1.002	2900
WN	73619.09	11454.46	49326.30	65242.50	76207.00	83019.50	88784.35	1.002	2000
WR	85242.25	13374.64	56855.40	75549.75	88253.50	96196.00	102952.25	1.002	1800
WS	101393.56	15681.59	67877.75	89947.75	104985.50	114334.75	122069.07	1.002	2400
WV	75291.68	11684.04	50471.82	66785.50	78032.00	84894.75	90757.25	1.002	2200
YO	137576.13	20659.74	93431.37	122290.00	142427.50	154575.00	164761.45	1.002	3200
ZE	3104.47	487.74	2096.95	2750.00	3198.00	3492.00	3821.02	1.003	1100

Supplementary Table 2. UK 2019 estimated dog population mean per capita, per postcode area, along with lower and upper 95% credible intervals. UK 2019 estimated dog population (i.e., marginal posterior distribution estimate) and human population size extracted from 2011 census[27] are listed for reference. N.B. The estimate for the TD dog population is an outlier i.e., relatively high compared to the human population (posterior mean = 2.94). We advise interpreting the TD postcode area estimate with caution.

Postcode Area	Population Mean, Per Capita	Lower 95% CI	Upper 95% CI	Population Estimate	Human Population Estimate
AB	0.23	0.16	0.28	117383.10	499692.00
AL	0.17	0.11	0.21	42802.85	250427.00
B	0.10	0.07	0.11	185557.58	1904658.00
BA	0.22	0.15	0.27	96594.09	434166.00
BB	0.18	0.12	0.22	89828.55	488917.00
BD	0.19	0.13	0.23	110722.08	578336.00
BH	0.20	0.13	0.24	108880.21	551987.00
BL	0.17	0.12	0.21	65823.67	380259.00
BN	0.16	0.11	0.19	128914.87	802831.00
BR	0.16	0.11	0.20	49294.78	299293.00
BS	0.17	0.11	0.20	156122.97	940241.00
BT	0.15	0.11	0.17	266366.69	1787720.00
CA	0.27	0.19	0.33	86506.00	318244.00
CB	0.19	0.13	0.23	82143.11	421467.00
CF	0.20	0.14	0.24	206076.70	1005334.00
CH	0.21	0.14	0.25	135523.81	659743.00
CM	0.23	0.15	0.27	147110.69	653492.00
CO	0.21	0.14	0.25	86494.39	411418.00
CR	0.11	0.07	0.13	42765.79	405982.00
CT	0.24	0.16	0.29	116979.44	482504.00
CV	0.20	0.14	0.24	164529.65	821807.00
CW	0.23	0.16	0.28	72246.46	309489.00
DA	0.22	0.15	0.27	95675.72	430560.00
DD	0.21	0.14	0.26	59892.47	280568.00
DE	0.21	0.15	0.26	157028.55	730620.00
DG	0.30	0.20	0.36	45119.00	151139.00
DH	0.35	0.23	0.42	107508.40	309211.00
DL	0.40	0.27	0.48	144663.48	360975.00
DN	0.26	0.18	0.31	194832.71	755713.00
DT	0.26	0.17	0.31	55118.43	213203.00
DY	0.25	0.16	0.30	100889.01	410598.00
E	0.05	0.04	0.07	54401.92	990035.00
EC	0.13	0.09	0.16	4381.42	33205.00
EH	0.20	0.14	0.24	177538.97	868113.00
EN	0.19	0.13	0.23	65005.95	344434.00
EX	0.26	0.18	0.32	144787.23	547511.00
FK	0.23	0.15	0.28	63296.03	276371.00

FY	0.32	0.21	0.39	88112.41	276623.00
G	0.15	0.10	0.18	176846.42	1183614.00
GL	0.23	0.16	0.28	140401.08	605821.00
GU	0.19	0.13	0.22	135478.95	725368.00
GY	0.24	0.16	0.29	15004.62	62915.00
HA	0.09	0.06	0.11	44212.06	480953.00
HD	0.20	0.13	0.24	53107.47	262814.00
HG	0.37	0.24	0.44	50631.16	138343.00
HP	0.21	0.14	0.25	101700.58	488351.00
HR	0.30	0.21	0.36	53365.17	176493.00
HS	0.13	0.09	0.16	3676.04	27663.00
HU	0.25	0.17	0.30	110063.96	443223.00
HX	0.30	0.20	0.36	47738.40	160378.00
IG	0.09	0.06	0.11	31356.98	335694.00
IM	0.18	0.12	0.22	15520.46	85716.00
IP	0.26	0.17	0.31	152694.01	595934.00
IV	0.23	0.16	0.28	53226.62	228056.00
JE	0.11	0.07	0.13	10680.02	97857.00
KA	0.28	0.19	0.33	102074.01	370877.00
KT	0.14	0.09	0.17	73867.20	531664.00
KW	0.22	0.15	0.27	11930.50	53132.00
KY	0.21	0.14	0.25	75579.18	363956.00
L	0.24	0.16	0.29	204244.43	857079.00
LA	0.23	0.16	0.28	76956.72	328704.00
LD	0.36	0.24	0.44	17965.07	49792.00
LE	0.19	0.13	0.22	182752.30	985795.00
LL	0.23	0.16	0.28	125664.53	537467.00
LN	0.27	0.18	0.32	78454.21	293310.00
LS	0.18	0.12	0.22	141095.74	774180.00
LU	0.17	0.11	0.21	57394.45	335950.00
M	0.12	0.08	0.14	134661.03	1167402.00
ME	0.23	0.15	0.28	140045.01	607143.00
MK	0.20	0.14	0.24	103274.93	507978.00
ML	0.28	0.19	0.34	108671.19	382997.00
N	0.07	0.05	0.08	58589.87	848197.00
NE	0.20	0.14	0.24	233101.27	1162698.00
NG	0.19	0.13	0.23	222858.60	1163185.00
NN	0.23	0.15	0.27	149434.26	653215.00
NP	0.23	0.15	0.27	111072.72	488368.00
NR	0.22	0.15	0.27	160435.81	722087.00
NW	0.10	0.07	0.12	54164.62	551407.00
OL	0.13	0.09	0.16	61659.88	462833.00
OX	0.21	0.14	0.25	125862.69	612827.00
PA	0.19	0.12	0.22	59596.50	321350.00

PE	0.26	0.18	0.31	233828.18	890223.00
PH	0.27	0.18	0.32	43841.40	165118.00
PL	0.24	0.16	0.29	131827.50	542719.00
PO	0.20	0.13	0.24	162854.84	822331.00
PR	0.22	0.15	0.27	116099.48	520556.00
RG	0.18	0.12	0.21	138484.61	778677.00
RH	0.21	0.14	0.26	114205.64	532536.00
RM	0.20	0.13	0.24	102179.85	516824.00
S	0.18	0.13	0.22	249256.89	1358507.00
SA	0.37	0.26	0.45	272934.76	730232.00
SE	0.09	0.06	0.11	90181.03	988702.00
SG	0.23	0.15	0.27	90779.96	402911.00
SK	0.16	0.11	0.19	94888.30	603795.00
SL	0.15	0.10	0.18	56193.60	373607.00
SM	0.11	0.07	0.14	24291.61	217048.00
SN	0.22	0.15	0.26	100442.85	459049.00
SO	0.21	0.14	0.25	139899.73	665193.00
SP	0.33	0.22	0.40	77537.26	232524.00
SR	0.36	0.24	0.43	90107.89	250826.00
SS	0.30	0.20	0.36	154154.54	518677.00
ST	0.25	0.17	0.30	159730.78	644068.00
SW	0.07	0.05	0.09	63278.33	874844.00
SY	0.35	0.24	0.42	119472.18	342140.00
TA	0.29	0.20	0.35	93910.04	322197.00
TD	2.94	1.96	3.56	53849.11	18324.00
TF	0.41	0.27	0.49	85902.76	212061.00
TN	0.23	0.16	0.28	158559.65	680816.00
TQ	0.25	0.17	0.31	71675.88	281404.00
TR	0.27	0.18	0.32	77900.16	293864.00
TS	0.30	0.20	0.36	181755.25	602474.00
TW	0.11	0.07	0.13	54593.72	490472.00
UB	0.08	0.06	0.10	30946.30	371969.00
W	0.07	0.05	0.09	37634.73	533706.00
WA	0.21	0.14	0.25	127351.09	616180.00
WC	0.06	0.04	0.07	2046.09	35995.00
WD	0.20	0.13	0.24	51399.94	255988.00
WF	0.25	0.17	0.30	128321.00	512657.00
WN	0.24	0.16	0.29	73619.09	308483.00
WR	0.30	0.20	0.36	85242.25	287414.00
WS	0.23	0.15	0.27	101393.56	449687.00
WV	0.19	0.13	0.23	75291.68	395857.00
YO	0.24	0.17	0.29	137576.13	562439.00
ZE	0.13	0.09	0.17	3104.47	23086.00

Supplementary Table 3. UK 2019 dog population regional estimates for proportional age demographics (%). Age group (AG) population estimate, per region, and associated proportional age group demographics both within (($N_{\text{AG, region}}/N_{\text{region}}$)*100) and between regions (($N_{\text{AG, region}}/N_{\text{total AG}}$)*100). Example: 15.2% of Central Scotland's population is within the geriatric development period ('within region') and 1.3% of the UK geriatric population can be found within Central Scotland ('between regions').

Region	Age Group	Population Estimate	Proportion Age Group WITHIN Region (%)	Proportion Age Group BETWEEN Regions (%)
Central Scotland	Geriatric	26158.13	15.2	1.3
Central Scotland	Senior Adults	55444.04	32.2	1.3
Central Scotland	Mature Adults	68623.34	39.9	1.4
Central Scotland	Young Adults	15152.05	8.8	1.4
Central Scotland	Juveniles	4941.44	2.9	1.5
Central Scotland	Puppies	1648.21	1.0	1.4
Channel Islands	Geriatric	3772.58	14.7	0.2
Channel Islands	Senior Adults	8511.97	33.1	0.2
Channel Islands	Mature Adults	10411.78	40.5	0.2
Channel Islands	Young Adults	2011.37	7.8	0.2
Channel Islands	Juveniles	703.13	2.7	0.2
Channel Islands	Puppies	273.82	1.1	0.2
East England	Geriatric	201460.72	15.4	10.1
East England	Senior Adults	438181.69	33.5	10.5
East England	Mature Adults	509933.37	38.9	10.4
East England	Young Adults	113709.43	8.7	10.6
East England	Juveniles	33735.60	2.6	9.9
East England	Puppies	12517.75	1.0	10.7
East Midlands	Geriatric	111381.30	15.6	5.6
East Midlands	Senior Adults	238447.35	33.5	5.7
East Midlands	Mature Adults	276289.26	38.8	5.6
East Midlands	Young Adults	59079.23	8.3	5.5
East Midlands	Juveniles	20071.45	2.8	5.9
East Midlands	Puppies	6805.11	1.0	5.8
Glasgow	Geriatric	28763.47	16.3	1.4
Glasgow	Senior Adults	56747.39	32.1	1.4
Glasgow	Mature Adults	70258.92	39.7	1.4
Glasgow	Young Adults	14329.94	8.1	1.3
Glasgow	Juveniles	4824.99	2.7	1.4
Glasgow	Puppies	1921.71	1.1	1.6
Highlands and Islands	Geriatric	9192.79	12.8	0.5
Highlands and Islands	Senior Adults	22824.53	31.7	0.5
Highlands and Islands	Mature Adults	30335.55	42.2	0.6
Highlands and Islands	Young Adults	7145.86	9.9	0.7
Highlands and Islands	Juveniles	1909.58	2.7	0.6

Highlands and Islands	Puppies	529.32	0.7	0.5
Isle of Man	Geriatric	2103.88	13.6	0.1
Isle of Man	Senior Adults	5249.36	33.8	0.1
Isle of Man	Mature Adults	6315.10	40.7	0.1
Isle of Man	Young Adults	1372.70	8.8	0.1
Isle of Man	Juveniles	410.43	2.6	0.1
Isle of Man	Puppies	68.98	0.4	0.1
London	Geriatric	159318.41	17.0	8.0
London	Senior Adults	307197.24	32.9	7.3
London	Mature Adults	359294.61	38.4	7.3
London	Young Adults	75465.99	8.1	7.0
London	Juveniles	23637.21	2.5	7.0
London	Puppies	9678.72	1.0	8.3
Lothian	Geriatric	29235.48	16.5	1.5
Lothian	Senior Adults	57966.76	32.7	1.4
Lothian	Mature Adults	69826.78	39.3	1.4
Lothian	Young Adults	14333.43	8.1	1.3
Lothian	Juveniles	4372.58	2.5	1.3
Lothian	Puppies	1803.93	1.0	1.5
Mid Scotland and Fife	Geriatric	14864.90	12.4	0.7
Mid Scotland and Fife	Senior Adults	39082.62	32.7	0.9
Mid Scotland and Fife	Mature Adults	50018.28	41.9	1.0
Mid Scotland and Fife	Young Adults	11132.29	9.3	1.0
Mid Scotland and Fife	Juveniles	3280.37	2.7	1.0
Mid Scotland and Fife	Puppies	1042.12	0.9	0.9
Mid Wales	Geriatric	2935.70	16.3	0.1
Mid Wales	Senior Adults	6191.02	34.5	0.1
Mid Wales	Mature Adults	6823.32	38.0	0.1
Mid Wales	Young Adults	1570.34	8.7	0.1
Mid Wales	Juveniles	375.21	2.1	0.1
Mid Wales	Puppies	69.48	0.4	0.1
North East England	Geriatric	123052.41	16.3	6.2
North East England	Senior Adults	254831.51	33.7	6.1
North East England	Mature Adults	290272.05	38.3	5.9
North East England	Young Adults	61479.02	8.1	5.7
North East England	Juveniles	20865.59	2.8	6.1
North East England	Puppies	6635.70	0.9	5.7
North East Scotland	Geriatric	25272.74	14.3	1.3
North East Scotland	Senior Adults	59080.18	33.3	1.4
North East Scotland	Mature Adults	70596.75	39.8	1.4
North East Scotland	Young Adults	15973.47	9.0	1.5
North East Scotland	Juveniles	4960.25	2.8	1.5
North East Scotland	Puppies	1392.18	0.8	1.2
North Wales	Geriatric	17017.66	13.5	0.9

North Wales	Senior Adults	42322.37	33.7	1.0
North Wales	Mature Adults	50550.07	40.2	1.0
North Wales	Young Adults	11407.58	9.1	1.1
North Wales	Juveniles	3579.71	2.8	1.1
North Wales	Puppies	787.16	0.6	0.7
North West England	Geriatric	214138.94	14.5	10.7
North West England	Senior Adults	472747.83	31.9	11.3
North West England	Mature Adults	596111.86	40.3	12.1
North West England	Young Adults	137862.87	9.3	12.8
North West England	Juveniles	44841.78	3.0	13.2
North West England	Puppies	14925.09	1.0	12.8
Northern Ireland	Geriatric	45087.37	16.9	2.3
Northern Ireland	Senior Adults	103501.47	38.9	2.5
Northern Ireland	Mature Adults	88025.55	33.0	1.8
Northern Ireland	Young Adults	20910.21	7.9	1.9
Northern Ireland	Juveniles	6908.73	2.6	2.0
Northern Ireland	Puppies	1933.35	0.7	1.7
South East England	Geriatric	254478.93	15.7	12.7
South East England	Senior Adults	539540.67	33.4	12.9
South East England	Mature Adults	632825.78	39.1	12.9
South East England	Young Adults	133129.67	8.2	12.4
South East England	Juveniles	40731.58	2.5	12.0
South East England	Puppies	15723.04	1.0	13.5
South Scotland	Geriatric	27766.39	13.8	1.4
South Scotland	Senior Adults	65667.23	32.7	1.6
South Scotland	Mature Adults	82509.08	41.0	1.7
South Scotland	Young Adults	18412.03	9.2	1.7
South Scotland	Juveniles	5180.57	2.6	1.5
South Scotland	Puppies	1506.83	0.7	1.3
South Wales	Geriatric	47599.41	15.0	2.4
South Wales	Senior Adults	103071.27	32.5	2.5
South Wales	Mature Adults	125924.61	39.7	2.6
South Wales	Young Adults	29441.96	9.3	2.7
South Wales	Juveniles	8458.35	2.7	2.5
South Wales	Puppies	2653.83	0.8	2.3
South West England	Geriatric	188271.96	15.0	9.4
South West England	Senior Adults	405558.55	32.3	9.7
South West England	Mature Adults	501097.57	39.9	10.2
South West England	Young Adults	112772.41	9.0	10.5
South West England	Juveniles	35595.99	2.8	10.5
South West England	Puppies	11901.22	0.9	10.2
West Midlands	Geriatric	198412.01	17.5	9.9
West Midlands	Senior Adults	379577.26	33.6	9.1
West Midlands	Mature Adults	421427.94	37.2	8.6

West Midlands	Young Adults	92136.09	8.1	8.6
West Midlands	Juveniles	29703.06	2.6	8.7
West Midlands	Puppies	10118.25	0.9	8.7
West Scotland	Geriatric	7001.64	11.7	0.4
West Scotland	Senior Adults	19626.18	32.9	0.5
West Scotland	Mature Adults	25496.88	42.8	0.5
West Scotland	Young Adults	5238.52	8.8	0.5
West Scotland	Juveniles	1655.11	2.8	0.5
West Scotland	Puppies	578.18	1.0	0.5
West Wales	Geriatric	52480.04	19.2	2.6
West Wales	Senior Adults	98466.36	36.1	2.3
West Wales	Mature Adults	94088.00	34.5	1.9
West Wales	Young Adults	20728.65	7.6	1.9
West Wales	Juveniles	5671.07	2.1	1.7
West Wales	Puppies	1500.63	0.5	1.3
Yorkshire and The Humber	Geriatric	208413.19	16.7	10.4
Yorkshire and The Humber	Senior Adults	413238.04	33.1	9.9
Yorkshire and The Humber	Mature Adults	480384.14	38.5	9.8
Yorkshire and The Humber	Young Adults	102716.57	8.2	9.5
Yorkshire and The Humber	Juveniles	33134.38	2.7	9.8
Yorkshire and The Humber	Puppies	10805.96	0.9	9.3

Supplementary Table 4. UK 2019 dog population estimate, per purebred, and associated proportional breed demographics (%), ranked by popularity. Purebreds of an equal estimated population size are secondary ranked alphabetically.

Rank	Purebreds	Population Estimate	Proportion of UK Population (%)
1	Retriever Labrador	1107026.89	10.2
2	Spaniel Cocker	751084.86	6.9
3	Staffordshire Bull Terrier	509953.75	4.7
4	Spaniel English Springer	470051.42	4.3
5	German Shepherd Dog	384517.08	3.5
6	French Bulldog	368361.24	3.4
7	Retriever Golden	297247.98	2.7
8	Pug	289760.06	2.7
9	Border Terrier	275497.48	2.5
10	Shih Tzu	263181.01	2.4
11	Cavalier King Charles Spaniel	262664.4	2.4
12	Jack Russell Terrier	262464.8	2.4
13	Bulldog	262441.32	2.4
14	West Highland White Terrier	228089.62	2.1
15	Boxer	227065.2	2.1
16	Yorkshire Terrier	225022.24	2.1
17	Miniature Schnauzer	223583.95	2.1
18	Border Collie	219618.37	2
19	Chihuahua Smooth Coat	181019.31	1.7
20	Lhasa Apso	176449.07	1.6
21	Poodle	162074.96	1.5
22	Dachshund Miniature Smooth Haired	154161.41	1.4
23	Whippet	139710.98	1.3
24	Beagle	126528.6	1.2
25	Bichon Frise	126011.99	1.2
26	Rottweiler	119525	1.1
27	Siberian Husky	104229.2	1
28	Chihuahua Long Coat	103788.91	1
29	Hungarian Vizsla	99943.68	0.9
30	Bull Terrier	96926.2	0.9
31	Dogue De Bordeaux	91575.17	0.8
32	Shar Pei	91331.54	0.8
33	Weimaraner	74929.14	0.7
34	Dobermann	74926.2	0.7
35	Pomeranian	70793.32	0.7
36	Dalmatian	70138.75	0.6
37	German Shorthaired Pointer	66155.56	0.6
38	Boston Terrier	61385.71	0.6
39	Cairn Terrier	57939.68	0.5
40	Maltese	55330.21	0.5

41	Retriever Flat Coated	54164.9	0.5
42	Tibetan Terrier	53304.86	0.5
43	Rhodesian Ridgeback	50525.14	0.5
44	American Akita	48285.52	0.4
45	Alaskan Malamute	47563.43	0.4
46	Greyhound	47269.91	0.4
47	Shetland Sheepdog	47043.89	0.4
48	Great Dane	46809.06	0.4
49	Basset Hound	45382.51	0.4
50	Bullmastiff	42855.23	0.4
51	Dachshund Miniature Long Haired	42303.39	0.4
52	Newfoundland	38249.76	0.4
53	Scottish Terrier	36661.77	0.3
54	Collie Rough	36274.31	0.3
55	Irish Setter	34345.82	0.3
56	Fox Terrier Wire	30981.98	0.3
57	Pointer	29851.9	0.3
58	Airedale Terrier	29828.41	0.3
59	Bedlington Terrier	29781.45	0.3
60	Parson Russell Terrier	28909.67	0.3
61	Dachshund Smooth Haired	28883.25	0.3
62	Dachshund Miniature Wire Haired	28354.9	0.3
63	Papillon	27756.1	0.3
64	King Charles Spaniel	27286.45	0.3
65	Norfolk Terrier	26262.04	0.2
66	St Bernard	25930.35	0.2
67	Bernese Mountain Dog	25105.53	0.2
68	Bearded Collie	24861.9	0.2
69	Chow Chow	24794.39	0.2
70	Old English Sheepdog	24456.83	0.2
71	Chinese Crested	21316.07	0.2
72	Lakeland Terrier	20652.7	0.2
73	Mastiff	20558.77	0.2
74	Italian Spinone	19613.61	0.2
75	Dachshund Wire Haired	18342.63	0.2
76	Miniature Pinscher	17881.78	0.2
77	Welsh Terrier	17602.93	0.2
78	Soft Coated Wheaten Terrier	17397.46	0.2
79	Schnauzer	17018.81	0.2
80	Welsh Corgi Pembroke	16951.3	0.2
81	German Wirehaired Pointer	16469.91	0.2
82	Pekingese	16373.05	0.2
83	Spaniel Welsh Springer	16097.13	0.1
84	Samoyed	16058.97	0.1

85	Japanese Akita Inu	15759.57	0.1
86	Irish Terrier	15554.1	0.1
87	Leonberger	15198.93	0.1
88	Spaniel American Cocker	14934.76	0.1
89	English Setter	14658.84	0.1
90	Havanese	12695.13	0.1
91	Japanese Shiba Inu	12436.82	0.1
92	Belgian Shepherd Dog Malinois	12025.88	0.1
93	Brittany	11782.25	0.1
94	Italian Greyhound	11664.84	0.1
95	Irish Wolfhound	11397.73	0.1
96	Gordon Setter	11218.68	0.1
97	Coton De Tulear	11089.52	0.1
98	Saluki	10825.35	0.1
99	Deerhound	10026.95	0.1
100	Giant Schnauzer	9877.25	0.1
101	Spaniel Clumber	9756.9	0.1
102	Japanese Chin	9389.99	0.1
103	Dachshund Long Haired	9290.19	0.1
104	Collie Smooth	9184.52	0.1
105	Retriever Nova Scotia Duck Tolling	9017.21	0.1
106	Tibetan Spaniel	8905.67	0.1
107	Japanese Spitz	8582.79	0.1
108	Kerry Blue Terrier	8576.92	0.1
109	Griffon Bruxellois	8298.06	0.1
110	Spanish Water Dog	8039.76	0.1
111	Manchester Terrier	8028.02	0.1
112	Bull Terrier Miniature	8019.21	0.1
113	Australian Shepherd	7141.56	0.1
114	Neapolitan Mastiff	6686.59	0.1
115	Portuguese Water Dog	6572.11	0.1
116	Afghan Hound	6463.51	0.1
117	Bolognese	6369.58	0.1
118	Lancashire Heeler	6234.56	0.1
119	Norwich Terrier	6219.88	0.1
120	Affenpinscher	5735.56	0.1
121	Fox Terrier Smooth	5644.56	0.1
122	Pyrenean Mountain Dog	5409.74	<0.1
123	Welsh Corgi Cardigan	5268.84	<0.1
124	Briard	5251.23	<0.1
125	Dandie Dinmont Terrier	5183.72	<0.1
126	German Spitz Mittel	5075.12	<0.1
127	English Toy Terrier	4972.38	<0.1
128	Basset Griffon Vendeen Petit	4916.61	<0.1

129	Keeshond	4693.53	<0.1
130	Spaniel Field	4532.09	<0.1
131	Spaniel Irish Water	4526.22	<0.1
132	Eurasier	4432.29	<0.1
133	Large Münsterländer	4303.13	<0.1
134	Löwchen	4270.85	<0.1
135	Basset Fauve De Bretagne	4156.37	<0.1
136	Basset Griffon Vendeen Grand	4077.12	<0.1
137	Sealyham Terrier	4018.41	<0.1
138	German Spitz Klein	3804.14	<0.1
139	Italian Cane Corso	3801.2	<0.1
140	Borzoi	3795.33	<0.1
141	Irish Red And White Setter	3777.72	<0.1
142	Bracco Italiano	3692.59	<0.1
143	Retriever Chesapeake Bay	3522.35	<0.1
144	Retriever Curly Coated	3490.06	<0.1
145	Belgian Shepherd Dog Tervueren	3440.16	<0.1
146	Finnish Lapphund	3404.94	<0.1
147	Norwegian Elkhound	3363.84	<0.1
148	Russian Black Terrier	3334.49	<0.1
149	Tibetan Mastiff	3181.85	<0.1
150	Australian Cattle Dog	3017.48	<0.1
151	Bouvier Des Flandres	2996.93	<0.1
152	German Pinscher	2903	<0.1
153	Presa Canario	2891.26	<0.1
154	Hungarian Puli	2770.91	<0.1
155	Spaniel Sussex	2703.4	<0.1
156	Bloodhound	2627.08	<0.1
157	Portuguese Podengo	2609.47	<0.1
158	Glen Of Imaal Terrier	2603.6	<0.1
159	Lagotto Romagnolo	2574.25	<0.1
160	Australian Silky Terrier	2468.58	<0.1
161	Belgian Shepherd Dog Groenendael	2456.84	<0.1
162	American Staffordshire Terrier	2430.42	<0.1
163	Polish Lowland Sheepdog	2386.39	<0.1
164	Bavarian Mountain Hound	2342.36	<0.1
165	Basenji	2125.15	<0.1
166	Australian Kelpie	2098.73	<0.1
167	Skye Terrier	2040.03	<0.1
168	Korthals Griffon	1990.13	<0.1
169	Schipperke	1910.87	<0.1
170	Slovakian Rough Haired Pointer	1828.69	<0.1
171	Swedish Vallhund	1802.27	<0.1
172	Catalan Sheepdog	1731.82	<0.1

173	Foxhound	1620.28	<0.1
174	Russian Toy	1364.91	<0.1
175	Maremma Sheepdog	1285.66	<0.1
176	Anatolian Shepherd Dog	1282.72	<0.1
177	Cesky Terrier	1276.85	<0.1
178	Great Swiss Mountain Dog	1241.63	<0.1
179	Hovawart	1232.82	<0.1
180	Caucasian Shepherd Dog	1229.89	<0.1
181	Otterhound	1200.53	<0.1
182	Kooikerhondje	1197.6	<0.1
183	German Longhaired Pointer	1171.18	<0.1
184	Norwegian Buhund	1165.31	<0.1
185	Xoloitzcuintle	1156.5	<0.1
186	Pharaoh Hound	1071.38	<0.1
187	Beauceron	1006.8	<0.1
188	Finnish Spitz	968.65	<0.1
189	Estrela Mountain Dog	927.55	<0.1
190	Harrier	851.23	<0.1
191	Ibizan Hound	842.43	<0.1
192	Canadian Eskimo Dog	833.62	<0.1
193	Canaan Dog	757.3	<0.1
194	Dutch Shepherd Dog	645.76	<0.1
195	Cirneco dell’Etna	543.03	<0.1
196	Spanish Mastiff	537.16	<0.1
197	Pyrenean Sheepdog Long Haired	528.35	<0.1
198	Hamiltonstovare	525.42	<0.1
199	Canarian Warren Hound	519.55	<0.1
200	Hungarian Pumi	487.26	<0.1
201	Entlebucher Mountain Dog	484.32	<0.1
202	Portuguese Pointer	469.65	<0.1
203	Komondor	425.62	<0.1
204	Korean Jindo	425.62	<0.1
205	Spanish Greyhound	393.33	<0.1
206	Basset Bleu De Gascogne	349.3	<0.1
207	Belgian Shepherd Dog Laekenois	331.69	<0.1
208	Sloughi	322.88	<0.1
209	Czechoslovakian Wolfdog	305.27	<0.1
210	White Swiss Shepherd Dog	296.46	<0.1
211	Romanian Carpathian Shepherd Dog	293.53	<0.1
212	Bergamasco	278.85	<0.1
213	Central Asia Shepherd Dog	278.85	<0.1
214	Auvergne Pointer	275.92	<0.1
215	Small Münsterländer	226.02	<0.1
216	Pyrenean Sheepdog Smooth Faced	223.08	<0.1

217	Greenland Dog	199.6	<0.1
218	Hellenic Hound	196.66	<0.1
219	Stabijhoun	190.79	<0.1
220	Thai Ridgeback Dog	176.12	<0.1
221	Turkish Kangal Dog	173.18	<0.1
222	Barbet	167.31	<0.1
223	Dutch Schapendoes	161.44	<0.1
224	German Hunting Terrier	149.7	<0.1
225	Picardy Sheepdog	149.7	<0.1
226	Romanian Bucovina Shepherd	137.96	<0.1
227	Spanish Hound	137.96	<0.1
228	Black And Tan Coonhound	105.67	<0.1
229	Griffon Fauve De Bretagne	102.74	<0.1
230	Swiss Hound	99.8	<0.1
231	Yugoslavian Shepherd Dog	99.8	<0.1
232	Spaniel American Water	96.86	<0.1
233	Polish Hound	88.06	<0.1
234	Portuguese Sheepdog	88.06	<0.1
235	Romanian Mioritic Shepherd Dog	85.12	<0.1
236	Saarloos Wolfhound	85.12	<0.1
237	Hungarian Kuvasz	82.19	<0.1
238	Australian Terrier	79.25	<0.1
239	Alpine Dachsbracke	76.32	<0.1
240	Azawakh	76.32	<0.1
241	Fila Brasileiro	73.38	<0.1
242	Tatra Shepherd Dog	70.45	<0.1
243	German Spitz Giant	67.51	<0.1
244	Bohemian Wire Haired Pointing Griffon	64.58	<0.1
245	Italian Short Haired Segugio	61.64	<0.1
246	Swedish Lapphund	58.71	<0.1
247	Austrian Pinscher	55.77	<0.1
248	Mudi	52.84	<0.1
249	Bouvier Des Ardennes	49.9	<0.1
250	Croatian Shepherd Dog	49.9	<0.1
251	French Spaniel	49.9	<0.1
252	German Roughhaired Pointer	46.96	<0.1
253	Hungarian Greyhound	41.09	<0.1
254	Italian Volpino	41.09	<0.1
255	Norwegian Lundehund	41.09	<0.1
256	Castro Laboreiro Dog	38.16	<0.1
257	Hungarian Hound Transylvanian Scent Hound	38.16	<0.1
258	Jamthund	38.16	<0.1
259	Shikoku	35.22	<0.1
260	Pyrenean Mastiff	32.29	<0.1

261	Serbian Hound	32.29	<0.1
262	Blue Picardy Spaniel	29.35	<0.1
263	Hanoverian Scent Hound	29.35	<0.1
264	Kromfohrlander	29.35	<0.1
265	Bosnian Broken Haired Hound	23.48	<0.1
266	Bourbonnais Pointing Dog	23.48	<0.1
267	Hokkaido	23.48	<0.1
268	Landseer	23.48	<0.1
269	South Russian Shepherd Dog	23.48	<0.1
270	Griffon Bleu De Gascogne	20.55	<0.1
271	Peruvian Hairless Dog	20.55	<0.1
272	Ariegeois	17.61	<0.1
273	Atlas Mountain Dog	17.61	<0.1
274	Austrian Black And Tan Hound	17.61	<0.1
275	Frisian Water Dog	17.61	<0.1
276	German Spaniel	17.61	<0.1
277	Grand Bleu Gascogne	17.61	<0.1
278	Majorca Mastiff	17.61	<0.1
279	Norrbottenspitz	17.61	<0.1
280	Petit Brabancon	17.61	<0.1
281	Polish Greyhound	17.61	<0.1
282	Posavatz Hound	17.61	<0.1
283	Slovakian Hound	17.61	<0.1
284	Tosa	17.61	<0.1
285	Danish Swedish Farmdog	14.68	<0.1
286	French Pointing Dog Pyrenean	14.68	<0.1
287	German Hound	14.68	<0.1
288	Griffon Nivernais	14.68	<0.1
289	Istrian Short Haired Hound	14.68	<0.1
290	Lapponian Herder	14.68	<0.1
291	Majorca Shepherd Dog	14.68	<0.1
292	Rafeiro do Alentejo	14.68	<0.1
293	Artois Hound	11.74	<0.1
294	Basset Artesien Normand	11.74	<0.1
295	Brazilian Terrier	11.74	<0.1
296	Briquet Griffon Vendeen	11.74	<0.1
297	Burgos Pointing Dog	11.74	<0.1
298	Drentsche Partridge Dog	11.74	<0.1
299	East Siberia Laika	11.74	<0.1
300	French White And Black Hound	11.74	<0.1
301	Karst Shepherd Dog	11.74	<0.1
302	Norwegian Hound	11.74	<0.1
303	Pudelpointer	11.74	<0.1
304	West Siberian Laika	11.74	<0.1

305	Westphalian Dachsbracke	11.74	<0.1
306	Billy	8.81	<0.1
307	Dutch Smoushond	8.81	<0.1
308	Icelandic Sheepdog	8.81	<0.1
309	Kishu	8.81	<0.1
310	Picardy Spaniel	8.81	<0.1
311	Taiwan Dog	8.81	<0.1
312	Ariege Pointing Dog	5.87	<0.1
313	Cimarron Uruguayo	5.87	<0.1
314	Great Anglo French Hound	5.87	<0.1
315	Kai	5.87	<0.1
316	Karelian Bear Dog	5.87	<0.1
317	Old Danish Pointing Dog	5.87	<0.1
318	Serbian Tricolour Hound	5.87	<0.1
319	Drever	2.94	<0.1
320	Finnish Hound	2.94	<0.1
321	French Tricolour Hound	2.94	<0.1
322	Gascon Saintongeois	2.94	<0.1
323	Japanese Terrier	2.94	<0.1
324	Petit Bleu De Gascogne	2.94	<0.1
325	Pont Audemer Spaniel	2.94	<0.1
326	Russian European Laika	2.94	<0.1
327	Saint Germain Pointer	2.94	<0.1
328	Saint Miguel Cattle Dog	2.94	<0.1
329	Schillerstovare	2.94	<0.1
330	Slovakian Chuvach	2.94	<0.1
331	Tyrolean Hound	2.94	<0.1

Supplementary Table 5. UK 2019 dog population estimate, per crossbred, and associated proportional breed demographics (%), ranked by popularity. Crossbreds of an equal estimated population size are secondary ranked alphabetically.

Rank	Crossbreds	Population Estimate	Proportion of UK Population (%)
1	Mix Breed	706823.44	39.3
2	Border Collie Cross/Type	81677.35	4.5
3	Poodle X Spaniel Cocker	73173.82	4.1
4	Staffordshire Bull Terrier Cross/Type	72437.06	4.0
5	Retriever Labrador Cross/Type	66035.19	3.7
6	Jack Russell Terrier Cross/Type	54009.32	3.0
7	Poodle X Retriever Labrador	44953.95	2.5
8	Chihuahua Smooth Coat Cross/Type	44472.56	2.5
9	Rottweiler Cross/Type	34504.32	1.9
10	Spaniel Cocker Cross/Type	30054.42	1.7
11	Bulldog Cross/Type	26869.63	1.5
12	German Shepherd Dog Cross/Type	26229.74	1.5
13	Shih Tzu Cross/Type	25654.43	1.4
14	Yorkshire Terrier Cross/Type	22654.56	1.3
15	Cavalier King Charles Spaniel X Poodle	19369.97	1.1
16	Spaniel Cocker X Spaniel English Springer	18639.08	1.0
17	Pug Cross/Type	17661.63	1.0
18	Poodle Cross/Type	16716.47	0.9
19	Bichon Frise Cross/Type	16654.83	0.9
20	Siberian Husky Cross/Type	15022.81	0.8
21	Spaniel English Springer Cross/Type	14808.53	0.8
22	Bichon Frise X Cavalier King Charles Spaniel	14597.19	0.8
23	Pomeranian Cross/Type	11444.69	0.6
24	Bull Terrier X Staffordshire Bull Terrier	10939.82	0.6
25	Bullmastiff X Dogue De Bordeaux	9777.45	0.5
26	Chihuahua Smooth Coat X Yorkshire Terrier	9495.66	0.5
27	Maltese Cross/Type	9049.5	0.5
28	Border Terrier Cross/Type	8676.71	0.5
29	Lhasa Apso Cross/Type	8594.53	0.5
30	Jack Russell Terrier X Pug	8389.06	0.5
31	Whippet Cross/Type	8233.49	0.5
32	West Highland White Terrier Cross/Type	8060.3	0.4
33	Jack Russell Terrier X Poodle	7479.12	0.4
34	Cavalier King Charles Spaniel Cross/Type	7185.59	0.4
35	Poodle X Retriever Golden	7006.53	0.4
36	Mastiff Cross/Type	6815.74	0.4
37	Beagle Cross/Type	6492.86	0.4
38	Boxer Cross/Type	6442.96	0.4
39	Beagle X Pug	6222.81	0.3
40	Bullmastiff Cross/Type	6214.01	0.3

41	Dachshund Smooth Haired Cross/Type	5732.62	0.3
42	French Bulldog Cross/Type	5553.57	0.3
43	Poodle X Shih Tzu	4899	0.3
44	Shar Pei Cross/Type	4684.72	0.3
45	Jack Russell Terrier X Parson Russell Terrier	4291.39	0.2
46	Lakeland Terrier Cross/Type	4006.67	0.2
47	Greyhound Cross/Type	3812.94	0.2
48	American Akita Cross/Type	3789.46	0.2
49	Saluki Cross/Type	3669.11	0.2
50	Bedlington Terrier Cross/Type	3381.45	0.2
51	Cairn Terrier Cross/Type	3360.91	0.2
52	American Staffordshire Terrier Cross/Type	3355.04	0.2
53	Dogue De Bordeaux Cross/Type	3319.81	0.2
54	Dobermann X German Pinscher	3302.2	0.2
55	Jack Russell Terrier X Yorkshire Terrier	3278.72	0.2
56	Alaskan Malamute Cross/Type	3240.56	0.2
57	Retriever Golden Cross/Type	3173.05	0.2
58	Chihuahua Smooth Coat X Jack Russell Terrier	2944.09	0.2
59	Spaniel English Springer X Spaniel Welsh Springer	2917.68	0.2
60	Pointer Cross/Type	2820.81	0.2
61	Bull Terrier Cross/Type	2744.5	0.2
62	Border Collie X Retriever Labrador	2703.4	0.2
63	Boxer X Staffordshire Bull Terrier	2550.77	0.1
64	Chihuahua Long Coat Cross/Type	2480.32	0.1
65	Rhodesian Ridgeback Cross/Type	2471.51	0.1
66	Bearded Collie Cross/Type	2448.03	0.1
67	Dalmatian Cross/Type	2386.39	0.1
68	Maltese X Poodle	2222.01	0.1
69	Welsh Corgi Pembroke Cross/Type	2216.14	0.1
70	Poodle X Schnauzer	2125.15	0.1
71	Border Collie X Staffordshire Bull Terrier	2119.28	0.1
72	King Charles Spaniel Cross/Type	2119.28	0.1
73	Bulldog X Bull Terrier	1975.45	0.1
74	Dobermann Cross/Type	1963.71	0.1
75	Scottish Terrier Cross/Type	1954.9	0.1
76	Miniature Schnauzer Cross/Type	1902.07	0.1
77	Border Collie X Jack Russell Terrier	1893.26	0.1
78	Schnauzer Cross/Type	1893.26	0.1
79	German Shorthaired Pointer X Weimaraner	1784.66	0.1
80	Bulldog X Staffordshire Bull Terrier	1769.98	0.1
81	Bichon Frise X Shih Tzu	1761.17	0.1
82	Cavalier King Charles Spaniel X Pug	1758.24	0.1
83	Poodle X Spaniel English Springer	1752.37	0.1
84	Basset Hound Cross/Type	1711.27	0.1

85	Retriever Labrador X Spaniel Cocker	1681.92	0.1
86	Chinese Crested Cross/Type	1508.74	0.1
87	Great Dane X Mastiff	1491.13	0.1
88	Japanese Akita Inu Cross/Type	1467.64	0.1
89	Bullmastiff X Spanish Mastiff	1370.78	0.1
90	Newfoundland Cross/Type	1364.91	0.1
91	Boston Terrier Cross/Type	1332.62	0.1
92	Belgian Shepherd Dog Malinois Cross/Type	1326.75	0.1
93	Collie Smooth Cross/Type	1317.94	0.1
94	Greyhound X Saluki	1309.14	0.1
95	Great Dane Cross/Type	1282.72	0.1
96	Poodle X West Highland White Terrier	1282.72	0.1
97	Retriever Labrador X Siberian Husky	1256.3	0.1
98	Fox Terrier Wire Cross/Type	1238.69	0.1
99	German Shepherd Dog X Rottweiler	1182.92	0.1
100	Tibetan Terrier Cross/Type	1174.12	0.1
101	Papillon Cross/Type	1138.89	0.1
102	Norfolk Terrier Cross/Type	1130.09	0.1
103	Retriever Labrador X Spaniel English Springer	1080.19	0.1
104	Dachshund Miniature Smooth Haired Cross/Type	1077.25	0.1
105	Border Terrier X Lakeland Terrier	1059.64	0.1
106	Weimaraner Cross/Type	1030.29	0.1
107	German Shorthaired Pointer Cross/Type	998	0.1
108	Italian Cane Corso X Mastiff	968.65	0.1
109	Miniature Pinscher Cross/Type	962.77	0.1
110	Bichon Frise X Yorkshire Terrier	956.9	0.1
111	Pekingese Cross/Type	921.68	0.1
112	Parson Russell Terrier Cross/Type	912.87	0.1
113	Poodle X Yorkshire Terrier	901.13	0.1
114	Australian Kelpie Cross/Type	857.1	<0.1
115	Collie Rough Cross/Type	851.23	<0.1
116	Hungarian Vizsla Cross/Type	845.36	<0.1
117	Retriever Labrador X Staffordshire Bull Terrier	733.82	<0.1
118	Presa Canario Cross/Type	713.28	<0.1
119	Dobermann X Miniature Pinscher	692.73	<0.1
120	Jack Russell Terrier X Lakeland Terrier	669.25	<0.1
121	Shetland Sheepdog Cross/Type	663.38	<0.1
122	Border Terrier X Yorkshire Terrier	648.7	<0.1
123	German Spitz Mittel Cross/Type	642.83	<0.1
124	Deerhound Cross/Type	631.09	<0.1
125	Mastiff X Staffordshire Bull Terrier	601.73	<0.1
126	Old English Sheepdog Cross/Type	598.8	<0.1
127	Retriever Flat Coated Cross/Type	566.51	<0.1
128	Bichon Frise X Bolognese	543.03	<0.1

129	Irish Wolfhound Cross/Type	540.09	<0.1
130	Chihuahua Smooth Coat X Papillon	537.16	<0.1
131	St Bernard Cross/Type	531.29	<0.1
132	Retriever Golden X Retriever Labrador	504.87	<0.1
133	Bichon Frise X Poodle	499	<0.1
134	Foxhound Cross/Type	481.39	<0.1
135	Chow Chow Cross/Type	472.58	<0.1
136	Italian Greyhound Cross/Type	472.58	<0.1
137	Samoyed Cross/Type	463.78	<0.1
138	Poodle X Pug	460.84	<0.1
139	Jack Russell Terrier X Staffordshire Bull Terrier	452.03	<0.1
140	Manchester Terrier Cross/Type	434.42	<0.1
141	Griffon Bruxellois Cross/Type	428.55	<0.1
142	Cavalier King Charles Spaniel X Spaniel American Cocker	416.81	<0.1
143	Neapolitan Mastiff Cross/Type	402.13	<0.1
144	Bichon Frise X Havanese	390.39	<0.1
145	Coton De Tulear Cross/Type	390.39	<0.1
146	Schipperke X Siberian Husky	390.39	<0.1
147	Border Terrier X Jack Russell Terrier	381.59	<0.1
148	Basset Hound X Spaniel English Springer	378.65	<0.1
149	German Shepherd Dog X Retriever Labrador	378.65	<0.1
150	Japanese Chin Cross/Type	375.72	<0.1
151	Shih Tzu X Yorkshire Terrier	369.85	<0.1
152	Cavalier King Charles Spaniel X Pomeranian	366.91	<0.1
153	Irish Red And White Setter Cross/Type	363.98	<0.1
154	English Setter Cross/Type	361.04	<0.1
155	Havanese Cross/Type	352.23	<0.1
156	Spaniel American Cocker Cross/Type	337.55	<0.1
157	Airedale Terrier Cross/Type	319.95	<0.1
158	Border Collie X German Shepherd Dog	308.21	<0.1
159	Chihuahua Smooth Coat X Poodle	308.21	<0.1
160	Bernese Mountain Dog Cross/Type	305.27	<0.1
161	Bullmastiff X Staffordshire Bull Terrier	287.66	<0.1
162	Welsh Terrier Cross/Type/type	287.66	<0.1
163	Maltese X Shih Tzu	281.79	<0.1
164	Tibetan Spaniel Cross/Type	278.85	<0.1
165	German Shepherd Dog X Siberian Husky	275.92	<0.1
166	Rottweiler X Staffordshire Bull Terrier	275.92	<0.1
167	Dogue De Bordeaux X Retriever Labrador	264.18	<0.1
168	Alaskan Malamute X Siberian Husky	255.37	<0.1
169	Shar Pei X Staffordshire Bull Terrier	252.43	<0.1
170	Irish Terrier Cross/Type	246.56	<0.1
171	Chihuahua Smooth Coat X Shih Tzu	237.76	<0.1
172	Romanian Carpathian Shepherd Dog Cross/Type	234.82	<0.1

173	Jack Russell Terrier X Shih Tzu	231.89	<0.1
174	Beagle X Jack Russell Terrier	228.95	<0.1
175	German Shepherd Dog X Staffordshire Bull Terrier	226.02	<0.1
176	Maltese X Yorkshire Terrier	226.02	<0.1
177	Border Collie X Spaniel English Springer	223.08	<0.1
178	Boston Terrier X Bulldog	220.15	<0.1
179	Chihuahua Smooth Coat X Pomeranian	217.21	<0.1
180	Landseer X Newfoundland	214.28	<0.1
181	Pomeranian X Shih Tzu	214.28	<0.1
182	Rhodesian Ridgeback X Staffordshire Bull Terrier	214.28	<0.1
183	Soft Coated Wheaten Terrier Cross/Type	211.34	<0.1
184	Boxer X Bull Terrier	205.47	<0.1
185	Leonberger Cross/Type	196.66	<0.1
186	Australian Cattle Dog Cross/Type	193.73	<0.1
187	Chihuahua Smooth Coat X Pug	193.73	<0.1
188	French Bulldog X Pug	190.79	<0.1
189	Lancashire Heeler Cross/Type	190.79	<0.1
190	Australian Shepherd Cross/Type	187.86	<0.1
191	Jack Russell Terrier X West Highland White Terrier	187.86	<0.1
192	Japanese Spitz Cross/Type	187.86	<0.1
193	Pug X Shih Tzu	184.92	<0.1
194	Staffordshire Bull Terrier X Yorkshire Terrier	184.92	<0.1
195	German Shepherd Dog X Poodle	181.99	<0.1
196	Dachshund Smooth Haired X Jack Russell Terrier	179.05	<0.1
197	Jack Russell Terrier X Pomeranian	170.25	<0.1
198	Portuguese Podengo Cross/Type	170.25	<0.1
199	Staffordshire Bull Terrier X Whippet	170.25	<0.1
200	Spaniel Clumber Cross/Type	167.31	<0.1
201	Retriever Labrador X Rottweiler	164.38	<0.1
202	Black And Tan Coonhound Cross/Type	161.44	<0.1
203	German Wirehaired Pointer Cross/Type	161.44	<0.1
204	Brittany Cross/Type	152.64	<0.1
205	Canarian Warren Hound Cross/Type	149.7	<0.1
206	Dachshund Miniature Long Haired Cross/Type	146.76	<0.1
207	Australian Silky Terrier Cross/Type	143.83	<0.1
208	Border Collie X Spaniel Cocker	137.96	<0.1
209	Spanish Mastiff Cross/Type	137.96	<0.1
210	Bichon Frise X Maltese	135.02	<0.1
211	Cavalier King Charles Spaniel X Retriever Labrador	132.09	<0.1
212	Lhasa Apso X Maltese	132.09	<0.1
213	Pekingese X Poodle	132.09	<0.1
214	Pyrenean Mountain Dog Cross/Type	132.09	<0.1
215	Cavalier King Charles Spaniel X Shih Tzu	129.15	<0.1
216	Giant Schnauzer Cross/Type	123.28	<0.1

217	Anatolian Shepherd Dog Cross/Type	120.35	<0.1
218	Beauceron Cross/Type	120.35	<0.1
219	Bloodhound Cross/Type	120.35	<0.1
220	Retriever Labrador X Whippet	120.35	<0.1
221	Spanish Water Dog Cross/Type	120.35	<0.1
222	Bulldog X Mastiff	114.48	<0.1
223	Caucasian Shepherd Dog Cross/Type	114.48	<0.1
224	Anatolian Shepherd Dog X Turkish Kangal Dog	111.54	<0.1
225	Dutch Shepherd Dog Cross/Type	108.61	<0.1
226	Jack Russell Terrier X Welsh Corgi Pembroke	105.67	<0.1
227	Pointer X Retriever Labrador	105.67	<0.1
228	Bichon Frise X Miniature Schnauzer	102.74	<0.1
229	Boxer X Retriever Labrador	102.74	<0.1
230	Pointer X Small Münsterländer	102.74	<0.1
231	Pomeranian X Poodle	102.74	<0.1
232	Siberian Husky X Staffordshire Bull Terrier	102.74	<0.1
233	Bichon Frise X West Highland White Terrier	99.8	<0.1
234	Border Collie X Siberian Husky	99.8	<0.1
235	Bullmastiff X Rottweiler	99.8	<0.1
236	Afghan Hound Cross/Type	96.86	<0.1
237	Czechoslovakian Wolfdog Cross/Type	96.86	<0.1
238	Japanese Shiba Inu Cross/Type	96.86	<0.1
239	Affenpinscher Cross/Type	93.93	<0.1
240	Affenpinscher X Yorkshire Terrier	93.93	<0.1
241	Beagle X Cavalier King Charles Spaniel	93.93	<0.1
242	American Akita X German Shepherd Dog	90.99	<0.1
243	Bichon Frise X Jack Russell Terrier	90.99	<0.1
244	Lhasa Apso X Yorkshire Terrier	88.06	<0.1
245	Retriever Labrador X Spaniel American Cocker	88.06	<0.1
246	Chihuahua Smooth Coat X Dachshund Smooth Haired	85.12	<0.1
247	Chihuahua Smooth Coat X Maltese	85.12	<0.1
248	Harrier Cross/Type	85.12	<0.1
249	Lhasa Apso X Shih Tzu	82.19	<0.1
250	Beagle X Harrier	79.25	<0.1
251	Hellenic Hound Cross/Type	79.25	<0.1
252	Basenji Cross/Type	76.32	<0.1
253	Bichon Frise X Lhasa Apso	76.32	<0.1
254	Gordon Setter Cross/Type	76.32	<0.1
255	Kerry Blue Terrier Cross/Type	76.32	<0.1
256	Miniature Schnauzer X Yorkshire Terrier	76.32	<0.1
257	West Highland White Terrier X Yorkshire Terrier	76.32	<0.1
258	Bulldog X Bullmastiff	73.38	<0.1
259	German Pinscher Cross/Type	73.38	<0.1
260	Hungarian Puli Cross/Type	73.38	<0.1

261	Pomeranian X Siberian Husky	73.38	<0.1
262	Russian Black Terrier Cross/Type	73.38	<0.1
263	American Akita X Staffordshire Bull Terrier	70.45	<0.1
264	Italian Spinone Cross/Type	70.45	<0.1
265	Bedlington Terrier X Whippet	67.51	<0.1
266	Bulldog X Pekingese	67.51	<0.1
267	Dachshund Miniature Wire Haired Cross/Type	67.51	<0.1
268	Jack Russell Terrier X Spaniel English Springer	67.51	<0.1
269	Saluki X Whippet	67.51	<0.1
270	Basset Griffon Vendeen Petit Cross/Type	64.58	<0.1
271	Cavalier King Charles Spaniel X Chihuahua Smooth Coat	64.58	<0.1
272	Mastiff X Rottweiler	64.58	<0.1
273	Alaskan Malamute X German Shepherd Dog	61.64	<0.1
274	Finnish Spitz Cross/Type	61.64	<0.1
275	French Bulldog X Staffordshire Bull Terrier	61.64	<0.1
276	Bolognese Cross/Type	58.71	<0.1
277	Dachshund Wire Haired Cross/Type	58.71	<0.1
278	German Longhaired Pointer Cross/Type	58.71	<0.1
279	Jack Russell Terrier X Lhasa Apso	58.71	<0.1
280	Pomeranian X Yorkshire Terrier	58.71	<0.1
281	Thai Ridgeback Dog Cross/Type	58.71	<0.1
282	Welsh Corgi Cardigan Cross/Type	58.71	<0.1
283	Dogue De Bordeaux X Staffordshire Bull Terrier	55.77	<0.1
284	Norwich Terrier Cross/Type	55.77	<0.1
285	Sealyham Terrier Cross/Type	55.77	<0.1
286	Bichon Frise X Chihuahua Smooth Coat	52.84	<0.1
287	Boxer X Mastiff	52.84	<0.1
288	Briard Cross/Type	52.84	<0.1
289	Cairn Terrier X Yorkshire Terrier	52.84	<0.1
290	Collie Rough X Collie Smooth	52.84	<0.1
291	Dalmatian X Retriever Labrador	52.84	<0.1
292	Löwchen Cross/Type	52.84	<0.1
293	Norwegian Elkhound Cross/Type	52.84	<0.1
294	Skye Terrier Cross/Type	52.84	<0.1
295	Spaniel Irish Water Cross/Type	52.84	<0.1
296	Tibetan Mastiff Cross/Type	52.84	<0.1
297	Border Collie X Dachshund Smooth Haired	49.9	<0.1
298	Border Collie X Poodle	49.9	<0.1
299	Boxer X Bullmastiff	49.9	<0.1
300	Cairn Terrier X Jack Russell Terrier	49.9	<0.1
301	Catalan Sheepdog Cross/Type	49.9	<0.1
302	Chihuahua Smooth Coat X Miniature Pinscher	49.9	<0.1
303	Ibizan Hound Cross/Type	49.9	<0.1
304	Jack Russell Terrier X Spaniel Cocker	49.9	<0.1

305	Lakeland Terrier X Yorkshire Terrier	49.9	<0.1
306	Lhasa Apso X Poodle	49.9	<0.1
307	Retriever Labrador X Rhodesian Ridgeback	49.9	<0.1
308	Spaniel Field Cross/Type	49.9	<0.1
309	Border Collie X Lakeland Terrier	46.96	<0.1
310	Borzoi X Deerhound	46.96	<0.1
311	Cavalier King Charles Spaniel X Jack Russell Terrier	46.96	<0.1
312	Chihuahua Smooth Coat X French Bulldog	46.96	<0.1
313	Chihuahua Smooth Coat X Pekingese	46.96	<0.1
314	Dachshund Long Haired Cross/Type	46.96	<0.1
315	Dobermann X Retriever Labrador	46.96	<0.1
316	Dogue De Bordeaux X Rottweiler	46.96	<0.1
317	English Toy Terrier Cross/Type	46.96	<0.1
318	Greyhound X Whippet	46.96	<0.1
319	Irish Setter Cross/Type	46.96	<0.1
320	Picardy Sheepdog Cross/Type	46.96	<0.1
321	Bichon Frise X Pug	44.03	<0.1
322	Bulldog X Scottish Terrier	44.03	<0.1
323	Sloughi Cross/Type	44.03	<0.1
324	Bedlington Terrier X Greyhound	41.09	<0.1
325	Border Collie X Whippet	41.09	<0.1
326	Bull Terrier Miniature Cross/Type	41.09	<0.1
327	Bulldog X Rottweiler	41.09	<0.1
328	Cavalier King Charles Spaniel X Spaniel Cocker	41.09	<0.1
329	Chihuahua Smooth Coat X Lhasa Apso	41.09	<0.1
330	Croatian Shepherd Dog Cross/Type	41.09	<0.1
331	Dachshund Smooth Haired X Poodle	41.09	<0.1
332	Dobermann X Rottweiler	41.09	<0.1
333	Large Münsterländer Cross/Type	41.09	<0.1
334	Poodle X Spaniel American Cocker	41.09	<0.1
335	Pug X Yorkshire Terrier	41.09	<0.1
336	Spanish Greyhound Cross/Type	41.09	<0.1
337	American Akita X Siberian Husky	38.16	<0.1
338	Bichon Frise X Pomeranian	38.16	<0.1
339	Bulldog X French Bulldog	38.16	<0.1
340	Bulldog X Pug	38.16	<0.1
341	Cesky Terrier Cross/Type	38.16	<0.1
342	Dandie Dinmont Terrier Cross/Type	38.16	<0.1
343	Dobermann X Staffordshire Bull Terrier	38.16	<0.1
344	German Shepherd Dog X Mastiff	38.16	<0.1
345	Russian Toy Cross/Type	38.16	<0.1
346	Bichon Frise X Spaniel Cocker	35.22	<0.1
347	Border Collie X Rottweiler	35.22	<0.1
348	Canadian Eskimo Dog Cross/Type	35.22	<0.1

349	Chihuahua Smooth Coat X Chinese Crested	35.22	<0.1
350	Glen Of Imaal Terrier Cross/Type	35.22	<0.1
351	Hungarian Vizsla X Retriever Labrador	35.22	<0.1
352	Miniature Schnauzer X Poodle	35.22	<0.1
353	Poodle X Shetland Sheepdog	35.22	<0.1
354	Retriever Chesapeake Bay Cross/Type	35.22	<0.1
355	Retriever Curly Coated Cross/Type	35.22	<0.1
356	Retriever Nova Scotia Duck Tolling Cross/Type	35.22	<0.1
357	American Akita X Rottweiler	32.29	<0.1
358	Belgian Shepherd Dog Groenendael Cross/Type	32.29	<0.1
359	Bulldog X Dogue De Bordeaux	32.29	<0.1
360	Dogue De Bordeaux X Mastiff	32.29	<0.1
361	French Bulldog X Shih Tzu	32.29	<0.1
362	German Shepherd Dog X Greyhound	32.29	<0.1
363	Italian Spinone X Schnauzer	32.29	<0.1
364	Jack Russell Terrier X Maltese	32.29	<0.1
365	Jack Russell Terrier X Norfolk Terrier	32.29	<0.1
366	Korthals Griffon Cross/Type	32.29	<0.1
367	Mastiff X Retriever Labrador	32.29	<0.1
368	Papillon X Pomeranian	32.29	<0.1
369	Portuguese Pointer Cross/Type	32.29	<0.1
370	Shih Tzu X West Highland White Terrier	32.29	<0.1
371	Border Collie X Retriever Golden	29.35	<0.1
372	Bouvier Des Flandres Cross/Type	29.35	<0.1
373	Bulldog X Shar Pei	29.35	<0.1
374	Jack Russell Terrier X Whippet	29.35	<0.1
375	King Charles Spaniel X Shih Tzu	29.35	<0.1
376	Pharaoh Hound Cross/Type	29.35	<0.1
377	Pomeranian X Pug	29.35	<0.1
378	Portuguese Water Dog Cross/Type	29.35	<0.1
379	Siberian Husky X Weimaraner	29.35	<0.1
380	Spaniel Clumber X Spaniel English Springer	29.35	<0.1
381	Spaniel English Springer X Staffordshire Bull Terrier	29.35	<0.1
382	Spaniel Sussex Cross/Type	29.35	<0.1
383	Swiss Hound Cross/Type	29.35	<0.1
384	American Akita X Retriever Labrador	26.42	<0.1
385	Basset Hound X Retriever Labrador	26.42	<0.1
386	Basset Hound X Shar Pei	26.42	<0.1
387	Beagle X Retriever Labrador	26.42	<0.1
388	Border Collie X Welsh Corgi Pembroke	26.42	<0.1
389	Border Terrier X Poodle	26.42	<0.1
390	Boston Terrier X Pug	26.42	<0.1
391	Boxer X Bulldog	26.42	<0.1
392	Bulldog X Retriever Labrador	26.42	<0.1

393	Dachshund Miniature Smooth Haired X Jack Russell Terrier	26.42	<0.1
394	Jack Russell Terrier X King Charles Spaniel	26.42	<0.1
395	Keeshond Cross/Type	26.42	<0.1
396	Mastiff X Rhodesian Ridgeback	26.42	<0.1
397	Miniature Pinscher X Pug	26.42	<0.1
398	Retriever Labrador X Shar Pei	26.42	<0.1
399	Rottweiler X Siberian Husky	26.42	<0.1
400	Schnauzer X Spaniel Cocker	26.42	<0.1
401	Beagle X Staffordshire Bull Terrier	23.48	<0.1
402	Border Collie X Saluki	23.48	<0.1
403	Border Terrier X West Highland White Terrier	23.48	<0.1
404	Borzoi Cross/Type	23.48	<0.1
405	German Shepherd Dog X Rhodesian Ridgeback	23.48	<0.1
406	German Spitz Mittel X Pomeranian	23.48	<0.1
407	Jack Russell Terrier X Retriever Labrador	23.48	<0.1
408	Otterhound Cross/Type	23.48	<0.1
409	Schnauzer X West Highland White Terrier	23.48	<0.1
410	Bosnian Broken Haired Hound Cross/Type	20.55	<0.1
411	Boston Terrier X French Bulldog	20.55	<0.1
412	Boxer X Rottweiler	20.55	<0.1
413	Cairn Terrier X West Highland White Terrier	20.55	<0.1
414	Canaan Dog Cross/Type	20.55	<0.1
415	Central Asia Shepherd Dog Cross/Type	20.55	<0.1
416	German Shepherd Dog X Retriever Golden	20.55	<0.1
417	German Shepherd Dog X Spaniel English Springer	20.55	<0.1
418	Lhasa Apso X Pug	20.55	<0.1
419	Maltese X West Highland White Terrier	20.55	<0.1
420	Maremma Sheepdog Cross/Type	20.55	<0.1
421	Norfolk Terrier X Yorkshire Terrier	20.55	<0.1
422	Pekingese X Shih Tzu	20.55	<0.1
423	Spaniel English Springer X Weimaraner	20.55	<0.1
424	Spanish Hound Cross/Type	20.55	<0.1
425	Affenpinscher X Poodle	17.61	<0.1
426	American Akita X Mastiff	17.61	<0.1
427	Beagle X Kerry Blue Terrier	17.61	<0.1
428	Beagle X Pointer	17.61	<0.1
429	Beagle X Spaniel Cocker	17.61	<0.1
430	Belgian Shepherd Dog Tervueren Cross/Type	17.61	<0.1
431	Bichon Frise X Schnauzer	17.61	<0.1
432	Border Collie X Dobermann	17.61	<0.1
433	Border Terrier X Pug	17.61	<0.1
434	Boxer X Great Dane	17.61	<0.1
435	Bulldog X German Shepherd Dog	17.61	<0.1
436	Bulldog X Saluki	17.61	<0.1

437	Bullmastiff X Great Dane	17.61	<0.1
438	Coton De Tulear X Poodle	17.61	<0.1
439	Dutch Shepherd Dog X Poodle	17.61	<0.1
440	Fox Terrier Smooth Cross/Type	17.61	<0.1
441	Fox Terrier Wire X Jack Russell Terrier	17.61	<0.1
442	Greyhound X Staffordshire Bull Terrier	17.61	<0.1
443	Jack Russell Terrier X Schnauzer	17.61	<0.1
444	Kooikerhondje Cross/Type	17.61	<0.1
445	Large Münsterländer X Pointer	17.61	<0.1
446	Majorca Mastiff Cross/Type	17.61	<0.1
447	Maltese X Pomeranian	17.61	<0.1
448	Miniature Schnauzer X West Highland White Terrier	17.61	<0.1
449	Norrbottenspitz Cross/Type	17.61	<0.1
450	Papillon X Shih Tzu	17.61	<0.1
451	Pointer X Spaniel English Springer	17.61	<0.1
452	Scottish Terrier X West Highland White Terrier	17.61	<0.1
453	Scottish Terrier X Yorkshire Terrier	17.61	<0.1
454	Shih Tzu X Spaniel Cocker	17.61	<0.1
455	American Akita X Shar Pei	14.68	<0.1
456	Australian Kelpie X Border Collie	14.68	<0.1
457	Austrian Pinscher Cross/Type	14.68	<0.1
458	Bavarian Mountain Hound Cross/Type	14.68	<0.1
459	Beagle X Boston Terrier	14.68	<0.1
460	Beagle X Poodle	14.68	<0.1
461	Beagle X Spaniel English Springer	14.68	<0.1
462	Belgian Shepherd Dog Malinois X German Shepherd Dog	14.68	<0.1
463	Belgian Shepherd Dog Malinois X Siberian Husky	14.68	<0.1
464	Bernese Mountain Dog X Newfoundland	14.68	<0.1
465	Border Collie X Yorkshire Terrier	14.68	<0.1
466	Boxer X German Shepherd Dog	14.68	<0.1
467	Cairn Terrier X Norfolk Terrier	14.68	<0.1
468	Cavalier King Charles Spaniel X Lhasa Apso	14.68	<0.1
469	Cavalier King Charles Spaniel X Yorkshire Terrier	14.68	<0.1
470	Dachshund Smooth Haired X Yorkshire Terrier	14.68	<0.1
471	Dobermann X German Shepherd Dog	14.68	<0.1
472	Great Swiss Mountain Dog Cross/Type	14.68	<0.1
473	Greyhound X Retriever Labrador	14.68	<0.1
474	King Charles Spaniel X Pug	14.68	<0.1
475	King Charles Spaniel X Spaniel English Springer	14.68	<0.1
476	Lhasa Apso X West Highland White Terrier	14.68	<0.1
477	Mastiff X Siberian Husky	14.68	<0.1
478	Papillon X Spaniel Cocker	14.68	<0.1
479	Pekingese X Pug	14.68	<0.1
480	Pomeranian X Shetland Sheepdog	14.68	<0.1

481	Presa Canario X Staffordshire Bull Terrier	14.68	<0.1
482	Pug X Staffordshire Bull Terrier	14.68	<0.1
483	Retriever Golden X Spaniel Cocker	14.68	<0.1
484	Spaniel Cocker X Staffordshire Bull Terrier	14.68	<0.1
485	Staffordshire Bull Terrier X West Highland White Terrier	14.68	<0.1
486	American Akita X Dobermann	11.74	<0.1
487	Basset Fauve De Bretagne Cross/Type	11.74	<0.1
488	Basset Griffon Vendeen Grand Cross/Type	11.74	<0.1
489	Basset Hound X Spaniel Cocker	11.74	<0.1
490	Bedlington Terrier X Jack Russell Terrier	11.74	<0.1
491	Belgian Shepherd Dog Malinois X Caucasian Shepherd Dog	11.74	<0.1
492	Bichon Frise X Cairn Terrier	11.74	<0.1
493	Bichon Frise X Dachshund Smooth Haired	11.74	<0.1
494	Bichon Frise X Staffordshire Bull Terrier	11.74	<0.1
495	Border Collie X Boxer	11.74	<0.1
496	Border Collie X Greyhound	11.74	<0.1
497	Bull Terrier X Mastiff	11.74	<0.1
498	Bull Terrier X Whippet	11.74	<0.1
499	Bullmastiff X Rhodesian Ridgeback	11.74	<0.1
500	Cavalier King Charles Spaniel X West Highland White Terrier	11.74	<0.1
501	Chihuahua Smooth Coat X Staffordshire Bull Terrier	11.74	<0.1
502	Chihuahua Smooth Coat X West Highland White Terrier	11.74	<0.1
503	Dachshund Smooth Haired X Retriever Labrador	11.74	<0.1
504	Dalmatian X Staffordshire Bull Terrier	11.74	<0.1
505	Fox Terrier Wire X Poodle	11.74	<0.1
506	German Hound Cross/Type	11.74	<0.1
507	German Shepherd Dog X Jack Russell Terrier	11.74	<0.1
508	German Shepherd Dog X Japanese Akita Inu	11.74	<0.1
509	German Shepherd Dog X Newfoundland	11.74	<0.1
510	German Spitz Klein Cross/Type	11.74	<0.1
511	Greyhound X Sloughi	11.74	<0.1
512	Hungarian Hound Transylvanian Scent Hound Cross/Type	11.74	<0.1
513	Hungarian Kuvasz Cross/Type	11.74	<0.1
514	Irish Terrier X Pug	11.74	<0.1
515	Jack Russell Terrier X Miniature Schnauzer	11.74	<0.1
516	Jack Russell Terrier X Siberian Husky	11.74	<0.1
517	King Charles Spaniel X Spaniel Cocker	11.74	<0.1
518	Mudi Cross/Type	11.74	<0.1
519	Pyrenean Mastiff Cross/Type	11.74	<0.1
520	Rhodesian Ridgeback X Rottweiler	11.74	<0.1
521	Romanian Bucovina Shepherd Cross/Type	11.74	<0.1
522	Saarloos Wolfhound Cross/Type	11.74	<0.1
523	Schipperke Cross/Type	11.74	<0.1
524	Shih Tzu X Spaniel English Springer	11.74	<0.1

525	Spaniel English Springer X Whippet	11.74	<0.1
526	Stabijhoun Cross/Type	11.74	<0.1
527	Swedish Vallhund Cross/Type	11.74	<0.1
528	Xoloitzcuintle Cross/Type	11.74	<0.1
529	Yugoslavian Shepherd Dog Cross/Type	11.74	<0.1
530	Alpine Dachsbracke Cross/Type	8.81	<0.1
531	Auvergne Pointer Cross/Type	8.81	<0.1
532	Basset Hound X Beagle	8.81	<0.1
533	Basset Hound X Dachshund Smooth Haired	8.81	<0.1
534	Beagle X Border Collie	8.81	<0.1
535	Bearded Collie X Border Collie	8.81	<0.1
536	Bedlington Terrier X Poodle	8.81	<0.1
537	Bichon Frise X Border Terrier	8.81	<0.1
538	Bichon Frise X Retriever Labrador	8.81	<0.1
539	Border Collie X Bulldog	8.81	<0.1
540	Border Collie X Cairn Terrier	8.81	<0.1
541	Border Collie X Dalmatian	8.81	<0.1
542	Border Collie X Pointer	8.81	<0.1
543	Border Collie X Samoyed	8.81	<0.1
544	Border Collie X Shetland Sheepdog	8.81	<0.1
545	Border Terrier X Cairn Terrier	8.81	<0.1
546	Border Terrier X Miniature Schnauzer	8.81	<0.1
547	Border Terrier X Pomeranian	8.81	<0.1
548	Border Terrier X Shih Tzu	8.81	<0.1
549	Boxer X Pointer	8.81	<0.1
550	Boxer X Retriever Golden	8.81	<0.1
551	Boxer X Rhodesian Ridgeback	8.81	<0.1
552	Boxer X Spaniel Cocker	8.81	<0.1
553	Bulldog X Spanish Mastiff	8.81	<0.1
554	Bullmastiff X Retriever Labrador	8.81	<0.1
555	Bullmastiff X Siberian Husky	8.81	<0.1
556	Castro Laboreiro Dog Cross/Type	8.81	<0.1
557	Cavalier King Charles Spaniel X Spaniel English Springer	8.81	<0.1
558	Chihuahua Smooth Coat X German Spitz Mittel	8.81	<0.1
559	Chihuahua Smooth Coat X Russian Toy	8.81	<0.1
560	Dalmatian X Jack Russell Terrier	8.81	<0.1
561	Dutch Smoushond Cross/Type	8.81	<0.1
562	Eurasier Cross/Type	8.81	<0.1
563	German Shepherd Dog X Pointer	8.81	<0.1
564	Great Dane X Rottweiler	8.81	<0.1
565	Greyhound X Rottweiler	8.81	<0.1
566	Hamiltonstovare Cross/Type	8.81	<0.1
567	Hokkaido Cross/Type	8.81	<0.1
568	Hungarian Vizsla X Staffordshire Bull Terrier	8.81	<0.1

569	Irish Setter X Retriever Labrador	8.81	<0.1
570	Italian Greyhound X Whippet	8.81	<0.1
571	Italian Short Haired Segugio Cross/Type	8.81	<0.1
572	Jack Russell Terrier X Norwich Terrier	8.81	<0.1
573	Jack Russell Terrier X Pointer	8.81	<0.1
574	Jack Russell Terrier X Scottish Terrier	8.81	<0.1
575	Jack Russell Terrier X Tibetan Terrier	8.81	<0.1
576	Jamthund Cross/Type	8.81	<0.1
577	Japanese Spitz X Pomeranian	8.81	<0.1
578	King Charles Spaniel X Lhasa Apso	8.81	<0.1
579	King Charles Spaniel X Poodle	8.81	<0.1
580	Lakeland Terrier X West Highland White Terrier	8.81	<0.1
581	Lhasa Apso X Spaniel Cocker	8.81	<0.1
582	Norwegian Buhund Cross/Type	8.81	<0.1
583	Old English Sheepdog X Retriever Labrador	8.81	<0.1
584	Papillon X Yorkshire Terrier	8.81	<0.1
585	Pointer X Spaniel Cocker	8.81	<0.1
586	Polish Lowland Sheepdog Cross/Type	8.81	<0.1
587	Pomeranian X Welsh Corgi Pembroke	8.81	<0.1
588	Poodle X Retriever Flat Coated	8.81	<0.1
589	Poodle X Soft Coated Wheaten Terrier	8.81	<0.1
590	Poodle X St Bernard	8.81	<0.1
591	Poodle X Whippet	8.81	<0.1
592	Portuguese Sheepdog Cross/Type	8.81	<0.1
593	Pug X Shar Pei	8.81	<0.1
594	Pug X Spaniel Cocker	8.81	<0.1
595	Pyrenean Sheepdog Smooth Faced Cross/Type	8.81	<0.1
596	Retriever Flat Coated X Retriever Labrador	8.81	<0.1
597	Retriever Golden X Spaniel English Springer	8.81	<0.1
598	Retriever Labrador X Weimaraner	8.81	<0.1
599	Romanian Mioritic Shepherd Dog Cross/Type	8.81	<0.1
600	Schnauzer X Shih Tzu	8.81	<0.1
601	Spaniel American Cocker X Spaniel English Springer	8.81	<0.1
602	Spaniel American Water Cross/Type	8.81	<0.1
603	Spaniel Welsh Springer Cross/Type	8.81	<0.1
604	Staffordshire Bull Terrier X Welsh Corgi Pembroke	8.81	<0.1
605	White Swiss Shepherd Dog Cross/Type	8.81	<0.1
606	Alaskan Malamute X Rottweiler	5.87	<0.1
607	Atlas Mountain Dog Cross/Type	5.87	<0.1
608	Australian Cattle Dog X Staffordshire Bull Terrier	5.87	<0.1
609	Barbet Cross/Type	5.87	<0.1
610	Basset Hound X Poodle	5.87	<0.1
611	Basset Hound X Staffordshire Bull Terrier	5.87	<0.1
612	Beagle X Shih Tzu	5.87	<0.1

613	Bearded Collie X Greyhound	5.87	<0.1
614	Belgian Shepherd Dog Laekenois Cross/Type	5.87	<0.1
615	Belgian Shepherd Dog Malinois X Retriever Labrador	5.87	<0.1
616	Bernese Mountain Dog X Poodle	5.87	<0.1
617	Bichon Frise X Border Collie	5.87	<0.1
618	Bichon Frise X Griffon Bruxellois	5.87	<0.1
619	Bichon Frise X Miniature Pinscher	5.87	<0.1
620	Bichon Frise X Spaniel English Springer	5.87	<0.1
621	Bichon Frise X Tibetan Terrier	5.87	<0.1
622	Bloodhound X Retriever Labrador	5.87	<0.1
623	Border Collie X Border Terrier	5.87	<0.1
624	Border Collie X Collie Rough	5.87	<0.1
625	Border Collie X Old English Sheepdog	5.87	<0.1
626	Border Collie X Retriever Flat Coated	5.87	<0.1
627	Border Collie X Shar Pei	5.87	<0.1
628	Border Terrier X Chihuahua Smooth Coat	5.87	<0.1
629	Boston Terrier X Chihuahua Smooth Coat	5.87	<0.1
630	Boston Terrier X Jack Russell Terrier	5.87	<0.1
631	Boston Terrier X Shih Tzu	5.87	<0.1
632	Boxer X Dogue De Bordeaux	5.87	<0.1
633	Boxer X Greyhound	5.87	<0.1
634	Boxer X Shar Pei	5.87	<0.1
635	Boxer X Siberian Husky	5.87	<0.1
636	Boxer X Whippet	5.87	<0.1
637	Bracco Italiano Cross/Type	5.87	<0.1
638	Brazilian Terrier Cross/Type	5.87	<0.1
639	Broholmer X Mastiff	5.87	<0.1
640	Bull Terrier X Jack Russell Terrier	5.87	<0.1
641	Bull Terrier X Rottweiler	5.87	<0.1
642	Bulldog X Presa Canario	5.87	<0.1
643	Bullmastiff X German Shepherd Dog	5.87	<0.1
644	Cairn Terrier X Poodle	5.87	<0.1
645	Cairn Terrier X Scottish Terrier	5.87	<0.1
646	Cairn Terrier X Shih Tzu	5.87	<0.1
647	Cavalier King Charles Spaniel X Dachshund Smooth Haired	5.87	<0.1
648	Cavalier King Charles Spaniel X Maltese	5.87	<0.1
649	Chihuahua Smooth Coat X Griffon Bruxellois	5.87	<0.1
650	Chihuahua Smooth Coat X King Charles Spaniel	5.87	<0.1
651	Chihuahua Smooth Coat X Lakeland Terrier	5.87	<0.1
652	Chihuahua Smooth Coat X Welsh Corgi Pembroke	5.87	<0.1
653	Chinese Crested X Lhasa Apso	5.87	<0.1
654	Chinese Crested X Maltese	5.87	<0.1
655	Chinese Crested X Poodle	5.87	<0.1
656	Chow Chow X Jack Russell Terrier	5.87	<0.1

657	Chow Chow X Siberian Husky	5.87	<0.1
658	Dachshund Smooth Haired X French Bulldog	5.87	<0.1
659	Dachshund Smooth Haired X Shih Tzu	5.87	<0.1
660	Dachshund Smooth Haired X Spaniel Cocker	5.87	<0.1
661	Dachshund Smooth Haired X Staffordshire Bull Terrier	5.87	<0.1
662	Dachshund Smooth Haired X Welsh Corgi Pembroke	5.87	<0.1
663	Dalmatian X Pointer	5.87	<0.1
664	Dalmatian X Weimaraner	5.87	<0.1
665	Dogue De Bordeaux X German Shepherd Dog	5.87	<0.1
666	Dogue De Bordeaux X Rhodesian Ridgeback	5.87	<0.1
667	English Setter X Retriever Golden	5.87	<0.1
668	Estrela Mountain Dog Cross/Type	5.87	<0.1
669	Finnish Lapphund X Samoyed	5.87	<0.1
670	French Bulldog X Jack Russell Terrier	5.87	<0.1
671	Frisian Water Dog Cross/Type	5.87	<0.1
672	German Shepherd Dog X Pyrenean Mountain Dog	5.87	<0.1
673	German Shepherd Dog X Saluki	5.87	<0.1
674	German Shepherd Dog X St Bernard	5.87	<0.1
675	German Spitz Giant Cross/Type	5.87	<0.1
676	German Wirehaired Pointer X Weimaraner	5.87	<0.1
677	Griffon Bruxellois X Shih Tzu	5.87	<0.1
678	Hanoverian Scent Hound Cross/Type	5.87	<0.1
679	Havanese X Poodle	5.87	<0.1
680	Hovawart Cross/Type	5.87	<0.1
681	Hungarian Pumi Cross/Type	5.87	<0.1
682	Irish Red And White Setter X Retriever Labrador	5.87	<0.1
683	Irish Setter X Poodle	5.87	<0.1
684	Irish Setter X Spaniel Cocker	5.87	<0.1
685	Istrian Short Haired Hound Cross/Type	5.87	<0.1
686	Italian Cane Corso Cross/Type	5.87	<0.1
687	Jack Russell Terrier X Manchester Terrier	5.87	<0.1
688	Jack Russell Terrier X Miniature Pinscher	5.87	<0.1
689	Jack Russell Terrier X Papillon	5.87	<0.1
690	Jack Russell Terrier X Pekingese	5.87	<0.1
691	Jack Russell Terrier X Shetland Sheepdog	5.87	<0.1
692	Japanese Akita Inu X Rottweiler	5.87	<0.1
693	Japanese Akita Inu X Siberian Husky	5.87	<0.1
694	Japanese Akita Inu X Staffordshire Bull Terrier	5.87	<0.1
695	Kai Cross/Type	5.87	<0.1
696	King Charles Spaniel X Maltese	5.87	<0.1
697	Kishu Cross/Type	5.87	<0.1
698	Komondor Cross/Type	5.87	<0.1
699	Korean Jindo Cross/Type	5.87	<0.1
700	Lagotto Romagnolo Cross/Type	5.87	<0.1

701	Maltese X Pug	5.87	<0.1
702	Maltese X Spaniel Cocker	5.87	<0.1
703	Mastiff X Poodle	5.87	<0.1
704	Norfolk Terrier X Shih Tzu	5.87	<0.1
705	Norfolk Terrier X West Highland White Terrier	5.87	<0.1
706	Old English Sheepdog X St Bernard	5.87	<0.1
707	Peruvian Hairless Dog Cross/Type	5.87	<0.1
708	Picardy Spaniel Cross/Type	5.87	<0.1
709	Pointer X Staffordshire Bull Terrier	5.87	<0.1
710	Pomeranian X Spaniel Cocker	5.87	<0.1
711	Pomeranian X Staffordshire Bull Terrier	5.87	<0.1
712	Pont Audemer Spaniel Cross/Type	5.87	<0.1
713	Poodle X Spaniel Clumber	5.87	<0.1
714	Poodle X Tibetan Terrier	5.87	<0.1
715	Pug X Retriever Labrador	5.87	<0.1
716	Pug X West Highland White Terrier	5.87	<0.1
717	Rafeiro do Alentejo Cross/Type	5.87	<0.1
718	Retriever Flat Coated X Retriever Golden	5.87	<0.1
719	Retriever Flat Coated X Spaniel English Springer	5.87	<0.1
720	Retriever Labrador X Spanish Water Dog	5.87	<0.1
721	Shih Tzu X Staffordshire Bull Terrier	5.87	<0.1
722	Shih Tzu X Tibetan Terrier	5.87	<0.1
723	Shikoku Cross/Type	5.87	<0.1
724	Small Münsterländer Cross/Type	5.87	<0.1
725	Spaniel Cocker X West Highland White Terrier	5.87	<0.1
726	Spaniel Cocker X Whippet	5.87	<0.1
727	Welsh Terrier Cross/Type	5.87	<0.1
728	Afghan Hound X Spaniel Cocker	2.94	<0.1
729	Airedale Terrier X Australian Shepherd	2.94	<0.1
730	Airedale Terrier X German Shepherd Dog	2.94	<0.1
731	Airedale Terrier X Retriever Labrador	2.94	<0.1
732	Alaskan Malamute X American Akita	2.94	<0.1
733	Alaskan Malamute X Dalmatian	2.94	<0.1
734	Alaskan Malamute X Dobermann	2.94	<0.1
735	Alaskan Malamute X Retriever Golden	2.94	<0.1
736	Alaskan Malamute X Staffordshire Bull Terrier	2.94	<0.1
737	American Akita X Beagle	2.94	<0.1
738	American Akita X Belgian Shepherd Dog	2.94	<0.1
739	American Akita X Bullmastiff	2.94	<0.1
740	American Akita X Jack Russell Terrier	2.94	<0.1
741	American Akita X Rhodesian Ridgeback	2.94	<0.1
742	American Akita X Samoyed	2.94	<0.1
743	American Staffordshire Terrier X Bulldog	2.94	<0.1
744	American Staffordshire Terrier X Shar Pei	2.94	<0.1

745	Australian Cattle Dog X Chihuahua Smooth Coat	2.94	<0.1
746	Australian Cattle Dog X German Shepherd Dog	2.94	<0.1
747	Australian Cattle Dog X Yorkshire Terrier	2.94	<0.1
748	Australian Kelpie X Weimaraner	2.94	<0.1
749	Australian Kelpie X Welsh Corgi Pembroke	2.94	<0.1
750	Australian Shepherd X Border Collie	2.94	<0.1
751	Australian Shepherd X Boxer	2.94	<0.1
752	Australian Shepherd X Poodle	2.94	<0.1
753	Australian Silky Terrier X Yorkshire Terrier	2.94	<0.1
754	Australian Terrier Cross/Type	2.94	<0.1
755	Austrian Black And Tan Hound Cross/Type	2.94	<0.1
756	Basenji X Whippet	2.94	<0.1
757	Basset Artesien Normand Cross/Type	2.94	<0.1
758	Basset Bleu De Gascogne Cross/Type	2.94	<0.1
759	Basset Hound X Border Collie	2.94	<0.1
760	Basset Hound X Bulldog	2.94	<0.1
761	Basset Hound X Cavalier King Charles Spaniel	2.94	<0.1
762	Basset Hound X German Shepherd Dog	2.94	<0.1
763	Basset Hound X Jack Russell Terrier	2.94	<0.1
764	Basset Hound X Yorkshire Terrier	2.94	<0.1
765	Beagle X Bichon Frise	2.94	<0.1
766	Beagle X Boxer	2.94	<0.1
767	Beagle X Cairn Terrier	2.94	<0.1
768	Beagle X Chihuahua Smooth Coat	2.94	<0.1
769	Beagle X Dachshund Smooth Haired	2.94	<0.1
770	Beagle X Fox Terrier Wire	2.94	<0.1
771	Beagle X German Shepherd Dog	2.94	<0.1
772	Beagle X Parson Russell Terrier	2.94	<0.1
773	Beagle X Pomeranian	2.94	<0.1
774	Beagle X Spaniel Welsh Springer	2.94	<0.1
775	Beagle X Weimaraner	2.94	<0.1
776	Beagle X Whippet	2.94	<0.1
777	Beagle X Yorkshire Terrier	2.94	<0.1
778	Bearded Collie X Old English Sheepdog	2.94	<0.1
779	Bearded Collie X Poodle	2.94	<0.1
780	Bearded Collie X Spaniel Cocker	2.94	<0.1
781	Bearded Collie X Spaniel English Springer	2.94	<0.1
782	Bedlington Terrier X Border Collie	2.94	<0.1
783	Bedlington Terrier X Italian Greyhound	2.94	<0.1
784	Bedlington Terrier X Lakeland Terrier	2.94	<0.1
785	Bedlington Terrier X Shih Tzu	2.94	<0.1
786	Bedlington Terrier X West Highland White Terrier	2.94	<0.1
787	Belgian Shepherd Dog Malinois X Border Collie	2.94	<0.1
788	Belgian Shepherd Dog Malinois X Boxer	2.94	<0.1

789	Belgian Shepherd Dog Malinois X Dutch Shepherd Dog	2.94	<0.1
790	Belgian Shepherd Dog Malinois X Mastiff	2.94	<0.1
791	Belgian Shepherd Dog Malinois X Staffordshire Bull Terrier	2.94	<0.1
792	Bernese Mountain Dog X Border Collie	2.94	<0.1
793	Bernese Mountain Dog X German Shepherd Dog	2.94	<0.1
794	Bernese Mountain Dog X Retriever Labrador	2.94	<0.1
795	Bichon Frise X Chinese Crested	2.94	<0.1
796	Bichon Frise X Fox Terrier Wire	2.94	<0.1
797	Bichon Frise X French Bulldog	2.94	<0.1
798	Bichon Frise X King Charles Spaniel	2.94	<0.1
799	Bichon Frise X Löwchen	2.94	<0.1
800	Bichon Frise X Norfolk Terrier	2.94	<0.1
801	Bichon Frise X Papillon	2.94	<0.1
802	Bichon Frise X Samoyed	2.94	<0.1
803	Bichon Frise X Scottish Terrier	2.94	<0.1
804	Bichon Frise X Soft Coated Wheaten Terrier	2.94	<0.1
805	Bohemian Wire Haired Pointing Griffon Cross/Type	2.94	<0.1
806	Bohemian Wire Haired Pointing Griffon X Fox Terrier Wire	2.94	<0.1
807	Bolognese X Poodle	2.94	<0.1
808	Border Collie X Bull Terrier	2.94	<0.1
809	Border Collie X Cavalier King Charles Spaniel	2.94	<0.1
810	Border Collie X Chihuahua Smooth Coat	2.94	<0.1
811	Border Collie X English Setter	2.94	<0.1
812	Border Collie X German Hunting Terrier	2.94	<0.1
813	Border Collie X German Wirehaired Pointer	2.94	<0.1
814	Border Collie X Great Dane	2.94	<0.1
815	Border Collie X Hungarian Puli	2.94	<0.1
816	Border Collie X Irish Wolfhound	2.94	<0.1
817	Border Collie X Newfoundland	2.94	<0.1
818	Border Collie X Portuguese Water Dog	2.94	<0.1
819	Border Collie X Rough Collie	2.94	<0.1
820	Border Collie X Small Münsterländer	2.94	<0.1
821	Border Collie X Tibetan Terrier	2.94	<0.1
822	Border Collie X Wheaten Terrier	2.94	<0.1
823	Border Terrier X French Bulldog	2.94	<0.1
824	Border Terrier X Manchester Terrier	2.94	<0.1
825	Border Terrier X Parson Russell Terrier	2.94	<0.1
826	Border Terrier X Spaniel Cocker	2.94	<0.1
827	Border Terrier X Staffordshire Bull Terrier	2.94	<0.1
828	Border Terrier X Whippet	2.94	<0.1
829	Boston Terrier X Cavalier King Charles Spaniel	2.94	<0.1
830	Boston Terrier X Poodle	2.94	<0.1
831	Bourbonnais Pointing Dog Cross/Type	2.94	<0.1
832	Boxer X Dalmatian	2.94	<0.1

833	Boxer X Hungarian Vizsla	2.94	<0.1
834	Boxer X Poodle	2.94	<0.1
835	Briquet Griffon Vendeen Cross/Type	2.94	<0.1
836	Brittany X Pointer	2.94	<0.1
837	Bull Terrier X Chihuahua Smooth Coat	2.94	<0.1
838	Bull Terrier X German Shepherd Dog	2.94	<0.1
839	Bull Terrier X Pomeranian	2.94	<0.1
840	Bull Terrier X Retriever Labrador	2.94	<0.1
841	Bull Terrier X Rhodesian Ridgeback	2.94	<0.1
842	Bull Terrier X Spaniel English Springer	2.94	<0.1
843	Bulldog X Great Dane	2.94	<0.1
844	Bulldog X Greyhound	2.94	<0.1
845	Bulldog X Neapolitan Mastiff	2.94	<0.1
846	Bulldog X Pomeranian	2.94	<0.1
847	Bulldog X Rhodesian Ridgeback	2.94	<0.1
848	Bullmastiff X French Bulldog	2.94	<0.1
849	Bullmastiff X Greyhound	2.94	<0.1
850	Bullmastiff X Shar Pei	2.94	<0.1
851	Bullmastiff X Spaniel English Springer	2.94	<0.1
852	Burgos Pointing Dog Cross/Type	2.94	<0.1
853	Cairn Terrier X Dachshund Smooth Haired	2.94	<0.1
854	Cairn Terrier X Kerry Blue Terrier	2.94	<0.1
855	Cairn Terrier X Lakeland Terrier	2.94	<0.1
856	Cairn Terrier X Lhasa Apso	2.94	<0.1
857	Cairn Terrier X Miniature Schnauzer	2.94	<0.1
858	Cairn Terrier X Spaniel American Cocker	2.94	<0.1
859	Canarian Warren Hound X Retriever Golden	2.94	<0.1
860	Canarian Warren Hound X Retriever Labrador	2.94	<0.1
861	Cavalier King Charles Spaniel X Dachshund Miniature Smooth Haired	2.94	<0.1
862	Cavalier King Charles Spaniel X Miniature Schnauzer	2.94	<0.1
863	Cavalier King Charles Spaniel X Norfolk Terrier	2.94	<0.1
864	Cavalier King Charles Spaniel X Pekingese	2.94	<0.1
865	Cavalier King Charles Spaniel X Schnauzer	2.94	<0.1
866	Cavalier King Charles Spaniel X Welsh Corgi Pembroke	2.94	<0.1
867	Chihuahua Smooth Coat X Cavalier King Charles Spaniel	2.94	<0.1
868	Chihuahua Smooth Coat X Greyhound	2.94	<0.1
869	Chihuahua Smooth Coat X Italian Greyhound	2.94	<0.1
870	Chihuahua Smooth Coat X Japanese Shiba Inu	2.94	<0.1
871	Chihuahua Smooth Coat X Norwich Terrier	2.94	<0.1
872	Chihuahua Smooth Coat X Retriever Labrador	2.94	<0.1
873	Chihuahua Smooth Coat X Spaniel Cocker	2.94	<0.1
874	Chihuahua Smooth Coat X Tibetan Spaniel	2.94	<0.1
875	Chihuahua Smooth Coat X Whippet	2.94	<0.1
876	Chinese Crested X German Shepherd Dog	2.94	<0.1

877	Chinese Crested X Jack Russell Terrier	2.94	<0.1
878	Chinese Crested X Pug	2.94	<0.1
879	Chinese Crested X Shih Tzu	2.94	<0.1
880	Chinese Crested X Tibetan Spaniel	2.94	<0.1
881	Chinese Crested X Yorkshire Terrier	2.94	<0.1
882	Chow Chow X German Shepherd Dog	2.94	<0.1
883	Chow Chow X Retriever Labrador	2.94	<0.1
884	Chow Chow X Staffordshire Bull Terrier	2.94	<0.1
885	Collie Rough X Hungarian Puli	2.94	<0.1
886	Coton De Tulear X Lhasa Apso	2.94	<0.1
887	Coton De Tulear X Shih Tzu	2.94	<0.1
888	Dachshund Long Haired X Dachshund Smooth Haired	2.94	<0.1
889	Dachshund Miniature Smooth Haired X Miniature Pinscher	2.94	<0.1
890	Dachshund Smooth Haired X Miniature Pinscher	2.94	<0.1
891	Dachshund Smooth Haired X Pomeranian	2.94	<0.1
892	Dachshund Smooth Haired X Pug	2.94	<0.1
893	Dachshund Smooth Haired X Schnauzer	2.94	<0.1
894	Dachshund Smooth Haired X Shetland Sheepdog	2.94	<0.1
895	Dalmatian X Dobermann	2.94	<0.1
896	Dalmatian X Newfoundland	2.94	<0.1
897	Dalmatian X Rottweiler	2.94	<0.1
898	Dalmatian X Spaniel English Springer	2.94	<0.1
899	Deerhound X Saluki	2.94	<0.1
900	Deerhound X Whippet	2.94	<0.1
901	Dobermann X Giant Schnauzer	2.94	<0.1
902	Dobermann X Jack Russell Terrier	2.94	<0.1
903	Dobermann X Pointer	2.94	<0.1
904	Dobermann X Retriever Golden	2.94	<0.1
905	Dobermann X Siberian Husky	2.94	<0.1
906	Dobermann X Weimaraner	2.94	<0.1
907	Dogue De Bordeaux X Great Dane	2.94	<0.1
908	Dogue De Bordeaux X Greyhound	2.94	<0.1
909	Dutch Schapendoes Cross/Type	2.94	<0.1
910	English Setter X Gordon Setter	2.94	<0.1
911	English Setter X Pointer	2.94	<0.1
912	English Setter X Poodle	2.94	<0.1
913	Entlebucher Mountain Dog Cross/Type	2.94	<0.1
914	Fila Brasileiro Cross/Type	2.94	<0.1
915	Finnish Hound Cross/Type	2.94	<0.1
916	Finnish Lapphund Cross/Type	2.94	<0.1
917	Fox Terrier Wire X Miniature Schnauzer	2.94	<0.1
918	Fox Terrier Wire X Scottish Terrier	2.94	<0.1
919	Fox Terrier Wire X Welsh Corgi Pembroke	2.94	<0.1
920	Foxhound X Jack Russell Terrier	2.94	<0.1

921	Foxhound X Papillon	2.94	<0.1
922	Foxhound X St Bernard	2.94	<0.1
923	French Bulldog X Mastiff	2.94	<0.1
924	French Bulldog X Neapolitan Mastiff	2.94	<0.1
925	French Bulldog X Pomeranian	2.94	<0.1
926	French Bulldog X Shar Pei	2.94	<0.1
927	French Bulldog X Spaniel Cocker	2.94	<0.1
928	French White And Black Hound Cross/Type	2.94	<0.1
929	German Hunting Terrier Cross/Type	2.94	<0.1
930	German Shepherd Dog X Giant Schnauzer	2.94	<0.1
931	German Shepherd Dog X Large Münsterländer	2.94	<0.1
932	German Shepherd Dog X Leonberger	2.94	<0.1
933	German Shepherd Dog X Pug	2.94	<0.1
934	German Shepherd Dog X Retriever Flat Coated	2.94	<0.1
935	German Shepherd Dog X Shar Pei	2.94	<0.1
936	German Shepherd Dog X Spaniel Cocker	2.94	<0.1
937	German Shepherd Dog X Weimaraner	2.94	<0.1
938	German Shepherd Dog X Welsh Corgi Pembroke	2.94	<0.1
939	German Shepherd Dog X Whippet	2.94	<0.1
940	German Shorthaired Pointer X Retriever Labrador	2.94	<0.1
941	German Shorthaired Pointer X Spaniel English Springer	2.94	<0.1
942	German Shorthaired Pointer X Staffordshire Bull Terrier	2.94	<0.1
943	German Spitz Mittel X Poodle	2.94	<0.1
944	Glen Of Imaal Terrier X Shih Tzu	2.94	<0.1
945	Great Dane X Siberian Husky	2.94	<0.1
946	Great Dane X Staffordshire Bull Terrier	2.94	<0.1
947	Great Dane X Yorkshire Terrier	2.94	<0.1
948	Great Swiss Mountain Dog X Italian Short Haired Segugio	2.94	<0.1
949	Greenland Dog Cross/Type	2.94	<0.1
950	Greyhound X Irish Wolfhound	2.94	<0.1
951	Greyhound X Siberian Husky	2.94	<0.1
952	Griffon Bruxellois X Pomeranian	2.94	<0.1
953	Griffon Bruxellois X Poodle	2.94	<0.1
954	Griffon Bruxellois X Spaniel Cocker	2.94	<0.1
955	Havanese X Maltese	2.94	<0.1
956	Havanese X Yorkshire Terrier	2.94	<0.1
957	Hungarian Puli X Spaniel Cocker	2.94	<0.1
958	Hungarian Pumi X Mudi	2.94	<0.1
959	Hungarian Vizsla X Pointer	2.94	<0.1
960	Hungarian Vizsla X Rhodesian Ridgeback	2.94	<0.1
961	Hungarian Vizsla X Spaniel Cocker	2.94	<0.1
962	Hungarian Vizsla X Weimaraner	2.94	<0.1
963	Icelandic Sheepdog Cross/Type	2.94	<0.1
964	Irish Setter X Siberian Husky	2.94	<0.1

965	Irish Terrier X Parson Russell Terrier	2.94	<0.1
966	Irish Terrier X Weimaraner	2.94	<0.1
967	Irish Wolfhound X Retriever Labrador	2.94	<0.1
968	Irish Wolfhound X Rottweiler	2.94	<0.1
969	Italian Cane Corso X Neapolitan Mastiff	2.94	<0.1
970	Italian Greyhound X Jack Russell Terrier	2.94	<0.1
971	Italian Greyhound X Pointer	2.94	<0.1
972	Italian Greyhound X Poodle	2.94	<0.1
973	Italian Volpino Cross/Type	2.94	<0.1
974	Jack Russell Terrier X Saluki	2.94	<0.1
975	Jack Russell Terrier X Shar Pei	2.94	<0.1
976	Jack Russell Terrier X Stabijhoun	2.94	<0.1
977	Japanese Akita Inu X Rhodesian Ridgeback	2.94	<0.1
978	Japanese Akita Inu X St Bernard	2.94	<0.1
979	Japanese Shiba Inu X Pomeranian	2.94	<0.1
980	Japanese Spitz X Shetland Sheepdog	2.94	<0.1
981	Japanese Spitz X Welsh Corgi Pembroke	2.94	<0.1
982	Karst Shepherd Dog Cross/Type	2.94	<0.1
983	King Charles Spaniel X Miniature Schnauzer	2.94	<0.1
984	King Charles Spaniel X Papillon	2.94	<0.1
985	King Charles Spaniel X Pomeranian	2.94	<0.1
986	King Charles Spaniel X Retriever Labrador	2.94	<0.1
987	King Charles Spaniel X Staffordshire Bull Terrier	2.94	<0.1
988	King Charles Spaniel X Welsh Corgi Pembroke	2.94	<0.1
989	King Charles Spaniel X West Highland White Terrier	2.94	<0.1
990	King Charles Spaniel X Yorkshire Terrier	2.94	<0.1
991	Lakeland Terrier X Pug	2.94	<0.1
992	Lakeland Terrier X Shih Tzu	2.94	<0.1
993	Landseer Cross/Type	2.94	<0.1
994	Lapponian Herder Cross/Type	2.94	<0.1
995	Leonberger X Newfoundland	2.94	<0.1
996	Lhasa Apso X Retriever Labrador	2.94	<0.1
997	Lhasa Apso X Staffordshire Bull Terrier	2.94	<0.1
998	Lhasa Apso X Tibetan Terrier	2.94	<0.1
999	Maremma Sheepdog X Norwegian Elkhound	2.94	<0.1
1000	Mastiff X Neapolitan Mastiff	2.94	<0.1
1001	Mastiff X Newfoundland	2.94	<0.1
1002	Mastiff X Rafeiro do Alentejo	2.94	<0.1
1003	Miniature Pinscher X Pomeranian	2.94	<0.1
1004	Miniature Pinscher X Poodle	2.94	<0.1
1005	Miniature Schnauzer X Scottish Terrier	2.94	<0.1
1006	Miniature Schnauzer X Shih Tzu	2.94	<0.1
1007	Neapolitan Mastiff X Rhodesian Ridgeback	2.94	<0.1
1008	Newfoundland X Old English Sheepdog	2.94	<0.1

1009	Newfoundland X Poodle	2.94	<0.1
1010	Newfoundland X Retriever Labrador	2.94	<0.1
1011	Newfoundland X Rottweiler	2.94	<0.1
1012	Newfoundland X St Bernard	2.94	<0.1
1013	Newfoundland X Staffordshire Bull Terrier	2.94	<0.1
1014	Norfolk Terrier X Staffordshire Bull Terrier	2.94	<0.1
1015	Norwich Terrier X Yorkshire Terrier	2.94	<0.1
1016	Old English Sheepdog X Poodle	2.94	<0.1
1017	Papillon X West Highland White Terrier	2.94	<0.1
1018	Parson Russell Terrier X Pomeranian	2.94	<0.1
1019	Parson Russell Terrier X Pug	2.94	<0.1
1020	Pekingese X West Highland White Terrier	2.94	<0.1
1021	Pointer X Retriever Golden	2.94	<0.1
1022	Pomeranian X Retriever Labrador	2.94	<0.1
1023	Poodle X Retriever Nova Scotia Duck Tolling	2.94	<0.1
1024	Poodle X Russian Black Terrier	2.94	<0.1
1025	Poodle X Samoyed	2.94	<0.1
1026	Poodle X Scottish Terrier	2.94	<0.1
1027	Poodle X Siberian Husky	2.94	<0.1
1028	Poodle X Staffordshire Bull Terrier	2.94	<0.1
1029	Poodle X Weimaraner	2.94	<0.1
1030	Porcelaine Cross/Type	2.94	<0.1
1031	Portuguese Podengo X Retriever Labrador	2.94	<0.1
1032	Presa Canario X Retriever Labrador	2.94	<0.1
1033	Presa Canario X Rhodesian Ridgeback	2.94	<0.1
1034	Pug X Spaniel English Springer	2.94	<0.1
1035	Pyrenean Sheepdog Long Haired Cross/Type	2.94	<0.1
1036	Retriever Golden X Rhodesian Ridgeback	2.94	<0.1
1037	Retriever Golden X Siberian Husky	2.94	<0.1
1038	Retriever Golden X Spaniel Irish Water	2.94	<0.1
1039	Retriever Labrador X Retriever Nova Scotia Duck Tolling	2.94	<0.1
1040	Retriever Labrador X Shih Tzu	2.94	<0.1
1041	Retriever Labrador X Welsh Corgi Pembroke	2.94	<0.1
1042	Retriever Labrador X West Highland White Terrier	2.94	<0.1
1043	Rhodesian Ridgeback X Shar Pei	2.94	<0.1
1044	Rhodesian Ridgeback X Weimaraner	2.94	<0.1
1045	Rottweiler X Spaniel Cocker	2.94	<0.1
1046	Russian European Laika Cross/Type	2.94	<0.1
1047	Saint Germain Pointer Cross/Type	2.94	<0.1
1048	Samoyed X Siberian Husky	2.94	<0.1
1049	Samoyed X Spaniel Cocker	2.94	<0.1
1050	Schnauzer X Yorkshire Terrier	2.94	<0.1
1051	Serbian Hound Cross/Type	2.94	<0.1
1052	Shar Pei X Shih Tzu	2.94	<0.1

1053	Shar Pei X Siberian Husky	2.94	<0.1
1054	Shar Pei X Spaniel Irish Water	2.94	<0.1
1055	Shetland Sheepdog X Spaniel Cocker	2.94	<0.1
1056	Shetland Sheepdog X Whippet	2.94	<0.1
1057	Shih Tzu X Welsh Corgi Pembroke	2.94	<0.1
1058	Soft Coated Wheaten Terrier X Staffordshire Bull Terrier	2.94	<0.1
1059	Soft Coated Wheaten Terrier X West Highland White Terrier	2.94	<0.1
1060	Spaniel American Cocker X Spaniel Cocker	2.94	<0.1
1061	Spaniel Clumber X Spaniel Cocker	2.94	<0.1
1062	Spaniel Cocker X Spaniel Field	2.94	<0.1
1063	Spaniel Cocker X Spanish Water Dog	2.94	<0.1
1064	Spaniel English Springer X West Highland White Terrier	2.94	<0.1
1065	Spanish Hound X Spanish Mastiff	2.94	<0.1
1066	Staffordshire Bull Terrier X Weimaraner	2.94	<0.1
1067	Tibetan Spaniel X Welsh Corgi Pembroke	2.94	<0.1
1068	Tosa Cross/Type	2.94	<0.1
1069	West Highland White Terrier X Whippet	2.94	<0.1
1070	West Siberian Laika Cross/Type	2.94	<0.1

Supplementary Table 6. UK 2019 dog population estimate for all purebreds per country, with associated proportional breed demographics (%), ranked by popularity. Purebreds of an equal estimated population size are secondary ranked alphabetically.

	England			Northern Ireland			Scotland			Wales		
	**Breed**	**Population Estimate**	**Proportion of Country Population (%)**	**Breed**	**Population Estimate**	**Proportion of Country Population (%)**	**Breed**	**Population Estimate**	**Proportion of Country Population (%)**	**Breed**	**Population Estimate**	**Proportion of Country Population (%)**
1	Retriever Labrador	917737.71	10.2	Miniature Schnauzer	20301.56	8.5	Retriever Labrador	136080.11	13.3	Retriever Labrador	51637.71	7.9
2	Spaniel Cocker	630477.89	7	Retriever Labrador	20015.49	8.4	Spaniel Cocker	84025.91	8.2	French Bulldog	39071.87	6
3	Staffordshire Bull Terrier	429722.86	4.8	Yorkshire Terrier	15115.75	6.4	Lhasa Apso	48777.52	4.8	Spaniel Cocker	35124.42	5.4
4	Spaniel English Springer	382201.01	4.2	Spaniel Cocker	13886.81	5.8	Spaniel English Springer	46712.12	4.6	Spaniel English Springer	33680.45	5.2
5	German Shepherd Dog	319796.35	3.6	Spaniel English Springer	13136.17	5.5	German Shepherd Dog	42537.85	4.2	Staffordshire Bull Terrier	31776.36	4.9
6	French Bulldog	310404.15	3.5	Shih Tzu	12136.08	5.1	Staffordshire Bull Terrier	33791.75	3.3	Cavalier King Charles Spaniel	22585.82	3.5
7	Pug	249401.54	2.8	Staffordshire Bull Terrier	9836.11	4.1	West Highland White Terrier	31661.13	3.1	Bulldog	21477.87	3.3
8	Retriever Golden	245873.81	2.7	Cavalier King Charles Spaniel	8197.52	3.4	Retriever Golden	31449.93	3.1	Shih Tzu	18832.11	2.9
9	Border Terrier	238142.95	2.6	Lhasa Apso	8000.7	3.4	Border Terrier	30381.51	3	Pug	18226.68	2.8
10	Bulldog	227418.04	2.5	German Shepherd Dog	7559.02	3.2	Border Collie	26076.79	2.5	German Shepherd Dog	17993.58	2.8
11	Jack Russell Terrier	223543.57	2.5	West Highland White Terrier	6648.18	2.8	French Bulldog	23806.41	2.3	Dachshund Miniature Smooth Haired	17024.89	2.6
12	Shih Tzu	214305.14	2.4	Retriever Golden	6558.93	2.8	Yorkshire Terrier	22269.01	2.2	Retriever Golden	16931.04	2.6
13	Cavalier King Charles Spaniel	213096.06	2.4	Boxer	6536.04	2.7	Pug	22011.22	2.1	Yorkshire Terrier	16216.63	2.5
14	Boxer	186059.17	2.1	Bichon Frise	5858.64	2.5	Boxer	21085.68	2.1	Bichon Frise	15907.85	2.4
15	Border Collie	179618.8	2	Rottweiler	4849.4	2	Cavalier King Charles Spaniel	20685.02	2	Boxer	15414.42	2.4
16	West Highland White Terrier	176739.32	2	Pug	4821.93	2	Miniature Schnauzer	18961.27	1.9	West Highland White Terrier	14533.51	2.2
17	Miniature Schnauzer	170832.64	1.9	Jack Russell Terrier	3988.91	1.7	Jack Russell Terrier	17762.41	1.7	Jack Russell Terrier	13380.16	2
18	Yorkshire Terrier	168462.72	1.9	Chihuahua Long Coat	3558.67	1.5	Shih Tzu	17135.03	1.7	Whippet	12163.23	1.9
19	Chihuahua Smooth Coat	157544.85	1.8	Siberian Husky	3467.12	1.5	Bulldog	14678.29	1.4	Miniature Schnauzer	11203.61	1.7
20	Poodle	138112.18	1.5	American Akita	2840.07	1.2	Chihuahua Smooth Coat	13081.88	1.3	Poodle	10897.86	1.7

21	Dachshund Miniature Smooth Haired	128668.71	1.4	French Bulldog	2634.1	1.1	Beagle	12122.17	1.2	Border Terrier	10695.04	1.6
22	Whippet	116602.06	1.3	Bulldog	2613.5	1.1	Poodle	12066.26	1.2	Border Collie	9771.75	1.5
23	Lhasa Apso	115402.03	1.3	Poodle	2414.4	1	Bichon Frise	11873.7	1.2	Chihuahua Smooth Coat	9293.46	1.4
24	Beagle	103627.85	1.2	Chihuahua Smooth Coat	2286.24	1	Siberian Husky	10861.19	1.1	Beagle	9105.77	1.4
25	Rottweiler	97826.69	1.1	Border Collie	2238.18	0.9	Whippet	10851.87	1.1	Chihuahua Long Coat	8015.98	1.2
26	Bichon Frise	91564.22	1	Beagle	2066.54	0.9	Hungarian Vizsla	10153.05	1	Bull Terrier	6069.5	0.9
27	Bull Terrier	86453.53	1	Pomeranian	2041.37	0.9	Rottweiler	9923.22	1	Shar Pei	6066.48	0.9
28	Hungarian Vizsla	85618.33	1	Cairn Terrier	1915.5	0.8	Chihuahua Long Coat	9258.57	0.9	Rottweiler	5382.33	0.8
29	Siberian Husky	84930.88	0.9	Dachshund Miniature Smooth Haired	1759.88	0.7	Dachshund Miniature Smooth Haired	8211.89	0.8	Alaskan Malamute	5230.97	0.8
30	Chihuahua Long Coat	83160.98	0.9	Dobermann	1688.94	0.7	Cairn Terrier	7311.19	0.7	Hungarian Vizsla	5067.51	0.8
31	Dogue De Bordeaux	81131.78	0.9	Whippet	1489.83	0.6	Weimaraner	7140.37	0.7	Lhasa Apso	5013.02	0.8
32	Shar Pei	79687.52	0.9	Kerry Blue Terrier	1389.14	0.6	German Shorthaired Pointer	7053.41	0.7	Weimaraner	4955.5	0.8
33	Dobermann	64180.58	0.7	Shetland Sheepdog	1370.83	0.6	Dalmatian	6922.96	0.7	Dogue De Bordeaux	4785.98	0.7
34	Weimaraner	62271.99	0.7	Border Terrier	1363.96	0.6	Maltese	6631.01	0.6	Dobermann	4586.18	0.7
35	Dalmatian	59754.34	0.7	Weimaraner	1263.27	0.5	Retriever Flat Coated	6090.59	0.6	Siberian Husky	4486.29	0.7
36	Pomeranian	58536.21	0.7	Dogue De Bordeaux	1219.79	0.5	Pomeranian	5829.7	0.6	Basset Hound	4177.51	0.6
37	German Shorthaired Pointer	56347.21	0.6	Bull Terrier	1141.98	0.5	Dogue De Bordeaux	5634.03	0.5	Welsh Corgi Pembroke	4095.78	0.6
38	Boston Terrier	52542.09	0.6	Newfoundland	1109.94	0.5	Shar Pei	5584.33	0.5	Boston Terrier	4062.48	0.6
39	Retriever Flat Coated	47144.96	0.5	Irish Setter	1077.9	0.5	Alaskan Malamute	5432.15	0.5	Pomeranian	4062.48	0.6
40	Tibetan Terrier	46358	0.5	Maltese	952.03	0.4	Shetland Sheepdog	5133.99	0.5	Cairn Terrier	3796.09	0.6
41	Maltese	45577.08	0.5	Shar Pei	952.03	0.4	Scottish Terrier	5012.86	0.5	Dalmatian	3650.78	0.6
42	Cairn Terrier	44989.12	0.5	Dachshund Miniature Long Haired	919.99	0.4	Dobermann	4845.14	0.5	Scottish Terrier	3266.33	0.5
43	Rhodesian Ridgeback	44187.09	0.5	Tibetan Terrier	881.08	0.4	Boston Terrier	4842.03	0.5	German Shorthaired Pointer	3048.37	0.5
44	Greyhound	40930.73	0.5	Basset Hound	862.78	0.4	Collie Rough	4814.08	0.5	Bullmastiff	2972.7	0.5
45	Great Dane	39778.94	0.4	Fox Terrier Wire	784.97	0.3	American Akita	4807.87	0.5	Old English Sheepdog	2785.01	0.4

46	Shetland Sheepdog	38503.53	0.4	Bullmastiff	768.95	0.3	Tibetan Terrier	4537.66	0.4	Welsh Terrier	2715.38	0.4
47	American Akita	38295.48	0.4	Bernese Mountain Dog	748.35	0.3	Newfoundland	4453.8	0.4	Dachshund Miniature Long Haired	2639.7	0.4
48	Basset Hound	37776.87	0.4	Collie Rough	716.31	0.3	Rhodesian Ridgeback	4320.25	0.4	Newfoundland	2542.83	0.4
49	Alaskan Malamute	36613.02	0.4	Great Dane	711.73	0.3	Bull Terrier	4273.66	0.4	Great Dane	2497.43	0.4
50	Dachshund Miniature Long Haired	36495.43	0.4	Samoyed	704.87	0.3	Great Dane	4227.07	0.4	Maltese	2494.4	0.4
51	Bullmastiff	35780.84	0.4	German Shorthaired Pointer	700.29	0.3	Papillon	4090.42	0.4	Shetland Sheepdog	2312.77	0.4
52	Newfoundland	30486.23	0.3	Boston Terrier	675.12	0.3	Bearded Collie	3702.18	0.4	Rhodesian Ridgeback	2303.69	0.4
53	Collie Rough	29210.82	0.3	Schnauzer	638.5	0.3	Greyhound	3577.95	0.3	Chow Chow	2158.38	0.3
54	Irish Setter	28839.95	0.3	Hungarian Vizsla	608.75	0.3	Irish Setter	3152.45	0.3	St Bernard	2022.16	0.3
55	Scottish Terrier	28010.79	0.3	Scottish Terrier	606.46	0.3	Bullmastiff	2844.97	0.3	Fox Terrier Wire	1904.1	0.3
56	Pointer	26527.33	0.3	Chow Chow	526.36	0.2	Parson Russell Terrier	2798.38	0.3	Tibetan Terrier	1810.26	0.3
57	Bedlington Terrier	26415.77	0.3	King Charles Spaniel	512.63	0.2	Basset Hound	2677.25	0.3	Collie Rough	1789.07	0.3
58	Airedale Terrier	26346.42	0.3	Japanese Spitz	503.48	0.2	Bernese Mountain Dog	2652.4	0.3	Papillon	1695.22	0.3
59	Fox Terrier Wire	26029.83	0.3	St Bernard	478.3	0.2	Dachshund Miniature Long Haired	2587.18	0.3	Spaniel Welsh Springer	1646.79	0.3
60	Dachshund Smooth Haired	25414.74	0.3	Alaskan Malamute	462.28	0.2	German Wirehaired Pointer	2528.17	0.2	Retriever Flat Coated	1589.27	0.2
61	Dachshund Miniature Wire Haired	25043.87	0.3	Dalmatian	459.99	0.2	Pointer	2338.71	0.2	Chinese Crested	1574.14	0.2
62	Parson Russell Terrier	24862.97	0.3	Collie Smooth	421.09	0.2	Fox Terrier Wire	2335.61	0.2	King Charles Spaniel	1562.03	0.2
63	Norfolk Terrier	24612.71	0.3	Lakeland Terrier	411.94	0.2	St Bernard	2257.96	0.2	Greyhound	1471.21	0.2
64	King Charles Spaniel	23093.07	0.3	Old English Sheepdog	393.63	0.2	Chinese Crested	2161.68	0.2	Bedlington Terrier	1453.05	0.2
65	Papillon	22119.17	0.2	Dachshund Smooth Haired	373.03	0.2	Airedale Terrier	2152.36	0.2	Irish Setter	1446.99	0.2
66	St Bernard	21392.52	0.2	Parson Russell Terrier	373.03	0.2	Samoyed	2077.82	0.2	Airedale Terrier	1437.91	0.2
67	Bernese Mountain Dog	20442.75	0.2	Pekingese	340.99	0.1	Deerhound	2037.44	0.2	Bernese Mountain Dog	1428.83	0.2
68	Chow Chow	20138.22	0.2	Japanese Akita Inu	336.41	0.1	Dachshund Miniature Wire Haired	2031.23	0.2	Dachshund Miniature Wire Haired	1380.4	0.2

69	Bearded Collie	20099.02	0.2	Rhodesian Ridgeback	336.41	0.1	Chow Chow	1959.8	0.2	Pekingese	1338.02	0.2
70	Old English Sheepdog	19821.63	0.2	Belgian Shepherd Dog Malinois	331.84	0.1	King Charles Spaniel	1851.09	0.2	Dachshund Smooth Haired	1271.42	0.2
71	Mastiff	18075.85	0.2	Soft Coated Wheaten Terrier	304.37	0.1	Gordon Setter	1844.88	0.2	American Akita	1262.34	0.2
72	Italian Spinone	17668.81	0.2	Dachshund Miniature Wire Haired	299.8	0.1	Lakeland Terrier	1829.35	0.2	German Wirehaired Pointer	1041.35	0.2
73	Lakeland Terrier	17418.55	0.2	Greyhound	265.47	0.1	Bedlington Terrier	1764.13	0.2	Mastiff	1011.08	0.2
74	Chinese Crested	17376.34	0.2	Pointer	251.74	0.1	Tibetan Spaniel	1587.09	0.2	Parson Russell Terrier	992.92	0.2
75	Dachshund Wire Haired	16269.77	0.2	Retriever Flat Coated	251.74	0.1	Old English Sheepdog	1562.25	0.2	Pointer	992.92	0.2
76	Miniature Pinscher	15494.88	0.2	Irish Wolfhound	249.45	0.1	Dachshund Wire Haired	1493.92	0.1	Samoyed	950.54	0.1
77	Soft Coated Wheaten Terrier	15111.96	0.2	Miniature Pinscher	242.58	0.1	Dachshund Smooth Haired	1472.18	0.1	Italian Greyhound	944.48	0.1
78	Schnauzer	14533.05	0.2	Bearded Collie	228.85	0.1	Italian Spinone	1472.18	0.1	Bearded Collie	896.05	0.1
79	Irish Terrier	14306.91	0.2	English Setter	212.83	0.1	Soft Coated Wheaten Terrier	1403.85	0.1	Japanese Shiba Inu	823.39	0.1
80	Welsh Terrier	14113.94	0.2	Dachshund Wire Haired	210.54	0.1	Mastiff	1369.68	0.1	Norfolk Terrier	796.15	0.1
81	Leonberger	13836.54	0.2	Irish Terrier	210.54	0.1	Miniature Pinscher	1323.1	0.1	Welsh Corgi Cardigan	793.12	0.1
82	Pekingese	13800.36	0.2	Airedale Terrier	201.39	0.1	Welsh Corgi Pembroke	1236.13	0.1	Collie Smooth	741.66	0.1
83	Japanese Akita Inu	13721.97	0.2	Mastiff	201.39	0.1	Japanese Akita Inu	1099.47	0.1	Miniature Pinscher	723.5	0.1
84	Spaniel American Cocker	13601.37	0.1	Papillon	189.95	0.1	Japanese Spitz	1096.37	0.1	Lakeland Terrier	714.42	0.1
85	Spaniel Welsh Springer	13456.64	0.1	Bedlington Terrier	178.51	0.1	Spaniel Welsh Springer	1090.16	0.1	Irish Wolfhound	696.25	0.1
86	German Wirehaired Pointer	13121.95	0.1	Chinese Crested	176.22	0.1	Collie Smooth	1087.05	0.1	Saluki	693.23	0.1
87	English Setter	13100.85	0.1	Welsh Corgi Pembroke	167.06	0.1	Norfolk Terrier	1052.89	0.1	Soft Coated Wheaten Terrier	684.14	0.1
88	Samoyed	12196.3	0.1	Japanese Chin	155.62	0.1	Pekingese	950.39	0.1	Italian Spinone	675.06	0.1
89	Havanese	11759.1	0.1	Brittany	153.33	0.1	Schnauzer	947.29	0.1	Griffon Bruxellois	650.84	0.1
90	Welsh Corgi Pembroke	11566.13	0.1	Griffon Bruxellois	151.04	0.1	Leonberger	934.86	0.1	Dachshund Wire Haired	641.76	0.1
91	Japanese Shiba Inu	10803.3	0.1	Dachshund Long Haired	148.75	0.1	Welsh Terrier	934.86	0.1	Deerhound	641.76	0.1
92	Brittany	10366.1	0.1	Spaniel Irish Water	148.75	0.1	Dandie Dinmont Terrier	928.65	0.1	Brittany	617.55	0.1

93	Belgian Shepherd Dog Malinois	10290.72	0.1	Havanese	139.6	0.1	English Setter	878.96	0.1	Belgian Shepherd Dog Malinois	599.38	0.1
94	Italian Greyhound	10142.98	0.1	Tibetan Spaniel	132.73	0.1	Japanese Shiba Inu	832.37	0.1	Sealyham Terrier	584.25	0.1
95	Coton De Tulear	10055.54	0.1	Spaniel Welsh Springer	125.87	0.1	Brittany	791.99	0.1	Japanese Akita Inu	547.92	0.1
96	Irish Wolfhound	9729.9	0.1	Italian Spinone	119	0.1	Irish Wolfhound	791.99	0.1	Schnauzer	529.76	0.1
97	Saluki	9346.98	0.1	Norwegian Elkhound	119	0.1	Japanese Chin	770.25	0.1	Borzoi	526.73	0.1
98	Gordon Setter	9175.12	0.1	Gordon Setter	116.72	<0.1	Skye Terrier	736.09	0.1	Leonberger	526.73	0.1
99	Giant Schnauzer	9120.84	0.1	Basset Fauve De Bretagne	109.85	<0.1	Spaniel American Cocker	736.09	0.1	English Setter	511.59	0.1
100	Spaniel Clumber	8825.36	0.1	Eurasier	100.7	<0.1	Eurasier	708.14	0.1	Irish Terrier	496.46	0.1
101	Retriever Nova Scotia Duck Tolling	8303.74	0.1	Norfolk Terrier	100.7	<0.1	Coton De Tulear	664.65	0.1	Spaniel American Cocker	493.44	0.1
102	Dachshund Long Haired	8086.64	0.1	Irish Red And White Setter	96.12	<0.1	Spaniel Clumber	642.91	0.1	Japanese Chin	478.3	0.1
103	Japanese Chin	8068.55	0.1	Afghan Hound	93.83	<0.1	Italian Greyhound	621.17	0.1	Tibetan Spaniel	441.97	0.1
104	Manchester Terrier	7474.57	0.1	Coton De Tulear	93.83	<0.1	Kerry Blue Terrier	618.07	0.1	Japanese Spitz	423.81	0.1
105	Deerhound	7420.3	0.1	Giant Schnauzer	89.25	<0.1	Australian Shepherd	614.96	0.1	Neapolitan Mastiff	411.7	0.1
106	Spanish Water Dog	7314.77	0.1	Russian Black Terrier	89.25	<0.1	Saluki	590.11	0.1	English Toy Terrier	408.67	0.1
107	Bull Terrier Miniature	7254.46	0.1	Deerhound	86.96	<0.1	Dachshund Long Haired	587.01	0.1	Kerry Blue Terrier	399.59	0.1
108	Griffon Bruxellois	7037.37	0.1	Bull Terrier Miniature	84.68	<0.1	Spaniel Irish Water	568.37	0.1	Spaniel Clumber	393.53	0.1
109	Tibetan Spaniel	6805.2	0.1	Borzoi	80.1	<0.1	Havanese	565.27	0.1	Australian Shepherd	357.21	0.1
110	Collie Smooth	6545.9	0.1	Spaniel American Cocker	77.81	<0.1	Irish Terrier	565.27	0.1	Retriever Chesapeake Bay	354.18	0.1
111	Japanese Spitz	6419.26	0.1	German Longhaired Pointer	73.23	<0.1	Spanish Water Dog	540.42	0.1	Basset Griffon Vendeen Petit	351.15	0.1
112	Australian Shepherd	6147.9	0.1	Neapolitan Mastiff	73.23	<0.1	Bull Terrier Miniature	528	0.1	Briard	345.1	0.1
113	Portuguese Water Dog	6000.16	0.1	Spanish Water Dog	70.94	<0.1	Retriever Nova Scotia Duck Tolling	503.15	<0.1	Fox Terrier Smooth	336.02	0.1
114	Neapolitan Mastiff	5918.75	0.1	Leonberger	68.66	<0.1	Afghan Hound	500.04	<0.1	Dachshund Long Haired	332.99	0.1
115	Norwich Terrier	5831.31	0.1	Norwegian Buhund	68.66	<0.1	Giant Schnauzer	496.94	<0.1	Coton De Tulear	305.75	<0.1
116	Kerry Blue Terrier	5819.25	0.1	Fox Terrier Smooth	66.37	<0.1	Griffon Bruxellois	493.83	<0.1	Bull Terrier Miniature	296.66	<0.1

117	Bolognese	5804.17	0.1	Basset Griffon Vendeen Petit	64.08	<0.1	Large Münsterländer	487.62	<0.1	German Spitz Klein	275.47	<0.1
118	Lancashire Heeler	5764.98	0.1	English Toy Terrier	64.08	<0.1	Belgian Shepherd Dog Malinois	481.41	<0.1	Tibetan Mastiff	275.47	<0.1
119	Afghan Hound	5581.05	0.1	Saluki	64.08	<0.1	Fox Terrier Smooth	472.09	<0.1	Retriever Nova Scotia Duck Tolling	272.45	<0.1
120	Affenpinscher	5439.34	0.1	Australian Shepherd	61.79	<0.1	Bolognese	465.88	<0.1	Havanese	266.39	<0.1
121	Pyrenean Mountain Dog	4821.23	0.1	Bouvier Des Flandres	59.5	<0.1	Briard	456.56	<0.1	Gordon Setter	263.37	<0.1
122	Fox Terrier Smooth	4763.94	0.1	Keeshond	59.5	<0.1	Portuguese Water Dog	431.71	<0.1	Pyrenean Mountain Dog	248.23	<0.1
123	Briard	4498.61	0.1	Bavarian Mountain Hound	52.64	<0.1	Welsh Corgi Cardigan	413.08	<0.1	Afghan Hound	242.17	<0.1
124	German Spitz Mittel	4420.22	<0.1	Tibetan Mastiff	52.64	<0.1	Bavarian Mountain Hound	391.34	<0.1	German Spitz Mittel	239.15	<0.1
125	English Toy Terrier	4323.73	<0.1	German Wirehaired Pointer	45.77	<0.1	Pyrenean Mountain Dog	344.75	<0.1	Bracco Italiano	236.12	<0.1
126	Basset Griffon Vendeen Petit	4257.4	<0.1	Glen Of Imaal Terrier	45.77	<0.1	Finnish Lapphund	338.54	<0.1	Giant Schnauzer	233.09	<0.1
127	Dandie Dinmont Terrier	4221.22	<0.1	Pyrenean Mountain Dog	45.77	<0.1	Manchester Terrier	332.33	<0.1	Australian Kelpie	217.96	<0.1
128	Keeshond	4212.17	<0.1	Spaniel Clumber	45.77	<0.1	Spaniel Field	332.33	<0.1	Manchester Terrier	211.9	<0.1
129	Welsh Corgi Cardigan	4046.34	<0.1	Large Münsterländer	43.48	<0.1	Retriever Chesapeake Bay	323.01	<0.1	Lancashire Heeler	205.85	<0.1
130	Spaniel Field	3958.9	<0.1	Spaniel Field	41.19	<0.1	Russian Black Terrier	323.01	<0.1	Polish Lowland Sheepdog	202.82	<0.1
131	Löwchen	3898.6	<0.1	American Staffordshire Terrier	38.91	<0.1	Irish Red And White Setter	316.8	<0.1	Cesky Terrier	190.71	<0.1
132	Basset Griffon Vendeen Grand	3805.13	<0.1	Japanese Shiba Inu	38.91	<0.1	German Spitz Klein	304.37	<0.1	Norwich Terrier	190.71	<0.1
133	Basset Fauve De Bretagne	3802.11	<0.1	Löwchen	38.91	<0.1	Borzoi	279.53	<0.1	Spanish Water Dog	190.71	<0.1
134	Spaniel Irish Water	3681.5	<0.1	German Spitz Klein	36.62	<0.1	Neapolitan Mastiff	279.53	<0.1	Spaniel Field	187.69	<0.1
135	Large Münsterländer	3669.44	<0.1	Australian Cattle Dog	32.04	<0.1	Norwich Terrier	279.53	<0.1	Bolognese	178.6	<0.1
136	Eurasier	3572.96	<0.1	Australian Silky Terrier	32.04	<0.1	Keeshond	276.42	<0.1	Belgian Shepherd Dog Tervueren	169.52	<0.1
137	Sealyham Terrier	3313.66	<0.1	Portuguese Water Dog	32.04	<0.1	Norwegian Elkhound	276.42	<0.1	Keeshond	163.47	<0.1
138	Italian Cane Corso	3295.56	<0.1	Welsh Terrier	32.04	<0.1	Tibetan Mastiff	257.79	<0.1	Retriever Curly Coated	163.47	<0.1

139	Irish Red And White Setter	3289.53	<0.1	Retriever Chesapeake Bay	29.75	<0.1	German Pinscher	251.57	<0.1	Large Münsterländer	160.44	<0.1
140	German Spitz Klein	3274.46	<0.1	Bloodhound	27.46	<0.1	German Spitz Mittel	251.57	<0.1	Estrela Mountain Dog	154.39	<0.1
141	Bracco Italiano	3259.38	<0.1	Foxhound	27.46	<0.1	Lancashire Heeler	248.47	<0.1	Australian Silky Terrier	148.33	<0.1
142	Retriever Curly Coated	3162.9	<0.1	Italian Greyhound	27.46	<0.1	Basset Griffon Vendeen Petit	245.36	<0.1	Skye Terrier	142.28	<0.1
143	Belgian Shepherd Dog Tervueren	3159.88	<0.1	Manchester Terrier	27.46	<0.1	Bracco Italiano	242.26	<0.1	Xoloitzcuintle	142.28	<0.1
144	Finnish Lapphund	3042.29	<0.1	Briard	25.17	<0.1	Löwchen	229.83	<0.1	Bouvier Des Flandres	139.25	<0.1
145	Borzoi	2930.73	<0.1	Caucasian Shepherd Dog	25.17	<0.1	Basset Griffon Vendeen Grand	223.62	<0.1	Norwegian Elkhound	136.22	<0.1
146	Retriever Chesapeake Bay	2852.34	<0.1	Dandie Dinmont Terrier	22.89	<0.1	Affenpinscher	211.2	<0.1	Spaniel Irish Water	136.22	<0.1
147	Russian Black Terrier	2825.2	<0.1	Retriever Nova Scotia Duck Tolling	22.89	<0.1	Basenji	204.99	<0.1	Australian Cattle Dog	130.17	<0.1
148	Norwegian Elkhound	2755.85	<0.1	Swedish Vallhund	22.89	<0.1	Bouvier Des Flandres	204.99	<0.1	Otterhound	127.14	<0.1
149	Australian Cattle Dog	2668.41	<0.1	Catalan Sheepdog	20.6	<0.1	Hungarian Puli	201.88	<0.1	Portuguese Water Dog	127.14	<0.1
150	Tibetan Mastiff	2626.2	<0.1	German Spitz Mittel	20.6	<0.1	English Toy Terrier	198.77	<0.1	Anatolian Shepherd Dog	124.11	<0.1
151	Spaniel Sussex	2596.05	<0.1	Lagotto Romagnolo	20.6	<0.1	Australian Kelpie	192.56	<0.1	Hungarian Puli	121.09	<0.1
152	Bouvier Des Flandres	2590.02	<0.1	Lancashire Heeler	20.6	<0.1	Australian Cattle Dog	189.46	<0.1	Affenpinscher	115.03	<0.1
153	Presa Canario	2590.02	<0.1	Affenpinscher	18.31	<0.1	Basset Fauve De Bretagne	186.35	<0.1	Löwchen	115.03	<0.1
154	German Pinscher	2553.84	<0.1	Basenji	18.31	<0.1	Bloodhound	177.03	<0.1	Dandie Dinmont Terrier	108.98	<0.1
155	Hungarian Puli	2415.14	<0.1	Cirneco dell'Etna	18.31	<0.1	Glen Of Imaal Terrier	173.93	<0.1	Korthals Griffon	108.98	<0.1
156	Portuguese Podengo	2403.08	<0.1	Hovawart	18.31	<0.1	Belgian Shepherd Dog Tervueren	170.82	<0.1	Basset Griffon Vendeen Grand	105.95	<0.1
157	Lagotto Romagnolo	2378.96	<0.1	Norwich Terrier	18.31	<0.1	Retriever Curly Coated	170.82	<0.1	Belgian Shepherd Dog Groenendael	102.92	<0.1
158	Bloodhound	2339.76	<0.1	Portuguese Podengo	18.31	<0.1	Italian Cane Corso	167.72	<0.1	Irish Red And White Setter	102.92	<0.1
159	Glen Of Imaal Terrier	2327.7	<0.1	Schipperke	18.31	<0.1	Anatolian Shepherd Dog	164.61	<0.1	Italian Cane Corso	96.87	<0.1
160	Belgian Shepherd Dog Groenendael	2237.24	<0.1	Welsh Corgi Cardigan	18.31	<0.1	American Staffordshire Terrier	161.5	<0.1	Maremma Sheepdog	93.84	<0.1

161	Australian Silky Terrier	2128.7	<0.1	Basset Bleu De Gascogne	16.02	<0.1	Lagotto Romagnolo	158.4	<0.1	Bavarian Mountain Hound	87.79	<0.1
162	American Staffordshire Terrier	2080.46	<0.1	German Pinscher	16.02	<0.1	Australian Silky Terrier	145.98	<0.1	Bloodhound	87.79	<0.1
163	Polish Lowland Sheepdog	2077.44	<0.1	Russian Toy	16.02	<0.1	Hovawart	142.87	<0.1	Finnish Spitz	84.76	<0.1
164	Basenji	1848.29	<0.1	Sealyham Terrier	16.02	<0.1	Schipperke	133.55	<0.1	Presa Canario	84.76	<0.1
165	Bavarian Mountain Hound	1842.26	<0.1	Skye Terrier	16.02	<0.1	Belgian Shepherd Dog Groenendael	130.45	<0.1	Russian Black Terrier	81.73	<0.1
166	Korthals Griffon	1778.94	<0.1	Australian Kelpie	13.73	<0.1	Norwegian Buhund	127.34	<0.1	Schipperke	81.73	<0.1
167	Slovakian Rough Haired Pointer	1709.59	<0.1	Harrier	13.73	<0.1	Sealyham Terrier	121.13	<0.1	Basset Fauve De Bretagne	78.71	<0.1
168	Schipperke	1688.49	<0.1	Korthals Griffon	13.73	<0.1	Foxhound	118.02	<0.1	American Staffordshire Terrier	75.68	<0.1
169	Swedish Vallhund	1670.4	<0.1	Bolognese	11.44	<0.1	Polish Lowland Sheepdog	118.02	<0.1	Norwegian Buhund	75.68	<0.1
170	Catalan Sheepdog	1552.8	<0.1	Bracco Italiano	11.44	<0.1	Canadian Eskimo Dog	114.92	<0.1	Glen Of Imaal Terrier	72.65	<0.1
171	Australian Kelpie	1531.7	<0.1	Cesky Terrier	11.44	<0.1	German Longhaired Pointer	114.92	<0.1	Eurasier	69.63	<0.1
172	Foxhound	1402.05	<0.1	Kooikerhondje	11.44	<0.1	Catalan Sheepdog	105.6	<0.1	Finnish Lapphund	66.6	<0.1
173	Russian Toy	1233.2	<0.1	Presa Canario	11.44	<0.1	Great Swiss Mountain Dog	102.49	<0.1	Slovakian Rough Haired Pointer	66.6	<0.1
174	Skye Terrier	1178.93	<0.1	Belgian Shepherd Dog Tervueren	9.15	<0.1	Korthals Griffon	99.39	<0.1	Catalan Sheepdog	63.57	<0.1
175	Maremma Sheepdog	1148.77	<0.1	Dutch Shepherd Dog	9.15	<0.1	Presa Canario	99.39	<0.1	Foxhound	63.57	<0.1
176	Kooikerhondje	1130.68	<0.1	Finnish Lapphund	9.15	<0.1	Portuguese Podengo	83.86	<0.1	Greenland Dog	60.54	<0.1
177	Great Swiss Mountain Dog	1115.61	<0.1	Anatolian Shepherd Dog	6.87	<0.1	Finnish Spitz	80.75	<0.1	Pyrenean Sheepdog Long Haired	60.54	<0.1
178	Caucasian Shepherd Dog	1082.44	<0.1	Beauceron	6.87	<0.1	Slovakian Rough Haired Pointer	80.75	<0.1	Swedish Vallhund	60.54	<0.1
179	Otterhound	1055.3	<0.1	Belgian Shepherd Dog Groenendael	6.87	<0.1	Spaniel Sussex	80.75	<0.1	German Pinscher	57.52	<0.1
180	Cesky Terrier	1046.26	<0.1	Belgian Shepherd Dog Laekenois	6.87	<0.1	Pharaoh Hound	77.65	<0.1	Turkish Kangal Dog	57.52	<0.1

181	Hovawart	1028.17	<0.1	Canaan Dog	6.87	<0.1	Maremma Sheepdog	59.01	<0.1	Spaniel Sussex	54.49	<0.1
182	Xoloitzcuintle	970.88	<0.1	Canadian Eskimo Dog	6.87	<0.1	Xoloitzcuintle	55.91	<0.1	Basenji	51.46	<0.1
183	German Longhaired Pointer	964.85	<0.1	German Hunting Terrier	6.87	<0.1	Beauceron	52.8	<0.1	Portuguese Podengo	51.46	<0.1
184	Anatolian Shepherd Dog	949.77	<0.1	Great Swiss Mountain Dog	6.87	<0.1	Ibizan Hound	52.8	<0.1	Pharaoh Hound	48.43	<0.1
185	Beauceron	940.73	<0.1	Hungarian Puli	6.87	<0.1	Swedish Vallhund	52.8	<0.1	Caucasian Shepherd Dog	39.35	<0.1
186	Pharaoh Hound	934.7	<0.1	Italian Cane Corso	6.87	<0.1	Caucasian Shepherd Dog	49.69	<0.1	White Swiss Shepherd Dog	39.35	<0.1
187	Norwegian Buhund	880.43	<0.1	Retriever Curly Coated	6.87	<0.1	Hungarian Pumi	49.69	<0.1	Great Swiss Mountain Dog	36.33	<0.1
188	Finnish Spitz	802.03	<0.1	Romanian Mioritic Shepherd Dog	6.87	<0.1	Cesky Terrier	43.48	<0.1	Russian Toy	30.27	<0.1
189	Harrier	756.8	<0.1	White Swiss Shepherd Dog	6.87	<0.1	Estrela Mountain Dog	43.48	<0.1	Canaan Dog	27.24	<0.1
190	Ibizan Hound	753.79	<0.1	Basset Griffon Vendeen Grand	4.58	<0.1	Pyrenean Sheepdog Long Haired	43.48	<0.1	Hovawart	27.24	<0.1
191	Estrela Mountain Dog	747.76	<0.1	Black And Tan Coonhound	4.58	<0.1	Canarian Warren Hound	40.38	<0.1	Lagotto Romagnolo	27.24	<0.1
192	Canadian Eskimo Dog	708.56	<0.1	Entlebucher Mountain Dog	4.58	<0.1	Russian Toy	40.38	<0.1	Belgian Shepherd Dog Laekenois	24.22	<0.1
193	Canaan Dog	699.52	<0.1	Finnish Spitz	4.58	<0.1	Komondor	37.27	<0.1	Cirneco dell’Etna	24.22	<0.1
194	Dutch Shepherd Dog	569.86	<0.1	Hungarian Kuvasz	4.58	<0.1	Basset Bleu De Gascogne	34.16	<0.1	Canadian Eskimo Dog	21.19	<0.1
195	Hamiltonstovare	494.49	<0.1	Ibizan Hound	4.58	<0.1	Harrier	31.06	<0.1	Dutch Shepherd Dog	18.16	<0.1
196	Cirneco dell’Etna	485.44	<0.1	Otterhound	4.58	<0.1	Kooikerhondje	31.06	<0.1	Harrier	18.16	<0.1
197	Spanish Mastiff	476.39	<0.1	Pharaoh Hound	4.58	<0.1	Spanish Mastiff	31.06	<0.1	Kooikerhondje	18.16	<0.1
198	Entlebucher Mountain Dog	452.27	<0.1	Polish Lowland Sheepdog	4.58	<0.1	Spanish Greyhound	27.95	<0.1	Czechoslovakia n Wolfdog	15.14	<0.1
199	Portuguese Pointer	446.24	<0.1	Spaniel Sussex	4.58	<0.1	Dutch Shepherd Dog	24.85	<0.1	Dutch Schapendoes	15.14	<0.1
200	Pyrenean Sheepdog Long Haired	440.21	<0.1	Alpine Dachsbracke	2.29	<0.1	Belgian Shepherd Dog Laekenois	21.74	<0.1	Entlebucher Mountain Dog	15.14	<0.1
201	Hungarian Pumi	428.15	<0.1	Australian Terrier	2.29	<0.1	Canaan Dog	21.74	<0.1	Hamiltonstovare	15.14	<0.1
202	Canarian Warren Hound	419.11	<0.1	Auvergne Pointer	2.29	<0.1	Entlebucher Mountain Dog	21.74	<0.1	Korean Jindo	15.14	<0.1
203	Korean Jindo	413.08	<0.1	Azawakh	2.29	<0.1	Hamiltonstovare	21.74	<0.1	Spanish Mastiff	15.14	<0.1
204	Spanish Greyhound	355.79	<0.1	Blue Picardy Spaniel	2.29	<0.1	Portuguese Pointer	21.74	<0.1	Basset Bleu De Gascogne	12.11	<0.1

205	Komondor	343.73	<0.1	Central Asia Shepherd Dog	2.29	<0.1	Romanian Carpathian Shepherd Dog	21.74	<0.1	Beauceron	12.11	<0.1
206	Sloughi	298.5	<0.1	Dutch Schapendoes	2.29	<0.1	White Swiss Shepherd Dog	21.74	<0.1	German Longhaired Pointer	12.11	<0.1
207	Basset Bleu De Gascogne	286.44	<0.1	East Siberia Laika	2.29	<0.1	Cirneco dell'Etna	18.64	<0.1	Barbet	9.08	<0.1
208	Belgian Shepherd Dog Laekenois	277.39	<0.1	Estrela Mountain Dog	2.29	<0.1	Polish Hound	15.53	<0.1	Canarian Warren Hound	9.08	<0.1
209	Czechoslovakia n Wolfdog	277.39	<0.1	Fila Brasileiro	2.29	<0.1	Pyrenean Sheepdog Smooth Faced	15.53	<0.1	Hungarian Pumi	9.08	<0.1
210	Romanian Carpathian Shepherd Dog	262.32	<0.1	Hungarian Pumi	2.29	<0.1	Turkish Kangal Dog	15.53	<0.1	Ibizan Hound	9.08	<0.1
211	Auvergne Pointer	259.3	<0.1	Karelian Bear Dog	2.29	<0.1	Auvergne Pointer	12.42	<0.1	Romanian Bucovina Shepherd	9.08	<0.1
212	Bergamasco	259.3	<0.1	Komondor	2.29	<0.1	Bergamasco	12.42	<0.1	Spaniel American Water	9.08	<0.1
213	Central Asia Shepherd Dog	259.3	<0.1	Korean Jindo	2.29	<0.1	Otterhound	12.42	<0.1	Spanish Greyhound	9.08	<0.1
214	White Swiss Shepherd Dog	226.14	<0.1	Maremma Sheepdog	2.29	<0.1	Saarloos Wolfhound	12.42	<0.1	Thai Ridgeback Dog	9.08	<0.1
215	Pyrenean Sheepdog Smooth Faced	208.05	<0.1	Norrbottenspitz	2.29	<0.1	Small Münsterländer	12.42	<0.1	Yugoslavian Shepherd Dog	9.08	<0.1
216	Small Münsterländer	208.05	<0.1	Picardy Sheepdog	2.29	<0.1	Stabijhoun	12.42	<0.1	Alpine Dachsbracke	6.05	<0.1
217	Stabijhoun	183.92	<0.1	Polish Hound	2.29	<0.1	Barbet	9.32	<0.1	Australian Terrier	6.05	<0.1
218	Hellenic Hound	174.88	<0.1	Shikoku	2.29	<0.1	Central Asia Shepherd Dog	9.32	<0.1	Auvergne Pointer	6.05	<0.1
219	Thai Ridgeback Dog	165.83	<0.1	Slovakian Rough Haired Pointer	2.29	<0.1	French Spaniel	9.32	<0.1	Bergamasco	6.05	<0.1
220	Barbet	147.74	<0.1	Spaniel American Water	2.29	<0.1	German Hunting Terrier	9.32	<0.1	Bohemian Wire Haired Pointing Griffon	6.05	<0.1
221	Picardy Sheepdog	144.73	<0.1	Spanish Mastiff	2.29	<0.1	Hellenic Hound	9.32	<0.1	Hellenic Hound	6.05	<0.1
222	Greenland Dog	141.71	<0.1	Tatra Shepherd Dog	2.29	<0.1	Romanian Mioritic Shepherd Dog	9.32	<0.1	Jamthund	6.05	<0.1
223	Dutch Schapendoes	129.65	<0.1	Xoloitzcuintle	2.29	<0.1	Swiss Hound	9.32	<0.1	Komondor	6.05	<0.1
224	Romanian Bucovina Shepherd	123.62	<0.1	NA	NA	NA	Atlas Mountain Dog	6.21	<0.1	Portuguese Sheepdog	6.05	<0.1

225	Spanish Hound	123.62	<0.1	NA	NA	NA	Austrian Pinscher	6.21	<0.1	Posavatz Hound	6.05	<0.1
226	German Hunting Terrier	117.59	<0.1	NA	NA	NA	Cimarron Uruguayo	6.21	<0.1	Romanian Mioritic Shepherd Dog	6.05	<0.1
227	Turkish Kangal Dog	105.53	<0.1	NA	NA	NA	German Roughhaired Pointer	6.21	<0.1	Sloughi	6.05	<0.1
228	Griffon Fauve De Bretagne	99.5	<0.1	NA	NA	NA	German Spitz Giant	6.21	<0.1	South Russian Shepherd Dog	6.05	<0.1
229	Swiss Hound	87.44	<0.1	NA	NA	NA	Hungarian Hound Transylvanian Scent Hound	6.21	<0.1	Spanish Hound	6.05	<0.1
230	Black And Tan Coonhound	84.42	<0.1	NA	NA	NA	Peruvian Hairless Dog	6.21	<0.1	Westphalian Dachsbracke	6.05	<0.1
231	Azawakh	75.38	<0.1	NA	NA	NA	Slovakian Hound	6.21	<0.1	Ariegeois	3.03	<0.1
232	Yugoslavian Shepherd Dog	75.38	<0.1	NA	NA	NA	Spanish Hound	6.21	<0.1	Castro Laboreiro Dog	3.03	<0.1
233	Australian Terrier	72.36	<0.1	NA	NA	NA	Alpine Dachsbracke	3.11	<0.1	Central Asia Shepherd Dog	3.03	<0.1
234	Fila Brasileiro	72.36	<0.1	NA	NA	NA	Ariegeois	3.11	<0.1	Gascon Saintongeois	3.03	<0.1
235	Hungarian Kuvasz	69.35	<0.1	NA	NA	NA	Austrian Black And Tan Hound	3.11	<0.1	German Roughhaired Pointer	3.03	<0.1
236	Saarloos Wolfhound	69.35	<0.1	NA	NA	NA	Billy	3.11	<0.1	German Spaniel	3.03	<0.1
237	Spaniel American Water	69.35	<0.1	NA	NA	NA	Blue Picardy Spaniel	3.11	<0.1	German Spitz Giant	3.03	<0.1
238	Portuguese Sheepdog	66.33	<0.1	NA	NA	NA	Bourbonnais Pointing Dog	3.11	<0.1	Hokkaido	3.03	<0.1
239	Polish Hound	63.32	<0.1	NA	NA	NA	Czechoslovakia n Wolfdog	3.11	<0.1	Karst Shepherd Dog	3.03	<0.1
240	Tatra Shepherd Dog	63.32	<0.1	NA	NA	NA	Dutch Schapendoes	3.11	<0.1	Lapponian Herder	3.03	<0.1
241	Alpine Dachsbracke	60.3	<0.1	NA	NA	NA	French Pointing Dog Pyrenean	3.11	<0.1	Picardy Sheepdog	3.03	<0.1
242	German Spitz Giant	60.3	<0.1	NA	NA	NA	German Spaniel	3.11	<0.1	Polish Hound	3.03	<0.1
243	Bohemian Wire Haired Pointing Griffon	57.29	<0.1	NA	NA	NA	Griffon Fauve De Bretagne	3.11	<0.1	Portuguese Pointer	3.03	<0.1
244	Swedish Lapphund	57.29	<0.1	NA	NA	NA	Hanoverian Scent Hound	3.11	<0.1	Pyrenean Mastiff	3.03	<0.1
245	Italian Short Haired Segugio	54.27	<0.1	NA	NA	NA	Hungarian Kuvasz	3.11	<0.1	Romanian Carpathian Shepherd Dog	3.03	<0.1
246	Mudi	54.27	<0.1	NA	NA	NA	Italian Short Haired Segugio	3.11	<0.1	Saint Miguel Cattle Dog	3.03	<0.1

247	Romanian Mioritic Shepherd Dog	54.27	<0.1	NA	NA	NA	Kishu	3.11	<0.1	Serbian Tricolour Hound	3.03	<0.1
248	Austrian Pinscher	48.24	<0.1	NA	NA	NA	Korean Jindo	3.11	<0.1	Shikoku	3.03	<0.1
249	Croatian Shepherd Dog	48.24	<0.1	NA	NA	NA	Majorca Mastiff	3.11	<0.1	NA	NA	NA
250	Bouvier Des Ardennes	45.23	<0.1	NA	NA	NA	Norwegian Hound	3.11	<0.1	NA	NA	NA
251	Italian Volpino	42.21	<0.1	NA	NA	NA	Norwegian Lundehund	3.11	<0.1	NA	NA	NA
252	German Roughhaired Pointer	39.2	<0.1	NA	NA	NA	Portuguese Sheepdog	3.11	<0.1	NA	NA	NA
253	Hungarian Greyhound	39.2	<0.1	NA	NA	NA	Posavatz Hound	3.11	<0.1	NA	NA	NA
254	Castro Laboreiro Dog	36.18	<0.1	NA	NA	NA	Pudelpointer	3.11	<0.1	NA	NA	NA
255	French Spaniel	36.18	<0.1	NA	NA	NA	Pyrenean Mastiff	3.11	<0.1	NA	NA	NA
256	Norwegian Lundehund	36.18	<0.1	NA	NA	NA	Romanian Bucovina Shepherd	3.11	<0.1	NA	NA	NA
257	Hungarian Hound Transylvanian Scent Hound	33.17	<0.1	NA	NA	NA	Shikoku	3.11	<0.1	NA	NA	NA
258	Jamthund	33.17	<0.1	NA	NA	NA	Spaniel American Water	3.11	<0.1	NA	NA	NA
259	Kromfohrlander	30.15	<0.1	NA	NA	NA	Thai Ridgeback Dog	3.11	<0.1	NA	NA	NA
260	Serbian Hound	30.15	<0.1	NA	NA	NA	Tyrolean Hound	3.11	<0.1	NA	NA	NA
261	Pyrenean Mastiff	27.14	<0.1	NA	NA	NA	Westphalian Dachsbracke	3.11	<0.1	NA	NA	NA
262	Shikoku	27.14	<0.1	NA	NA	NA	Yugoslavian Shepherd Dog	3.11	<0.1	NA	NA	NA
263	Blue Picardy Spaniel	24.12	<0.1	NA	NA	NA	NA	NA	NA	NA	NA	NA
264	Hanoverian Scent Hound	24.12	<0.1	NA	NA	NA	NA	NA	NA	NA	NA	NA
265	Landseer	24.12	<0.1	NA	NA	NA	NA	NA	NA	NA	NA	NA
266	Bosnian Broken Haired Hound	21.11	<0.1	NA	NA	NA	NA	NA	NA	NA	NA	NA
267	Bourbonnais Pointing Dog	21.11	<0.1	NA	NA	NA	NA	NA	NA	NA	NA	NA
268	Hokkaido	21.11	<0.1	NA	NA	NA	NA	NA	NA	NA	NA	NA
269	Frisian Water Dog	18.09	<0.1	NA	NA	NA	NA	NA	NA	NA	NA	NA
270	Griffon Bleu De Gascogne	18.09	<0.1	NA	NA	NA	NA	NA	NA	NA	NA	NA
271	Petit Brabancon	18.09	<0.1	NA	NA	NA	NA	NA	NA	NA	NA	NA

272	South Russian Shepherd Dog	18.09	<0.1	NA	NA	NA	NA	NA	NA	NA	NA	NA
273	Tosa	18.09	<0.1	NA	NA	NA	NA	NA	NA	NA	NA	NA
274	Austrian Black And Tan Hound	15.08	<0.1	NA	NA	NA	NA	NA	NA	NA	NA	NA
275	German Hound	15.08	<0.1	NA	NA	NA	NA	NA	NA	NA	NA	NA
276	Grand Bleu Gascogne	15.08	<0.1	NA	NA	NA	NA	NA	NA	NA	NA	NA
277	Griffon Nivernais	15.08	<0.1	NA	NA	NA	NA	NA	NA	NA	NA	NA
278	Istrian Short Haired Hound	15.08	<0.1	NA	NA	NA	NA	NA	NA	NA	NA	NA
279	Majorca Shepherd Dog	15.08	<0.1	NA	NA	NA	NA	NA	NA	NA	NA	NA
280	Norrbottenspitz	15.08	<0.1	NA	NA	NA	NA	NA	NA	NA	NA	NA
281	Polish Greyhound	15.08	<0.1	NA	NA	NA	NA	NA	NA	NA	NA	NA
282	Rafeiro do Alentejo	15.08	<0.1	NA	NA	NA	NA	NA	NA	NA	NA	NA
283	Ariegeois	12.06	<0.1	NA	NA	NA	NA	NA	NA	NA	NA	NA
284	Artois Hound	12.06	<0.1	NA	NA	NA	NA	NA	NA	NA	NA	NA
285	Atlas Mountain Dog	12.06	<0.1	NA	NA	NA	NA	NA	NA	NA	NA	NA
286	Basset Artesien Normand	12.06	<0.1	NA	NA	NA	NA	NA	NA	NA	NA	NA
287	Brazilian Terrier	12.06	<0.1	NA	NA	NA	NA	NA	NA	NA	NA	NA
288	Briquet Griffon Vendeen	12.06	<0.1	NA	NA	NA	NA	NA	NA	NA	NA	NA
289	Burgos Pointing Dog	12.06	<0.1	NA	NA	NA	NA	NA	NA	NA	NA	NA
290	Danish Swedish Farmdog	12.06	<0.1	NA	NA	NA	NA	NA	NA	NA	NA	NA
291	French Pointing Dog Pyrenean	12.06	<0.1	NA	NA	NA	NA	NA	NA	NA	NA	NA
292	French White And Black Hound	12.06	<0.1	NA	NA	NA	NA	NA	NA	NA	NA	NA
293	German Spaniel	12.06	<0.1	NA	NA	NA	NA	NA	NA	NA	NA	NA
294	Lapponian Herder	12.06	<0.1	NA	NA	NA	NA	NA	NA	NA	NA	NA
295	Majorca Mastiff	12.06	<0.1	NA	NA	NA	NA	NA	NA	NA	NA	NA
296	Peruvian Hairless Dog	12.06	<0.1	NA	NA	NA	NA	NA	NA	NA	NA	NA
297	Slovakian Hound	12.06	<0.1	NA	NA	NA	NA	NA	NA	NA	NA	NA
298	West Siberian Laika	12.06	<0.1	NA	NA	NA	NA	NA	NA	NA	NA	NA
299	Drentsche Partridge Dog	9.05	<0.1	NA	NA	NA	NA	NA	NA	NA	NA	NA
300	East Siberia Laika	9.05	<0.1	NA	NA	NA	NA	NA	NA	NA	NA	NA

301	Icelandic Sheepdog	9.05	<0.1	NA	NA	NA	NA	NA	NA	NA	NA	NA
302	Karst Shepherd Dog	9.05	<0.1	NA	NA	NA	NA	NA	NA	NA	NA	NA
303	Norwegian Hound	9.05	<0.1	NA	NA	NA	NA	NA	NA	NA	NA	NA
304	Picardy Spaniel	9.05	<0.1	NA	NA	NA	NA	NA	NA	NA	NA	NA
305	Posavatz Hound	9.05	<0.1	NA	NA	NA	NA	NA	NA	NA	NA	NA
306	Pudelpointer	9.05	<0.1	NA	NA	NA	NA	NA	NA	NA	NA	NA
307	Ariege Pointing Dog	6.03	<0.1	NA	NA	NA	NA	NA	NA	NA	NA	NA
308	Billy	6.03	<0.1	NA	NA	NA	NA	NA	NA	NA	NA	NA
309	Dutch Smoushond	6.03	<0.1	NA	NA	NA	NA	NA	NA	NA	NA	NA
310	Great Anglo French Hound	6.03	<0.1	NA	NA	NA	NA	NA	NA	NA	NA	NA
311	Kai	6.03	<0.1	NA	NA	NA	NA	NA	NA	NA	NA	NA
312	Kishu	6.03	<0.1	NA	NA	NA	NA	NA	NA	NA	NA	NA
313	Old Danish Pointing Dog	6.03	<0.1	NA	NA	NA	NA	NA	NA	NA	NA	NA
314	Taiwan Dog	6.03	<0.1	NA	NA	NA	NA	NA	NA	NA	NA	NA
315	Drever	3.02	<0.1	NA	NA	NA	NA	NA	NA	NA	NA	NA
316	Finnish Hound	3.02	<0.1	NA	NA	NA	NA	NA	NA	NA	NA	NA
317	French Tricolour Hound	3.02	<0.1	NA	NA	NA	NA	NA	NA	NA	NA	NA
318	Japanese Terrier	3.02	<0.1	NA	NA	NA	NA	NA	NA	NA	NA	NA
319	Karelian Bear Dog	3.02	<0.1	NA	NA	NA	NA	NA	NA	NA	NA	NA
320	Petit Bleu De Gascogne	3.02	<0.1	NA	NA	NA	NA	NA	NA	NA	NA	NA
321	Pont Audemer Spaniel	3.02	<0.1	NA	NA	NA	NA	NA	NA	NA	NA	NA
322	Saint Germain Pointer	3.02	<0.1	NA	NA	NA	NA	NA	NA	NA	NA	NA
323	Schillerstovare	3.02	<0.1	NA	NA	NA	NA	NA	NA	NA	NA	NA
324	Serbian Tricolour Hound	3.02	<0.1	NA	NA	NA	NA	NA	NA	NA	NA	NA
325	Slovakian Chuvach	3.02	<0.1	NA	NA	NA	NA	NA	NA	NA	NA	NA
326	Westphalian Dachsbracke	3.02	<0.1	NA	NA	NA	NA	NA	NA	NA	NA	NA

Supplementary Table 7. UK 2019 dog population estimate for all crossbreds per country, with associated proportional breed demographics (%), ranked by popularity. Crossbreds of an equal estimated population size are secondary ranked alphabetically.

	England			Northern Ireland			Scotland			Wales		
	**Breed**	**Population Estimate**	**Proportion of Country Population (%)**	**Breed**	**Population Estimate**	**Proportion of Country Population (%)**	**Breed**	**Population Estimate**	**Proportion of Country Population (%)**	**Breed**	**Population Estimate**	**Proportion of Country Population (%)**
1	Mix Breed	579398.39	38.9	Mix Breed	12689.9	44.2	Mix Breed	63300.53	48.4	Mix Breed	33837.85	42.1
2	Border Collie Cross/Type	64165.53	4.3	Border Collie Cross/Type	1695.8	5.9	Border Collie Cross/Type	8975.93	6.9	Border Collie Cross/Type	3538.78	4.4
3	Poodle X Spaniel Cocker	62250.91	4.2	Retriever Labrador Cross/Type	1682.07	5.9	Retriever Labrador Cross/Type	5972.57	4.6	Poodle X Spaniel Cocker	2745.66	3.4
4	Staffordshire Bull Terrier Cross/Type	58469.9	3.9	Poodle X Spaniel Cocker	936.01	3.3	Staffordshire Bull Terrier Cross/Type	4220.86	3.2	Staffordshire Bull Terrier Cross/Type	2694.19	3.4
5	Retriever Labrador Cross/Type	53118	3.6	Jack Russell Terrier Cross/Type	693.42	2.4	Poodle X Spaniel Cocker	4099.73	3.1	Retriever Labrador Cross/Type	2234.06	2.8
6	Jack Russell Terrier Cross/Type	45839.42	3.1	Spaniel Cocker Cross/Type	615.61	2.1	Poodle X Retriever Labrador	3568.63	2.7	Jack Russell Terrier Cross/Type	2228.01	2.8
7	Chihuahua Smooth Coat Cross/Type	38603.04	2.6	Rottweiler Cross/Type	583.58	2	Rottweiler Cross/Type	3528.26	2.7	Chihuahua Smooth Coat Cross/Type	2212.87	2.8
8	Poodle X Retriever Labrador	38509.57	2.6	Chihuahua Smooth Coat Cross/Type	542.38	1.9	Chihuahua Smooth Coat Cross/Type	2882.24	2.2	Rottweiler Cross/Type	1552.94	1.9
9	Rottweiler Cross/Type	28514.33	1.9	Staffordshire Bull Terrier Cross/Type	455.42	1.6	Bulldog Cross/Type	2195.84	1.7	Bulldog Cross/Type	1434.88	1.8
10	Spaniel Cocker Cross/Type	23590.58	1.6	Yorkshire Terrier Cross/Type	434.82	1.5	Jack Russell Terrier Cross/Type	2143.04	1.6	Bichon Frise Cross/Type	1404.61	1.7
11	Shih Tzu Cross/Type	21896.06	1.5	Shih Tzu Cross/Type	421.09	1.5	Spaniel Cocker Cross/Type	1742.39	1.3	Poodle X Retriever Labrador	1338.01	1.7
12	Bulldog Cross/Type	21847.82	1.5	Poodle X Retriever Labrador	411.94	1.4	German Shepherd Dog Cross/Type	1652.32	1.3	Shih Tzu Cross/Type	1147.3	1.4
13	German Shepherd Dog Cross/Type	21618.67	1.5	German Shepherd Dog Cross/Type	386.76	1.3	Spaniel Cocker X Spaniel English Springer	1161.59	0.9	Bichon Frise X Cavalier King Charles Spaniel	1132.17	1.4
14	Yorkshire Terrier Cross/Type	19242.72	1.3	Siberian Husky Cross/Type	345.57	1.2	Shih Tzu Cross/Type	1155.38	0.9	Spaniel Cocker Cross/Type	1114	1.4
15	Cavalier King Charles Spaniel X Poodle	17744.2	1.2	Spaniel English Springer Cross/Type	343.28	1.2	Lhasa Apso Cross/Type	1130.53	0.9	Yorkshire Terrier Cross/Type	1083.73	1.3

16	Spaniel Cocker X Spaniel English Springer	16393.4	1.1	Bichon Frise X Cavalier King Charles Spaniel	336.41	1.2	Siberian Husky Cross/Type	1090.16	0.8	Siberian Husky Cross/Type	1017.13	1.3
17	Pug Cross/Type	14894.87	1	Bichon Frise Cross/Type	320.39	1.1	Bichon Frise Cross/Type	910.02	0.7	German Shepherd Dog Cross/Type	971.73	1.2
18	Poodle Cross/Type	14539.08	1	Pug Cross/Type	299.8	1	Bull Terrier X Staffordshire Bull Terrier	863.43	0.7	Pug Cross/Type	874.86	1.1
19	Bichon Frise Cross/Type	13308.9	0.9	Bulldog Cross/Type	286.07	1	Yorkshire Terrier Cross/Type	823.05	0.6	Spaniel Cocker X Spaniel English Springer	808.26	1
20	Bichon Frise X Cavalier King Charles Spaniel	12259.62	0.8	Pomeranian Cross/Type	274.62	1	Pug Cross/Type	819.95	0.6	Spaniel English Springer Cross/Type	735.61	0.9
21	Spaniel English Springer Cross/Type	12211.38	0.8	Lhasa Apso Cross/Type	212.83	0.7	Maltese Cross/Type	810.63	0.6	Cavalier King Charles Spaniel X Poodle	696.25	0.9
22	Siberian Husky Cross/Type	11924.94	0.8	Chihuahua Smooth Coat X Yorkshire Terrier	189.95	0.7	Cavalier King Charles Spaniel X Poodle	770.26	0.6	Poodle Cross/Type	653.87	0.8
23	Bull Terrier X Staffordshire Bull Terrier	9778.15	0.7	Beagle Cross/Type	169.35	0.6	Bullmastiff X Dogue De Bordeaux	723.67	0.6	Jack Russell Terrier X Pug	644.79	0.8
24	Pomeranian Cross/Type	9657.54	0.6	Maltese Cross/Type	162.49	0.6	Poodle Cross/Type	717.45	0.5	Chihuahua Smooth Coat X Yorkshire Terrier	575.16	0.7
25	Bullmastiff X Dogue De Bordeaux	8810.29	0.6	Cavalier King Charles Spaniel Cross/Type	160.2	0.6	Bichon Frise X Cavalier King Charles Spaniel	692.61	0.5	Pomeranian Cross/Type	514.62	0.6
26	Chihuahua Smooth Coat X Yorkshire Terrier	7718.8	0.5	Cairn Terrier Cross/Type	151.04	0.5	Poodle X Retriever Golden	639.81	0.5	Bull Terrier X Staffordshire Bull Terrier	463.16	0.6
27	Border Terrier Cross/Type	7471.56	0.5	Poodle Cross/Type	148.75	0.5	Chihuahua Smooth Coat X Yorkshire Terrier	621.17	0.5	Beagle Cross/Type	396.56	0.5
28	Maltese Cross/Type	7290.65	0.5	West Highland White Terrier Cross/Type	137.31	0.5	Spaniel English Springer Cross/Type	611.85	0.5	Whippet Cross/Type	396.56	0.5
29	Jack Russell Terrier X Poodle	6844.4	0.5	Jack Russell Terrier X Pug	135.02	0.5	Jack Russell Terrier X Pug	549.74	0.4	West Highland White Terrier Cross/Type	393.53	0.5
30	Jack Russell Terrier X Pug	6790.13	0.5	Spaniel Cocker X Spaniel English Springer	135.02	0.5	Beagle X Pug	528	0.4	Dachshund Smooth Haired Cross/Type	384.45	0.5
31	Whippet Cross/Type	6741.89	0.5	Cavalier King Charles Spaniel X Poodle	123.58	0.4	Pomeranian Cross/Type	422.4	0.3	Shar Pei Cross/Type	372.34	0.5
32	West Highland White Terrier Cross/Type	6597.16	0.4	Bullmastiff X Dogue De Bordeaux	121.29	0.4	West Highland White Terrier Cross/Type	344.75	0.3	Bullmastiff X Dogue De Bordeaux	360.23	0.4

33	Lhasa Apso Cross/Type	6380.07	0.4	Dobermann X German Pinscher	121.29	0.4	Boxer Cross/Type	341.64	0.3	Cavalier King Charles Spaniel Cross/Type	357.21	0.4
34	Mastiff Cross/Type	5945.89	0.4	Whippet Cross/Type	116.72	0.4	Jack Russell Terrier X Parson Russell Terrier	329.22	0.3	Border Terrier Cross/Type	314.83	0.4
35	Cavalier King Charles Spaniel Cross/Type	5568.99	0.4	Boxer Cross/Type	114.43	0.4	Dobermann X German Pinscher	326.12	0.2	Poodle X Retriever Golden	302.72	0.4
36	Poodle X Retriever Golden	5490.6	0.4	Bull Terrier X Staffordshire Bull Terrier	112.14	0.4	Mastiff Cross/Type	307.48	0.2	Maltese Cross/Type	278.5	0.3
37	Beagle X Pug	5219.23	0.4	Dachshund Smooth Haired Cross/Type	107.56	0.4	Whippet Cross/Type	291.95	0.2	Alaskan Malamute Cross/Type	275.47	0.3
38	Boxer Cross/Type	5207.17	0.3	Cavalier King Charles Spaniel X Pug	84.68	0.3	Border Terrier Cross/Type	267.1	0.2	Beagle X Pug	269.42	0.3
39	Beagle Cross/Type	5170.99	0.3	Border Terrier Cross/Type	75.52	0.3	German Shorthaired Pointer X Weimaraner	245.36	0.2	French Bulldog Cross/Type	248.23	0.3
40	Bullmastiff Cross/Type	5092.6	0.3	Miniature Schnauzer Cross/Type	75.52	0.3	Alaskan Malamute Cross/Type	239.15	0.2	Lhasa Apso Cross/Type	248.23	0.3
41	Dachshund Smooth Haired Cross/Type	4715.7	0.3	American Akita Cross/Type	73.23	0.3	Bearded Collie Cross/Type	239.15	0.2	Jack Russell Terrier X Poodle	245.2	0.3
42	French Bulldog Cross/Type	4703.64	0.3	Retriever Golden Cross/Type	70.94	0.2	Bullmastiff Cross/Type	239.15	0.2	Boxer Cross/Type	239.15	0.3
43	Poodle X Shih Tzu	4420.22	0.3	Border Collie X Retriever Labrador	68.66	0.2	Jack Russell Terrier X Poodle	239.15	0.2	Bullmastiff Cross/Type	227.04	0.3
44	Shar Pei Cross/Type	3847.34	0.3	Border Collie X Jack Russell Terrier	64.08	0.2	Border Collie X Staffordshire Bull Terrier	236.05	0.2	Spaniel English Springer X Spaniel Welsh Springer	220.98	0.3
45	Jack Russell Terrier X Parson Russell Terrier	3711.66	0.2	Beagle X Pug	61.79	0.2	French Bulldog Cross/Type	217.41	0.2	Lakeland Terrier Cross/Type	196.77	0.2
46	Lakeland Terrier Cross/Type	3244.31	0.2	Bullmastiff Cross/Type	61.79	0.2	Retriever Golden Cross/Type	214.3	0.2	Greyhound Cross/Type	193.74	0.2
47	American Akita Cross/Type	3180.99	0.2	Welsh Corgi Pembroke Cross/Type	59.5	0.2	Border Collie X Retriever Labrador	211.2	0.2	Mastiff Cross/Type	187.69	0.2
48	Greyhound Cross/Type	3111.64	0.2	Schnauzer Cross/Type	57.21	0.2	Poodle X Shih Tzu	204.99	0.2	Jack Russell Terrier X Yorkshire Terrier	184.66	0.2

49	Saluki Cross/Type	3078.47	0.2	Lakeland Terrier Cross/Type	54.92	0.2	Shar Pei Cross/Type	201.88	0.2	Poodle X Shih Tzu	178.6	0.2
50	Bedlington Terrier Cross/Type	2945.81	0.2	American Staffordshire Terrier Cross/Type	52.64	0.2	American Staffordshire Terrier Cross/Type	189.46	0.1	American Staffordshire Terrier Cross/Type	175.58	0.2
51	Dogue De Bordeaux Cross/Type	2843.29	0.2	Collie Smooth Cross/Type	52.64	0.2	Cairn Terrier Cross/Type	189.46	0.1	Bichon Frise X Shih Tzu	172.55	0.2
52	Jack Russell Terrier X Yorkshire Terrier	2828.22	0.2	Bearded Collie Cross/Type	45.77	0.2	Cavalier King Charles Spaniel Cross/Type	186.35	0.1	Dobermann X German Pinscher	163.47	0.2
53	American Staffordshire Terrier Cross/Type	2822.19	0.2	French Bulldog Cross/Type	45.77	0.2	Dachshund Smooth Haired Cross/Type	183.25	0.1	Rhodesian Ridgeback Cross/Type	154.39	0.2
54	Dobermann X German Pinscher	2749.82	0.2	Poodle X Retriever Golden	45.77	0.2	Beagle Cross/Type	180.14	0.1	Jack Russell Terrier X Parson Russell Terrier	148.33	0.2
55	Chihuahua Smooth Coat X Jack Russell Terrier	2701.58	0.2	Bulldog X Bull Terrier	36.62	0.1	Dalmatian Cross/Type	180.14	0.1	Bedlington Terrier Cross/Type	142.28	0.2
56	Cairn Terrier Cross/Type	2614.14	0.2	Dalmatian Cross/Type	36.62	0.1	Great Dane X Mastiff	177.03	0.1	Saluki Cross/Type	139.25	0.2
57	Spaniel English Springer X Spaniel Welsh Springer	2457.35	0.2	Jack Russell Terrier X Yorkshire Terrier	36.62	0.1	Lakeland Terrier Cross/Type	177.03	0.1	Welsh Corgi Pembroke Cross/Type	139.25	0.2
58	Retriever Golden Cross/Type	2421.17	0.2	Mastiff Cross/Type	36.62	0.1	Bullmastiff X Spanish Mastiff	152.19	0.1	Miniature Schnauzer Cross/Type	133.2	0.2
59	Alaskan Malamute Cross/Type	2418.16	0.2	Shar Pei Cross/Type	36.62	0.1	Pointer Cross/Type	142.87	0.1	Cairn Terrier Cross/Type	130.17	0.2
60	Bull Terrier Cross/Type	2391.02	0.2	Great Dane X Mastiff	34.33	0.1	American Akita Cross/Type	130.45	0.1	Chihuahua Long Coat Cross/Type	124.11	0.2
61	Pointer Cross/Type	2378.96	0.2	Greyhound Cross/Type	34.33	0.1	Greyhound Cross/Type	124.23	0.1	Dalmatian Cross/Type	124.11	0.2
62	Boxer X Staffordshire Bull Terrier	2252.32	0.2	King Charles Spaniel Cross/Type	34.33	0.1	Border Collie X Jack Russell Terrier	114.92	0.1	Schnauzer Cross/Type	124.11	0.2
63	Border Collie X Retriever Labrador	2204.08	0.1	German Shorthaired Pointer X Weimaraner	32.04	0.1	Boxer X Staffordshire Bull Terrier	111.81	0.1	Bulldog X Bull Terrier	121.09	0.2
64	Maltese X Poodle	2047.29	0.1	Jack Russell Terrier X Poodle	32.04	0.1	King Charles Spaniel Cross/Type	111.81	0.1	Bearded Collie Cross/Type	108.98	0.1
65	Chihuahua Long Coat Cross/Type	2044.28	0.1	Poodle X Shih Tzu	32.04	0.1	Bichon Frise X Shih Tzu	108.71	0.1	Australian Kelpie Cross/Type	105.95	0.1

66	Rhodesian Ridgeback Cross/Type	2026.18	0.1	Dobermann Cross/Type	29.75	0.1	Miniature Schnauzer Cross/Type	108.71	0.1	Retriever Golden Cross/Type	105.95	0.1
67	Dalmatian Cross/Type	1905.58	0.1	Border Collie X Staffordshire Bull Terrier	27.46	0.1	Dobermann X Miniature Pinscher	102.49	0.1	Border Collie X Retriever Labrador	102.92	0.1
68	Poodle X Schnauzer	1851.31	0.1	Irish Red And White Setter Cross/Type	27.46	0.1	Dogue De Bordeaux Cross/Type	102.49	0.1	Collie Smooth Cross/Type	102.92	0.1
69	Bearded Collie Cross/Type	1830.2	0.1	Poodle X Schnauzer	27.46	0.1	Chihuahua Smooth Coat X Jack Russell Terrier	99.39	0.1	Cavalier King Charles Spaniel X Pug	99.9	0.1
70	Welsh Corgi Pembroke Cross/Type	1754.82	0.1	Collie Rough Cross/Type	25.17	0.1	Retriever Labrador X Spaniel Cocker	99.39	0.1	Dogue De Bordeaux Cross/Type	99.9	0.1
71	King Charles Spaniel Cross/Type	1703.56	0.1	Dogue De Bordeaux Cross/Type	25.17	0.1	Rhodesian Ridgeback Cross/Type	96.28	0.1	Bichon Frise X Yorkshire Terrier	96.87	0.1
72	Bulldog X Bull Terrier	1694.52	0.1	Rhodesian Ridgeback Cross/Type	25.17	0.1	Schnauzer Cross/Type	93.18	0.1	Border Collie X Jack Russell Terrier	96.87	0.1
73	Border Collie X Staffordshire Bull Terrier	1661.35	0.1	Spaniel English Springer X Spaniel Welsh Springer	25.17	0.1	Spaniel English Springer X Spaniel Welsh Springer	93.18	0.1	King Charles Spaniel Cross/Type	93.84	0.1
74	Dobermann Cross/Type	1643.26	0.1	Basset Hound Cross/Type	22.89	0.1	Cavalier King Charles Spaniel X Pug	86.96	0.1	Dobermann Cross/Type	84.76	0.1
75	Scottish Terrier Cross/Type	1616.12	0.1	Newfoundland Cross/Type	22.89	0.1	Saluki Cross/Type	86.96	0.1	Chihuahua Smooth Coat X Jack Russell Terrier	81.73	0.1
76	Bulldog X Staffordshire Bull Terrier	1613.11	0.1	Retriever Labrador X Siberian Husky	22.89	0.1	Poodle X Spaniel English Springer	83.86	0.1	Retriever Labrador X Spaniel Cocker	81.73	0.1
77	Poodle X Spaniel English Springer	1549.79	0.1	Alaskan Malamute Cross/Type	20.6	0.1	Poodle X Schnauzer	80.75	0.1	Border Collie X Staffordshire Bull Terrier	78.71	0.1
78	German Shorthaired Pointer X Weimaraner	1471.4	0.1	Boxer X Staffordshire Bull Terrier	20.6	0.1	Bull Terrier Cross/Type	77.65	0.1	Boston Terrier Cross/Type	78.71	0.1
79	Basset Hound Cross/Type	1450.29	0.1	Chihuahua Long Coat Cross/Type	20.6	0.1	Italian Cane Corso X Mastiff	77.65	0.1	German Shorthaired Pointer X Weimaraner	78.71	0.1
80	Bichon Frise X Shih Tzu	1447.27	0.1	Fox Terrier Wire Cross/Type	20.6	0.1	Jack Russell Terrier X Yorkshire Terrier	77.65	0.1	Poodle X Spaniel English Springer	78.71	0.1

81	Cavalier King Charles Spaniel X Pug	1405.06	0.1	Foxhound Cross/Type	20.6	0.1	Scottish Terrier Cross/Type	77.65	0.1	Scottish Terrier Cross/Type	78.71	0.1
82	Miniature Schnauzer Cross/Type	1405.06	0.1	Japanese Akita Inu Cross/Type	20.6	0.1	Retriever Labrador X Siberian Husky	74.54	0.1	American Akita Cross/Type	75.68	0.1
83	Retriever Labrador X Spaniel Cocker	1402.05	0.1	Pointer Cross/Type	20.6	0.1	Bedlington Terrier Cross/Type	68.33	0.1	Basset Hound Cross/Type	75.68	0.1
84	Border Collie X Jack Russell Terrier	1393	0.1	Samoyed Cross/Type	20.6	0.1	Collie Smooth Cross/Type	68.33	0.1	Bull Terrier Cross/Type	75.68	0.1
85	Schnauzer Cross/Type	1383.96	0.1	Belgian Shepherd Dog Malinois Cross/Type	18.31	0.1	German Shorthaired Pointer Cross/Type	68.33	0.1	Bullmastiff X Spanish Mastiff	75.68	0.1
86	Japanese Akita Inu Cross/Type	1332.7	0.1	Bernese Mountain Dog Cross/Type	18.31	0.1	Great Dane Cross/Type	68.33	0.1	Boxer X Staffordshire Bull Terrier	72.65	0.1
87	Great Dane X Mastiff	1275.41	0.1	Scottish Terrier Cross/Type	18.31	0.1	Maltese X Poodle	68.33	0.1	Pointer Cross/Type	66.6	0.1
88	Greyhound X Saluki	1266.37	0.1	Soft Coated Wheaten Terrier Cross/Type	18.31	0.1	Newfoundland Cross/Type	68.33	0.1	Chinese Crested Cross/Type	63.57	0.1
89	Chinese Crested Cross/Type	1230.18	0.1	Tibetan Terrier Cross/Type	18.31	0.1	Pekingese Cross/Type	65.22	<0.1	Belgian Shepherd Dog Malinois Cross/Type	60.54	0.1
90	Bullmastiff X Spanish Mastiff	1166.87	0.1	Retriever Labrador X Spaniel Cocker	16.02	0.1	Retriever Golden X Retriever Labrador	65.22	<0.1	Retriever Labrador X Siberian Husky	60.54	0.1
91	Poodle X West Highland White Terrier	1154.8	0.1	Retriever Labrador X Spaniel English Springer	16.02	0.1	Bichon Frise X Yorkshire Terrier	62.12	<0.1	Tibetan Terrier Cross/Type	60.54	0.1
92	Belgian Shepherd Dog Malinois Cross/Type	1130.68	0.1	Border Terrier X Yorkshire Terrier	13.73	<0.1	Papillon Cross/Type	62.12	<0.1	Bulldog X Staffordshire Bull Terrier	57.52	0.1
93	German Shepherd Dog X Rottweiler	1100.53	0.1	Bullmastiff X Spanish Mastiff	13.73	<0.1	Welsh Corgi Pembroke Cross/Type	62.12	<0.1	Newfoundland Cross/Type	57.52	0.1
94	Newfoundland Cross/Type	1094.5	0.1	Chihuahua Smooth Coat X Jack Russell Terrier	13.73	<0.1	Boston Terrier Cross/Type	59.01	<0.1	Italian Cane Corso X Mastiff	54.49	0.1
95	Boston Terrier Cross/Type	1064.35	0.1	Irish Wolfhound Cross/Type	13.73	<0.1	Dobermann Cross/Type	55.91	<0.1	Poodle X West Highland White Terrier	51.46	0.1

96	Great Dane Cross/Type	1064.35	0.1	Old English Sheepdog Cross/Type	13.73	<0.1	German Shepherd Dog X Rottweiler	55.91	<0.1	Maltese X Poodle	48.43	0.1
97	Fox Terrier Wire Cross/Type	1043.24	0.1	Bedlington Terrier Cross/Type	11.44	<0.1	Tibetan Terrier Cross/Type	55.91	<0.1	Pekingese Cross/Type	42.38	0.1
98	Retriever Labrador X Spaniel English Springer	1031.18	0.1	Bichon Frise X Yorkshire Terrier	11.44	<0.1	Bulldog X Bull Terrier	52.8	<0.1	Poodle X Schnauzer	42.38	0.1
99	Retriever Labrador X Siberian Husky	1016.11	0.1	Chow Chow Cross/Type	11.44	<0.1	Bulldog X Staffordshire Bull Terrier	52.8	<0.1	Collie Rough Cross/Type	39.35	<0.1
100	Norfolk Terrier Cross/Type	982.94	0.1	German Spitz Mittel Cross/Type	11.44	<0.1	Chinese Crested Cross/Type	49.69	<0.1	Deerhound Cross/Type	39.35	<0.1
101	Border Terrier X Lakeland Terrier	946.76	0.1	Greyhound X Saluki	11.44	<0.1	Japanese Akita Inu Cross/Type	46.59	<0.1	Great Dane X Mastiff	39.35	<0.1
102	Collie Smooth Cross/Type	946.76	0.1	Hungarian Vizsla Cross/Type	11.44	<0.1	Fox Terrier Wire Cross/Type	43.48	<0.1	Welsh Terrier Cross/Type/type	39.35	<0.1
103	Papillon Cross/Type	931.68	0.1	Jack Russell Terrier X Parson Russell Terrier	11.44	<0.1	Chihuahua Long Coat Cross/Type	40.38	<0.1	Dachshund Miniature Smooth Haired Cross/Type	36.33	<0.1
104	Tibetan Terrier Cross/Type	928.67	0.1	Pekingese Cross/Type	11.44	<0.1	Irish Red And White Setter Cross/Type	40.38	<0.1	Pekingese X Poodle	36.33	<0.1
105	Dachshund Miniature Smooth Haired Cross/Type	889.47	0.1	Border Terrier X Lakeland Terrier	9.15	<0.1	Border Terrier X Lakeland Terrier	37.27	<0.1	German Shorthaired Pointer Cross/Type	33.3	<0.1
106	Poodle X Yorkshire Terrier	880.43	0.1	Bull Terrier Cross/Type	9.15	<0.1	Border Terrier X Yorkshire Terrier	37.27	<0.1	Great Dane Cross/Type	33.3	<0.1
107	Weimaraner Cross/Type	865.35	0.1	English Setter Cross/Type	9.15	<0.1	German Spitz Mittel Cross/Type	37.27	<0.1	Hungarian Vizsla Cross/Type	33.3	<0.1
108	Italian Cane Corso X Mastiff	853.29	0.1	Great Dane Cross/Type	9.15	<0.1	Australian Kelpie Cross/Type	34.16	<0.1	Papillon Cross/Type	33.3	<0.1
109	German Shorthaired Pointer Cross/Type	814.09	0.1	Italian Cane Corso X Mastiff	9.15	<0.1	Bichon Frise X Bolognese	34.16	<0.1	Weimaraner Cross/Type	33.3	<0.1
110	Miniature Pinscher Cross/Type	811.08	0.1	Kerry Blue Terrier Cross/Type	9.15	<0.1	Boxer X Bull Terrier	34.16	<0.1	Presa Canario Cross/Type	30.27	<0.1
111	Parson Russell Terrier Cross/Type	783.94	0.1	Poodle X West Highland White Terrier	9.15	<0.1	Chihuahua Smooth Coat X Papillon	34.16	<0.1	Samoyed Cross/Type	27.24	<0.1

112	Bichon Frise X Yorkshire Terrier	762.83	0.1	Saluki Cross/Type	9.15	<0.1	Deerhound Cross/Type	34.16	<0.1	Shetland Sheepdog Cross/Type	27.24	<0.1
113	Hungarian Vizsla Cross/Type	714.59	<0.1	Shetland Sheepdog Cross/Type	9.15	<0.1	Retriever Labrador X Spaniel English Springer	34.16	<0.1	Fox Terrier Wire Cross/Type	24.22	<0.1
114	Pekingese Cross/Type	714.59	<0.1	Spaniel Irish Water Cross/Type	9.15	<0.1	Greyhound X Saluki	31.06	<0.1	German Spitz Mittel Cross/Type	24.22	<0.1
115	Collie Rough Cross/Type	705.55	<0.1	St Bernard Cross/Type	9.15	<0.1	Havanese Cross/Type	31.06	<0.1	Greyhound X Saluki	24.22	<0.1
116	Retriever Labrador X Staffordshire Bull Terrier	687.46	<0.1	Weimaraner Cross/Type	9.15	<0.1	Japanese Chin Cross/Type	31.06	<0.1	Jack Russell Terrier X Lakeland Terrier	24.22	<0.1
117	Australian Kelpie Cross/Type	642.23	<0.1	Deerhound Cross/Type	6.87	<0.1	Poodle X West Highland White Terrier	31.06	<0.1	Miniature Pinscher Cross/Type	24.22	<0.1
118	Jack Russell Terrier X Lakeland Terrier	618.11	<0.1	Dobermann X Miniature Pinscher	6.87	<0.1	St Bernard Cross/Type	31.06	<0.1	Border Terrier X Lakeland Terrier	21.19	<0.1
119	Presa Canario Cross/Type	612.08	<0.1	German Shorthaired Pointer Cross/Type	6.87	<0.1	Collie Rough Cross/Type	27.95	<0.1	Cavalier King Charles Spaniel X Spaniel American Cocker	21.19	<0.1
120	Dobermann X Miniature Pinscher	590.97	<0.1	Irish Terrier Cross/Type	6.87	<0.1	Irish Wolfhound Cross/Type	27.95	<0.1	Chihuahua Smooth Coat X Papillon	21.19	<0.1
121	Mastiff X Staffordshire Bull Terrier	578.91	<0.1	Miniature Pinscher Cross/Type	6.87	<0.1	Retriever Labrador X Staffordshire Bull Terrier	27.95	<0.1	German Shepherd Dog X Poodle	21.19	<0.1
122	Border Terrier X Yorkshire Terrier	566.85	<0.1	Norwegian Elkhound Cross/Type	6.87	<0.1	Belgian Shepherd Dog Malinois Cross/Type	24.85	<0.1	Japanese Akita Inu Cross/Type	21.19	<0.1
123	German Spitz Mittel Cross/Type	518.61	<0.1	Parson Russell Terrier Cross/Type	6.87	<0.1	English Setter Cross/Type	24.85	<0.1	Beagle X Jack Russell Terrier	18.16	<0.1
124	Shetland Sheepdog Cross/Type	509.56	<0.1	Schipperke X Siberian Husky	6.87	<0.1	Italian Greyhound Cross/Type	24.85	<0.1	Boston Terrier X Bulldog	18.16	<0.1
125	Deerhound Cross/Type	503.53	<0.1	Airedale Terrier Cross/Type	4.58	<0.1	Miniature Pinscher Cross/Type	24.85	<0.1	German Shepherd Dog X Rottweiler	18.16	<0.1
126	Bichon Frise X Poodle	491.47	<0.1	Australian Kelpie Cross/Type	4.58	<0.1	Parson Russell Terrier Cross/Type	24.85	<0.1	Norfolk Terrier Cross/Type	18.16	<0.1

127	Old English Sheepdog Cross/Type	479.41	<0.1	Australian Shepherd Cross/Type	4.58	<0.1	Presa Canario Cross/Type	24.85	<0.1	Old English Sheepdog Cross/Type	18.16	<0.1
128	Bichon Frise X Bolognese	476.39	<0.1	Beagle X Harrier	4.58	<0.1	Bichon Frise X Havanese	21.74	<0.1	Parson Russell Terrier Cross/Type	18.16	<0.1
129	Retriever Flat Coated Cross/Type	467.35	<0.1	Bichon Frise X Bolognese	4.58	<0.1	Cavalier King Charles Spaniel X Spaniel American Cocker	21.74	<0.1	Bichon Frise X Havanese	15.14	<0.1
130	Chihuahua Smooth Coat X Papillon	458.3	<0.1	Bichon Frise X Havanese	4.58	<0.1	Hungarian Vizsla Cross/Type	21.74	<0.1	Foxhound Cross/Type	15.14	<0.1
131	Retriever Golden X Retriever Labrador	446.24	<0.1	Black And Tan Coonhound Cross/Type	4.58	<0.1	Schipperke X Siberian Husky	21.74	<0.1	Landseer X Newfoundland	15.14	<0.1
132	Irish Wolfhound Cross/Type	425.14	<0.1	Boston Terrier X Bulldog	4.58	<0.1	Basset Hound Cross/Type	18.64	<0.1	Manchester Terrier Cross/Type	15.14	<0.1
133	Poodle X Pug	425.14	<0.1	Bouvier Des Flandres Cross/Type	4.58	<0.1	Chihuahua Smooth Coat X Poodle	18.64	<0.1	Tibetan Spaniel Cross/Type	15.14	<0.1
134	Jack Russell Terrier X Staffordshire Bull Terrier	422.12	<0.1	Bulldog X Staffordshire Bull Terrier	4.58	<0.1	German Shepherd Dog X Poodle	18.64	<0.1	Airedale Terrier Cross/Type	12.11	<0.1
135	St Bernard Cross/Type	419.11	<0.1	Czechoslovakia n Wolfdog Cross/Type	4.58	<0.1	Irish Terrier Cross/Type	18.64	<0.1	Border Terrier X Yorkshire Terrier	12.11	<0.1
136	Manchester Terrier Cross/Type	404.03	<0.1	German Shepherd Dog X Poodle	4.58	<0.1	Old English Sheepdog Cross/Type	18.64	<0.1	Boxer X Bull Terrier	12.11	<0.1
137	Chow Chow Cross/Type	401.02	<0.1	Griffon Bruxellois Cross/Type	4.58	<0.1	Shetland Sheepdog Cross/Type	18.64	<0.1	Dobermann X Miniature Pinscher	12.11	<0.1
138	Italian Greyhound Cross/Type	394.99	<0.1	Harrier Cross/Type	4.58	<0.1	Beagle X Cavalier King Charles Spaniel	15.53	<0.1	English Setter Cross/Type	12.11	<0.1
139	Foxhound Cross/Type	391.97	<0.1	Irish Setter Cross/Type	4.58	<0.1	Neapolitan Mastiff Cross/Type	15.53	<0.1	Griffon Bruxellois Cross/Type	12.11	<0.1
140	Basset Hound X Spaniel English Springer	388.96	<0.1	Japanese Spitz Cross/Type	4.58	<0.1	Poodle X Yorkshire Terrier	15.53	<0.1	Irish Red And White Setter Cross/Type	12.11	<0.1
141	Cavalier King Charles Spaniel X Spaniel American Cocker	385.94	<0.1	Maltese X Poodle	4.58	<0.1	Russian Black Terrier Cross/Type	15.53	<0.1	Japanese Chin Cross/Type	12.11	<0.1

142	Border Terrier X Jack Russell Terrier	376.89	<0.1	Norfolk Terrier Cross/Type	4.58	<0.1	Samoyed Cross/Type	15.53	<0.1	Pyrenean Mountain Dog Cross/Type	12.11	<0.1
143	German Shepherd Dog X Retriever Labrador	364.83	<0.1	Norwegian Buhund Cross/Type	4.58	<0.1	Tibetan Spaniel Cross/Type	15.53	<0.1	Retriever Flat Coated Cross/Type	12.11	<0.1
144	Schipperke X Siberian Husky	361.82	<0.1	Poodle X Spaniel English Springer	4.58	<0.1	Weimaraner Cross/Type	15.53	<0.1	Retriever Labrador X Spaniel English Springer	12.11	<0.1
145	Shih Tzu X Yorkshire Terrier	361.82	<0.1	Presa Canario Cross/Type	4.58	<0.1	Anatolian Shepherd Dog X Turkish Kangal Dog	12.42	<0.1	St Bernard Cross/Type	12.11	<0.1
146	Griffon Bruxellois Cross/Type	352.77	<0.1	Spanish Water Dog Cross/Type	4.58	<0.1	Australian Cattle Dog Cross/Type	12.42	<0.1	Anatolian Shepherd Dog X Turkish Kangal Dog	9.08	<0.1
147	Samoyed Cross/Type	349.76	<0.1	Tibetan Spaniel Cross/Type	4.58	<0.1	Bernese Mountain Dog Cross/Type	12.42	<0.1	Australian Cattle Dog Cross/Type	9.08	<0.1
148	Coton De Tulear Cross/Type	343.73	<0.1	Affenpinscher X Yorkshire Terrier	2.29	<0.1	Black And Tan Coonhound Cross/Type	12.42	<0.1	Australian Shepherd Cross/Type	9.08	<0.1
149	Neapolitan Mastiff Cross/Type	343.73	<0.1	Australian Cattle Dog Cross/Type	2.29	<0.1	Border Collie X Siberian Husky	12.42	<0.1	Beauceron Cross/Type	9.08	<0.1
150	Bichon Frise X Havanese	319.61	<0.1	Bavarian Mountain Hound Cross/Type	2.29	<0.1	Boston Terrier X Bulldog	12.42	<0.1	Bichon Frise X Bolognese	9.08	<0.1
151	Border Collie X German Shepherd Dog	316.59	<0.1	Beagle X Cavalier King Charles Spaniel	2.29	<0.1	Chihuahua Smooth Coat X Shih Tzu	12.42	<0.1	Bichon Frise X Poodle	9.08	<0.1
152	English Setter Cross/Type	307.55	<0.1	Bichon Frise X Shih Tzu	2.29	<0.1	Dutch Shepherd Dog Cross/Type	12.42	<0.1	Chow Chow Cross/Type	9.08	<0.1
153	Japanese Chin Cross/Type	295.49	<0.1	Borzoi Cross/Type	2.29	<0.1	German Wirehaired Pointer Cross/Type	12.42	<0.1	Coton De Tulear Cross/Type	9.08	<0.1
154	Spaniel American Cocker Cross/Type	295.49	<0.1	Caucasian Shepherd Dog Cross/Type	2.29	<0.1	Griffon Bruxellois Cross/Type	12.42	<0.1	Dachshund Wire Haired Cross/Type	9.08	<0.1
155	Chihuahua Smooth Coat X Poodle	289.45	<0.1	Cavalier King Charles Spaniel X Chihuahua Smooth Coat	2.29	<0.1	Jack Russell Terrier X Lakeland Terrier	12.42	<0.1	Irish Terrier Cross/Type	9.08	<0.1
156	Havanese Cross/Type	289.45	<0.1	Chinese Crested Cross/Type	2.29	<0.1	Kerry Blue Terrier Cross/Type	12.42	<0.1	Irish Wolfhound Cross/Type	9.08	<0.1

157	Airedale Terrier Cross/Type	280.41	<0.1	Dachshund Miniature Smooth Haired Cross/Type	2.29	<0.1	Retriever Labrador X Whippet	12.42	<0.1	Italian Greyhound Cross/Type	9.08	<0.1
158	German Shepherd Dog X Siberian Husky	277.39	<0.1	Dachshund Miniature Wire Haired Cross/Type	2.29	<0.1	Border Collie X Lakeland Terrier	9.32	<0.1	Leonberger Cross/Type	9.08	<0.1
159	Rottweiler X Staffordshire Bull Terrier	274.38	<0.1	Dachshund Wire Haired Cross/Type	2.29	<0.1	Bouvier Des Flandres Cross/Type	9.32	<0.1	Poodle X Pug	9.08	<0.1
160	Bullmastiff X Staffordshire Bull Terrier	271.36	<0.1	Dutch Shepherd Dog Cross/Type	2.29	<0.1	Canarian Warren Hound Cross/Type	9.32	<0.1	Poodle X Yorkshire Terrier	9.08	<0.1
161	Dogue De Bordeaux X Retriever Labrador	268.35	<0.1	German Spitz Giant Cross/Type	2.29	<0.1	Chihuahua Smooth Coat X Pomeranian	9.32	<0.1	Retriever Labrador X Staffordshire Bull Terrier	9.08	<0.1
162	Alaskan Malamute X Siberian Husky	262.32	<0.1	Great Swiss Mountain Dog Cross/Type	2.29	<0.1	Dachshund Miniature Smooth Haired Cross/Type	9.32	<0.1	Soft Coated Wheaten Terrier Cross/Type	9.08	<0.1
163	Maltese X Shih Tzu	259.3	<0.1	Havanese Cross/Type	2.29	<0.1	Japanese Spitz Cross/Type	9.32	<0.1	Spanish Mastiff Cross/Type	9.08	<0.1
164	Shar Pei X Staffordshire Bull Terrier	250.26	<0.1	Jack Russell Terrier X Lakeland Terrier	2.29	<0.1	Landseer X Newfoundland	9.32	<0.1	Staffordshire Bull Terrier X Whippet	9.08	<0.1
165	Irish Red And White Setter Cross/Type	241.21	<0.1	Jamthund Cross/Type	2.29	<0.1	Maltese X Shih Tzu	9.32	<0.1	Staffordshire Bull Terrier X Yorkshire Terrier	9.08	<0.1
166	Bernese Mountain Dog Cross/Type	235.18	<0.1	Landseer X Newfoundland	2.29	<0.1	Norfolk Terrier Cross/Type	9.32	<0.1	Afghan Hound Cross/Type	6.05	<0.1
167	Jack Russell Terrier X Shih Tzu	235.18	<0.1	Large Münsterländer X Pointer	2.29	<0.1	Portuguese Water Dog Cross/Type	9.32	<0.1	Beagle X Harrier	6.05	<0.1
168	Border Collie X Spaniel English Springer	229.15	<0.1	Leonberger Cross/Type	2.29	<0.1	Retriever Flat Coated Cross/Type	9.32	<0.1	Bichon Frise X Pug	6.05	<0.1
169	Maltese X Yorkshire Terrier	226.14	<0.1	Manchester Terrier Cross/Type	2.29	<0.1	Soft Coated Wheaten Terrier Cross/Type	9.32	<0.1	Canaan Dog Cross/Type	6.05	<0.1
170	Chihuahua Smooth Coat X Shih Tzu	220.11	<0.1	Neapolitan Mastiff Cross/Type	2.29	<0.1	Spaniel American Cocker Cross/Type	9.32	<0.1	Cavalier King Charles Spaniel X Shih Tzu	6.05	<0.1
171	German Shepherd Dog X Staffordshire Bull Terrier	217.09	<0.1	Papillon Cross/Type	2.29	<0.1	Spanish Mastiff Cross/Type	9.32	<0.1	Chihuahua Smooth Coat X Maltese	6.05	<0.1

172	Welsh Terrier Cross/Type/type	214.08	<0.1	Pomeranian X Shih Tzu	2.29	<0.1	American Akita X Rottweiler	6.21	<0.1	Chihuahua Smooth Coat X Poodle	6.05	<0.1
173	Rhodesian Ridgeback X Staffordshire Bull Terrier	208.05	<0.1	Poodle X Pug	2.29	<0.1	Bavarian Mountain Hound Cross/Type	6.21	<0.1	Croatian Shepherd Dog Cross/Type	6.05	<0.1
174	Beagle X Jack Russell Terrier	205.03	<0.1	Poodle X Yorkshire Terrier	2.29	<0.1	Beagle X Jack Russell Terrier	6.21	<0.1	Giant Schnauzer Cross/Type	6.05	<0.1
175	Chihuahua Smooth Coat X Pomeranian	205.03	<0.1	Retriever Flat Coated Cross/Type	2.29	<0.1	Bullmastiff X Staffordshire Bull Terrier	6.21	<0.1	Hellenic Hound Cross/Type	6.05	<0.1
176	Tibetan Spaniel Cross/Type	202.02	<0.1	Retriever Golden X Retriever Labrador	2.29	<0.1	Chihuahua Smooth Coat X Pug	6.21	<0.1	Jack Russell Terrier X Staffordshire Bull Terrier	6.05	<0.1
177	Romanian Carpathian Shepherd Dog Cross/Type	199	<0.1	Retriever Labrador X Whippet	2.29	<0.1	Chow Chow Cross/Type	6.21	<0.1	Jack Russell Terrier X Welsh Corgi Pembroke	6.05	<0.1
178	Pomeranian X Shih Tzu	195.99	<0.1	Schipperke Cross/Type	2.29	<0.1	Dachshund Smooth Haired X Jack Russell Terrier	6.21	<0.1	Japanese Shiba Inu Cross/Type	6.05	<0.1
179	Jack Russell Terrier X West Highland White Terrier	192.97	<0.1	Spaniel American Water Cross/Type	2.29	<0.1	English Toy Terrier Cross/Type	6.21	<0.1	Japanese Spitz Cross/Type	6.05	<0.1
180	Irish Terrier Cross/Type	189.95	<0.1	Stabijhoun Cross/Type	2.29	<0.1	French Bulldog X Pug	6.21	<0.1	Kerry Blue Terrier Cross/Type	6.05	<0.1
181	Chihuahua Smooth Coat X Pug	186.94	<0.1	Staffordshire Bull Terrier X Yorkshire Terrier	2.29	<0.1	German Pinscher Cross/Type	6.21	<0.1	Norwich Terrier Cross/Type	6.05	<0.1
182	Boston Terrier X Bulldog	183.92	<0.1	White Swiss Shepherd Dog Cross/Type	2.29	<0.1	Ibizan Hound Cross/Type	6.21	<0.1	Picardy Sheepdog Cross/Type	6.05	<0.1
183	Pug X Shih Tzu	183.92	<0.1	NA	NA	NA	Jack Russell Terrier X Staffordshire Bull Terrier	6.21	<0.1	Retriever Golden X Retriever Labrador	6.05	<0.1
184	Lancashire Heeler Cross/Type	177.89	<0.1	NA	NA	NA	Jack Russell Terrier X Welsh Corgi Pembroke	6.21	<0.1	Retriever Labrador X Spaniel American Cocker	6.05	<0.1
185	Landseer X Newfoundland	177.89	<0.1	NA	NA	NA	Lhasa Apso X Maltese	6.21	<0.1	Sloughi Cross/Type	6.05	<0.1
186	Dachshund Smooth Haired X Jack Russell Terrier	174.88	<0.1	NA	NA	NA	Lhasa Apso X Yorkshire Terrier	6.21	<0.1	Spaniel American Cocker Cross/Type	6.05	<0.1

187	French Bulldog X Pug	168.85	<0.1	NA	NA	NA	Pomeranian X Shih Tzu	6.21	<0.1	Welsh Corgi Cardigan Cross/Type	6.05	<0.1
188	Jack Russell Terrier X Pomeranian	165.83	<0.1	NA	NA	NA	Pug X Yorkshire Terrier	6.21	<0.1	Alpine Dachsbracke Cross/Type	3.03	<0.1
189	Boxer X Bull Terrier	162.82	<0.1	NA	NA	NA	Pyrenean Mountain Dog Cross/Type	6.21	<0.1	Anatolian Shepherd Dog Cross/Type	3.03	<0.1
190	Retriever Labrador X Rottweiler	162.82	<0.1	NA	NA	NA	Spaniel Irish Water Cross/Type	6.21	<0.1	Basset Griffon Vendeen Petit Cross/Type	3.03	<0.1
191	Staffordshire Bull Terrier X Whippet	156.79	<0.1	NA	NA	NA	Spanish Hound Cross/Type	6.21	<0.1	Beagle X Border Collie	3.03	<0.1
192	Staffordshire Bull Terrier X Yorkshire Terrier	156.79	<0.1	NA	NA	NA	Staffordshire Bull Terrier X Whippet	6.21	<0.1	Bedlington Terrier X Greyhound	3.03	<0.1
193	Australian Shepherd Cross/Type	153.77	<0.1	NA	NA	NA	Staffordshire Bull Terrier X Yorkshire Terrier	6.21	<0.1	Bernese Mountain Dog Cross/Type	3.03	<0.1
194	Australian Cattle Dog Cross/Type	150.76	<0.1	NA	NA	NA	Swiss Hound Cross/Type	6.21	<0.1	Bolognese Cross/Type	3.03	<0.1
195	Leonberger Cross/Type	150.76	<0.1	NA	NA	NA	Welsh Terrier Cross/Type/type	6.21	<0.1	Border Collie X Dobermann	3.03	<0.1
196	Soft Coated Wheaten Terrier Cross/Type	147.74	<0.1	NA	NA	NA	Alaskan Malamute X German Shepherd Dog	3.11	<0.1	Border Collie X Spaniel Cocker	3.03	<0.1
197	Black And Tan Coonhound Cross/Type	144.73	<0.1	NA	NA	NA	Anatolian Shepherd Dog Cross/Type	3.11	<0.1	Border Collie X Welsh Corgi Pembroke	3.03	<0.1
198	Spaniel Clumber Cross/Type	144.73	<0.1	NA	NA	NA	Australian Shepherd Cross/Type	3.11	<0.1	Border Terrier X Jack Russell Terrier	3.03	<0.1
199	German Shepherd Dog X Poodle	141.71	<0.1	NA	NA	NA	Basset Hound X Beagle	3.11	<0.1	Briard Cross/Type	3.03	<0.1
200	Border Collie X Spaniel Cocker	138.7	<0.1	NA	NA	NA	Beagle X Harrier	3.11	<0.1	Brittany Cross/Type	3.03	<0.1
201	German Wirehaired Pointer Cross/Type	138.7	<0.1	NA	NA	NA	Belgian Shepherd Dog Groenendael Cross/Type	3.11	<0.1	Brittany X Pointer	3.03	<0.1
202	Bichon Frise X Maltese	135.68	<0.1	NA	NA	NA	Bichon Frise X Lhasa Apso	3.11	<0.1	Bulldog X Pekingese	3.03	<0.1
203	Brittany Cross/Type	135.68	<0.1	NA	NA	NA	Borzoi X Deerhound	3.11	<0.1	Bullmastiff X Rottweiler	3.03	<0.1
204	Australian Silky Terrier Cross/Type	132.67	<0.1	NA	NA	NA	Bosnian Broken Haired Hound Cross/Type	3.11	<0.1	Catalan Sheepdog Cross/Type	3.03	<0.1

205	Japanese Spitz Cross/Type	132.67	<0.1	NA	NA	NA	Briard Cross/Type	3.11	<0.1	Chihuahua Smooth Coat X Pomeranian	3.03	<0.1
206	Portuguese Podengo Cross/Type	132.67	<0.1	NA	NA	NA	Brittany Cross/Type	3.11	<0.1	Dachshund Miniature Long Haired Cross/Type	3.03	<0.1
207	Dachshund Miniature Long Haired Cross/Type	129.65	<0.1	NA	NA	NA	Bull Terrier Miniature Cross/Type	3.11	<0.1	Dachshund Miniature Wire Haired Cross/Type	3.03	<0.1
208	Cavalier King Charles Spaniel X Retriever Labrador	126.64	<0.1	NA	NA	NA	Bulldog X Pekingese	3.11	<0.1	Dachshund Smooth Haired X Jack Russell Terrier	3.03	<0.1
209	Canarian Warren Hound Cross/Type	123.62	<0.1	NA	NA	NA	Cairn Terrier X Yorkshire Terrier	3.11	<0.1	Dutch Shepherd Dog Cross/Type	3.03	<0.1
210	Spanish Mastiff Cross/Type	120.61	<0.1	NA	NA	NA	Canaan Dog Cross/Type	3.11	<0.1	Eurasier Cross/Type	3.03	<0.1
211	Cavalier King Charles Spaniel X Shih Tzu	117.59	<0.1	NA	NA	NA	Caucasian Shepherd Dog Cross/Type	3.11	<0.1	Fox Terrier Smooth Cross/Type	3.03	<0.1
212	Bulldog X Mastiff	114.58	<0.1	NA	NA	NA	Cavalier King Charles Spaniel X Chihuahua Smooth Coat	3.11	<0.1	German Longhaired Pointer Cross/Type	3.03	<0.1
213	Lhasa Apso X Maltese	114.58	<0.1	NA	NA	NA	Cavalier King Charles Spaniel X Retriever Labrador	3.11	<0.1	German Shepherd Dog X Retriever Labrador	3.03	<0.1
214	Pyrenean Mountain Dog Cross/Type	108.55	<0.1	NA	NA	NA	Cavalier King Charles Spaniel X Shih Tzu	3.11	<0.1	German Shepherd Dog X Rhodesian Ridgeback	3.03	<0.1
215	Spanish Water Dog Cross/Type	108.55	<0.1	NA	NA	NA	Cavalier King Charles Spaniel X Yorkshire Terrier	3.11	<0.1	German Wirehaired Pointer Cross/Type	3.03	<0.1
216	Beauceron Cross/Type	105.53	<0.1	NA	NA	NA	Chihuahua Smooth Coat X Dachshund Smooth Haired	3.11	<0.1	Great Swiss Mountain Dog Cross/Type	3.03	<0.1
217	Bichon Frise X Miniature Schnauzer	105.53	<0.1	NA	NA	NA	Chihuahua Smooth Coat X Maltese	3.11	<0.1	Harrier Cross/Type	3.03	<0.1
218	Boxer X Retriever Labrador	105.53	<0.1	NA	NA	NA	Coton De Tulear Cross/Type	3.11	<0.1	Ibizan Hound Cross/Type	3.03	<0.1
219	Retriever Labrador X Whippet	105.53	<0.1	NA	NA	NA	Czechoslovakian Wolfdog Cross/Type	3.11	<0.1	Jack Russell Terrier X	3.03	<0.1

										Miniature Schnauzer		
220	Bichon Frise X West Highland White Terrier	102.52	<0.1	NA	NA	NA	Dachshund Wire Haired Cross/Type	3.11	<0.1	Jack Russell Terrier X Retriever Labrador	3.03	<0.1
221	Giant Schnauzer Cross/Type	99.5	<0.1	NA	NA	NA	Dobermann X Retriever Labrador	3.11	<0.1	Keeshond Cross/Type	3.03	<0.1
222	Pomeranian X Poodle	99.5	<0.1	NA	NA	NA	Dobermann X Staffordshire Bull Terrier	3.11	<0.1	Korean Jindo Cross/Type	3.03	<0.1
223	Anatolian Shepherd Dog Cross/Type	96.48	<0.1	NA	NA	NA	French Bulldog X Shih Tzu	3.11	<0.1	Korthals Griffon Cross/Type	3.03	<0.1
224	Bloodhound Cross/Type	96.48	<0.1	NA	NA	NA	German Longhaired Pointer Cross/Type	3.11	<0.1	Lhasa Apso X Maltese	3.03	<0.1
225	Caucasian Shepherd Dog Cross/Type	96.48	<0.1	NA	NA	NA	German Shepherd Dog X Retriever Labrador	3.11	<0.1	Maltese X Shih Tzu	3.03	<0.1
226	Siberian Husky X Staffordshire Bull Terrier	96.48	<0.1	NA	NA	NA	German Shepherd Dog X Staffordshire Bull Terrier	3.11	<0.1	Maltese X Yorkshire Terrier	3.03	<0.1
227	American Akita X German Shepherd Dog	93.47	<0.1	NA	NA	NA	German Spitz Klein Cross/Type	3.11	<0.1	Neapolitan Mastiff Cross/Type	3.03	<0.1
228	Anatolian Shepherd Dog X Turkish Kangal Dog	93.47	<0.1	NA	NA	NA	Giant Schnauzer Cross/Type	3.11	<0.1	Pharaoh Hound Cross/Type	3.03	<0.1
229	Bichon Frise X Jack Russell Terrier	93.47	<0.1	NA	NA	NA	Gordon Setter Cross/Type	3.11	<0.1	Pointer X Small Münsterländer	3.03	<0.1
230	Bullmastiff X Rottweiler	93.47	<0.1	NA	NA	NA	Harrier Cross/Type	3.11	<0.1	Pointer X Spaniel Cocker	3.03	<0.1
231	Jack Russell Terrier X Welsh Corgi Pembroke	93.47	<0.1	NA	NA	NA	Jack Russell Terrier X Spaniel English Springer	3.11	<0.1	Pomeranian X Shih Tzu	3.03	<0.1
232	Pekingese X Poodle	93.47	<0.1	NA	NA	NA	Japanese Shiba Inu Cross/Type	3.11	<0.1	Portuguese Podengo Cross/Type	3.03	<0.1
233	Pointer X Retriever Labrador	93.47	<0.1	NA	NA	NA	Kai Cross/Type	3.11	<0.1	Portuguese Water Dog Cross/Type	3.03	<0.1
234	Pointer X Small Münsterländer	93.47	<0.1	NA	NA	NA	Kishu Cross/Type	3.11	<0.1	Pug X Shih Tzu	3.03	<0.1

235	Border Collie X Siberian Husky	87.44	<0.1	NA	NA	NA	Large Münsterländer Cross/Type	3.11	<0.1	Pyrenean Mastiff Cross/Type	3.03	<0.1
236	Dutch Shepherd Dog Cross/Type	87.44	<0.1	NA	NA	NA	Large Münsterländer X Pointer	3.11	<0.1	Retriever Curly Coated Cross/Type	3.03	<0.1
237	Chihuahua Smooth Coat X Dachshund Smooth Haired	84.42	<0.1	NA	NA	NA	Maltese X Yorkshire Terrier	3.11	<0.1	Retriever Labrador X Whippet	3.03	<0.1
238	Lhasa Apso X Shih Tzu	84.42	<0.1	NA	NA	NA	Norwegian Elkhound Cross/Type	3.11	<0.1	Romanian Carpathian Shepherd Dog Cross/Type	3.03	<0.1
239	Retriever Labrador X Spaniel American Cocker	84.42	<0.1	NA	NA	NA	Picardy Sheepdog Cross/Type	3.11	<0.1	Schipperke X Siberian Husky	3.03	<0.1
240	Czechoslovakia n Wolfdog Cross/Type	81.41	<0.1	NA	NA	NA	Pointer X Retriever Labrador	3.11	<0.1	Sealyham Terrier Cross/Type	3.03	<0.1
241	Japanese Shiba Inu Cross/Type	81.41	<0.1	NA	NA	NA	Pointer X Small Münsterländer	3.11	<0.1	Shar Pei X Staffordshire Bull Terrier	3.03	<0.1
242	Chihuahua Smooth Coat X Maltese	78.39	<0.1	NA	NA	NA	Poodle X Pug	3.11	<0.1	Small Münsterländer Cross/Type	3.03	<0.1
243	Lhasa Apso X Yorkshire Terrier	78.39	<0.1	NA	NA	NA	Portuguese Podengo Cross/Type	3.11	<0.1	Spaniel Clumber Cross/Type	3.03	<0.1
244	Miniature Schnauzer X Yorkshire Terrier	78.39	<0.1	NA	NA	NA	Pyrenean Sheepdog Long Haired Cross/Type	3.11	<0.1	Spaniel Sussex Cross/Type	3.03	<0.1
245	West Highland White Terrier X Yorkshire Terrier	78.39	<0.1	NA	NA	NA	Rafeiro do Alentejo Cross/Type	3.11	<0.1	Spanish Greyhound Cross/Type	3.03	<0.1
246	Bichon Frise X Lhasa Apso	75.38	<0.1	NA	NA	NA	Retriever Chesapeake Bay Cross/Type	3.11	<0.1	Spanish Water Dog Cross/Type	3.03	<0.1
247	American Akita X Staffordshire Bull Terrier	72.36	<0.1	NA	NA	NA	Retriever Labrador X Rottweiler	3.11	<0.1	Thai Ridgeback Dog Cross/Type	3.03	<0.1
248	Harrier Cross/Type	72.36	<0.1	NA	NA	NA	Rhodesian Ridgeback X Staffordshire Bull Terrier	3.11	<0.1	Xoloitzcuintle Cross/Type	3.03	<0.1
249	Beagle X Cavalier King Charles Spaniel	69.35	<0.1	NA	NA	NA	Romanian Carpathian Shepherd Dog Cross/Type	3.11	<0.1	NA	NA	NA

250	Affenpinscher Cross/Type	66.33	<0.1	NA	NA	NA	Russian Toy Cross/Type	3.11	<0.1	NA	NA	NA
251	Afghan Hound Cross/Type	66.33	<0.1	NA	NA	NA	Saint Germain Pointer Cross/Type	3.11	<0.1	NA	NA	NA
252	Beagle X Harrier	66.33	<0.1	NA	NA	NA	Shih Tzu X Yorkshire Terrier	3.11	<0.1	NA	NA	NA
253	Bedlington Terrier X Whippet	66.33	<0.1	NA	NA	NA	Stabijhoun Cross/Type	3.11	<0.1	NA	NA	NA
254	Bulldog X Bullmastiff	66.33	<0.1	NA	NA	NA	Welsh Corgi Cardigan Cross/Type	3.11	<0.1	NA	NA	NA
255	Hellenic Hound Cross/Type	66.33	<0.1	NA	NA	NA	NA	NA	NA	NA	NA	NA
256	Hungarian Puli Cross/Type	66.33	<0.1	NA	NA	NA	NA	NA	NA	NA	NA	NA
257	Mastiff X Rottweiler	66.33	<0.1	NA	NA	NA	NA	NA	NA	NA	NA	NA
258	Pomeranian X Siberian Husky	66.33	<0.1	NA	NA	NA	NA	NA	NA	NA	NA	NA
259	Basenji Cross/Type	63.32	<0.1	NA	NA	NA	NA	NA	NA	NA	NA	NA
260	Basset Griffon Vendeen Petit Cross/Type	63.32	<0.1	NA	NA	NA	NA	NA	NA	NA	NA	NA
261	Bulldog X Pekingese	63.32	<0.1	NA	NA	NA	NA	NA	NA	NA	NA	NA
262	French Bulldog X Staffordshire Bull Terrier	63.32	<0.1	NA	NA	NA	NA	NA	NA	NA	NA	NA
263	German Pinscher Cross/Type	63.32	<0.1	NA	NA	NA	NA	NA	NA	NA	NA	NA
264	Gordon Setter Cross/Type	63.32	<0.1	NA	NA	NA	NA	NA	NA	NA	NA	NA
265	Jack Russell Terrier X Spaniel English Springer	63.32	<0.1	NA	NA	NA	NA	NA	NA	NA	NA	NA
266	Saluki X Whippet	63.32	<0.1	NA	NA	NA	NA	NA	NA	NA	NA	NA
267	Affenpinscher X Yorkshire Terrier	60.3	<0.1	NA	NA	NA	NA	NA	NA	NA	NA	NA
268	Cavalier King Charles Spaniel X Chihuahua Smooth Coat	60.3	<0.1	NA	NA	NA	NA	NA	NA	NA	NA	NA
269	Italian Spinone Cross/Type	60.3	<0.1	NA	NA	NA	NA	NA	NA	NA	NA	NA

270	Jack Russell Terrier X Lhasa Apso	60.3	<0.1	NA	NA	NA	NA	NA	NA	NA	NA	NA
271	Pomeranian X Yorkshire Terrier	60.3	<0.1	NA	NA	NA	NA	NA	NA	NA	NA	NA
272	Alaskan Malamute X German Shepherd Dog	57.29	<0.1	NA	NA	NA	NA	NA	NA	NA	NA	NA
273	Dogue De Bordeaux X Staffordshire Bull Terrier	57.29	<0.1	NA	NA	NA	NA	NA	NA	NA	NA	NA
274	Bichon Frise X Chihuahua Smooth Coat	54.27	<0.1	NA	NA	NA	NA	NA	NA	NA	NA	NA
275	Boxer X Mastiff	54.27	<0.1	NA	NA	NA	NA	NA	NA	NA	NA	NA
276	Dalmatian X Retriever Labrador	54.27	<0.1	NA	NA	NA	NA	NA	NA	NA	NA	NA
277	Finnish Spitz Cross/Type	54.27	<0.1	NA	NA	NA	NA	NA	NA	NA	NA	NA
278	Bolognese Cross/Type	51.26	<0.1	NA	NA	NA	NA	NA	NA	NA	NA	NA
279	Border Collie X Dachshund Smooth Haired	51.26	<0.1	NA	NA	NA	NA	NA	NA	NA	NA	NA
280	Border Collie X Poodle	51.26	<0.1	NA	NA	NA	NA	NA	NA	NA	NA	NA
281	Boxer X Bullmastiff	51.26	<0.1	NA	NA	NA	NA	NA	NA	NA	NA	NA
282	Cairn Terrier X Jack Russell Terrier	51.26	<0.1	NA	NA	NA	NA	NA	NA	NA	NA	NA
283	Cairn Terrier X Yorkshire Terrier	51.26	<0.1	NA	NA	NA	NA	NA	NA	NA	NA	NA
284	Chihuahua Smooth Coat X Miniature Pinscher	51.26	<0.1	NA	NA	NA	NA	NA	NA	NA	NA	NA
285	Lakeland Terrier X Yorkshire Terrier	51.26	<0.1	NA	NA	NA	NA	NA	NA	NA	NA	NA
286	Lhasa Apso X Poodle	51.26	<0.1	NA	NA	NA	NA	NA	NA	NA	NA	NA
287	Retriever Labrador X Rhodesian Ridgeback	51.26	<0.1	NA	NA	NA	NA	NA	NA	NA	NA	NA
288	Skye Terrier Cross/Type	51.26	<0.1	NA	NA	NA	NA	NA	NA	NA	NA	NA

289	Spaniel Field Cross/Type	51.26	<0.1	NA	NA	NA	NA	NA	NA	NA	NA	NA
290	Thai Ridgeback Dog Cross/Type	51.26	<0.1	NA	NA	NA	NA	NA	NA	NA	NA	NA
291	Tibetan Mastiff Cross/Type	51.26	<0.1	NA	NA	NA	NA	NA	NA	NA	NA	NA
292	Cavalier King Charles Spaniel X Jack Russell Terrier	48.24	<0.1	NA	NA	NA	NA	NA	NA	NA	NA	NA
293	Chihuahua Smooth Coat X French Bulldog	48.24	<0.1	NA	NA	NA	NA	NA	NA	NA	NA	NA
294	Collie Rough X Collie Smooth	48.24	<0.1	NA	NA	NA	NA	NA	NA	NA	NA	NA
295	Greyhound X Whippet	48.24	<0.1	NA	NA	NA	NA	NA	NA	NA	NA	NA
296	Jack Russell Terrier X Spaniel Cocker	48.24	<0.1	NA	NA	NA	NA	NA	NA	NA	NA	NA
297	Sealyham Terrier Cross/Type	48.24	<0.1	NA	NA	NA	NA	NA	NA	NA	NA	NA
298	Borzoi X Deerhound	45.23	<0.1	NA	NA	NA	NA	NA	NA	NA	NA	NA
299	Cavalier King Charles Spaniel X Pomeranian	45.23	<0.1	NA	NA	NA	NA	NA	NA	NA	NA	NA
300	Chihuahua Smooth Coat X Pekingese	45.23	<0.1	NA	NA	NA	NA	NA	NA	NA	NA	NA
301	Dachshund Miniature Wire Haired Cross/Type	45.23	<0.1	NA	NA	NA	NA	NA	NA	NA	NA	NA
302	German Longhaired Pointer Cross/Type	45.23	<0.1	NA	NA	NA	NA	NA	NA	NA	NA	NA
303	Kerry Blue Terrier Cross/Type	45.23	<0.1	NA	NA	NA	NA	NA	NA	NA	NA	NA
304	Border Collie X Whippet	42.21	<0.1	NA	NA	NA	NA	NA	NA	NA	NA	NA
305	Bulldog X Rottweiler	42.21	<0.1	NA	NA	NA	NA	NA	NA	NA	NA	NA
306	Catalan Sheepdog Cross/Type	42.21	<0.1	NA	NA	NA	NA	NA	NA	NA	NA	NA
307	Cavalier King Charles Spaniel	42.21	<0.1	NA	NA	NA	NA	NA	NA	NA	NA	NA

	X Spaniel Cocker											
308	Dachshund Long Haired Cross/Type	42.21	<0.1	NA	NA	NA	NA	NA	NA	NA	NA	NA
309	Dachshund Smooth Haired X Poodle	42.21	<0.1	NA	NA	NA	NA	NA	NA	NA	NA	NA
310	Dobermann X Rottweiler	42.21	<0.1	NA	NA	NA	NA	NA	NA	NA	NA	NA
311	Dogue De Bordeaux X Rottweiler	42.21	<0.1	NA	NA	NA	NA	NA	NA	NA	NA	NA
312	Norwich Terrier Cross/Type	42.21	<0.1	NA	NA	NA	NA	NA	NA	NA	NA	NA
313	American Akita X Siberian Husky	39.2	<0.1	NA	NA	NA	NA	NA	NA	NA	NA	NA
314	Bichon Frise X Pomeranian	39.2	<0.1	NA	NA	NA	NA	NA	NA	NA	NA	NA
315	Bichon Frise X Pug	39.2	<0.1	NA	NA	NA	NA	NA	NA	NA	NA	NA
316	Border Collie X Lakeland Terrier	39.2	<0.1	NA	NA	NA	NA	NA	NA	NA	NA	NA
317	Bulldog X French Bulldog	39.2	<0.1	NA	NA	NA	NA	NA	NA	NA	NA	NA
318	Bulldog X Pug	39.2	<0.1	NA	NA	NA	NA	NA	NA	NA	NA	NA
319	Bulldog X Scottish Terrier	39.2	<0.1	NA	NA	NA	NA	NA	NA	NA	NA	NA
320	Cesky Terrier Cross/Type	39.2	<0.1	NA	NA	NA	NA	NA	NA	NA	NA	NA
321	Dobermann X Retriever Labrador	39.2	<0.1	NA	NA	NA	NA	NA	NA	NA	NA	NA
322	English Toy Terrier Cross/Type	39.2	<0.1	NA	NA	NA	NA	NA	NA	NA	NA	NA
323	German Shepherd Dog X Mastiff	39.2	<0.1	NA	NA	NA	NA	NA	NA	NA	NA	NA
324	Löwchen Cross/Type	39.2	<0.1	NA	NA	NA	NA	NA	NA	NA	NA	NA
325	Poodle X Spaniel American Cocker	39.2	<0.1	NA	NA	NA	NA	NA	NA	NA	NA	NA
326	Russian Black Terrier Cross/Type	39.2	<0.1	NA	NA	NA	NA	NA	NA	NA	NA	NA
327	Sloughi Cross/Type	39.2	<0.1	NA	NA	NA	NA	NA	NA	NA	NA	NA

328	Bedlington Terrier X Greyhound	36.18	<0.1	NA	NA	NA	NA	NA	NA	NA	NA	NA
329	Bichon Frise X Spaniel Cocker	36.18	<0.1	NA	NA	NA	NA	NA	NA	NA	NA	NA
330	Border Collie X Rottweiler	36.18	<0.1	NA	NA	NA	NA	NA	NA	NA	NA	NA
331	Chihuahua Smooth Coat X Chinese Crested	36.18	<0.1	NA	NA	NA	NA	NA	NA	NA	NA	NA
332	Chihuahua Smooth Coat X Lhasa Apso	36.18	<0.1	NA	NA	NA	NA	NA	NA	NA	NA	NA
333	Dachshund Wire Haired Cross/Type	36.18	<0.1	NA	NA	NA	NA	NA	NA	NA	NA	NA
334	Dandie Dinmont Terrier Cross/Type	36.18	<0.1	NA	NA	NA	NA	NA	NA	NA	NA	NA
335	Dobermann X Staffordshire Bull Terrier	36.18	<0.1	NA	NA	NA	NA	NA	NA	NA	NA	NA
336	Hungarian Vizsla X Retriever Labrador	36.18	<0.1	NA	NA	NA	NA	NA	NA	NA	NA	NA
337	Miniature Schnauzer X Poodle	36.18	<0.1	NA	NA	NA	NA	NA	NA	NA	NA	NA
338	Picardy Sheepdog Cross/Type	36.18	<0.1	NA	NA	NA	NA	NA	NA	NA	NA	NA
339	Pug X Yorkshire Terrier	36.18	<0.1	NA	NA	NA	NA	NA	NA	NA	NA	NA
340	Welsh Corgi Cardigan Cross/Type	36.18	<0.1	NA	NA	NA	NA	NA	NA	NA	NA	NA
341	Bull Terrier Miniature Cross/Type	33.17	<0.1	NA	NA	NA	NA	NA	NA	NA	NA	NA
342	Bulldog X Dogue De Bordeaux	33.17	<0.1	NA	NA	NA	NA	NA	NA	NA	NA	NA
343	Dogue De Bordeaux X Mastiff	33.17	<0.1	NA	NA	NA	NA	NA	NA	NA	NA	NA
344	Glen Of Imaal Terrier Cross/Type	33.17	<0.1	NA	NA	NA	NA	NA	NA	NA	NA	NA
345	Ibizan Hound Cross/Type	33.17	<0.1	NA	NA	NA	NA	NA	NA	NA	NA	NA

346	Italian Spinone X Schnauzer	33.17	<0.1	NA	NA	NA	NA	NA	NA	NA	NA	NA
347	Jack Russell Terrier X Maltese	33.17	<0.1	NA	NA	NA	NA	NA	NA	NA	NA	NA
348	Jack Russell Terrier X Norfolk Terrier	33.17	<0.1	NA	NA	NA	NA	NA	NA	NA	NA	NA
349	Mastiff X Retriever Labrador	33.17	<0.1	NA	NA	NA	NA	NA	NA	NA	NA	NA
350	Norwegian Elkhound Cross/Type	33.17	<0.1	NA	NA	NA	NA	NA	NA	NA	NA	NA
351	Papillon X Pomeranian	33.17	<0.1	NA	NA	NA	NA	NA	NA	NA	NA	NA
352	Portuguese Pointer Cross/Type	33.17	<0.1	NA	NA	NA	NA	NA	NA	NA	NA	NA
353	Russian Toy Cross/Type	33.17	<0.1	NA	NA	NA	NA	NA	NA	NA	NA	NA
354	Shih Tzu X West Highland White Terrier	33.17	<0.1	NA	NA	NA	NA	NA	NA	NA	NA	NA
355	Border Collie X Retriever Golden	30.15	<0.1	NA	NA	NA	NA	NA	NA	NA	NA	NA
356	Briard Cross/Type	30.15	<0.1	NA	NA	NA	NA	NA	NA	NA	NA	NA
357	Canadian Eskimo Dog Cross/Type	30.15	<0.1	NA	NA	NA	NA	NA	NA	NA	NA	NA
358	German Shepherd Dog X Greyhound	30.15	<0.1	NA	NA	NA	NA	NA	NA	NA	NA	NA
359	Jack Russell Terrier X Whippet	30.15	<0.1	NA	NA	NA	NA	NA	NA	NA	NA	NA
360	Korthals Griffon Cross/Type	30.15	<0.1	NA	NA	NA	NA	NA	NA	NA	NA	NA
361	Large Münsterländer Cross/Type	30.15	<0.1	NA	NA	NA	NA	NA	NA	NA	NA	NA
362	Pomeranian X Pug	30.15	<0.1	NA	NA	NA	NA	NA	NA	NA	NA	NA
363	Retriever Nova Scotia Duck Tolling Cross/Type	30.15	<0.1	NA	NA	NA	NA	NA	NA	NA	NA	NA
364	Siberian Husky X Weimaraner	30.15	<0.1	NA	NA	NA	NA	NA	NA	NA	NA	NA

365	Spaniel Clumber X Spaniel English Springer	30.15	<0.1	NA	NA	NA	NA	NA	NA	NA	NA	NA
366	Spaniel English Springer X Staffordshire Bull Terrier	30.15	<0.1	NA	NA	NA	NA	NA	NA	NA	NA	NA
367	Spanish Greyhound Cross/Type	30.15	<0.1	NA	NA	NA	NA	NA	NA	NA	NA	NA
368	American Akita X Retriever Labrador	27.14	<0.1	NA	NA	NA	NA	NA	NA	NA	NA	NA
369	American Akita X Rottweiler	27.14	<0.1	NA	NA	NA	NA	NA	NA	NA	NA	NA
370	Basset Hound X Retriever Labrador	27.14	<0.1	NA	NA	NA	NA	NA	NA	NA	NA	NA
371	Basset Hound X Shar Pei	27.14	<0.1	NA	NA	NA	NA	NA	NA	NA	NA	NA
372	Beagle X Retriever Labrador	27.14	<0.1	NA	NA	NA	NA	NA	NA	NA	NA	NA
373	Border Terrier X Poodle	27.14	<0.1	NA	NA	NA	NA	NA	NA	NA	NA	NA
374	Boxer X Bulldog	27.14	<0.1	NA	NA	NA	NA	NA	NA	NA	NA	NA
375	Bulldog X Retriever Labrador	27.14	<0.1	NA	NA	NA	NA	NA	NA	NA	NA	NA
376	Bulldog X Shar Pei	27.14	<0.1	NA	NA	NA	NA	NA	NA	NA	NA	NA
377	Croatian Shepherd Dog Cross/Type	27.14	<0.1	NA	NA	NA	NA	NA	NA	NA	NA	NA
378	Dachshund Miniature Smooth Haired X Jack Russell Terrier	27.14	<0.1	NA	NA	NA	NA	NA	NA	NA	NA	NA
379	French Bulldog X Shih Tzu	27.14	<0.1	NA	NA	NA	NA	NA	NA	NA	NA	NA
380	Jack Russell Terrier X King Charles Spaniel	27.14	<0.1	NA	NA	NA	NA	NA	NA	NA	NA	NA
381	King Charles Spaniel X Shih Tzu	27.14	<0.1	NA	NA	NA	NA	NA	NA	NA	NA	NA
382	Mastiff X Rhodesian Ridgeback	27.14	<0.1	NA	NA	NA	NA	NA	NA	NA	NA	NA

383	Miniature Pinscher X Pug	27.14	<0.1	NA	NA	NA	NA	NA	NA	NA	NA	NA
384	Pharaoh Hound Cross/Type	27.14	<0.1	NA	NA	NA	NA	NA	NA	NA	NA	NA
385	Retriever Labrador X Shar Pei	27.14	<0.1	NA	NA	NA	NA	NA	NA	NA	NA	NA
386	Schnauzer X Spaniel Cocker	27.14	<0.1	NA	NA	NA	NA	NA	NA	NA	NA	NA
387	Spaniel Irish Water Cross/Type	27.14	<0.1	NA	NA	NA	NA	NA	NA	NA	NA	NA
388	Beagle X Staffordshire Bull Terrier	24.12	<0.1	NA	NA	NA	NA	NA	NA	NA	NA	NA
389	Belgian Shepherd Dog Groenendael Cross/Type	24.12	<0.1	NA	NA	NA	NA	NA	NA	NA	NA	NA
390	Border Collie X Saluki	24.12	<0.1	NA	NA	NA	NA	NA	NA	NA	NA	NA
391	German Spitz Mittel X Pomeranian	24.12	<0.1	NA	NA	NA	NA	NA	NA	NA	NA	NA
392	Irish Setter Cross/Type	24.12	<0.1	NA	NA	NA	NA	NA	NA	NA	NA	NA
393	Otterhound Cross/Type	24.12	<0.1	NA	NA	NA	NA	NA	NA	NA	NA	NA
394	Poodle X Shetland Sheepdog	24.12	<0.1	NA	NA	NA	NA	NA	NA	NA	NA	NA
395	Rottweiler X Siberian Husky	24.12	<0.1	NA	NA	NA	NA	NA	NA	NA	NA	NA
396	Schnauzer X West Highland White Terrier	24.12	<0.1	NA	NA	NA	NA	NA	NA	NA	NA	NA
397	Swiss Hound Cross/Type	24.12	<0.1	NA	NA	NA	NA	NA	NA	NA	NA	NA
398	Border Collie X Welsh Corgi Pembroke	21.11	<0.1	NA	NA	NA	NA	NA	NA	NA	NA	NA
399	Border Terrier X West Highland White Terrier	21.11	<0.1	NA	NA	NA	NA	NA	NA	NA	NA	NA
400	Boston Terrier X French Bulldog	21.11	<0.1	NA	NA	NA	NA	NA	NA	NA	NA	NA
401	Boston Terrier X Pug	21.11	<0.1	NA	NA	NA	NA	NA	NA	NA	NA	NA
402	Boxer X Rottweiler	21.11	<0.1	NA	NA	NA	NA	NA	NA	NA	NA	NA

403	Cairn Terrier X West Highland White Terrier	21.11	<0.1	NA	NA	NA	NA	NA	NA	NA	NA	NA
404	Central Asia Shepherd Dog Cross/Type	21.11	<0.1	NA	NA	NA	NA	NA	NA	NA	NA	NA
405	German Shepherd Dog X Retriever Golden	21.11	<0.1	NA	NA	NA	NA	NA	NA	NA	NA	NA
406	German Shepherd Dog X Rhodesian Ridgeback	21.11	<0.1	NA	NA	NA	NA	NA	NA	NA	NA	NA
407	German Shepherd Dog X Spaniel English Springer	21.11	<0.1	NA	NA	NA	NA	NA	NA	NA	NA	NA
408	Jack Russell Terrier X Retriever Labrador	21.11	<0.1	NA	NA	NA	NA	NA	NA	NA	NA	NA
409	Maltese X West Highland White Terrier	21.11	<0.1	NA	NA	NA	NA	NA	NA	NA	NA	NA
410	Norfolk Terrier X Yorkshire Terrier	21.11	<0.1	NA	NA	NA	NA	NA	NA	NA	NA	NA
411	Pekingese X Shih Tzu	21.11	<0.1	NA	NA	NA	NA	NA	NA	NA	NA	NA
412	Spaniel English Springer X Weimaraner	21.11	<0.1	NA	NA	NA	NA	NA	NA	NA	NA	NA
413	American Akita X Mastiff	18.09	<0.1	NA	NA	NA	NA	NA	NA	NA	NA	NA
414	Beagle X Kerry Blue Terrier	18.09	<0.1	NA	NA	NA	NA	NA	NA	NA	NA	NA
415	Beagle X Pointer	18.09	<0.1	NA	NA	NA	NA	NA	NA	NA	NA	NA
416	Beagle X Spaniel Cocker	18.09	<0.1	NA	NA	NA	NA	NA	NA	NA	NA	NA
417	Bichon Frise X Schnauzer	18.09	<0.1	NA	NA	NA	NA	NA	NA	NA	NA	NA
418	Border Terrier X Pug	18.09	<0.1	NA	NA	NA	NA	NA	NA	NA	NA	NA
419	Bosnian Broken Haired Hound Cross/Type	18.09	<0.1	NA	NA	NA	NA	NA	NA	NA	NA	NA
420	Boxer X Great Dane	18.09	<0.1	NA	NA	NA	NA	NA	NA	NA	NA	NA
421	Bulldog X German Shepherd Dog	18.09	<0.1	NA	NA	NA	NA	NA	NA	NA	NA	NA

422	Bulldog X Saluki	18.09	<0.1	NA	NA	NA	NA	NA	NA	NA	NA	NA
423	Coton De Tulear X Poodle	18.09	<0.1	NA	NA	NA	NA	NA	NA	NA	NA	NA
424	Fox Terrier Wire X Jack Russell Terrier	18.09	<0.1	NA	NA	NA	NA	NA	NA	NA	NA	NA
425	Greyhound X Staffordshire Bull Terrier	18.09	<0.1	NA	NA	NA	NA	NA	NA	NA	NA	NA
426	Jack Russell Terrier X Schnauzer	18.09	<0.1	NA	NA	NA	NA	NA	NA	NA	NA	NA
427	Lhasa Apso X Pug	18.09	<0.1	NA	NA	NA	NA	NA	NA	NA	NA	NA
428	Majorca Mastiff Cross/Type	18.09	<0.1	NA	NA	NA	NA	NA	NA	NA	NA	NA
429	Maltese X Pomeranian	18.09	<0.1	NA	NA	NA	NA	NA	NA	NA	NA	NA
430	Miniature Schnauzer X West Highland White Terrier	18.09	<0.1	NA	NA	NA	NA	NA	NA	NA	NA	NA
431	Papillon X Shih Tzu	18.09	<0.1	NA	NA	NA	NA	NA	NA	NA	NA	NA
432	Pointer X Spaniel English Springer	18.09	<0.1	NA	NA	NA	NA	NA	NA	NA	NA	NA
433	Retriever Chesapeake Bay Cross/Type	18.09	<0.1	NA	NA	NA	NA	NA	NA	NA	NA	NA
434	Retriever Curly Coated Cross/Type	18.09	<0.1	NA	NA	NA	NA	NA	NA	NA	NA	NA
435	Scottish Terrier X West Highland White Terrier	18.09	<0.1	NA	NA	NA	NA	NA	NA	NA	NA	NA
436	Scottish Terrier X Yorkshire Terrier	18.09	<0.1	NA	NA	NA	NA	NA	NA	NA	NA	NA
437	Shih Tzu X Spaniel Cocker	18.09	<0.1	NA	NA	NA	NA	NA	NA	NA	NA	NA
438	Spaniel Sussex Cross/Type	18.09	<0.1	NA	NA	NA	NA	NA	NA	NA	NA	NA
439	Australian Kelpie X Border Collie	15.08	<0.1	NA	NA	NA	NA	NA	NA	NA	NA	NA
440	Austrian Pinscher Cross/Type	15.08	<0.1	NA	NA	NA	NA	NA	NA	NA	NA	NA
441	Beagle X Boston Terrier	15.08	<0.1	NA	NA	NA	NA	NA	NA	NA	NA	NA
442	Beagle X Poodle	15.08	<0.1	NA	NA	NA	NA	NA	NA	NA	NA	NA

443	Beagle X Spaniel English Springer	15.08	<0.1	NA	NA	NA	NA	NA	NA	NA	NA	NA
444	Belgian Shepherd Dog Malinois X German Shepherd Dog	15.08	<0.1	NA	NA	NA	NA	NA	NA	NA	NA	NA
445	Belgian Shepherd Dog Malinois X Siberian Husky	15.08	<0.1	NA	NA	NA	NA	NA	NA	NA	NA	NA
446	Bernese Mountain Dog X Newfoundland	15.08	<0.1	NA	NA	NA	NA	NA	NA	NA	NA	NA
447	Border Collie X Dobermann	15.08	<0.1	NA	NA	NA	NA	NA	NA	NA	NA	NA
448	Border Collie X Yorkshire Terrier	15.08	<0.1	NA	NA	NA	NA	NA	NA	NA	NA	NA
449	Bouvier Des Flandres Cross/Type	15.08	<0.1	NA	NA	NA	NA	NA	NA	NA	NA	NA
450	Boxer X German Shepherd Dog	15.08	<0.1	NA	NA	NA	NA	NA	NA	NA	NA	NA
451	Bullmastiff X Great Dane	15.08	<0.1	NA	NA	NA	NA	NA	NA	NA	NA	NA
452	Cairn Terrier X Norfolk Terrier	15.08	<0.1	NA	NA	NA	NA	NA	NA	NA	NA	NA
453	Cavalier King Charles Spaniel X Lhasa Apso	15.08	<0.1	NA	NA	NA	NA	NA	NA	NA	NA	NA
454	Dachshund Smooth Haired X Yorkshire Terrier	15.08	<0.1	NA	NA	NA	NA	NA	NA	NA	NA	NA
455	Dobermann X German Shepherd Dog	15.08	<0.1	NA	NA	NA	NA	NA	NA	NA	NA	NA
456	Fox Terrier Smooth Cross/Type	15.08	<0.1	NA	NA	NA	NA	NA	NA	NA	NA	NA
457	Greyhound X Retriever Labrador	15.08	<0.1	NA	NA	NA	NA	NA	NA	NA	NA	NA
458	Keeshond Cross/Type	15.08	<0.1	NA	NA	NA	NA	NA	NA	NA	NA	NA
459	King Charles Spaniel X Pug	15.08	<0.1	NA	NA	NA	NA	NA	NA	NA	NA	NA
460	King Charles Spaniel X	15.08	<0.1	NA	NA	NA	NA	NA	NA	NA	NA	NA

	Spaniel English Springer											
461	Lhasa Apso X West Highland White Terrier	15.08	<0.1	NA	NA	NA	NA	NA	NA	NA	NA	NA
462	Maremma Sheepdog Cross/Type	15.08	<0.1	NA	NA	NA	NA	NA	NA	NA	NA	NA
463	Norrbottenspitz Cross/Type	15.08	<0.1	NA	NA	NA	NA	NA	NA	NA	NA	NA
464	Papillon X Spaniel Cocker	15.08	<0.1	NA	NA	NA	NA	NA	NA	NA	NA	NA
465	Pekingese X Pug	15.08	<0.1	NA	NA	NA	NA	NA	NA	NA	NA	NA
466	Portuguese Water Dog Cross/Type	15.08	<0.1	NA	NA	NA	NA	NA	NA	NA	NA	NA
467	Presa Canario X Staffordshire Bull Terrier	15.08	<0.1	NA	NA	NA	NA	NA	NA	NA	NA	NA
468	Pug X Staffordshire Bull Terrier	15.08	<0.1	NA	NA	NA	NA	NA	NA	NA	NA	NA
469	Retriever Golden X Spaniel Cocker	15.08	<0.1	NA	NA	NA	NA	NA	NA	NA	NA	NA
470	Spaniel Cocker X Staffordshire Bull Terrier	15.08	<0.1	NA	NA	NA	NA	NA	NA	NA	NA	NA
471	Spanish Hound Cross/Type	15.08	<0.1	NA	NA	NA	NA	NA	NA	NA	NA	NA
472	Staffordshire Bull Terrier X West Highland White Terrier	15.08	<0.1	NA	NA	NA	NA	NA	NA	NA	NA	NA
473	Affenpinscher X Poodle	12.06	<0.1	NA	NA	NA	NA	NA	NA	NA	NA	NA
474	American Akita X Dobermann	12.06	<0.1	NA	NA	NA	NA	NA	NA	NA	NA	NA
475	American Akita X Shar Pei	12.06	<0.1	NA	NA	NA	NA	NA	NA	NA	NA	NA
476	Basset Hound X Spaniel Cocker	12.06	<0.1	NA	NA	NA	NA	NA	NA	NA	NA	NA
477	Bedlington Terrier X Jack Russell Terrier	12.06	<0.1	NA	NA	NA	NA	NA	NA	NA	NA	NA
478	Belgian Shepherd Dog Malinois X Caucasian Shepherd Dog	12.06	<0.1	NA	NA	NA	NA	NA	NA	NA	NA	NA

479	Bichon Frise X Cairn Terrier	12.06	<0.1	NA	NA	NA	NA	NA	NA	NA	NA	NA
480	Bichon Frise X Dachshund Smooth Haired	12.06	<0.1	NA	NA	NA	NA	NA	NA	NA	NA	NA
481	Bichon Frise X Staffordshire Bull Terrier	12.06	<0.1	NA	NA	NA	NA	NA	NA	NA	NA	NA
482	Border Collie X Boxer	12.06	<0.1	NA	NA	NA	NA	NA	NA	NA	NA	NA
483	Border Collie X Greyhound	12.06	<0.1	NA	NA	NA	NA	NA	NA	NA	NA	NA
484	Bull Terrier X Mastiff	12.06	<0.1	NA	NA	NA	NA	NA	NA	NA	NA	NA
485	Bull Terrier X Whippet	12.06	<0.1	NA	NA	NA	NA	NA	NA	NA	NA	NA
486	Bullmastiff X Rhodesian Ridgeback	12.06	<0.1	NA	NA	NA	NA	NA	NA	NA	NA	NA
487	Canaan Dog Cross/Type	12.06	<0.1	NA	NA	NA	NA	NA	NA	NA	NA	NA
488	Cavalier King Charles Spaniel X West Highland White Terrier	12.06	<0.1	NA	NA	NA	NA	NA	NA	NA	NA	NA
489	Cavalier King Charles Spaniel X Yorkshire Terrier	12.06	<0.1	NA	NA	NA	NA	NA	NA	NA	NA	NA
490	Chihuahua Smooth Coat X Staffordshire Bull Terrier	12.06	<0.1	NA	NA	NA	NA	NA	NA	NA	NA	NA
491	Chihuahua Smooth Coat X West Highland White Terrier	12.06	<0.1	NA	NA	NA	NA	NA	NA	NA	NA	NA
492	Dachshund Smooth Haired X Retriever Labrador	12.06	<0.1	NA	NA	NA	NA	NA	NA	NA	NA	NA
493	Dalmatian X Staffordshire Bull Terrier	12.06	<0.1	NA	NA	NA	NA	NA	NA	NA	NA	NA
494	Dutch Shepherd Dog X Poodle	12.06	<0.1	NA	NA	NA	NA	NA	NA	NA	NA	NA
495	Fox Terrier Wire X Poodle	12.06	<0.1	NA	NA	NA	NA	NA	NA	NA	NA	NA
496	German Hound Cross/Type	12.06	<0.1	NA	NA	NA	NA	NA	NA	NA	NA	NA

497	German Shepherd Dog X Jack Russell Terrier	12.06	<0.1	NA	NA	NA	NA	NA	NA	NA	NA	NA
498	German Shepherd Dog X Japanese Akita Inu	12.06	<0.1	NA	NA	NA	NA	NA	NA	NA	NA	NA
499	German Shepherd Dog X Newfoundland	12.06	<0.1	NA	NA	NA	NA	NA	NA	NA	NA	NA
500	Greyhound X Sloughi	12.06	<0.1	NA	NA	NA	NA	NA	NA	NA	NA	NA
501	Hungarian Hound Transylvanian Scent Hound Cross/Type	12.06	<0.1	NA	NA	NA	NA	NA	NA	NA	NA	NA
502	Hungarian Kuvasz Cross/Type	12.06	<0.1	NA	NA	NA	NA	NA	NA	NA	NA	NA
503	Irish Terrier X Pug	12.06	<0.1	NA	NA	NA	NA	NA	NA	NA	NA	NA
504	Jack Russell Terrier X Siberian Husky	12.06	<0.1	NA	NA	NA	NA	NA	NA	NA	NA	NA
505	King Charles Spaniel X Spaniel Cocker	12.06	<0.1	NA	NA	NA	NA	NA	NA	NA	NA	NA
506	Kooikerhondje Cross/Type	12.06	<0.1	NA	NA	NA	NA	NA	NA	NA	NA	NA
507	Large Münsterländer X Pointer	12.06	<0.1	NA	NA	NA	NA	NA	NA	NA	NA	NA
508	Mastiff X Siberian Husky	12.06	<0.1	NA	NA	NA	NA	NA	NA	NA	NA	NA
509	Mudi Cross/Type	12.06	<0.1	NA	NA	NA	NA	NA	NA	NA	NA	NA
510	Pomeranian X Shetland Sheepdog	12.06	<0.1	NA	NA	NA	NA	NA	NA	NA	NA	NA
511	Rhodesian Ridgeback X Rottweiler	12.06	<0.1	NA	NA	NA	NA	NA	NA	NA	NA	NA
512	Romanian Bucovina Shepherd Cross/Type	12.06	<0.1	NA	NA	NA	NA	NA	NA	NA	NA	NA
513	Saarloos Wolfhound Cross/Type	12.06	<0.1	NA	NA	NA	NA	NA	NA	NA	NA	NA

514	Spaniel English Springer X Whippet	12.06	<0.1	NA	NA	NA	NA	NA	NA	NA	NA	NA
515	Yugoslavian Shepherd Dog Cross/Type	12.06	<0.1	NA	NA	NA	NA	NA	NA	NA	NA	NA
516	Auvergne Pointer Cross/Type	9.05	<0.1	NA	NA	NA	NA	NA	NA	NA	NA	NA
517	Basset Fauve De Bretagne Cross/Type	9.05	<0.1	NA	NA	NA	NA	NA	NA	NA	NA	NA
518	Basset Hound X Dachshund Smooth Haired	9.05	<0.1	NA	NA	NA	NA	NA	NA	NA	NA	NA
519	Bearded Collie X Border Collie	9.05	<0.1	NA	NA	NA	NA	NA	NA	NA	NA	NA
520	Bedlington Terrier X Poodle	9.05	<0.1	NA	NA	NA	NA	NA	NA	NA	NA	NA
521	Belgian Shepherd Dog Tervueren Cross/Type	9.05	<0.1	NA	NA	NA	NA	NA	NA	NA	NA	NA
522	Bichon Frise X Border Terrier	9.05	<0.1	NA	NA	NA	NA	NA	NA	NA	NA	NA
523	Border Collie X Bulldog	9.05	<0.1	NA	NA	NA	NA	NA	NA	NA	NA	NA
524	Border Collie X Cairn Terrier	9.05	<0.1	NA	NA	NA	NA	NA	NA	NA	NA	NA
525	Border Collie X Dalmatian	9.05	<0.1	NA	NA	NA	NA	NA	NA	NA	NA	NA
526	Border Collie X Pointer	9.05	<0.1	NA	NA	NA	NA	NA	NA	NA	NA	NA
527	Border Collie X Samoyed	9.05	<0.1	NA	NA	NA	NA	NA	NA	NA	NA	NA
528	Border Collie X Shetland Sheepdog	9.05	<0.1	NA	NA	NA	NA	NA	NA	NA	NA	NA
529	Border Terrier X Cairn Terrier	9.05	<0.1	NA	NA	NA	NA	NA	NA	NA	NA	NA
530	Border Terrier X Miniature Schnauzer	9.05	<0.1	NA	NA	NA	NA	NA	NA	NA	NA	NA
531	Border Terrier X Pomeranian	9.05	<0.1	NA	NA	NA	NA	NA	NA	NA	NA	NA
532	Border Terrier X Shih Tzu	9.05	<0.1	NA	NA	NA	NA	NA	NA	NA	NA	NA
533	Borzoi Cross/Type	9.05	<0.1	NA	NA	NA	NA	NA	NA	NA	NA	NA
534	Boxer X Pointer	9.05	<0.1	NA	NA	NA	NA	NA	NA	NA	NA	NA

535	Boxer X Retriever Golden	9.05	<0.1	NA	NA	NA	NA	NA	NA	NA	NA	NA
536	Boxer X Rhodesian Ridgeback	9.05	<0.1	NA	NA	NA	NA	NA	NA	NA	NA	NA
537	Boxer X Spaniel Cocker	9.05	<0.1	NA	NA	NA	NA	NA	NA	NA	NA	NA
538	Bulldog X Spanish Mastiff	9.05	<0.1	NA	NA	NA	NA	NA	NA	NA	NA	NA
539	Bullmastiff X Retriever Labrador	9.05	<0.1	NA	NA	NA	NA	NA	NA	NA	NA	NA
540	Bullmastiff X Siberian Husky	9.05	<0.1	NA	NA	NA	NA	NA	NA	NA	NA	NA
541	Castro Laboreiro Dog Cross/Type	9.05	<0.1	NA	NA	NA	NA	NA	NA	NA	NA	NA
542	Cavalier King Charles Spaniel X Spaniel English Springer	9.05	<0.1	NA	NA	NA	NA	NA	NA	NA	NA	NA
543	Chihuahua Smooth Coat X German Spitz Mittel	9.05	<0.1	NA	NA	NA	NA	NA	NA	NA	NA	NA
544	Chihuahua Smooth Coat X Russian Toy	9.05	<0.1	NA	NA	NA	NA	NA	NA	NA	NA	NA
545	Dalmatian X Jack Russell Terrier	9.05	<0.1	NA	NA	NA	NA	NA	NA	NA	NA	NA
546	Dutch Smoushond Cross/Type	9.05	<0.1	NA	NA	NA	NA	NA	NA	NA	NA	NA
547	German Shepherd Dog X Pointer	9.05	<0.1	NA	NA	NA	NA	NA	NA	NA	NA	NA
548	Great Dane X Rottweiler	9.05	<0.1	NA	NA	NA	NA	NA	NA	NA	NA	NA
549	Great Swiss Mountain Dog Cross/Type	9.05	<0.1	NA	NA	NA	NA	NA	NA	NA	NA	NA
550	Greyhound X Rottweiler	9.05	<0.1	NA	NA	NA	NA	NA	NA	NA	NA	NA
551	Hokkaido Cross/Type	9.05	<0.1	NA	NA	NA	NA	NA	NA	NA	NA	NA
552	Hungarian Vizsla X Staffordshire Bull Terrier	9.05	<0.1	NA	NA	NA	NA	NA	NA	NA	NA	NA

553	Italian Greyhound X Whippet	9.05	<0.1	NA	NA	NA	NA	NA	NA	NA	NA	NA
554	Italian Short Haired Segugio Cross/Type	9.05	<0.1	NA	NA	NA	NA	NA	NA	NA	NA	NA
555	Jack Russell Terrier X Miniature Schnauzer	9.05	<0.1	NA	NA	NA	NA	NA	NA	NA	NA	NA
556	Jack Russell Terrier X Norwich Terrier	9.05	<0.1	NA	NA	NA	NA	NA	NA	NA	NA	NA
557	Jack Russell Terrier X Pointer	9.05	<0.1	NA	NA	NA	NA	NA	NA	NA	NA	NA
558	Jack Russell Terrier X Scottish Terrier	9.05	<0.1	NA	NA	NA	NA	NA	NA	NA	NA	NA
559	Jack Russell Terrier X Tibetan Terrier	9.05	<0.1	NA	NA	NA	NA	NA	NA	NA	NA	NA
560	Japanese Spitz X Pomeranian	9.05	<0.1	NA	NA	NA	NA	NA	NA	NA	NA	NA
561	King Charles Spaniel X Lhasa Apso	9.05	<0.1	NA	NA	NA	NA	NA	NA	NA	NA	NA
562	King Charles Spaniel X Poodle	9.05	<0.1	NA	NA	NA	NA	NA	NA	NA	NA	NA
563	Lakeland Terrier X West Highland White Terrier	9.05	<0.1	NA	NA	NA	NA	NA	NA	NA	NA	NA
564	Lhasa Apso X Spaniel Cocker	9.05	<0.1	NA	NA	NA	NA	NA	NA	NA	NA	NA
565	Old English Sheepdog X Retriever Labrador	9.05	<0.1	NA	NA	NA	NA	NA	NA	NA	NA	NA
566	Papillon X Yorkshire Terrier	9.05	<0.1	NA	NA	NA	NA	NA	NA	NA	NA	NA
567	Pomeranian X Welsh Corgi Pembroke	9.05	<0.1	NA	NA	NA	NA	NA	NA	NA	NA	NA
568	Poodle X Retriever Flat Coated	9.05	<0.1	NA	NA	NA	NA	NA	NA	NA	NA	NA
569	Poodle X Soft Coated Wheaten Terrier	9.05	<0.1	NA	NA	NA	NA	NA	NA	NA	NA	NA
570	Poodle X St Bernard	9.05	<0.1	NA	NA	NA	NA	NA	NA	NA	NA	NA

571	Poodle X Whippet	9.05	<0.1	NA	NA	NA	NA	NA	NA	NA	NA	NA
572	Portuguese Sheepdog Cross/Type	9.05	<0.1	NA	NA	NA	NA	NA	NA	NA	NA	NA
573	Pug X Shar Pei	9.05	<0.1	NA	NA	NA	NA	NA	NA	NA	NA	NA
574	Pug X Spaniel Cocker	9.05	<0.1	NA	NA	NA	NA	NA	NA	NA	NA	NA
575	Pyrenean Sheepdog Smooth Faced Cross/Type	9.05	<0.1	NA	NA	NA	NA	NA	NA	NA	NA	NA
576	Retriever Flat Coated X Retriever Labrador	9.05	<0.1	NA	NA	NA	NA	NA	NA	NA	NA	NA
577	Retriever Golden X Spaniel English Springer	9.05	<0.1	NA	NA	NA	NA	NA	NA	NA	NA	NA
578	Retriever Labrador X Weimaraner	9.05	<0.1	NA	NA	NA	NA	NA	NA	NA	NA	NA
579	Schnauzer X Shih Tzu	9.05	<0.1	NA	NA	NA	NA	NA	NA	NA	NA	NA
580	Spaniel American Cocker X Spaniel English Springer	9.05	<0.1	NA	NA	NA	NA	NA	NA	NA	NA	NA
581	Staffordshire Bull Terrier X Welsh Corgi Pembroke	9.05	<0.1	NA	NA	NA	NA	NA	NA	NA	NA	NA
582	Xoloitzcuintle Cross/Type	9.05	<0.1	NA	NA	NA	NA	NA	NA	NA	NA	NA
583	Alaskan Malamute X Rottweiler	6.03	<0.1	NA	NA	NA	NA	NA	NA	NA	NA	NA
584	Alpine Dachsbracke Cross/Type	6.03	<0.1	NA	NA	NA	NA	NA	NA	NA	NA	NA
585	Atlas Mountain Dog Cross/Type	6.03	<0.1	NA	NA	NA	NA	NA	NA	NA	NA	NA
586	Australian Cattle Dog X Staffordshire Bull Terrier	6.03	<0.1	NA	NA	NA	NA	NA	NA	NA	NA	NA
587	Barbet Cross/Type	6.03	<0.1	NA	NA	NA	NA	NA	NA	NA	NA	NA

588	Basset Griffon Vendeen Grand Cross/Type	6.03	<0.1	NA	NA	NA	NA	NA	NA	NA	NA	NA
589	Basset Hound X Beagle	6.03	<0.1	NA	NA	NA	NA	NA	NA	NA	NA	NA
590	Basset Hound X Poodle	6.03	<0.1	NA	NA	NA	NA	NA	NA	NA	NA	NA
591	Basset Hound X Staffordshire Bull Terrier	6.03	<0.1	NA	NA	NA	NA	NA	NA	NA	NA	NA
592	Bavarian Mountain Hound Cross/Type	6.03	<0.1	NA	NA	NA	NA	NA	NA	NA	NA	NA
593	Beagle X Border Collie	6.03	<0.1	NA	NA	NA	NA	NA	NA	NA	NA	NA
594	Beagle X Shih Tzu	6.03	<0.1	NA	NA	NA	NA	NA	NA	NA	NA	NA
595	Bearded Collie X Greyhound	6.03	<0.1	NA	NA	NA	NA	NA	NA	NA	NA	NA
596	Belgian Shepherd Dog Laekenois Cross/Type	6.03	<0.1	NA	NA	NA	NA	NA	NA	NA	NA	NA
597	Belgian Shepherd Dog Malinois X Retriever Labrador	6.03	<0.1	NA	NA	NA	NA	NA	NA	NA	NA	NA
598	Bernese Mountain Dog X Poodle	6.03	<0.1	NA	NA	NA	NA	NA	NA	NA	NA	NA
599	Bichon Frise X Border Collie	6.03	<0.1	NA	NA	NA	NA	NA	NA	NA	NA	NA
600	Bichon Frise X Griffon Bruxellois	6.03	<0.1	NA	NA	NA	NA	NA	NA	NA	NA	NA
601	Bichon Frise X Miniature Pinscher	6.03	<0.1	NA	NA	NA	NA	NA	NA	NA	NA	NA
602	Bichon Frise X Retriever Labrador	6.03	<0.1	NA	NA	NA	NA	NA	NA	NA	NA	NA
603	Bichon Frise X Spaniel English Springer	6.03	<0.1	NA	NA	NA	NA	NA	NA	NA	NA	NA
604	Bichon Frise X Tibetan Terrier	6.03	<0.1	NA	NA	NA	NA	NA	NA	NA	NA	NA
605	Bloodhound X Retriever Labrador	6.03	<0.1	NA	NA	NA	NA	NA	NA	NA	NA	NA

606	Border Collie X Border Terrier	6.03	<0.1	NA	NA	NA	NA	NA	NA	NA	NA	NA
607	Border Collie X Collie Rough	6.03	<0.1	NA	NA	NA	NA	NA	NA	NA	NA	NA
608	Border Collie X Old English Sheepdog	6.03	<0.1	NA	NA	NA	NA	NA	NA	NA	NA	NA
609	Border Collie X Retriever Flat Coated	6.03	<0.1	NA	NA	NA	NA	NA	NA	NA	NA	NA
610	Border Collie X Shar Pei	6.03	<0.1	NA	NA	NA	NA	NA	NA	NA	NA	NA
611	Border Terrier X Chihuahua Smooth Coat	6.03	<0.1	NA	NA	NA	NA	NA	NA	NA	NA	NA
612	Boston Terrier X Chihuahua Smooth Coat	6.03	<0.1	NA	NA	NA	NA	NA	NA	NA	NA	NA
613	Boston Terrier X Jack Russell Terrier	6.03	<0.1	NA	NA	NA	NA	NA	NA	NA	NA	NA
614	Boxer X Dogue De Bordeaux	6.03	<0.1	NA	NA	NA	NA	NA	NA	NA	NA	NA
615	Boxer X Greyhound	6.03	<0.1	NA	NA	NA	NA	NA	NA	NA	NA	NA
616	Boxer X Shar Pei	6.03	<0.1	NA	NA	NA	NA	NA	NA	NA	NA	NA
617	Boxer X Siberian Husky	6.03	<0.1	NA	NA	NA	NA	NA	NA	NA	NA	NA
618	Boxer X Whippet	6.03	<0.1	NA	NA	NA	NA	NA	NA	NA	NA	NA
619	Bracco Italiano Cross/Type	6.03	<0.1	NA	NA	NA	NA	NA	NA	NA	NA	NA
620	Brazilian Terrier Cross/Type	6.03	<0.1	NA	NA	NA	NA	NA	NA	NA	NA	NA
621	Broholmer X Mastiff	6.03	<0.1	NA	NA	NA	NA	NA	NA	NA	NA	NA
622	Bull Terrier X Jack Russell Terrier	6.03	<0.1	NA	NA	NA	NA	NA	NA	NA	NA	NA
623	Bull Terrier X Rottweiler	6.03	<0.1	NA	NA	NA	NA	NA	NA	NA	NA	NA
624	Bulldog X Presa Canario	6.03	<0.1	NA	NA	NA	NA	NA	NA	NA	NA	NA
625	Bullmastiff X German Shepherd Dog	6.03	<0.1	NA	NA	NA	NA	NA	NA	NA	NA	NA
626	Cairn Terrier X Poodle	6.03	<0.1	NA	NA	NA	NA	NA	NA	NA	NA	NA
627	Cairn Terrier X Scottish Terrier	6.03	<0.1	NA	NA	NA	NA	NA	NA	NA	NA	NA

628	Cairn Terrier X Shih Tzu	6.03	<0.1	NA	NA	NA	NA	NA	NA	NA	NA	NA
629	Cavalier King Charles Spaniel X Dachshund Smooth Haired	6.03	<0.1	NA	NA	NA	NA	NA	NA	NA	NA	NA
630	Cavalier King Charles Spaniel X Maltese	6.03	<0.1	NA	NA	NA	NA	NA	NA	NA	NA	NA
631	Chihuahua Smooth Coat X Griffon Bruxellois	6.03	<0.1	NA	NA	NA	NA	NA	NA	NA	NA	NA
632	Chihuahua Smooth Coat X King Charles Spaniel	6.03	<0.1	NA	NA	NA	NA	NA	NA	NA	NA	NA
633	Chihuahua Smooth Coat X Lakeland Terrier	6.03	<0.1	NA	NA	NA	NA	NA	NA	NA	NA	NA
634	Chihuahua Smooth Coat X Welsh Corgi Pembroke	6.03	<0.1	NA	NA	NA	NA	NA	NA	NA	NA	NA
635	Chinese Crested X Lhasa Apso	6.03	<0.1	NA	NA	NA	NA	NA	NA	NA	NA	NA
636	Chinese Crested X Maltese	6.03	<0.1	NA	NA	NA	NA	NA	NA	NA	NA	NA
637	Chinese Crested X Poodle	6.03	<0.1	NA	NA	NA	NA	NA	NA	NA	NA	NA
638	Chow Chow X Jack Russell Terrier	6.03	<0.1	NA	NA	NA	NA	NA	NA	NA	NA	NA
639	Chow Chow X Siberian Husky	6.03	<0.1	NA	NA	NA	NA	NA	NA	NA	NA	NA
640	Dachshund Smooth Haired X French Bulldog	6.03	<0.1	NA	NA	NA	NA	NA	NA	NA	NA	NA
641	Dachshund Smooth Haired X Shih Tzu	6.03	<0.1	NA	NA	NA	NA	NA	NA	NA	NA	NA
642	Dachshund Smooth Haired X Spaniel Cocker	6.03	<0.1	NA	NA	NA	NA	NA	NA	NA	NA	NA
643	Dachshund Smooth Haired X Staffordshire Bull Terrier	6.03	<0.1	NA	NA	NA	NA	NA	NA	NA	NA	NA

644	Dachshund Smooth Haired X Welsh Corgi Pembroke	6.03	<0.1	NA	NA	NA	NA	NA	NA	NA	NA	NA
645	Dalmatian X Pointer	6.03	<0.1	NA	NA	NA	NA	NA	NA	NA	NA	NA
646	Dalmatian X Weimaraner	6.03	<0.1	NA	NA	NA	NA	NA	NA	NA	NA	NA
647	Dogue De Bordeaux X Rhodesian Ridgeback	6.03	<0.1	NA	NA	NA	NA	NA	NA	NA	NA	NA
648	English Setter X Retriever Golden	6.03	<0.1	NA	NA	NA	NA	NA	NA	NA	NA	NA
649	Estrela Mountain Dog Cross/Type	6.03	<0.1	NA	NA	NA	NA	NA	NA	NA	NA	NA
650	Eurasier Cross/Type	6.03	<0.1	NA	NA	NA	NA	NA	NA	NA	NA	NA
651	Finnish Lapphund X Samoyed	6.03	<0.1	NA	NA	NA	NA	NA	NA	NA	NA	NA
652	French Bulldog X Jack Russell Terrier	6.03	<0.1	NA	NA	NA	NA	NA	NA	NA	NA	NA
653	German Shepherd Dog X Pyrenean Mountain Dog	6.03	<0.1	NA	NA	NA	NA	NA	NA	NA	NA	NA
654	German Shepherd Dog X Saluki	6.03	<0.1	NA	NA	NA	NA	NA	NA	NA	NA	NA
655	German Spitz Klein Cross/Type	6.03	<0.1	NA	NA	NA	NA	NA	NA	NA	NA	NA
656	Griffon Bruxellois X Shih Tzu	6.03	<0.1	NA	NA	NA	NA	NA	NA	NA	NA	NA
657	Havanese X Poodle	6.03	<0.1	NA	NA	NA	NA	NA	NA	NA	NA	NA
658	Hovawart Cross/Type	6.03	<0.1	NA	NA	NA	NA	NA	NA	NA	NA	NA
659	Hungarian Pumi Cross/Type	6.03	<0.1	NA	NA	NA	NA	NA	NA	NA	NA	NA
660	Irish Red And White Setter X Retriever Labrador	6.03	<0.1	NA	NA	NA	NA	NA	NA	NA	NA	NA
661	Irish Setter X Poodle	6.03	<0.1	NA	NA	NA	NA	NA	NA	NA	NA	NA

662	Irish Setter X Retriever Labrador	6.03	<0.1	NA	NA	NA	NA	NA	NA	NA	NA	NA
663	Irish Setter X Spaniel Cocker	6.03	<0.1	NA	NA	NA	NA	NA	NA	NA	NA	NA
664	Istrian Short Haired Hound Cross/Type	6.03	<0.1	NA	NA	NA	NA	NA	NA	NA	NA	NA
665	Jack Russell Terrier X Manchester Terrier	6.03	<0.1	NA	NA	NA	NA	NA	NA	NA	NA	NA
666	Jack Russell Terrier X Papillon	6.03	<0.1	NA	NA	NA	NA	NA	NA	NA	NA	NA
667	Jack Russell Terrier X Pekingese	6.03	<0.1	NA	NA	NA	NA	NA	NA	NA	NA	NA
668	Jack Russell Terrier X Shetland Sheepdog	6.03	<0.1	NA	NA	NA	NA	NA	NA	NA	NA	NA
669	Jamthund Cross/Type	6.03	<0.1	NA	NA	NA	NA	NA	NA	NA	NA	NA
670	Japanese Akita Inu X Rottweiler	6.03	<0.1	NA	NA	NA	NA	NA	NA	NA	NA	NA
671	Japanese Akita Inu X Siberian Husky	6.03	<0.1	NA	NA	NA	NA	NA	NA	NA	NA	NA
672	Japanese Akita Inu X Staffordshire Bull Terrier	6.03	<0.1	NA	NA	NA	NA	NA	NA	NA	NA	NA
673	King Charles Spaniel X Maltese	6.03	<0.1	NA	NA	NA	NA	NA	NA	NA	NA	NA
674	Komondor Cross/Type	6.03	<0.1	NA	NA	NA	NA	NA	NA	NA	NA	NA
675	Lagotto Romagnolo Cross/Type	6.03	<0.1	NA	NA	NA	NA	NA	NA	NA	NA	NA
676	Maltese X Pug	6.03	<0.1	NA	NA	NA	NA	NA	NA	NA	NA	NA
677	Maltese X Spaniel Cocker	6.03	<0.1	NA	NA	NA	NA	NA	NA	NA	NA	NA
678	Mastiff X Poodle	6.03	<0.1	NA	NA	NA	NA	NA	NA	NA	NA	NA
679	Norfolk Terrier X Shih Tzu	6.03	<0.1	NA	NA	NA	NA	NA	NA	NA	NA	NA
680	Norfolk Terrier X West Highland White Terrier	6.03	<0.1	NA	NA	NA	NA	NA	NA	NA	NA	NA

681	Old English Sheepdog X St Bernard	6.03	<0.1	NA	NA	NA	NA	NA	NA	NA	NA	NA
682	Peruvian Hairless Dog Cross/Type	6.03	<0.1	NA	NA	NA	NA	NA	NA	NA	NA	NA
683	Picardy Spaniel Cross/Type	6.03	<0.1	NA	NA	NA	NA	NA	NA	NA	NA	NA
684	Pointer X Spaniel Cocker	6.03	<0.1	NA	NA	NA	NA	NA	NA	NA	NA	NA
685	Pointer X Staffordshire Bull Terrier	6.03	<0.1	NA	NA	NA	NA	NA	NA	NA	NA	NA
686	Polish Lowland Sheepdog Cross/Type	6.03	<0.1	NA	NA	NA	NA	NA	NA	NA	NA	NA
687	Pomeranian X Spaniel Cocker	6.03	<0.1	NA	NA	NA	NA	NA	NA	NA	NA	NA
688	Pomeranian X Staffordshire Bull Terrier	6.03	<0.1	NA	NA	NA	NA	NA	NA	NA	NA	NA
689	Poodle X Spaniel Clumber	6.03	<0.1	NA	NA	NA	NA	NA	NA	NA	NA	NA
690	Poodle X Tibetan Terrier	6.03	<0.1	NA	NA	NA	NA	NA	NA	NA	NA	NA
691	Pug X Retriever Labrador	6.03	<0.1	NA	NA	NA	NA	NA	NA	NA	NA	NA
692	Pug X West Highland White Terrier	6.03	<0.1	NA	NA	NA	NA	NA	NA	NA	NA	NA
693	Retriever Flat Coated X Retriever Golden	6.03	<0.1	NA	NA	NA	NA	NA	NA	NA	NA	NA
694	Retriever Flat Coated X Spaniel English Springer	6.03	<0.1	NA	NA	NA	NA	NA	NA	NA	NA	NA
695	Retriever Labrador X Spanish Water Dog	6.03	<0.1	NA	NA	NA	NA	NA	NA	NA	NA	NA
696	Schipperke Cross/Type	6.03	<0.1	NA	NA	NA	NA	NA	NA	NA	NA	NA
697	Shih Tzu X Spaniel English Springer	6.03	<0.1	NA	NA	NA	NA	NA	NA	NA	NA	NA
698	Shih Tzu X Staffordshire Bull Terrier	6.03	<0.1	NA	NA	NA	NA	NA	NA	NA	NA	NA

699	Shih Tzu X Tibetan Terrier	6.03	<0.1	NA	NA	NA	NA	NA	NA	NA	NA	NA
700	Shikoku Cross/Type	6.03	<0.1	NA	NA	NA	NA	NA	NA	NA	NA	NA
701	Spaniel American Water Cross/Type	6.03	<0.1	NA	NA	NA	NA	NA	NA	NA	NA	NA
702	Spaniel Cocker X West Highland White Terrier	6.03	<0.1	NA	NA	NA	NA	NA	NA	NA	NA	NA
703	Spaniel Cocker X Whippet	6.03	<0.1	NA	NA	NA	NA	NA	NA	NA	NA	NA
704	Spaniel Welsh Springer Cross/Type	6.03	<0.1	NA	NA	NA	NA	NA	NA	NA	NA	NA
705	Stabijhoun Cross/Type	6.03	<0.1	NA	NA	NA	NA	NA	NA	NA	NA	NA
706	Swedish Vallhund Cross/Type	6.03	<0.1	NA	NA	NA	NA	NA	NA	NA	NA	NA
707	Welsh Terrier Cross/Type	6.03	<0.1	NA	NA	NA	NA	NA	NA	NA	NA	NA
708	White Swiss Shepherd Dog Cross/Type	6.03	<0.1	NA	NA	NA	NA	NA	NA	NA	NA	NA
709	Afghan Hound X Spaniel Cocker	3.02	<0.1	NA	NA	NA	NA	NA	NA	NA	NA	NA
710	Airedale Terrier X Australian Shepherd	3.02	<0.1	NA	NA	NA	NA	NA	NA	NA	NA	NA
711	Airedale Terrier X German Shepherd Dog	3.02	<0.1	NA	NA	NA	NA	NA	NA	NA	NA	NA
712	Airedale Terrier X Retriever Labrador	3.02	<0.1	NA	NA	NA	NA	NA	NA	NA	NA	NA
713	Alaskan Malamute X American Akita	3.02	<0.1	NA	NA	NA	NA	NA	NA	NA	NA	NA
714	Alaskan Malamute X Dalmatian	3.02	<0.1	NA	NA	NA	NA	NA	NA	NA	NA	NA
715	Alaskan Malamute X Dobermann	3.02	<0.1	NA	NA	NA	NA	NA	NA	NA	NA	NA
716	Alaskan Malamute X Retriever Golden	3.02	<0.1	NA	NA	NA	NA	NA	NA	NA	NA	NA
717	Alaskan Malamute X	3.02	<0.1	NA	NA	NA	NA	NA	NA	NA	NA	NA

	Staffordshire Bull Terrier											
718	American Akita X Beagle	3.02	<0.1	NA	NA	NA	NA	NA	NA	NA	NA	NA
719	American Akita X Belgian Shepherd Dog	3.02	<0.1	NA	NA	NA	NA	NA	NA	NA	NA	NA
720	American Akita X Bullmastiff	3.02	<0.1	NA	NA	NA	NA	NA	NA	NA	NA	NA
721	American Akita X Jack Russell Terrier	3.02	<0.1	NA	NA	NA	NA	NA	NA	NA	NA	NA
722	American Akita X Rhodesian Ridgeback	3.02	<0.1	NA	NA	NA	NA	NA	NA	NA	NA	NA
723	American Akita X Samoyed	3.02	<0.1	NA	NA	NA	NA	NA	NA	NA	NA	NA
724	American Staffordshire Terrier X Bulldog	3.02	<0.1	NA	NA	NA	NA	NA	NA	NA	NA	NA
725	American Staffordshire Terrier X Shar Pei	3.02	<0.1	NA	NA	NA	NA	NA	NA	NA	NA	NA
726	Australian Cattle Dog X Chihuahua Smooth Coat	3.02	<0.1	NA	NA	NA	NA	NA	NA	NA	NA	NA
727	Australian Cattle Dog X German Shepherd Dog	3.02	<0.1	NA	NA	NA	NA	NA	NA	NA	NA	NA
728	Australian Cattle Dog X Yorkshire Terrier	3.02	<0.1	NA	NA	NA	NA	NA	NA	NA	NA	NA
729	Australian Kelpie X Weimaraner	3.02	<0.1	NA	NA	NA	NA	NA	NA	NA	NA	NA
730	Australian Kelpie X Welsh Corgi Pembroke	3.02	<0.1	NA	NA	NA	NA	NA	NA	NA	NA	NA
731	Australian Shepherd X Border Collie	3.02	<0.1	NA	NA	NA	NA	NA	NA	NA	NA	NA
732	Australian Shepherd X Boxer	3.02	<0.1	NA	NA	NA	NA	NA	NA	NA	NA	NA
733	Australian Shepherd X Poodle	3.02	<0.1	NA	NA	NA	NA	NA	NA	NA	NA	NA
734	Australian Silky Terrier X Yorkshire Terrier	3.02	<0.1	NA	NA	NA	NA	NA	NA	NA	NA	NA

735	Australian Terrier Cross/Type	3.02	<0.1	NA	NA	NA	NA	NA	NA	NA	NA	NA
736	Austrian Black And Tan Hound Cross/Type	3.02	<0.1	NA	NA	NA	NA	NA	NA	NA	NA	NA
737	Basenji X Whippet	3.02	<0.1	NA	NA	NA	NA	NA	NA	NA	NA	NA
738	Basset Bleu De Gascogne Cross/Type	3.02	<0.1	NA	NA	NA	NA	NA	NA	NA	NA	NA
739	Basset Hound X Border Collie	3.02	<0.1	NA	NA	NA	NA	NA	NA	NA	NA	NA
740	Basset Hound X Bulldog	3.02	<0.1	NA	NA	NA	NA	NA	NA	NA	NA	NA
741	Basset Hound X Cavalier King Charles Spaniel	3.02	<0.1	NA	NA	NA	NA	NA	NA	NA	NA	NA
742	Basset Hound X German Shepherd Dog	3.02	<0.1	NA	NA	NA	NA	NA	NA	NA	NA	NA
743	Basset Hound X Jack Russell Terrier	3.02	<0.1	NA	NA	NA	NA	NA	NA	NA	NA	NA
744	Basset Hound X Yorkshire Terrier	3.02	<0.1	NA	NA	NA	NA	NA	NA	NA	NA	NA
745	Beagle X Bichon Frise	3.02	<0.1	NA	NA	NA	NA	NA	NA	NA	NA	NA
746	Beagle X Boxer	3.02	<0.1	NA	NA	NA	NA	NA	NA	NA	NA	NA
747	Beagle X Cairn Terrier	3.02	<0.1	NA	NA	NA	NA	NA	NA	NA	NA	NA
748	Beagle X Chihuahua Smooth Coat	3.02	<0.1	NA	NA	NA	NA	NA	NA	NA	NA	NA
749	Beagle X Dachshund Smooth Haired	3.02	<0.1	NA	NA	NA	NA	NA	NA	NA	NA	NA
750	Beagle X Fox Terrier Wire	3.02	<0.1	NA	NA	NA	NA	NA	NA	NA	NA	NA
751	Beagle X German Shepherd Dog	3.02	<0.1	NA	NA	NA	NA	NA	NA	NA	NA	NA
752	Beagle X Parson Russell Terrier	3.02	<0.1	NA	NA	NA	NA	NA	NA	NA	NA	NA
753	Beagle X Pomeranian	3.02	<0.1	NA	NA	NA	NA	NA	NA	NA	NA	NA
754	Beagle X Spaniel Welsh Springer	3.02	<0.1	NA	NA	NA	NA	NA	NA	NA	NA	NA
755	Beagle X Weimaraner	3.02	<0.1	NA	NA	NA	NA	NA	NA	NA	NA	NA

756	Beagle X Whippet	3.02	<0.1	NA	NA	NA	NA	NA	NA	NA	NA	NA
757	Beagle X Yorkshire Terrier	3.02	<0.1	NA	NA	NA	NA	NA	NA	NA	NA	NA
758	Bearded Collie X Old English Sheepdog	3.02	<0.1	NA	NA	NA	NA	NA	NA	NA	NA	NA
759	Bearded Collie X Poodle	3.02	<0.1	NA	NA	NA	NA	NA	NA	NA	NA	NA
760	Bearded Collie X Spaniel Cocker	3.02	<0.1	NA	NA	NA	NA	NA	NA	NA	NA	NA
761	Bearded Collie X Spaniel English Springer	3.02	<0.1	NA	NA	NA	NA	NA	NA	NA	NA	NA
762	Bedlington Terrier X Border Collie	3.02	<0.1	NA	NA	NA	NA	NA	NA	NA	NA	NA
763	Bedlington Terrier X Italian Greyhound	3.02	<0.1	NA	NA	NA	NA	NA	NA	NA	NA	NA
764	Bedlington Terrier X Lakeland Terrier	3.02	<0.1	NA	NA	NA	NA	NA	NA	NA	NA	NA
765	Bedlington Terrier X Shih Tzu	3.02	<0.1	NA	NA	NA	NA	NA	NA	NA	NA	NA
766	Bedlington Terrier X West Highland White Terrier	3.02	<0.1	NA	NA	NA	NA	NA	NA	NA	NA	NA
767	Belgian Shepherd Dog Malinois X Border Collie	3.02	<0.1	NA	NA	NA	NA	NA	NA	NA	NA	NA
768	Belgian Shepherd Dog Malinois X Boxer	3.02	<0.1	NA	NA	NA	NA	NA	NA	NA	NA	NA
769	Belgian Shepherd Dog Malinois X Dutch Shepherd Dog	3.02	<0.1	NA	NA	NA	NA	NA	NA	NA	NA	NA
770	Belgian Shepherd Dog Malinois X Mastiff	3.02	<0.1	NA	NA	NA	NA	NA	NA	NA	NA	NA
771	Belgian Shepherd Dog Malinois X Staffordshire Bull Terrier	3.02	<0.1	NA	NA	NA	NA	NA	NA	NA	NA	NA

772	Bernese Mountain Dog X Border Collie	3.02	<0.1	NA	NA	NA	NA	NA	NA	NA	NA	NA
773	Bernese Mountain Dog X German Shepherd Dog	3.02	<0.1	NA	NA	NA	NA	NA	NA	NA	NA	NA
774	Bernese Mountain Dog X Retriever Labrador	3.02	<0.1	NA	NA	NA	NA	NA	NA	NA	NA	NA
775	Bichon Frise X Chinese Crested	3.02	<0.1	NA	NA	NA	NA	NA	NA	NA	NA	NA
776	Bichon Frise X Fox Terrier Wire	3.02	<0.1	NA	NA	NA	NA	NA	NA	NA	NA	NA
777	Bichon Frise X French Bulldog	3.02	<0.1	NA	NA	NA	NA	NA	NA	NA	NA	NA
778	Bichon Frise X King Charles Spaniel	3.02	<0.1	NA	NA	NA	NA	NA	NA	NA	NA	NA
779	Bichon Frise X Löwchen	3.02	<0.1	NA	NA	NA	NA	NA	NA	NA	NA	NA
780	Bichon Frise X Norfolk Terrier	3.02	<0.1	NA	NA	NA	NA	NA	NA	NA	NA	NA
781	Bichon Frise X Papillon	3.02	<0.1	NA	NA	NA	NA	NA	NA	NA	NA	NA
782	Bichon Frise X Samoyed	3.02	<0.1	NA	NA	NA	NA	NA	NA	NA	NA	NA
783	Bichon Frise X Scottish Terrier	3.02	<0.1	NA	NA	NA	NA	NA	NA	NA	NA	NA
784	Bichon Frise X Soft Coated Wheaten Terrier	3.02	<0.1	NA	NA	NA	NA	NA	NA	NA	NA	NA
785	Bohemian Wire Haired Pointing Griffon Cross/Type	3.02	<0.1	NA	NA	NA	NA	NA	NA	NA	NA	NA
786	Bohemian Wire Haired Pointing Griffon X Fox Terrier Wire	3.02	<0.1	NA	NA	NA	NA	NA	NA	NA	NA	NA
787	Bolognese X Poodle	3.02	<0.1	NA	NA	NA	NA	NA	NA	NA	NA	NA
788	Border Collie X Bull Terrier	3.02	<0.1	NA	NA	NA	NA	NA	NA	NA	NA	NA
789	Border Collie X Cavalier King Charles Spaniel	3.02	<0.1	NA	NA	NA	NA	NA	NA	NA	NA	NA
790	Border Collie X Chihuahua Smooth Coat	3.02	<0.1	NA	NA	NA	NA	NA	NA	NA	NA	NA

791	Border Collie X English Setter	3.02	<0.1	NA	NA	NA	NA	NA	NA	NA	NA	NA
792	Border Collie X German Hunting Terrier	3.02	<0.1	NA	NA	NA	NA	NA	NA	NA	NA	NA
793	Border Collie X German Wirehaired Pointer	3.02	<0.1	NA	NA	NA	NA	NA	NA	NA	NA	NA
794	Border Collie X Great Dane	3.02	<0.1	NA	NA	NA	NA	NA	NA	NA	NA	NA
795	Border Collie X Hungarian Puli	3.02	<0.1	NA	NA	NA	NA	NA	NA	NA	NA	NA
796	Border Collie X Irish Wolfhound	3.02	<0.1	NA	NA	NA	NA	NA	NA	NA	NA	NA
797	Border Collie X Newfoundland	3.02	<0.1	NA	NA	NA	NA	NA	NA	NA	NA	NA
798	Border Collie X Portuguese Water Dog	3.02	<0.1	NA	NA	NA	NA	NA	NA	NA	NA	NA
799	Border Collie X Rough Collie	3.02	<0.1	NA	NA	NA	NA	NA	NA	NA	NA	NA
800	Border Collie X Small Münsterländer	3.02	<0.1	NA	NA	NA	NA	NA	NA	NA	NA	NA
801	Border Collie X Tibetan Terrier	3.02	<0.1	NA	NA	NA	NA	NA	NA	NA	NA	NA
802	Border Collie X Wheaten Terrier	3.02	<0.1	NA	NA	NA	NA	NA	NA	NA	NA	NA
803	Border Terrier X French Bulldog	3.02	<0.1	NA	NA	NA	NA	NA	NA	NA	NA	NA
804	Border Terrier X Manchester Terrier	3.02	<0.1	NA	NA	NA	NA	NA	NA	NA	NA	NA
805	Border Terrier X Parson Russell Terrier	3.02	<0.1	NA	NA	NA	NA	NA	NA	NA	NA	NA
806	Border Terrier X Spaniel Cocker	3.02	<0.1	NA	NA	NA	NA	NA	NA	NA	NA	NA
807	Border Terrier X Staffordshire Bull Terrier	3.02	<0.1	NA	NA	NA	NA	NA	NA	NA	NA	NA
808	Border Terrier X Whippet	3.02	<0.1	NA	NA	NA	NA	NA	NA	NA	NA	NA
809	Boston Terrier X Cavalier King Charles Spaniel	3.02	<0.1	NA	NA	NA	NA	NA	NA	NA	NA	NA
810	Boston Terrier X Poodle	3.02	<0.1	NA	NA	NA	NA	NA	NA	NA	NA	NA
811	Boston Terrier X Shih Tzu	3.02	<0.1	NA	NA	NA	NA	NA	NA	NA	NA	NA

812	Bourbonnais Pointing Dog Cross/Type	3.02	<0.1	NA	NA	NA	NA	NA	NA	NA	NA	NA
813	Boxer X Dalmatian	3.02	<0.1	NA	NA	NA	NA	NA	NA	NA	NA	NA
814	Boxer X Hungarian Vizsla	3.02	<0.1	NA	NA	NA	NA	NA	NA	NA	NA	NA
815	Boxer X Poodle	3.02	<0.1	NA	NA	NA	NA	NA	NA	NA	NA	NA
816	Briquet Griffon Vendeen Cross/Type	3.02	<0.1	NA	NA	NA	NA	NA	NA	NA	NA	NA
817	Bull Terrier X Chihuahua Smooth Coat	3.02	<0.1	NA	NA	NA	NA	NA	NA	NA	NA	NA
818	Bull Terrier X German Shepherd Dog	3.02	<0.1	NA	NA	NA	NA	NA	NA	NA	NA	NA
819	Bull Terrier X Pomeranian	3.02	<0.1	NA	NA	NA	NA	NA	NA	NA	NA	NA
820	Bull Terrier X Retriever Labrador	3.02	<0.1	NA	NA	NA	NA	NA	NA	NA	NA	NA
821	Bull Terrier X Rhodesian Ridgeback	3.02	<0.1	NA	NA	NA	NA	NA	NA	NA	NA	NA
822	Bull Terrier X Spaniel English Springer	3.02	<0.1	NA	NA	NA	NA	NA	NA	NA	NA	NA
823	Bulldog X Great Dane	3.02	<0.1	NA	NA	NA	NA	NA	NA	NA	NA	NA
824	Bulldog X Greyhound	3.02	<0.1	NA	NA	NA	NA	NA	NA	NA	NA	NA
825	Bulldog X Neapolitan Mastiff	3.02	<0.1	NA	NA	NA	NA	NA	NA	NA	NA	NA
826	Bulldog X Pomeranian	3.02	<0.1	NA	NA	NA	NA	NA	NA	NA	NA	NA
827	Bulldog X Rhodesian Ridgeback	3.02	<0.1	NA	NA	NA	NA	NA	NA	NA	NA	NA
828	Bullmastiff X French Bulldog	3.02	<0.1	NA	NA	NA	NA	NA	NA	NA	NA	NA
829	Bullmastiff X Greyhound	3.02	<0.1	NA	NA	NA	NA	NA	NA	NA	NA	NA
830	Bullmastiff X Shar Pei	3.02	<0.1	NA	NA	NA	NA	NA	NA	NA	NA	NA
831	Bullmastiff X Spaniel English Springer	3.02	<0.1	NA	NA	NA	NA	NA	NA	NA	NA	NA

832	Burgos Pointing Dog Cross/Type	3.02	<0.1	NA	NA	NA	NA	NA	NA	NA	NA	NA
833	Cairn Terrier X Kerry Blue Terrier	3.02	<0.1	NA	NA	NA	NA	NA	NA	NA	NA	NA
834	Cairn Terrier X Lakeland Terrier	3.02	<0.1	NA	NA	NA	NA	NA	NA	NA	NA	NA
835	Cairn Terrier X Lhasa Apso	3.02	<0.1	NA	NA	NA	NA	NA	NA	NA	NA	NA
836	Cairn Terrier X Miniature Schnauzer	3.02	<0.1	NA	NA	NA	NA	NA	NA	NA	NA	NA
837	Cairn Terrier X Spaniel American Cocker	3.02	<0.1	NA	NA	NA	NA	NA	NA	NA	NA	NA
838	Canarian Warren Hound X Retriever Golden	3.02	<0.1	NA	NA	NA	NA	NA	NA	NA	NA	NA
839	Canarian Warren Hound X Retriever Labrador	3.02	<0.1	NA	NA	NA	NA	NA	NA	NA	NA	NA
840	Cavalier King Charles Spaniel X Dachshund Miniature Smooth Haired	3.02	<0.1	NA	NA	NA	NA	NA	NA	NA	NA	NA
841	Cavalier King Charles Spaniel X Miniature Schnauzer	3.02	<0.1	NA	NA	NA	NA	NA	NA	NA	NA	NA
842	Cavalier King Charles Spaniel X Norfolk Terrier	3.02	<0.1	NA	NA	NA	NA	NA	NA	NA	NA	NA
843	Cavalier King Charles Spaniel X Pekingese	3.02	<0.1	NA	NA	NA	NA	NA	NA	NA	NA	NA
844	Cavalier King Charles Spaniel X Schnauzer	3.02	<0.1	NA	NA	NA	NA	NA	NA	NA	NA	NA
845	Cavalier King Charles Spaniel X Welsh Corgi Pembroke	3.02	<0.1	NA	NA	NA	NA	NA	NA	NA	NA	NA
846	Chihuahua Smooth Coat X Cavalier King Charles Spaniel	3.02	<0.1	NA	NA	NA	NA	NA	NA	NA	NA	NA

847	Chihuahua Smooth Coat X Greyhound	3.02	<0.1	NA	NA	NA	NA	NA	NA	NA	NA	NA
848	Chihuahua Smooth Coat X Italian Greyhound	3.02	<0.1	NA	NA	NA	NA	NA	NA	NA	NA	NA
849	Chihuahua Smooth Coat X Japanese Shiba Inu	3.02	<0.1	NA	NA	NA	NA	NA	NA	NA	NA	NA
850	Chihuahua Smooth Coat X Norwich Terrier	3.02	<0.1	NA	NA	NA	NA	NA	NA	NA	NA	NA
851	Chihuahua Smooth Coat X Ret. Labrador	3.02	<0.1	NA	NA	NA	NA	NA	NA	NA	NA	NA
852	Chihuahua Smooth Coat X Spaniel Cocker	3.02	<0.1	NA	NA	NA	NA	NA	NA	NA	NA	NA
853	Chihuahua Smooth Coat X Tibetan Spaniel	3.02	<0.1	NA	NA	NA	NA	NA	NA	NA	NA	NA
854	Chihuahua Smooth Coat X Whippet	3.02	<0.1	NA	NA	NA	NA	NA	NA	NA	NA	NA
855	Chinese Crested X German Shepherd Dog	3.02	<0.1	NA	NA	NA	NA	NA	NA	NA	NA	NA
856	Chinese Crested X Jack Russell Terrier	3.02	<0.1	NA	NA	NA	NA	NA	NA	NA	NA	NA
857	Chinese Crested X Pug	3.02	<0.1	NA	NA	NA	NA	NA	NA	NA	NA	NA
858	Chinese Crested X Shih Tzu	3.02	<0.1	NA	NA	NA	NA	NA	NA	NA	NA	NA
859	Chinese Crested X Tibetan Spaniel	3.02	<0.1	NA	NA	NA	NA	NA	NA	NA	NA	NA
860	Chinese Crested X Yorkshire Terrier	3.02	<0.1	NA	NA	NA	NA	NA	NA	NA	NA	NA
861	Chow Chow X German Shepherd Dog	3.02	<0.1	NA	NA	NA	NA	NA	NA	NA	NA	NA
862	Chow Chow X Retriever Labrador	3.02	<0.1	NA	NA	NA	NA	NA	NA	NA	NA	NA
863	Chow Chow X Staffordshire Bull Terrier	3.02	<0.1	NA	NA	NA	NA	NA	NA	NA	NA	NA

864	Collie Rough X Hungarian Puli	3.02	<0.1	NA	NA	NA	NA	NA	NA	NA	NA	NA
865	Coton De Tulear X Lhasa Apso	3.02	<0.1	NA	NA	NA	NA	NA	NA	NA	NA	NA
866	Coton De Tulear X Shih Tzu	3.02	<0.1	NA	NA	NA	NA	NA	NA	NA	NA	NA
867	Dachshund Long Haired X Dachshund Smooth Haired	3.02	<0.1	NA	NA	NA	NA	NA	NA	NA	NA	NA
868	Dachshund Miniature Smooth Haired X Miniature Pinscher	3.02	<0.1	NA	NA	NA	NA	NA	NA	NA	NA	NA
869	Dachshund Smooth Haired X Miniature Pinscher	3.02	<0.1	NA	NA	NA	NA	NA	NA	NA	NA	NA
870	Dachshund Smooth Haired X Pomeranian	3.02	<0.1	NA	NA	NA	NA	NA	NA	NA	NA	NA
871	Dachshund Smooth Haired X Pug	3.02	<0.1	NA	NA	NA	NA	NA	NA	NA	NA	NA
872	Dachshund Smooth Haired X Schnauzer	3.02	<0.1	NA	NA	NA	NA	NA	NA	NA	NA	NA
873	Dachshund Smooth Haired X Shetland Sheepdog	3.02	<0.1	NA	NA	NA	NA	NA	NA	NA	NA	NA
874	Dalmatian X Dobermann	3.02	<0.1	NA	NA	NA	NA	NA	NA	NA	NA	NA
875	Dalmatian X Newfoundland	3.02	<0.1	NA	NA	NA	NA	NA	NA	NA	NA	NA
876	Dalmatian X Rottweiler	3.02	<0.1	NA	NA	NA	NA	NA	NA	NA	NA	NA
877	Dalmatian X Spaniel English Springer	3.02	<0.1	NA	NA	NA	NA	NA	NA	NA	NA	NA
878	Deerhound X Saluki	3.02	<0.1	NA	NA	NA	NA	NA	NA	NA	NA	NA
879	Deerhound X Whippet	3.02	<0.1	NA	NA	NA	NA	NA	NA	NA	NA	NA
880	Dobermann X Giant Schnauzer	3.02	<0.1	NA	NA	NA	NA	NA	NA	NA	NA	NA
881	Dobermann X Jack Russell Terrier	3.02	<0.1	NA	NA	NA	NA	NA	NA	NA	NA	NA

882	Dobermann X Pointer	3.02	<0.1	NA	NA	NA	NA	NA	NA	NA	NA	NA
883	Dobermann X Retriever Golden	3.02	<0.1	NA	NA	NA	NA	NA	NA	NA	NA	NA
884	Dobermann X Siberian Husky	3.02	<0.1	NA	NA	NA	NA	NA	NA	NA	NA	NA
885	Dobermann X Weimaraner	3.02	<0.1	NA	NA	NA	NA	NA	NA	NA	NA	NA
886	Dogue De Bordeaux X German Shepherd Dog	3.02	<0.1	NA	NA	NA	NA	NA	NA	NA	NA	NA
887	Dogue De Bordeaux X Greyhound	3.02	<0.1	NA	NA	NA	NA	NA	NA	NA	NA	NA
888	Dutch Schapendoes Cross/Type	3.02	<0.1	NA	NA	NA	NA	NA	NA	NA	NA	NA
889	English Setter X Gordon Setter	3.02	<0.1	NA	NA	NA	NA	NA	NA	NA	NA	NA
890	English Setter X Pointer	3.02	<0.1	NA	NA	NA	NA	NA	NA	NA	NA	NA
891	English Setter X Poodle	3.02	<0.1	NA	NA	NA	NA	NA	NA	NA	NA	NA
892	Entlebucher Mountain Dog Cross/Type	3.02	<0.1	NA	NA	NA	NA	NA	NA	NA	NA	NA
893	Fila Brasileiro Cross/Type	3.02	<0.1	NA	NA	NA	NA	NA	NA	NA	NA	NA
894	Finnish Hound Cross/Type	3.02	<0.1	NA	NA	NA	NA	NA	NA	NA	NA	NA
895	Finnish Lapphund Cross/Type	3.02	<0.1	NA	NA	NA	NA	NA	NA	NA	NA	NA
896	Fox Terrier Wire X Miniature Schnauzer	3.02	<0.1	NA	NA	NA	NA	NA	NA	NA	NA	NA
897	Fox Terrier Wire X Scottish Terrier	3.02	<0.1	NA	NA	NA	NA	NA	NA	NA	NA	NA
898	Fox Terrier Wire X Welsh Corgi Pembroke	3.02	<0.1	NA	NA	NA	NA	NA	NA	NA	NA	NA
899	Foxhound X Jack Russell Terrier	3.02	<0.1	NA	NA	NA	NA	NA	NA	NA	NA	NA
900	Foxhound X Papillon	3.02	<0.1	NA	NA	NA	NA	NA	NA	NA	NA	NA
901	Foxhound X St Bernard	3.02	<0.1	NA	NA	NA	NA	NA	NA	NA	NA	NA

902	French Bulldog X Mastiff	3.02	<0.1	NA	NA	NA	NA	NA	NA	NA	NA	NA
903	French Bulldog X Neapolitan Mastiff	3.02	<0.1	NA	NA	NA	NA	NA	NA	NA	NA	NA
904	French Bulldog X Pomeranian	3.02	<0.1	NA	NA	NA	NA	NA	NA	NA	NA	NA
905	French Bulldog X Shar Pei	3.02	<0.1	NA	NA	NA	NA	NA	NA	NA	NA	NA
906	French Bulldog X Spaniel Cocker	3.02	<0.1	NA	NA	NA	NA	NA	NA	NA	NA	NA
907	French White And Black Hound Cross/Type	3.02	<0.1	NA	NA	NA	NA	NA	NA	NA	NA	NA
908	Frisian Water Dog Cross/Type	3.02	<0.1	NA	NA	NA	NA	NA	NA	NA	NA	NA
909	German Hunting Terrier Cross/Type	3.02	<0.1	NA	NA	NA	NA	NA	NA	NA	NA	NA
910	German Shepherd Dog X Giant Schnauzer	3.02	<0.1	NA	NA	NA	NA	NA	NA	NA	NA	NA
911	German Shepherd Dog X Large Münsterländer	3.02	<0.1	NA	NA	NA	NA	NA	NA	NA	NA	NA
912	German Shepherd Dog X Leonberger	3.02	<0.1	NA	NA	NA	NA	NA	NA	NA	NA	NA
913	German Shepherd Dog X Pug	3.02	<0.1	NA	NA	NA	NA	NA	NA	NA	NA	NA
914	German Shepherd Dog X Retriever Flat Coated	3.02	<0.1	NA	NA	NA	NA	NA	NA	NA	NA	NA
915	German Shepherd Dog X Shar Pei	3.02	<0.1	NA	NA	NA	NA	NA	NA	NA	NA	NA
916	German Shepherd Dog X Spaniel Cocker	3.02	<0.1	NA	NA	NA	NA	NA	NA	NA	NA	NA
917	German Shepherd Dog X St Bernard	3.02	<0.1	NA	NA	NA	NA	NA	NA	NA	NA	NA
918	German Shepherd Dog X Weimaraner	3.02	<0.1	NA	NA	NA	NA	NA	NA	NA	NA	NA

919	German Shepherd Dog X Welsh Corgi Pembroke	3.02	<0.1	NA	NA	NA	NA	NA	NA	NA	NA	NA
920	German Shepherd Dog X Whippet	3.02	<0.1	NA	NA	NA	NA	NA	NA	NA	NA	NA
921	German Shorthaired Pointer X Ret. Labrador	3.02	<0.1	NA	NA	NA	NA	NA	NA	NA	NA	NA
922	German Shorthaired Pointer X Spaniel English Springer	3.02	<0.1	NA	NA	NA	NA	NA	NA	NA	NA	NA
923	German Shorthaired Pointer X Staffordshire Bull Terrier	3.02	<0.1	NA	NA	NA	NA	NA	NA	NA	NA	NA
924	German Spitz Giant Cross/Type	3.02	<0.1	NA	NA	NA	NA	NA	NA	NA	NA	NA
925	German Spitz Mittel X Poodle	3.02	<0.1	NA	NA	NA	NA	NA	NA	NA	NA	NA
926	German Wirehaired Pointer X Weimaraner	3.02	<0.1	NA	NA	NA	NA	NA	NA	NA	NA	NA
927	Glen Of Imaal Terrier X Shih Tzu	3.02	<0.1	NA	NA	NA	NA	NA	NA	NA	NA	NA
928	Great Dane X Siberian Husky	3.02	<0.1	NA	NA	NA	NA	NA	NA	NA	NA	NA
929	Great Dane X Staffordshire Bull Terrier	3.02	<0.1	NA	NA	NA	NA	NA	NA	NA	NA	NA
930	Great Dane X Yorkshire Terrier	3.02	<0.1	NA	NA	NA	NA	NA	NA	NA	NA	NA
931	Great Swiss Mountain Dog X Italian Short Haired Segugio	3.02	<0.1	NA	NA	NA	NA	NA	NA	NA	NA	NA
932	Greyhound X Irish Wolfhound	3.02	<0.1	NA	NA	NA	NA	NA	NA	NA	NA	NA
933	Greyhound X Siberian Husky	3.02	<0.1	NA	NA	NA	NA	NA	NA	NA	NA	NA
934	Griffon Bruxellois X Pomeranian	3.02	<0.1	NA	NA	NA	NA	NA	NA	NA	NA	NA

935	Griffon Bruxellois X Poodle	3.02	<0.1	NA	NA	NA	NA	NA	NA	NA	NA	NA
936	Griffon Bruxellois X Spaniel Cocker	3.02	<0.1	NA	NA	NA	NA	NA	NA	NA	NA	NA
937	Hamiltonstovare Cross/Type	3.02	<0.1	NA	NA	NA	NA	NA	NA	NA	NA	NA
938	Hanoverian Scent Hound Cross/Type	3.02	<0.1	NA	NA	NA	NA	NA	NA	NA	NA	NA
939	Havanese X Maltese	3.02	<0.1	NA	NA	NA	NA	NA	NA	NA	NA	NA
940	Havanese X Yorkshire Terrier	3.02	<0.1	NA	NA	NA	NA	NA	NA	NA	NA	NA
941	Hungarian Pumi X Mudi	3.02	<0.1	NA	NA	NA	NA	NA	NA	NA	NA	NA
942	Hungarian Vizsla X Pointer	3.02	<0.1	NA	NA	NA	NA	NA	NA	NA	NA	NA
943	Hungarian Vizsla X Rhodesian Ridgeback	3.02	<0.1	NA	NA	NA	NA	NA	NA	NA	NA	NA
944	Hungarian Vizsla X Spaniel Cocker	3.02	<0.1	NA	NA	NA	NA	NA	NA	NA	NA	NA
945	Hungarian Vizsla X Weimaraner	3.02	<0.1	NA	NA	NA	NA	NA	NA	NA	NA	NA
946	Irish Setter X Siberian Husky	3.02	<0.1	NA	NA	NA	NA	NA	NA	NA	NA	NA
947	Irish Terrier X Parson Russell Terrier	3.02	<0.1	NA	NA	NA	NA	NA	NA	NA	NA	NA
948	Irish Terrier X Weimaraner	3.02	<0.1	NA	NA	NA	NA	NA	NA	NA	NA	NA
949	Irish Wolfhound X Ret. Labrador	3.02	<0.1	NA	NA	NA	NA	NA	NA	NA	NA	NA
950	Irish Wolfhound X Rottweiler	3.02	<0.1	NA	NA	NA	NA	NA	NA	NA	NA	NA
951	Italian Cane Corso Cross/Type	3.02	<0.1	NA	NA	NA	NA	NA	NA	NA	NA	NA
952	Italian Cane Corso X Neapolitan Mastiff	3.02	<0.1	NA	NA	NA	NA	NA	NA	NA	NA	NA
953	Italian Greyhound X Jack Russell Terrier	3.02	<0.1	NA	NA	NA	NA	NA	NA	NA	NA	NA

954	Italian Greyhound X Pointer	3.02	<0.1	NA	NA	NA	NA	NA	NA	NA	NA	NA
955	Italian Greyhound X Poodle	3.02	<0.1	NA	NA	NA	NA	NA	NA	NA	NA	NA
956	Italian Volpino Cross/Type	3.02	<0.1	NA	NA	NA	NA	NA	NA	NA	NA	NA
957	Jack Russell Terrier X Miniature Pinscher	3.02	<0.1	NA	NA	NA	NA	NA	NA	NA	NA	NA
958	Jack Russell Terrier X Saluki	3.02	<0.1	NA	NA	NA	NA	NA	NA	NA	NA	NA
959	Jack Russell Terrier X Shar Pei	3.02	<0.1	NA	NA	NA	NA	NA	NA	NA	NA	NA
960	Jack Russell Terrier X Stabijhoun	3.02	<0.1	NA	NA	NA	NA	NA	NA	NA	NA	NA
961	Japanese Akita Inu X Rhodesian Ridgeback	3.02	<0.1	NA	NA	NA	NA	NA	NA	NA	NA	NA
962	Japanese Akita Inu X St Bernard	3.02	<0.1	NA	NA	NA	NA	NA	NA	NA	NA	NA
963	Japanese Shiba Inu X Pomeranian	3.02	<0.1	NA	NA	NA	NA	NA	NA	NA	NA	NA
964	Japanese Spitz X Shetland Sheepdog	3.02	<0.1	NA	NA	NA	NA	NA	NA	NA	NA	NA
965	Japanese Spitz X Welsh Corgi Pembroke	3.02	<0.1	NA	NA	NA	NA	NA	NA	NA	NA	NA
966	Kai Cross/Type	3.02	<0.1	NA	NA	NA	NA	NA	NA	NA	NA	NA
967	Karst Shepherd Dog Cross/Type	3.02	<0.1	NA	NA	NA	NA	NA	NA	NA	NA	NA
968	King Charles Spaniel X Miniature Schnauzer	3.02	<0.1	NA	NA	NA	NA	NA	NA	NA	NA	NA
969	King Charles Spaniel X Papillon	3.02	<0.1	NA	NA	NA	NA	NA	NA	NA	NA	NA
970	King Charles Spaniel X Pomeranian	3.02	<0.1	NA	NA	NA	NA	NA	NA	NA	NA	NA
971	King Charles Spaniel X Ret. Labrador	3.02	<0.1	NA	NA	NA	NA	NA	NA	NA	NA	NA

972	King Charles Spaniel X Staffordshire Bull Terrier	3.02	<0.1	NA	NA	NA	NA	NA	NA	NA	NA	NA
973	King Charles Spaniel X Welsh Corgi Pembroke	3.02	<0.1	NA	NA	NA	NA	NA	NA	NA	NA	NA
974	King Charles Spaniel X West Highland White Terrier	3.02	<0.1	NA	NA	NA	NA	NA	NA	NA	NA	NA
975	King Charles Spaniel X Yorkshire Terrier	3.02	<0.1	NA	NA	NA	NA	NA	NA	NA	NA	NA
976	Korean Jindo Cross/Type	3.02	<0.1	NA	NA	NA	NA	NA	NA	NA	NA	NA
977	Lakeland Terrier X Pug	3.02	<0.1	NA	NA	NA	NA	NA	NA	NA	NA	NA
978	Lakeland Terrier X Shih Tzu	3.02	<0.1	NA	NA	NA	NA	NA	NA	NA	NA	NA
979	Landseer Cross/Type	3.02	<0.1	NA	NA	NA	NA	NA	NA	NA	NA	NA
980	Lapponian Herder Cross/Type	3.02	<0.1	NA	NA	NA	NA	NA	NA	NA	NA	NA
981	Leonberger X Newfoundland	3.02	<0.1	NA	NA	NA	NA	NA	NA	NA	NA	NA
982	Lhasa Apso X Ret. Labrador	3.02	<0.1	NA	NA	NA	NA	NA	NA	NA	NA	NA
983	Lhasa Apso X Staffordshire Bull Terrier	3.02	<0.1	NA	NA	NA	NA	NA	NA	NA	NA	NA
984	Lhasa Apso X Tibetan Terrier	3.02	<0.1	NA	NA	NA	NA	NA	NA	NA	NA	NA
985	Maremma Sheepdog X Norwegian Elkhound	3.02	<0.1	NA	NA	NA	NA	NA	NA	NA	NA	NA
986	Mastiff X Neapolitan Mastiff	3.02	<0.1	NA	NA	NA	NA	NA	NA	NA	NA	NA
987	Mastiff X Newfoundland	3.02	<0.1	NA	NA	NA	NA	NA	NA	NA	NA	NA
988	Mastiff X Rafeiro do Alentejo	3.02	<0.1	NA	NA	NA	NA	NA	NA	NA	NA	NA
989	Miniature Pinscher X Pomeranian	3.02	<0.1	NA	NA	NA	NA	NA	NA	NA	NA	NA
990	Miniature Pinscher X Poodle	3.02	<0.1	NA	NA	NA	NA	NA	NA	NA	NA	NA

991	Miniature Schnauzer X Scottish Terrier	3.02	<0.1	NA	NA	NA	NA	NA	NA	NA	NA	NA
992	Miniature Schnauzer X Shih Tzu	3.02	<0.1	NA	NA	NA	NA	NA	NA	NA	NA	NA
993	Neapolitan Mastiff X Rhodesian Ridgeback	3.02	<0.1	NA	NA	NA	NA	NA	NA	NA	NA	NA
994	Newfoundland X Old English Sheepdog	3.02	<0.1	NA	NA	NA	NA	NA	NA	NA	NA	NA
995	Newfoundland X Poodle	3.02	<0.1	NA	NA	NA	NA	NA	NA	NA	NA	NA
996	Newfoundland X Ret. Labrador	3.02	<0.1	NA	NA	NA	NA	NA	NA	NA	NA	NA
997	Newfoundland X Rottweiler	3.02	<0.1	NA	NA	NA	NA	NA	NA	NA	NA	NA
998	Newfoundland X St Bernard	3.02	<0.1	NA	NA	NA	NA	NA	NA	NA	NA	NA
999	Newfoundland X Staffordshire Bull Terrier	3.02	<0.1	NA	NA	NA	NA	NA	NA	NA	NA	NA
1000	Norfolk Terrier X Staffordshire Bull Terrier	3.02	<0.1	NA	NA	NA	NA	NA	NA	NA	NA	NA
1001	Norwegian Buhund Cross/Type	3.02	<0.1	NA	NA	NA	NA	NA	NA	NA	NA	NA
1002	Norwich Terrier X Yorkshire Terrier	3.02	<0.1	NA	NA	NA	NA	NA	NA	NA	NA	NA
1003	Old English Sheepdog X Poodle	3.02	<0.1	NA	NA	NA	NA	NA	NA	NA	NA	NA
1004	Papillon X West Highland White Terrier	3.02	<0.1	NA	NA	NA	NA	NA	NA	NA	NA	NA
1005	Parson Russell Terrier X Pomeranian	3.02	<0.1	NA	NA	NA	NA	NA	NA	NA	NA	NA
1006	Parson Russell Terrier X Pug	3.02	<0.1	NA	NA	NA	NA	NA	NA	NA	NA	NA
1007	Pekingese X West Highland White Terrier	3.02	<0.1	NA	NA	NA	NA	NA	NA	NA	NA	NA
1008	Pointer X Retriever Golden	3.02	<0.1	NA	NA	NA	NA	NA	NA	NA	NA	NA

1009	Pomeranian X Ret. Labrador	3.02	<0.1	NA	NA	NA	NA	NA	NA	NA	NA	NA
1010	Pont Audemer Spaniel Cross/Type	3.02	<0.1	NA	NA	NA	NA	NA	NA	NA	NA	NA
1011	Poodle X Retriever Nova Scotia Duck Tolling	3.02	<0.1	NA	NA	NA	NA	NA	NA	NA	NA	NA
1012	Poodle X Russian Black Terrier	3.02	<0.1	NA	NA	NA	NA	NA	NA	NA	NA	NA
1013	Poodle X Samoyed	3.02	<0.1	NA	NA	NA	NA	NA	NA	NA	NA	NA
1014	Poodle X Scottish Terrier	3.02	<0.1	NA	NA	NA	NA	NA	NA	NA	NA	NA
1015	Poodle X Siberian Husky	3.02	<0.1	NA	NA	NA	NA	NA	NA	NA	NA	NA
1016	Poodle X Staffordshire Bull Terrier	3.02	<0.1	NA	NA	NA	NA	NA	NA	NA	NA	NA
1017	Poodle X Weimaraner	3.02	<0.1	NA	NA	NA	NA	NA	NA	NA	NA	NA
1018	Porcelaine Cross/Type	3.02	<0.1	NA	NA	NA	NA	NA	NA	NA	NA	NA
1019	Portuguese Podengo X Ret. Labrador	3.02	<0.1	NA	NA	NA	NA	NA	NA	NA	NA	NA
1020	Presa Canario X Ret. Labrador	3.02	<0.1	NA	NA	NA	NA	NA	NA	NA	NA	NA
1021	Presa Canario X Rhodesian Ridgeback	3.02	<0.1	NA	NA	NA	NA	NA	NA	NA	NA	NA
1022	Pug X Spaniel English Springer	3.02	<0.1	NA	NA	NA	NA	NA	NA	NA	NA	NA
1023	Pyrenean Mastiff Cross/Type	3.02	<0.1	NA	NA	NA	NA	NA	NA	NA	NA	NA
1024	Rafeiro do Alentejo Cross/Type	3.02	<0.1	NA	NA	NA	NA	NA	NA	NA	NA	NA
1025	Ret. Labrador X Retriever Nova Scotia Duck Tolling	3.02	<0.1	NA	NA	NA	NA	NA	NA	NA	NA	NA
1026	Ret. Labrador X Shih Tzu	3.02	<0.1	NA	NA	NA	NA	NA	NA	NA	NA	NA
1027	Ret. Labrador X Welsh Corgi Pembroke	3.02	<0.1	NA	NA	NA	NA	NA	NA	NA	NA	NA

1028	Ret. Labrador X West Highland White Terrier	3.02	<0.1	NA	NA	NA	NA	NA	NA	NA	NA	NA
1029	Retriever Golden X Rhodesian Ridgeback	3.02	<0.1	NA	NA	NA	NA	NA	NA	NA	NA	NA
1030	Retriever Golden X Siberian Husky	3.02	<0.1	NA	NA	NA	NA	NA	NA	NA	NA	NA
1031	Retriever Golden X Spaniel Irish Water	3.02	<0.1	NA	NA	NA	NA	NA	NA	NA	NA	NA
1032	Rhodesian Ridgeback X Shar Pei	3.02	<0.1	NA	NA	NA	NA	NA	NA	NA	NA	NA
1033	Rhodesian Ridgeback X Weimaraner	3.02	<0.1	NA	NA	NA	NA	NA	NA	NA	NA	NA
1034	Romanian Mioritic Shepherd Dog Cross/Type	3.02	<0.1	NA	NA	NA	NA	NA	NA	NA	NA	NA
1035	Rottweiler X Spaniel Cocker	3.02	<0.1	NA	NA	NA	NA	NA	NA	NA	NA	NA
1036	Russian European Laika Cross/Type	3.02	<0.1	NA	NA	NA	NA	NA	NA	NA	NA	NA
1037	Samoyed X Siberian Husky	3.02	<0.1	NA	NA	NA	NA	NA	NA	NA	NA	NA
1038	Samoyed X Spaniel Cocker	3.02	<0.1	NA	NA	NA	NA	NA	NA	NA	NA	NA
1039	Schnauzer X Yorkshire Terrier	3.02	<0.1	NA	NA	NA	NA	NA	NA	NA	NA	NA
1040	Serbian Hound Cross/Type	3.02	<0.1	NA	NA	NA	NA	NA	NA	NA	NA	NA
1041	Shar Pei X Shih Tzu	3.02	<0.1	NA	NA	NA	NA	NA	NA	NA	NA	NA
1042	Shar Pei X Siberian Husky	3.02	<0.1	NA	NA	NA	NA	NA	NA	NA	NA	NA
1043	Shar Pei X Spaniel Irish Water	3.02	<0.1	NA	NA	NA	NA	NA	NA	NA	NA	NA
1044	Shetland Sheepdog X Spaniel Cocker	3.02	<0.1	NA	NA	NA	NA	NA	NA	NA	NA	NA
1045	Shetland Sheepdog X Whippet	3.02	<0.1	NA	NA	NA	NA	NA	NA	NA	NA	NA

1046	Shih Tzu X Welsh Corgi Pembroke	3.02	<0.1	NA	NA	NA	NA	NA	NA	NA	NA	NA
1047	Small Münsterländer Cross/Type	3.02	<0.1	NA	NA	NA	NA	NA	NA	NA	NA	NA
1048	Soft Coated Wheaten Terrier X Staffordshire Bull Terrier	3.02	<0.1	NA	NA	NA	NA	NA	NA	NA	NA	NA
1049	Soft Coated Wheaten Terrier X West Highland White Terrier	3.02	<0.1	NA	NA	NA	NA	NA	NA	NA	NA	NA
1050	Spaniel American Cocker X Spaniel Cocker	3.02	<0.1	NA	NA	NA	NA	NA	NA	NA	NA	NA
1051	Spaniel Clumber X Spaniel Cocker	3.02	<0.1	NA	NA	NA	NA	NA	NA	NA	NA	NA
1052	Spaniel Cocker X Spaniel Field	3.02	<0.1	NA	NA	NA	NA	NA	NA	NA	NA	NA
1053	Spaniel Cocker X Spanish Water Dog	3.02	<0.1	NA	NA	NA	NA	NA	NA	NA	NA	NA
1054	Spaniel English Springer X West Highland White Terrier	3.02	<0.1	NA	NA	NA	NA	NA	NA	NA	NA	NA
1055	Spanish Hound X Spanish Mastiff	3.02	<0.1	NA	NA	NA	NA	NA	NA	NA	NA	NA
1056	Staffordshire Bull Terrier X Weimaraner	3.02	<0.1	NA	NA	NA	NA	NA	NA	NA	NA	NA
1057	Tibetan Spaniel X Welsh Corgi Pembroke	3.02	<0.1	NA	NA	NA	NA	NA	NA	NA	NA	NA
1058	Tosa Cross/Type	3.02	<0.1	NA	NA	NA	NA	NA	NA	NA	NA	NA
1059	West Highland White Terrier X Whippet	3.02	<0.1	NA	NA	NA	NA	NA	NA	NA	NA	NA

Supplementary Table 8. UK 2019 dog population regional estimates for proportional cephalic index (CI) demographics (%). Cephalic index population estimate, per region, and associated proportional cephalic index demographics both within $((N_{\text{CI, region}}/N_{\text{region}})*100)$ and between regions $((N_{\text{CI, region}}/N_{\text{total CI}})*100)$. Example: 24.7% of Central Scotland's population are listed as brachycephalic ('within region') and 1.2% of the UK brachycephalic population can be found within Central Scotland ('between regions').

Region	Cephalic Index	Population Estimate	Proportion Cephalic Index WITHIN Region (%)	Proportion Cephalic Index BETWEEN Regions (%)
Central Scotland	Brachycephalic	42404.63	24.7	1.2
Central Scotland	Mesocephalic	105739.44	61.5	3.4
Central Scotland	Dolichocephalic	23823.15	13.9	0.4
Channel Islands	Brachycephalic	4907.65	19.1	0.1
Channel Islands	Mesocephalic	16801.03	65.4	0.5
Channel Islands	Dolichocephalic	3975.96	15.5	0.1
East England	Brachycephalic	269629.52	20.6	7.9
East England	Mesocephalic	820281.47	62.6	26.3
East England	Dolichocephalic	219627.57	16.8	3.6
East Midlands	Brachycephalic	154597.72	21.7	4.5
East Midlands	Mesocephalic	441175.41	62.0	14.1
East Midlands	Dolichocephalic	116300.58	16.3	1.9
Glasgow	Brachycephalic	48595.17	27.5	1.4
Glasgow	Mesocephalic	105409.54	59.6	3.4
Glasgow	Dolichocephalic	22841.71	12.9	0.4
Highlands and Islands	Brachycephalic	9550.22	13.3	0.3
Highlands and Islands	Mesocephalic	53043.34	73.7	1.7
Highlands and Islands	Dolichocephalic	9344.07	13.0	0.2
Isle of Man	Brachycephalic	2554.05	16.5	0.1
Isle of Man	Mesocephalic	10978.25	70.7	0.4
Isle of Man	Dolichocephalic	1988.16	12.8	0.0
London	Brachycephalic	230444.04	24.7	6.7
London	Mesocephalic	550844.16	58.9	17.6
London	Dolichocephalic	153303.97	16.4	2.5
Lothian	Brachycephalic	35299.28	19.9	1.0
Lothian	Mesocephalic	118030.14	66.5	3.8
Lothian	Dolichocephalic	24209.55	13.6	0.4
Mid Scotland and Fife	Brachycephalic	19286.77	16.2	0.6
Mid Scotland and Fife	Mesocephalic	84835.44	71.0	2.7
Mid Scotland and Fife	Dolichocephalic	15298.37	12.8	0.3
Mid Wales	Brachycephalic	2096.24	11.7	0.1
Mid Wales	Mesocephalic	12776.01	71.1	0.4
Mid Wales	Dolichocephalic	3092.82	17.2	0.1
North East England	Brachycephalic	190786.62	25.2	5.6
North East England	Mesocephalic	450780.2	59.5	14.4
North East England	Dolichocephalic	115569.46	15.3	1.9

North East Scotland	Brachycephalic	32644.08	18.4	1.0
North East Scotland	Mesocephalic	120903.47	68.2	3.9
North East Scotland	Dolichocephalic	23728.03	13.4	0.4
North Wales	Brachycephalic	27028.25	21.5	0.8
North Wales	Mesocephalic	78736.6	62.7	2.5
North Wales	Dolichocephalic	19899.69	15.8	0.3
North West England	Brachycephalic	411588.16	27.8	12.0
North West England	Mesocephalic	853512.46	57.6	27.3
North West England	Dolichocephalic	215527.76	14.6	3.5
Northern Ireland	Brachycephalic	61600.04	23.1	1.8
Northern Ireland	Mesocephalic	173648.6	65.2	5.6
Northern Ireland	Dolichocephalic	31118.05	11.7	0.5
South East England	Brachycephalic	314197.53	19.4	9.2
South East England	Mesocephalic	1031435.07	63.8	33.0
South East England	Dolichocephalic	270797.07	16.8	4.4
South Scotland	Brachycephalic	34449.69	17.1	1.0
South Scotland	Mesocephalic	138069.99	68.7	4.4
South Scotland	Dolichocephalic	28522.43	14.2	0.5
South Wales	Brachycephalic	96710.63	30.5	2.8
South Wales	Mesocephalic	169595.29	53.5	5.4
South Wales	Dolichocephalic	50843.5	16.0	0.8
South West England	Brachycephalic	211719.45	16.9	6.2
South West England	Mesocephalic	837631.65	66.7	26.8
South West England	Dolichocephalic	205846.61	16.4	3.4
West Midlands	Brachycephalic	246401.23	21.8	7.2
West Midlands	Mesocephalic	703313.86	62.2	22.5
West Midlands	Dolichocephalic	181659.53	16.1	3.0
West Scotland	Brachycephalic	13636.2	22.9	0.4
West Scotland	Mesocephalic	38157.55	64.0	1.2
West Scotland	Dolichocephalic	7802.75	13.1	0.1
West Wales	Brachycephalic	71058.25	26.0	2.1
West Wales	Mesocephalic	154391.4	56.6	4.9
West Wales	Dolichocephalic	47485.11	17.4	0.8
Yorkshire and The Humber	Brachycephalic	310194.38	24.8	9.1
Yorkshire and The Humber	Mesocephalic	745347.53	59.7	23.9
Yorkshire and The Humber	Dolichocephalic	193150.36	15.5	3.2
Central Scotland	Brachycephalic	42404.63	24.7	1.2
Central Scotland	Mesocephalic	105739.44	61.5	3.4
Central Scotland	Dolichocephalic	23823.15	13.9	0.4
Channel Islands	Brachycephalic	4907.65	19.1	0.1
Channel Islands	Mesocephalic	16801.03	65.4	0.5
Channel Islands	Dolichocephalic	3975.96	15.5	0.1
East England	Brachycephalic	269629.52	20.6	7.9
East England	Mesocephalic	820281.47	62.6	26.3

East England	Dolichocephalic	219627.57	16.8	3.6
East Midlands	Brachycephalic	154597.72	21.7	4.5
East Midlands	Mesocephalic	441175.41	62.0	14.1
East Midlands	Dolichocephalic	116300.58	16.3	1.9
Glasgow	Brachycephalic	48595.17	27.5	1.4
Glasgow	Mesocephalic	105409.54	59.6	3.4
Glasgow	Dolichocephalic	22841.71	12.9	0.4
Highlands and Islands	Brachycephalic	9550.22	13.3	0.3
Highlands and Islands	Mesocephalic	53043.34	73.7	1.7
Highlands and Islands	Dolichocephalic	9344.07	13.0	0.2
Isle of Man	Brachycephalic	2554.05	16.5	0.1
Isle of Man	Mesocephalic	10978.25	70.7	0.4
Isle of Man	Dolichocephalic	1988.16	12.8	0.0
London	Brachycephalic	230444.04	24.7	6.7
London	Mesocephalic	550844.16	58.9	17.6
London	Dolichocephalic	153303.97	16.4	2.5
Lothian	Brachycephalic	35299.28	19.9	1.0
Lothian	Mesocephalic	118030.14	66.5	3.8
Lothian	Dolichocephalic	24209.55	13.6	0.4
Mid Scotland and Fife	Brachycephalic	19286.77	16.2	0.6
Mid Scotland and Fife	Mesocephalic	84835.44	71.0	2.7
Mid Scotland and Fife	Dolichocephalic	15298.37	12.8	0.3
Mid Wales	Brachycephalic	2096.24	11.7	0.1
Mid Wales	Mesocephalic	12776.01	71.1	0.4
Mid Wales	Dolichocephalic	3092.82	17.2	0.1
North East England	Brachycephalic	190786.62	25.2	5.6
North East England	Mesocephalic	450780.2	59.5	14.4
North East England	Dolichocephalic	115569.46	15.3	1.9
North East Scotland	Brachycephalic	32644.08	18.4	1.0
North East Scotland	Mesocephalic	120903.47	68.2	3.9
North East Scotland	Dolichocephalic	23728.03	13.4	0.4
North Wales	Brachycephalic	27028.25	21.5	0.8
North Wales	Mesocephalic	78736.6	62.7	2.5
North Wales	Dolichocephalic	19899.69	15.8	0.3
North West England	Brachycephalic	411588.16	27.8	12.0
North West England	Mesocephalic	853512.46	57.6	27.3
North West England	Dolichocephalic	215527.76	14.6	3.5
Northern Ireland	Brachycephalic	61600.04	23.1	1.8
Northern Ireland	Mesocephalic	173648.6	65.2	5.6
Northern Ireland	Dolichocephalic	31118.05	11.7	0.5
South East England	Brachycephalic	314197.53	19.4	9.2
South East England	Mesocephalic	1031435.07	63.8	33.0
South East England	Dolichocephalic	270797.07	16.8	4.4
South Scotland	Brachycephalic	34449.69	17.1	1.0

South Scotland	Mesocephalic	138069.99	68.7	4.4
South Scotland	Dolichocephalic	28522.43	14.2	0.5
South Wales	Brachycephalic	96710.63	30.5	2.8
South Wales	Mesocephalic	169595.29	53.5	5.4
South Wales	Dolichocephalic	50843.5	16.0	0.8
South West England	Brachycephalic	211719.45	16.9	6.2
South West England	Mesocephalic	837631.65	66.7	26.8
South West England	Dolichocephalic	205846.61	16.4	3.4
West Midlands	Brachycephalic	246401.23	21.8	7.2
West Midlands	Mesocephalic	703313.86	62.2	22.5
West Midlands	Dolichocephalic	181659.53	16.1	3.0
West Scotland	Brachycephalic	13636.2	22.9	0.4
West Scotland	Mesocephalic	38157.55	64.0	1.2
West Scotland	Dolichocephalic	7802.75	13.1	0.1
West Wales	Brachycephalic	71058.25	26.0	2.1
West Wales	Mesocephalic	154391.4	56.6	4.9
West Wales	Dolichocephalic	47485.11	17.4	0.8
Yorkshire and The Humber	Brachycephalic	310194.38	24.8	9.1
Yorkshire and The Humber	Mesocephalic	745347.53	59.7	23.9
Yorkshire and The Humber	Dolichocephalic	193150.36	15.5	3.2

Supplementary Table 9. UK 2019 dog population regional estimates for proportional body size (BS) demographics (%). Body size population estimate, per region, and associated proportional body size demographics both within (($N_{BS, region}/N_{region}$)*100) and between regions (($N_{BS, region}/N_{total BS}$)*100). Example: 31.3% of Central Scotland's population are listed as large breeds ('within region') and 1.6% of the UK large-breed population can be found within Central Scotland ('between regions').

Region	Body Size	Population Estimate	Proportion Body Size WITHIN Region (%)	Proportion Body Size BETWEEN Regions (%)
Central Scotland	Large	53760.99	31.3	1.6
Central Scotland	Medium	26645.98	15.5	0.9
Central Scotland	Small	91560.25	53.2	1.5
Channel Islands	Large	7312.45	28.5	0.2
Channel Islands	Medium	4576.58	17.8	0.1
Channel Islands	Small	13795.61	53.7	0.2
East England	Large	425871.98	32.5	12.5
East England	Medium	228768.56	17.5	7.3
East England	Small	654898.02	50.0	10.7
East Midlands	Large	219211.03	30.8	6.4
East Midlands	Medium	136605.46	19.2	4.4
East Midlands	Small	356257.21	50.0	5.8
Glasgow	Large	45221.62	25.6	1.3
Glasgow	Medium	28516.02	16.1	0.9
Glasgow	Small	103108.78	58.3	1.7
Highlands and Islands	Large	26605.01	37.0	0.8
Highlands and Islands	Medium	13797.97	19.2	0.4
Highlands and Islands	Small	31534.65	43.8	0.5
Isle of Man	Large	5346.74	34.4	0.2
Isle of Man	Medium	3500.8	22.6	0.1
Isle of Man	Small	6672.92	43.0	0.1
London	Large	232590.12	24.9	6.8
London	Medium	149797.37	16	4.8
London	Small	552204.69	59.1	9.0
Lothian	Large	53251.25	30.0	1.6
Lothian	Medium	30175.36	17.0	1.0
Lothian	Small	94112.35	53.0	1.5
Mid Scotland and Fife	Large	44910.76	37.6	1.3
Mid Scotland and Fife	Medium	22058.21	18.5	0.7
Mid Scotland and Fife	Small	52451.6	43.9	0.9
Mid Wales	Large	5151.15	28.7	0.2
Mid Wales	Medium	4027.12	22.4	0.1
Mid Wales	Small	8786.8	48.9	0.1
North East England	Large	197852.33	26.1	5.8
North East England	Medium	134521.78	17.8	4.3
North East England	Small	424762.17	56.1	7.0
North East Scotland	Large	60431.12	34.1	1.8

North East Scotland	Medium	28542.07	16.1	0.9
North East Scotland	Small	88302.37	49.8	1.4
North Wales	Large	39549.32	31.5	1.2
North Wales	Medium	24011.83	19.1	0.8
North Wales	Small	62103.39	49.4	1.0
North West England	Large	394522.83	26.6	11.5
North West England	Medium	279223.28	18.9	8.9
North West England	Small	806882.27	54.5	13.2
Northern Ireland	Large	70419.97	26.4	2.1
Northern Ireland	Medium	38184.53	14.3	1.2
Northern Ireland	Small	157762.19	59.2	2.6
South East England	Large	503146.32	31.1	14.7
South East England	Medium	301763.38	18.7	9.7
South East England	Small	811519.98	50.2	13.3
South Scotland	Large	71253.16	35.4	2.1
South Scotland	Medium	32591.21	16.2	1.0
South Scotland	Small	97197.75	48.3	1.6
South Wales	Large	73531.44	23.2	2.2
South Wales	Medium	62508.11	19.7	2.0
South Wales	Small	181109.88	57.1	3.0
South West England	Large	399697.61	31.8	11.7
South West England	Medium	255364.59	20.3	8.2
South West England	Small	600135.5	47.8	9.8
West Midlands	Large	327887.18	29.0	9.6
West Midlands	Medium	215212.79	19.0	6.9
West Midlands	Small	588274.65	52.0	9.6
West Scotland	Large	17970.56	30.2	0.5
West Scotland	Medium	10747.65	18.0	0.3
West Scotland	Small	30878.29	51.8	0.5
West Wales	Large	67734.5	24.8	2.0
West Wales	Medium	42341.68	15.5	1.4
West Wales	Small	162858.59	59.7	2.7
Yorkshire and The Humber	Large	367904.04	29.5	10.8
Yorkshire and The Humber	Medium	224012.51	17.9	7.2
Yorkshire and The Humber	Small	656775.72	52.6	10.8
Central Scotland	Large	53760.99	31.3	1.6
Central Scotland	Medium	26645.98	15.5	0.9
Central Scotland	Small	91560.25	53.2	1.5
Channel Islands	Large	7312.45	28.5	0.2
Channel Islands	Medium	4576.58	17.8	0.1
Channel Islands	Small	13795.61	53.7	0.2
East England	Large	425871.98	32.5	12.5
East England	Medium	228768.56	17.5	7.3
East England	Small	654898.02	50.0	10.7

East Midlands	Large	219211.03	30.8	6.4
East Midlands	Medium	136605.46	19.2	4.4
East Midlands	Small	356257.21	50.0	5.8
Glasgow	Large	45221.62	25.6	1.3
Glasgow	Medium	28516.02	16.1	0.9
Glasgow	Small	103108.78	58.3	1.7
Highlands and Islands	Large	26605.01	37.0	0.8
Highlands and Islands	Medium	13797.97	19.2	0.4
Highlands and Islands	Small	31534.65	43.8	0.5
Isle of Man	Large	5346.74	34.4	0.2
Isle of Man	Medium	3500.8	22.6	0.1
Isle of Man	Small	6672.92	43.0	0.1
London	Large	232590.12	24.9	6.8
London	Medium	149797.37	16.0	4.8
London	Small	552204.69	59.1	9.0
Lothian	Large	53251.25	30.0	1.6
Lothian	Medium	30175.36	17.0	1.0
Lothian	Small	94112.35	53.0	1.5
Mid Scotland and Fife	Large	44910.76	37.6	1.3
Mid Scotland and Fife	Medium	22058.21	18.5	0.7
Mid Scotland and Fife	Small	52451.6	43.9	0.9
Mid Wales	Large	5151.15	28.7	0.2
Mid Wales	Medium	4027.12	22.4	0.1
Mid Wales	Small	8786.8	48.9	0.1
North East England	Large	197852.33	26.1	5.8
North East England	Medium	134521.78	17.8	4.3
North East England	Small	424762.17	56.1	7.0
North East Scotland	Large	60431.12	34.1	1.8
North East Scotland	Medium	28542.07	16.1	0.9
North East Scotland	Small	88302.37	49.8	1.4
North Wales	Large	39549.32	31.5	1.2
North Wales	Medium	24011.83	19.1	0.8
North Wales	Small	62103.39	49.4	1.0
North West England	Large	394522.83	26.6	11.5
North West England	Medium	279223.28	18.9	8.9
North West England	Small	806882.27	54.5	13.2
Northern Ireland	Large	70419.97	26.4	2.1
Northern Ireland	Medium	38184.53	14.3	1.2
Northern Ireland	Small	157762.19	59.2	2.6
South East England	Large	503146.32	31.1	14.7
South East England	Medium	301763.38	18.7	9.7
South East England	Small	811519.98	50.2	13.3
South Scotland	Large	71253.16	35.4	2.1
South Scotland	Medium	32591.21	16.2	1.0

South Scotland	Small	97197.75	48.3	1.6
South Wales	Large	73531.44	23.2	2.2
South Wales	Medium	62508.11	19.7	2.0
South Wales	Small	181109.88	57.1	3.0
South West England	Large	399697.61	31.8	11.7
South West England	Medium	255364.59	20.3	8.2
South West England	Small	600135.5	47.8	9.8
West Midlands	Large	327887.18	29.0	9.6
West Midlands	Medium	215212.79	19.0	6.9
West Midlands	Small	588274.65	52.0	9.6
West Scotland	Large	17970.56	30.2	0.5
West Scotland	Medium	10747.65	18.0	0.3
West Scotland	Small	30878.29	51.8	0.5
West Wales	Large	67734.5	24.8	2.0
West Wales	Medium	42341.68	15.5	1.4
West Wales	Small	162858.59	59.7	2.7
Yorkshire and The Humber	Large	367904.04	29.5	10.8
Yorkshire and The Humber	Medium	224012.51	17.9	7.2
Yorkshire and The Humber	Small	656775.72	52.6	10.8

Supplementary Table 10. Recognised breeds[25,26] used in analyses, and associated collapsed breed names, based on alternative breed names, known ancestry and/or presumed breed popularity. Presence of recognised breed does not ensure presence within dataset. 'NA' within collapsed breed names does not exclude possibility of alternate breed names – only absence of alternate breed names within dataset. Breed grouped by Cephalic Index (Brachycephalic, Mesocephalic, Dolichocephalic[31]) and Body Size (Small, Medium, Large[25,26]).

**Recognised Breed**	**Cephalic Index**	**Body Size**	**Collapsed Breed Names**
Affenpinscher	Brachycephalic	Small	NA
Afghan Hound	Dolichocephalic	Large	Hound Pashmi
Airedale Terrier	Dolichocephalic	Medium	Airedale
Alaskan Malamute	Mesocephalic	Large	Malamute, Alaskan Giant, Shepherd Alaskan, Utonagan
Alpine Dachsbracke	Mesocephalic	Medium	Dachsbracke, Basset des Alpes, Dachsbracke Alpine Alpenlandische Basset des Alpes, Dachsbracke Dutch Strellufstover
American Akita	Mesocephalic	Large	Akita, Akita Grand, Akita Miniature, Akita Standard
American Staffordshire Terrier	Mesocephalic	Medium	Amstaff, Staff American, Staffie American, Staffy American, Terrier American, Terrier Staffordshire Bull American
Anatolian Shepherd Dog	Mesocephalic	Large	Karabash Anatolian
Appenzell Cattle Dog	Mesocephalic	Medium	Appenzeller, Appenzeller Sennenhund, Appenzeller Sennenhunde, Mountain Appenzell, Mountain Appenzeller, Mountain Sennenhund
Ariege Pointing Dog	Mesocephalic	Medium	NA
Ariegeois	Mesocephalic	Medium	NA
Artois Hound	Mesocephalic	Medium	Chien d'Artois
Atlas Mountain Dog	Mesocephalic	Medium	Aidi, Atlas Schaferhund, Perro de paster del Atlas, Shepherd Atlas
Australian Cattle Dog	Mesocephalic	Medium	Heeler, Cattle Australian Stumpy Tail, Heeler Australian, Heeler Blue, Heeler Red
Australian Kelpie	Dolichocephalic	Medium	NA
Australian Shepherd	Mesocephalic	Medium	Collie Austrailian, Koolie, Koolie Australian, Sheepdog Australian, Shepherd American Miniature, Shepherd Australian Miniature, Shepherd North American
Australian Silky Terrier	Mesocephalic	Small	Silky, Terrier Silky, Terrier Sydney Silky
Australian Terrier	Mesocephalic	Small	NA
Austrian Black And Tan Hound	Mesocephalic	Medium	Brandlbracke Austrian, Hound Austrian
Austrian Pinscher	Dolichocephalic	Medium	Pinscher Austrian Short Hair, Pinscher Swiss Glattharidge, Pinscher Swiss Short Hair
Auvergne Pointer	Mesocephalic	Medium	Braque Auvergne, Braque D'Auvergne, Pointer Auvergne, Pointer French, Pointing French
Azawakh	Dolichocephalic	Medium	Sahara
Barbet	Mesocephalic	Medium	Water French
Basenji	Mesocephalic	Small	Bush Basenji African
Basset Artesien Normand	Dolichocephalic	Medium	NA
Basset Bleu de Gascogne	Dolichocephalic	Medium	Basset Blue Gascony, Basset Gascony Blue, Bleu de Gascogne Grand, Blue Gascony Griffon, Grand Bleu de Gascogne, Grand Bleu Gascogne, Griffon Bleu de Gascogne, Griffon Bleu de Gascogne Grand, Griffon Bleu de Gascogne Petit, Hound American Blue Gascon, Hound Bleu de Gascogne Grand
Basset Fauve de Bretagne	Mesocephalic	Medium	Basset Fauve, Fauve, Basset Brittany, Basset Fauve Bretagne, Basset Faux de Britt, Basset Fawn Brittany, Basset French, Fauves de Bretagne
Basset Griffon Vendeen Grand	Dolichocephalic	Medium	Griffon Vendeen Grand, Basset Grand, Basset Griffon Grand, Basset Griffon Hound Grand
Basset Griffon Vendeen Petit	Dolichocephalic	Medium	Basset Griffon Vendeen, Basset Griffon de Vendeen, Basset Griffon Hound Petit, Basset Griffon Petit, Basset Griffon Vendeen Small, Basset Vendeen Petit, Basset Vendeen Small

Basset Hound	Dolichocephalic	Medium	Basset, Basset Hush
Bavarian Mountain Hound	Mesocephalic	Medium	Hound Bavarian, Mountain Bavarian, Mountain Scent Hound Bavarian
Beagle	Mesocephalic	Small	Beagle Elizabethan, Beagle English, Beagle Mal, Beagle Pocket, Beagle Smooth, Beagle Standard
Beagle Harrier	Mesocephalic	Medium	Harrier Beagle
Bearded Collie	Mesocephalic	Medium	Beardie, Smithfield
Beauceron	Mesocephalic	Large	Bas Rouge, Berger de Beauce, Guard French, Shepherd Beauce
Bedlington Terrier	Dolichocephalic	Small	Bedlington
Belgian Shepherd Dog Groenendael	Dolichocephalic	Medium	Groenendael, Belgian Groenendael
Belgian Shepherd Dog Laekenois	Dolichocephalic	Medium	Laekenois, Belgian Laekenois
Belgian Shepherd Dog Malinois	Dolichocephalic	Medium	Malinois, Belgian Malinois, Shepherd Belgian, Wolfhound Belgian
Belgian Shepherd Dog Tervueren	Dolichocephalic	Medium	Tervueren, Belgian Tervuren
Bergamasco	Mesocephalic	Large	Cane da Pastore Bergamasco, Sheepdog Bergamasco, Shepherd Bergamasco Cane da Pastore Bergamasco, Shepherd Bergamese
Bernese Mountain Dog	Mesocephalic	Large	NA
Bichon Frise	Mesocephalic	Small	Bichon
Billy	Mesocephalic	Medium	NA
Black And Tan Coonhound	Mesocephalic	Large	Coonhound American, Coonhound American English, Coonhound Black Tan, Coonhound Bluetick, Coonhound English
Bloodhound	Dolichocephalic	Large	Braque du Bourbonnais
Blue Picardy Spaniel	Mesocephalic	Medium	NA
Bohemian Wire Haired Pointing Griffon	Mesocephalic	Medium	Griffon Pointing Wire Hair, Griffon Rough Hair, Pointing Griffon Wire Hair
Bolognese	Mesocephalic	Small	NA
Border Collie	Mesocephalic	Medium	Collie, Collie Border Welsh, Collie English, Collie Farm, Collie Irish, Collie Old Farm, Sheepdog Welsh, Sheepdog Welsh Working, Shepherd Welsh
Border Terrier	Mesocephalic	Small	Terrier Border
Borzoi	Dolichocephalic	Large	Hound East Russian Coursing, Hound Russian, Hound Russian Harlequin, Sighthound Russian, Sighthound Russian Hunting, Wolfhound Russian
Bosnian Broken Haired Hound		Medium	Bosanski Oštrodlaki Gonič Barak, Hound Bosnian
Boston Terrier	Mesocephalic	Small	NA
Bourbonnais Pointing Dog	Brachycephalic	Medium	Braque du Bourbonnais
Bouvier Des Ardennes	Mesocephalic	Large	NA
Bouvier Des Flandres	Mesocephalic	Large	NA
Boxer	Mesocephalic	Large	NA
Bracco Italiano	Brachycephalic	Large	Pointer Italian, Pointing Italian, Setter Italian
Brazilian Terrier	Dolichocephalic	Medium	Fox Paulistinha
Briard	Mesocephalic	Large	Berger de Brie, Briard French
Briquet Griffon Vendeen	Mesocephalic	Medium	Briquet
Brittany	Mesocephalic	Medium	Brittany French, Brittany Spaniel, Epagneul Breton, Spaniel Breton, Spaniel Brittany American, Spaniel Brittany French
Broholmer	Mesocephalic	Large	NA
Bull Terrier	Mesocephalic	Medium	Terrier Bull American, Terrier Bull English, Terrier Bull English Standard, Terrier Bull English Varkhond Standard, Terrier Bull Irish, Terrier Bull Old English
Bull Terrier Miniature	Dolichocephalic	Small	Terrier Bull English Miniature, Terrier Bull English Varkhond Miniature, Terrier Bull Miniature

Bulldog	Dolichocephalic	Medium	Bull American, Bulldog British, Bulldog Dorset, Bulldog Dorset Old English, Bulldog Dorset Old Tyme, Bulldog English, Bulldog Miniature, Bulldog Old English, Bulldog Old Tyme, Bulldog Old Victorian, Bulldog Olde English, Bulldog Olde Tyme, Bulldog Olde Victorian, Bulldog Standard, Bulldog Sussex, Bulldog Teacup, Bulldog Toy, Bulldog Valley, Bulldog Victorian, Bulldogge, Dorset Old Tyme
Bullmastiff	Brachycephalic	Large	Bullmastiff English, Bullmastiff Spanish
Burgos Pointing Dog	Brachycephalic	Large	Perdiguero de Burgos, Pointing Burgos
Cairn Terrier	Mesocephalic	Small	Cairn
Canaan Dog	Mesocephalic	Medium	Canaan, Canaan Egyptian, Shepherd Bedouin
Canadian Eskimo Dog	Mesocephalic	Medium	Eskimo, Eskie, Eskimo American, Hound Eskimo, Inuit Canadian, Inuit Eskimo American Miniature, Inuit Eskimo American Standard, Inuit Eskimo Canadian, Inuit Eskimo Northern
Canarian Warren Hound	Mesocephalic	Medium	Hound Canary Warren, Podenco, Podenco Andaluz, Podenco Canario, Podenco Spanish
Castro Laboreiro Dog	Dolichocephalic	Large	Castro Laboreiro, Cattle Portuguese, Watch Portuguese
Catalan Sheepdog	Mesocephalic	Medium	Sheepdog Spanish
Caucasian Shepherd Dog	Mesocephalic	Large	Carovcharka, Mountain Caucasian, Ovcharka, Ovtcharka Caucasian, Sheepdog Caucasian, Shepherd Asian
Cavalier King Charles Spaniel	Mesocephalic	Small	Cavalier
Central Asia Shepherd Dog	Brachycephalic	Large	Ovtcharka Central Asian, Ovtcharka Middle Asian, Sage Koochee, Sheepdog Central Asian
Cesky Terrier	Mesocephalic	Small	Terrier Bohemian, Terrier Czesky
Chihuahua Long Coat	Mesocephalic	Small	Chihuahua Long Hair
Chihuahua Smooth Coat	Brachycephalic	Small	Chi, Chihuahua, Chihuahua Deer Head, Chihuahua Miniature, Chihuahua Short Coat, Chihuahua Smooth Hair, Chihuahua Standard, Chihuahua Teacup
Chinese Crested	Brachycephalic	Small	Chinese Crested Hairless, Chinese Crested Powder Puff, Hairless Chinese, Hairless Chinese Crested, Japanese Crest, Powder Puff, Powderpuff Satz
Chow Chow	Mesocephalic	Large	NA
Cimarron Uruguayo	Mesocephalic	Medium	Perro Cimarrón Uruguayo Perro Criollo Perro Gaucho
Cirneco dell'Etna	Mesocephalic	Medium	Hound Sicilian
Collie Rough	Mesocephalic	Medium	NA
Collie Smooth	Dolichocephalic	Medium	NA
Coton de Tulear	Dolichocephalic	Small	Coton, Madagascan
Croatian Shepherd Dog	Mesocephalic	Medium	Sheepdog Croatian, Shepherd Bosnian, Shepherd Herzegovinian, Tornjak
Czechoslovakian Wolfdog	Dolichocephalic	Large	Chien Loup, Wolfdog Czech, Wolfdog Solvakian
Dachshund Long Haired	Dolichocephalic	Medium	Daxon Long Hair
Dachshund Miniature Long Haired	Dolichocephalic	Small	Daxon Long Hair Miniature
Dachshund Miniature Smooth Haired	Dolichocephalic	Small	Dachshund Miniature, Daxon Miniature, Daxon Smooth Hair Miniature, Sausage Miniature
Dachshund Miniature Wire Haired	Dolichocephalic	Small	Dachshund Miniature Wire Hair, Daxon Wire Hair Miniature
Dachshund Smooth Haired	Dolichocephalic	Medium	Dachshund, Dachshund Old Format, Dachshund Short Hair, Dachshund Standard, Dachshund Teckel, Dax, Daxon Smooth Hair, Sausage, Teckel
Dachshund Wire Haired	Dolichocephalic	Medium	Daxon Wire Hair
Dalmatian	Dolichocephalic	Medium	NA
Dandie Dinmont Terrier	Mesocephalic	Small	NA
Danish Swedish Farmdog	Mesocephalic	Small	NA
Deerhound	Mesocephalic	Large	Deerhound Scottish, Hound Scottish
Dobermann	Dolichocephalic	Large	Pinscher Doberman, Pinscher Dobermann
Dogo Argentino	Dolichocephalic	Large	Dogo Argentine, Mastiff Argentinian

Dogue de Bordeaux	Mesocephalic	Large	Bordeaux, Bulldog Bordeaux, French Mastiff, Mastiff Bordeaux, Mastiff French
Drentsche Partridge Dog	Brachycephalic	Medium	Drentse Patrijshond, Partridge Dutch
Drever	Mesocephalic	Medium	Strellufstover
Dutch Schapendoes	Mesocephalic	Medium	Schapendoes, Sheepdog Dutch
Dutch Shepherd Dog	Mesocephalic	Medium	Herder Dutch, Herdershond Hollandse, Shepherd Dutch German, Shepherd Dutch Long Hair, Shepherd Dutch Rough Hair, Shepherd Dutch Short Hair, Shepherd Holland
Dutch Smoushond	Dolichocephalic	Small	NA
East Siberia Laika	Mesocephalic	Medium	Laika
English Setter	Mesocephalic	Large	Setter, Blue Belton, Setter English Red
English Toy Terrier	Mesocephalic	Small	Terrier Black And Tan, Terrier Black And Tan Toy, Terrier Teacup, Terrier Toy
Entlebucher Mountain Dog	Mesocephalic	Medium	Entlebucher, Cattle Entlebuch, Entlebucher Sennenhund
Estrela Mountain Dog	Mesocephalic	Large	Guard Portuguese, Mountain Estrela Long Hair, Mountain Estrela Short Hair, Mountain Portuguese, Serra da Estrela
Eurasier	Mesocephalic	Medium	Eurasian
Fila Brasileiro	Mesocephalic	Large	Cão de Fila, Dogue Brasileiro, Mastiff Brazilian, Molosser Brazilian
Finnish Hound	Brachycephalic	Medium	NA
Finnish Lapphund	Mesocephalic	Medium	Lapphund Finnish
Finnish Spitz	Mesocephalic	Small	NA
Fox Terrier Smooth	Mesocephalic	Small	NA
Fox Terrier Wire	Dolichocephalic	Small	Terrier Fox, Terrier Fox Miniature, Terrier Fox Standard, Terrier Fox Teacup, Terrier Fox Toy
Foxhound	Dolichocephalic	Large	Foxhound English
French Bulldog	Mesocephalic	Small	French Bull, Frenchie, Quad, Quad Carrier
French Pointing Dog Gascogne Type	Mesocephalic	Medium	NA
French Pointing Dog Pyrenean Type	Mesocephalic	Medium	Braque Français, Braque Français de Grand Taille, Braque Français de Petite Taille
French Spaniel	Mesocephalic	Medium	NA
French Tricolour Hound	Mesocephalic	Medium	NA
French White And Black Hound	Mesocephalic	Medium	Chien Français Blanc et Noir, Levesque
Frisian Water Dog	Mesocephalic	Medium	Spaniel Water Dutch, Wetterhoun
Gascon Saintongeois	Mesocephalic	Large	Gascon Saintongeois Petit
German Hound	Dolichocephalic	Medium	Harvard German
German Hunting Terrier	Mesocephalic	Medium	Deutscher Jagdterrier, Jagdterrier, Terrier German Hunt, Terrier German Jgad
German Longhaired Pointer	Mesocephalic	Large	Deutsch Langhaar, Pointer German, Pointer Long Hair
German Pinscher	Mesocephalic	Medium	Pinscher, Glattharidge, Pincher
German Roughhaired Pointer	Dolichocephalic	Medium	Deutsch Stichelhaar, Pointer German Rough Hair
German Shepherd Dog	Mesocephalic	Large	Alsatian, Guard German, Sheepdog German, Shepherd American German, Shepherd Bohemian, Shepherd Caucasian German, Shepherd Czech, Shepherd Czech German, Shepherd German Double Coat, Shepherd German Harsh Coat, Shepherd German Long Coat
German Shorthaired Pointer	Dolichocephalic	Medium	Pointer Belgian Short Hair, Pointer German Short Hair
German Spaniel	Mesocephalic	Medium	NA
German Spitz Giant	Mesocephalic	Medium	Spitz Giant
German Spitz Klein	Mesocephalic	Small	Klein, Spitz Klein, Spitz Miniature, Spitz German Toy
German Spitz Medium	Mesocephalic	Medium	Spitz Medium
German Spitz Mittel	Mesocephalic	Medium	Spitz, Spitz Mittel, Spitz German, Spitz German Standard, Mittel
German Wirehaired Pointer	Mesocephalic	Large	Deutscher Stichelhaariger Vorstehhund, Pointer Wire Hair
Giant Schnauzer	Mesocephalic	Large	NA

Glen Of Imaal Terrier	Mesocephalic	Small	Terrier Irish Glen Of Imaal
Gordon Setter	Mesocephalic	Large	NA
Great Anglo French Hound	Dolichocephalic	Medium	Anglo Français Grand, Hound Anglo Français, Hound French
Great Anglo French Tricolour Hound	Mesocephalic	Large	NA
Great Anglo French White And Black Hound	Mesocephalic	Large	NA
Great Anglo French White And Orange Hound	Mesocephalic	Large	NA
Great Dane	Mesocephalic	Large	Deutschege
Great Swiss Mountain Dog	Dolichocephalic	Large	Cattle Great Swiss, Hound Mountain Swiss, Mountain Swiss, Mountain Swiss Bouvier Suisse
Greenland Dog	Mesocephalic	Large	Greenland, Grunlandshund, Hound Greenland
Greyhound	Mesocephalic	Large	Greyhound Australian, Greyhound Banjara, Greyhound English, Greyhound Irish, Greyhound Rampur, Hound West Russian Coursing
Griffon Bruxellois	Dolichocephalic	Small	Griffon, Brabancon Petit, Bruxellois Standard, Griffon Belge, Griffon Belgian, Griffon Brussels
Griffon Fauve de Bretagne	Brachycephalic	Medium	Fawn Brittany Griffon
Griffon Nivernais	Mesocephalic	Medium	Chien de Pays
Halden Hound	Mesocephalic	Medium	Haldenstøvare
Hamiltonstovare	Mesocephalic	Medium	Hound Hamilton, Hound Swedish Fox
Hanoverian Scent Hound	Mesocephalic	Large	Hound Hanover
Harrier	Mesocephalic	Medium	Hound Harrier
Havanese	Mesocephalic	Small	Terrier Havanese Toy
Hellenic Hound	Mesocephalic	Medium	Harehound, Harehound Greek, Hellenikos Poimenikos, Hound Greek
Hokkaido	Mesocephalic	Medium	Ainu
Hovawart	Mesocephalic	Large	NA
Hungarian Greyhound	Mesocephalic	Medium	Magyar Agar
Hungarian Hound Transylvanian Scent Hound	Dolichocephalic	Medium	Hound Transylvanian, Hound Transylvanian Scent, Hungarian Hound, Transylvanian Scent
Hungarian Kuvasz	Dolichocephalic	Large	NA
Hungarian Puli	Mesocephalic	Medium	Water Hungarian
Hungarian Pumi	Mesocephalic	Small	NA
Hungarian Vizsla	Mesocephalic	Medium	Vizsla, Pointer Hungarian Short Hair, Pointer Hungarian Wire Hair, Pointing Hungarian Short Hair, Pointing Hungarian Wire Hair, Vizsla German, Vizsla Hungarian Short Hair, Vizsla Hungarian Wire Hair, Vizsla Miniature, Vizsla Wire Hair
Ibizan Hound	Mesocephalic	Medium	Hound Bizanian, Hound Ibizan Rough Hair, Hound Ibizan Smooth Hair, Hunting Ibizian, Podenco Ibicenco, Podenco Ibizan, Warren Ibizan
Icelandic Sheepdog	Dolichocephalic	Medium	Friaar
Irish Red And White Setter	Mesocephalic	Large	NA
Irish Setter	Mesocephalic	Large	Setter Irish Red, Setter Red
Irish Terrier	Mesocephalic	Small	Terrier Irish Balla
Irish Wolfhound	Dolichocephalic	Large	Wolfhound, Wolfdog British
Istrian Short Haired Hound	Dolichocephalic	Medium	Hound Istrian Coarse Hair
Italian Cane Corso	Mesocephalic	Large	Cane Corso, Corso Italian, Corso King, Mastiff Italian, Mastiff Sicilian
Italian Greyhound	Brachycephalic	Small	Greyhound Italian, Piccolo Levriero Italiano, Sighthound Italian
Italian Short Haired Segugio	Dolichocephalic	Large	Hound Italian, Hound Segugio, Segugio Italiano
Italian Spinone	Dolichocephalic	Large	Spinone, Hunter Italian
Italian Volpino	Mesocephalic	Small	Volpino
Jack Russell Terrier	Mesocephalic	Small	Jack Russell, Terrier Irish Jack Russell, Terrier Jack Russell Miniature, Terrier Jack Russell Toy, Terrier Jack Russell Wire Hair

Jamthund	Mesocephalic	Medium	Elkhound Swedish
Japanese Akita Inu	Mesocephalic	Large	Akita Inu, Akita Inu Japanese, Akita Japanese, Japanese Great
Japanese Chin	Mesocephalic	Small	Chin, Spaniel Japanese
Japanese Shiba Inu	Brachycephalic	Small	Shiba
Japanese Spitz	Mesocephalic	Small	NA
Japanese Terrier	Mesocephalic	Small	NA
Kai	Mesocephalic	Medium	Kai Ken, Tiger
Karelian Bear Dog	Mesocephalic	Medium	Bear Karelian
Karst Shepherd Dog	Mesocephalic	Medium	Ovcar Krasky
Keeshond	Mesocephalic	Medium	Wolfspitz German
Kerry Blue Terrier	Mesocephalic	Medium	Terrier Irish Blue
King Charles Spaniel	Dolichocephalic	Small	Spaniel English Toy, Spaniel King Charles Miniature
Kishu	Brachycephalic	Medium	Inu Kishu, Kishu Ken
Komondor	Mesocephalic	Large	Sheepdog Hungarian
Kooikerhondje	Mesocephalic	Small	Kooiker, Dutch Kooiker, Spaniel Dutch, Waterfowl Dutch Small
Korean Jindo	Mesocephalic	Medium	NA
Korthals Griffon	Mesocephalic	Medium	Griffon Pointing French Wire Hair
Kromfohrlander	Mesocephalic	Medium	NA
Lagotto Romagnolo	Mesocephalic	Small	Lagotto, Lagotto Italian, Water Romagna
Lakeland Terrier	Mesocephalic	Small	NA
Lancashire Heeler	Dolichocephalic	Small	Heeler Lancashire, Heeler Ormskirk
Landseer	Mesocephalic	Large	Landseer Continental
Lapponian Herder	Mesocephalic	Medium	Lapinporokoira, Lapland Rein, Lapland Reindeer
Large Münsterländer	Mesocephalic	Large	Münsterländer Vorstehhund Grosser
Leonberger	Mesocephalic	Large	NA
Lhasa Apso	Mesocephalic	Small	NA
Löwchen	Brachycephalic	Small	Little Lion
Majorca Mastiff	Mesocephalic	Large	Ca de Bou Mallorquin, Mastiff Mallorquin, Perro de Presa Mallorquin, Perroo Mallorquin, Presa Mallorquin
Majorca Shepherd Dog	Brachycephalic	Medium	Perro de Pastor Mallorquin Ca de Bestiar, Shepherd Majorca Long Hair, Shepherd Majorca Short Hair
Maltese	Mesocephalic	Small	Maltese Micro, Maltese Miniature, Maltese Teacup
Manchester Terrier	Mesocephalic	Small	Manchester Old Format, Terrier Manchester Standard, Terrier Manchester Toy
Maremma Sheepdog	Dolichocephalic	Large	Abruzzenhund, Cane de Pastore, Maremma Pastore, Maremmo Abruzzese, Sheepdog Italian
Mastiff	Mesocephalic	Large	Mastiff English, Mastiff Old English
Medium Sized Anglo French Hound	Brachycephalic	Medium	Hound Anglo French Medium Sized
Miniature Pinscher	Dolichocephalic	Small	Pinscher Doberman Miniature, Pinscher Russian Miniature
Miniature Schnauzer	Dolichocephalic	Small	NA
Mix Breed	NA	NA	Hound, Terrier, African Sand, Africanis, Alopekis, American Indian, Balinese, Bear Tahltan, Bear Tiara Teddy, Bench Legged Feist, Biladi, Blue Lacy, Caldes, Canis Panther, Canoe, Cão de Castro Laboreiro, Carolina, Catahoula Leopard, Cattle Maasai, Cavapoochon, Chinese Chongqing, Chippiparai, Cobberdog Australian, Combai, Cormerlyn, Crossbreed, Cunucu Arubian, Cur Black Mouth, Cur Leopard, Cur Mountain, Cur Southern Blackmouth, Dingo, Doodle Comfort, Doodle Cross, Doodle Double, Feist Kemmer, Golden Labradoodle, Greek Kokoni, Hairless African, Hairless American, Heading New Zealand, Heinz, Hound American

			Leopard, Hound Caravan, Hound Cretan, Hound Estonian, Hound Generic, Hound Ground, Hound Latvian, Hound Lithuanian, Hound Plott, Hound Pot, Hound Scandinavian, Hound Trail, Huntaway, Huntaway New Zealand, Indian, Jackal, Jersey, Jocka, Kanni, Kirhiz, Kokon, Kokoni, Koolie German, Lacy, Lurcher, Meliteo Kinidio, Miki, Mongrel, Mouse Hunting Andalusiantive Australian, Non Pedigree, Pariah, Patterdale, Perro Ratonero Andaluz, Poi Hawaiian, Pointer Chester, Pointer Cyprus, Prague Krysarik, Prazsky Krysavik, Rastreador Brasileiro, Rat Hunter Prague, Ratonero Bodeguero Andaluz, Ratonero Mallorquin, Ratter Prague, Rescue Romanian, Sheepdog Greek, Sheepdog New Zealand, Shepherd American Tundra, Shepherd Dakotah, Shepherd Greek, Shepherd Italian, Sighthound Tahltan Kyrgyz, Simaku, Squirrel, Squirrel Cajun, Staghound, Street Balkan, Street Belgian, Street Romanian, Street Spanish, Taigan, Teddybear Tiara, Terrier American Crested Sand, Terrier American Hairless, Terrier Atlas, Terrier Austin, Terrier Australian Rough Coated, Terrier Beaver, Terrier Belgrade, Terrier Black Fell, Terrier Bull Guatemalan, Terrier Cyprus, Terrier Fell, Terrier Hairless, Terrier Italian, Terrier Lloyd, Terrier Lucas, Terrier Lucas Sporting, Terrier Patterdale, Terrier Plumber, Terrier Rat, Terrier Shropshire, Terrier Teddy Roosevelt, Terrier Tenterfield, Thai Bangkaew, Timber British, Trailhound, Treeing Tennessee Brindle, Wild Spanish
Mudi	Mesocephalic	Medium	Canis Ovilis Fenyesi, Mudi Hungarian
Neapolitan Mastiff	Mesocephalic	Large	Mastiff Napolitino, Mastino Napoletano
Newfoundland	Brachycephalic	Large	Moscow Vodolaz, Terranova
Norfolk Terrier	Mesocephalic	Small	NA
Norrbottenspitz	Mesocephalic	Medium	Norbottenspets, Spitz Nordic, Spitz Swedish
Norwegian Buhund	Mesocephalic	Medium	Buhund, Sheepdog Norwegian
Norwegian Elkhound	Mesocephalic	Medium	Elkhound
Norwegian Hound	Mesocephalic	Medium	Dunker
Norwegian Lundehund	Mesocephalic	Small	Lundehund, Lundhehund Norsk
Norwich Terrier	Mesocephalic	Small	NA
Old Danish Pointing Dog	Mesocephalic	Medium	Chicken Danish, Pointer Danish Old
Old English Sheepdog	Mesocephalic	Large	Sheepdog English, Sheepdog Working, Old English
Otterhound	Mesocephalic	Large	NA
Papillon	Mesocephalic	Small	Spaniel Continental Toy
Parson Russell Terrier	Mesocephalic	Small	NA
Pekingese	Mesocephalic	Small	NA
Peruvian Hairless Dog	Brachycephalic	NA	Alco Calato, Hairless Peruvian Large, Hairless Peruvian Medium, Hairless Peruvian Miniature, Inca Orchid Peruvian, Moonflower, Perro Flora, Perro sin Pelo del Peru, Peruvian Hairless Large, Peruvian Hairless Medium, Peruvian Hairless Miniature, Pila
Petit Bleu de Gascogne	Dolichocephalic	Medium	Bleu de Gascogne Petit, Gascon Petit, Gascony Small Blue, Hound French Hunting
Petit Brabancon	Dolichocephalic	Small	Griffon Brabancon Petite
Phalene	Brachycephalic	Small	NA
Pharaoh Hound	Mesocephalic	Medium	Hound Hunting Egyptian Pharoh, Kelb tal Fenek
Picardy Sheepdog	Dolichocephalic	Medium	Berger de Picard, Berger Picard
Picardy Spaniel	Mesocephalic	Medium	Spaniel Picardy
Pointer	Mesocephalic	Medium	Pointer Engilsh, Pointer English Short Hair, Pointer Short Hair
Poitevin	Mesocephalic	Medium	NA
Polish Greyhound	Dolichocephalic	Medium	Chart Polski
Polish Hound	Dolichocephalic	Medium	Hound Portuguese

Polish Lowland Sheepdog		Medium	Polski Owczarek Nizinny, Sheepdog Polish Lland, Sheepdog Portugese, Sheepdog Valee
Pomeranian	Mesocephalic	Small	Pomeranian Miniature, Pomeranian Teacup, Pomeranian Toy, Pom
Pont Audemer Spaniel	Mesocephalic	Medium	Epagneul Pont Audemer
Poodle	Mesocephalic	NA	Caniche, Poodle Australian, Poodle Cypress, Poodle Cyprus, Poodle Giant, Poodle Medium, Poodle Miniature, Poodle Moyen, Poodle Old Format Toy, Poodle Standard, Poodle Teacup, Poodle Thai, Poodle Toy
Porcelaine	Mesocephalic	Medium	NA
Portuguese Podengo	Dolichocephalic	Small	Hound Portuguese Warren Podengo, Hound Warren, Hound Warren Large, Hound Warren Long Hair, Hound Warren Medium, Hound Warren Short Hair, Hound Warren Small, Hound Warren Smooth Hair, Hound Warren Wire Hair, Hunting Portuguese, Podengo Canario, Podengo Pequeno, Podengo Portuguese Pequeno, Portuguese Podengo Large, Portuguese Podengo Long Hair, Portuguese Podengo Medium, Portuguese Podengo Short Hair, Portuguese Podengo Small, Portuguese Podengo Smooth Hair, Portuguese Podengo Wire Hair, Rabbit Portuguese, Podengo
Portuguese Pointer	Dolichocephalic	Medium	Perdigueiro Portuguese, Perdiguero Navarro, Pointer Old Spanish Navarro, Pointer Portuguese Perdigueiro, Pointing Portugese
Portuguese Sheepdog	Mesocephalic	Medium	Mountain Serra de Aires
Portuguese Water Dog	Dolichocephalic	Medium	Cão de Água Portie, Samador, Water Portuguese Long Hair, Water Portuguese Short Hair
Posavatz Hound	Mesocephalic	Medium	Hound Posavac, Hound Posavina
Presa Canario	Mesocephalic	Large	Brazilian Pressa, Canary, Dogo Canario, Perro de Presa Canario, Presa
Pudelpointer	Mesocephalic	Medium	NA
Pug	Mesocephalic	Small	Lo Sze Pugg
Pyrenean Mastiff	Mesocephalic	Large	NA
Pyrenean Mountain Dog	Brachycephalic	Large	Great Pyrenees
Pyrenean Sheepdog Long Haired	Brachycephalic	Medium	Sheepdog Pyrenean Long Hair
Pyrenean Sheepdog Smooth Faced	Mesocephalic	Medium	Berger des Pyrenees, Berger Petit, Sheepdog Pyrenean, Shepherd Pyrenean
Rafeiro do Alentejo	Mesocephalic	Large	Herder Alentejo
Retriever Chesapeake Bay	Mesocephalic	Large	NA
Retriever Curly Coated	Mesocephalic	Large	NA
Retriever Flat Coated	Mesocephalic	Large	NA
Retriever Golden	Mesocephalic	Large	NA
Retriever Labrador	Mesocephalic	Large	Labrador Black, Labrador Chocolate, Labrador Fox Red, Labrador Yellow, Labrodor, Retriever Fox Red, Labrador, Retriever
Retriever Nova Scotia Duck Tolling	Mesocephalic	Medium	Duck Toller, Retriever Tolling, Yarmouth Toller, Retriever Nova Scotia
Rhodesian Ridgeback	Mesocephalic	Large	Ridgeback Rhodesian Ridgeless, Ridgeback
Romanian Bucovina Shepherd	Mesocephalic	Large	Karakachan, Sheepdog Bukovina, Shepherd Bucovina, Shepherd Bulgarian, Shepherd Bulgarian Karakachan
Romanian Carpathian Shepherd Dog	Mesocephalic	Large	Sheepdog Carpathian, Sheepdog Romanian, Shepherd Romanian
Romanian Mioritic Shepherd Dog	Mesocephalic	Large	Sheepdog Mioritic, Sheepdog Romanian Mioritic
Rottweiler	Dolichocephalic	Large	Molosser Roman Utility, Rotti, Rottie, Rottweiler Roman
Russian Black Terrier	Mesocephalic	Large	Chornyi, Schnauzer Russian Bear, Terrier Tchiorny
Russian European Laika	Mesocephalic	Medium	Laika Russo, Laika Russo European
Russian Toy	Mesocephalic	Small	Russian Toy Long Hair, Russian Toy Smooth Hair, Terrier Moscow, Terrier Moscow Toy, Terrier Russian
Saarloos Wolfhound	Mesocephalic	Large	Wolf Saarloos

Saint Germain Pointer	Mesocephalic	Medium	Braque Saint Germain, Pointing St Germain
Saint Miguel Cattle Dog	Dolichocephalic	Large	NA
Saluki	Mesocephalic	Medium	Greyhound Persian, Hound Arabian, Hound Gazelle, Sighthound Persian
Samoyed	Mesocephalic	Medium	NA
Schillerstovare	Dolichocephalic	Medium	Hound Schiller
Schipperke	Mesocephalic	Small	NA
Schnauzer	Mesocephalic	Medium	Schnauser Standard
Scottish Terrier	Mesocephalic	Small	Scottie, Scotty, Terrier Aberdeen
Sealyham Terrier	Mesocephalic	Small	Terrier Sealydale
Serbian Hound	Dolichocephalic	Medium	Hound Balkan, Srpski Gonič
Serbian Tricolour Hound	Mesocephalic	Medium	Srpski Trobojni Gonič
Shar Pei	Mesocephalic	Medium	Mini Pei, Shar Pei American, Shar Pei Bear Coat, Shar Pei Chinese
Shetland Sheepdog	Mesocephalic	Small	Berger Shetland, Sheltie, Shetland Miniature
Shih Tzu	Mesocephalic	Small	Shih Tzu Imperial, Shih Tzu Micro, Shih Tzu Miniature, Shih Tzu Teacup, Tzu
Shikoku	Dolichocephalic	Medium	Kochi Ken, Mikawa Inu, Sanshu
Siberian Husky	Brachycephalic	Medium	Alaskan Husky, Alaskan Inuit, Huskie, Husky Alaskan, Husky Miniature, Inuit, Inuit Northern, Inuit Wolf, Husky
Skye Terrier	Mesocephalic	Small	NA
Sloughi	Mesocephalic	Medium	Greyhound Arabian
Slovakian Chuvach	Mesocephalic	Large	Slovensky Čuvač
Slovakian Hound	Dolichocephalic	Medium	Hound Black Forest, Hound Black Forrest, Slovensky Kopov
Slovakian Rough Haired Pointer	Mesocephalic	Large	NA
Smalandsstovare	Mesocephalic	Medium	NA
Small Münsterländer	Mesocephalic	Medium	Münsterländer
Soft Coated Wheaten Terrier	Mesocephalic	Medium	Terrier Irish Wheaten, Terrier Wheaten, Wheaten
South Russian Shepherd Dog	Mesocephalic	Large	Ovtcharka South Russian, Sheepdog South Russian
Spaniel American Cocker	Mesocephalic	Small	NA
Spaniel American Water	Mesocephalic	Medium	Spaniel Water
Spaniel Clumber	Mesocephalic	Large	NA
Spaniel Cocker	Mesocephalic	Small	Cocker American, Cocker English, Cocker Standard, Cocker Teacup, Cocker Toy, Cocker Working, Spaniel Miniature, Spaniel Short Legged, Spaniel Standard, Spaniel Teacup
Spaniel English Springer	Mesocephalic	Medium	Spaniel Springer, Springer
Spaniel Field	Mesocephalic	Medium	NA
Spaniel Irish Water	Mesocephalic	Medium	Spaniel Water Irish
Spaniel Sussex	Mesocephalic	Medium	NA
Spaniel Welsh Springer	Mesocephalic	Medium	Spaniel Welsh
Spanish Greyhound	Mesocephalic	Large	Galgo, Galgo Español, Hunting Spanish
Spanish Hound	Mesocephalic	Medium	Hound Spanish Large, Hound Spanish Small, Maneto, Sabuesos Español
Spanish Mastiff	Dolichocephalic	Large	Alano Spanish, Villano de Las Encartaciones, Mastin Spanish
Spanish Water Dog	Dolichocephalic	Medium	Spanish Water
St Bernard	Brachycephalic	Large	NA
Stabijhoun	Mesocephalic	Medium	Dutch Stabyhoun, Pointer Friesian
Staffordshire Bull Terrier	Mesocephalic	Small	Staff, Staffordshire, Staffie, Staffy
Swedish Lapphund	Mesocephalic	Medium	NA

Swedish Vallhund	Mesocephalic	Small	Cattle Swedish, Cattle Vallhund
Swiss Hound	Mesocephalic	Medium	Bruno de Jura, Hound Bernese, Hound Jura, Laufhund Swiss, Schweizer Laufhund, Schweizerischer Niederlaufhund
Taiwan Dog	Mesocephalic	Medium	Taiwan
Tatra Shepherd Dog	Dolichocephalic	Large	Mountain Polish, Mountain Tatra, Owczarek Podhalański, Sheepdog Polish, Sheepdog Tatra, Shepherd Polish, Shiper Tatra
Thai Ridgeback Dog	Mesocephalic	Medium	Mah Thai, Ridgeback Thai
Tibetan Mastiff	Mesocephalic	Large	Kyi Apso, Kyiapso Tibetan, Sheepdog Himalayan
Tibetan Spaniel	Mesocephalic	Small	NA
Tibetan Terrier	Brachycephalic	Small	NA
Tosa	Mesocephalic	Large	Japanese Tosa, Tosa Inu, Tosa Japanese, Tosa Ken
Turkish Kangal Dog	Mesocephalic	Large	Kangal Turkish, Kangal
Tyrolean Hound	Mesocephalic	Medium	Bracke Tyroler
Weimaraner	Dolichocephalic	Large	NA
Welsh Corgi Cardigan	Mesocephalic	Small	Corgi Cardi, Corgi Cardigan
Welsh Corgi Pembroke	Mesocephalic	Small	Corgi Pembrokeshire, Corgi Welsh, Corgi Welsh Old Format, Corgi
Welsh Terrier	Mesocephalic	Small	Terrier Welsh Fox
West Highland White Terrier	Mesocephalic	Small	Terrier Highland, Terrier West Highland, Westie, West Highland
West Siberian Laika	Dolichocephalic	Medium	NA
Westphalian Dachsbracke	Mesocephalic	Small	Hound German Deutsche Bracke, Swedish Dachsbracke
Whippet	Mesocephalic	Small	Whippet Standard, Whippet Wire Hair
White Swiss Shepherd Dog	Mesocephalic	Large	Berger Blanc Swiss, Shepherd Swiss
Xoloitzcuintle	Dolichocephalic	Small	Hairless Mexican, Hairless Mexican Miniature, Hairless Mexican Standard, Hairless Mexican Toy, Mexican Hairless, Mexican Hairless Intermediate, Mexican Hairless Miniature, Mexican Hairless Standard, Xoloitzcuintle Miniature, Xoloitzcuintle Standard, Xoloitzcuintle Toy, Xolo
Yorkshire Terrier	Dolichocephalic	Small	Terrier Yorkshie Standard, Terrier Yorkshire Miniature, Terrier Yorkshire Teacup, Yorki, Yorkie, Yorkshire
Yugoslavian Shepherd Dog	Dolichocephalic	Large	Charplaninatz, Mountain Illyrian, Mountain Kosovan, Mountain Macedonian, Mountain Yugoslav, Šarplaninac, Sharplanina, Šarplaninec Charplaninatz Illyrian, Sheepdog Illyrian, Sheepdog Serbian, Shepherd Yugoslavian

Supplementary Table 11. Classification of UK postcode area within region, determined by the UK National Statistics Postcode Directory[69].

Postcode Area	Region	Postcode Area	Region	Postcode Area	Region
AB	North East Scotland	GU	South East England	PH	Mid Scotland and Fife
AL	East England	GY	Channel Islands	PL	South West England
B	West Midlands	HA	London	PO	South East England
BA	South West England	HD	North West England	PR	North West England
BB	North West England	HG	Yorkshire and The Humber	RG	South East England
BD	Yorkshire and The Humber	HP	East England	RH	South East England
BH	South West England	HR	West Midlands	RM	London
BL	North West England	HS	Highlands and Islands	S	Yorkshire and The Humber
BN	South East England	HU	Yorkshire and The Humber	SA	West Wales
BR	London	HX	Yorkshire and The Humber	SE	London
BS	South West England	IG	London	SG	East England
BT	Northern Ireland	IM	Isle of Man	SK	North West England
CA	North West England	IP	East England	SL	South East England
CB	East England	IV	Highlands and Islands	SM	London
CF	South Wales	JE	Channel Islands	SN	South West England
CH	North West England	KA	South Scotland	SO	South East England
CM	East England	KT	London	SP	South West England
CO	East England	KW	Highlands and Islands	SR	North East England
CR	London	KY	Mid Scotland and Fife	SS	East England
CT	South East England	L	North West England	ST	West Midlands
CV	West Midlands	LL	North Wales	SW	London
CW	North West England	LN	Yorkshire and The Humber	SY	West Midlands
DA	South East England	LS	Yorkshire and The Humber	TA	South West England
DD	North East Scotland	LU	East England	TD	South Scotland
DE	East Midlands	M	North West England	TF	West Midlands
DG	South Scotland	ME	South East England	TN	South East England
DH	North East England	MK	South East England	TQ	South West England
DL	North East England	ML	Central Scotland	TR	South West England
DN	Yorkshire and The Humber	N	London	TS	North East England
DT	South West England	NA	Republic of Ireland	TW	London
DY	West Midlands	NE	North East England	UB	London
E	London	NG	East Midlands	W	London
EC	London	NN	East Midlands	WA	North West England
EH	Lothian	NP	South Wales	WC	London
EN	London	NR	East England	WD	London
EX	South West England	NW	London	WF	Yorkshire and The Humber
FK	Central Scotland	OL	North West England	WN	North West England
FY	North West England	OX	South East England	WR	West Midlands
G	Glasgow	PA	West Scotland	WS	West Midlands
GL	South West England	PE	East England	WV	West Midlands
				YO	Yorkshire and The Humber

Supplementary Note 1. Description of deduplication methodology. Full implementation of the workflow is available on Figshare: https://doi.org/10.6084/m9.figshare.24534151.v2, Source Code 1. As data were obtained from multiple sources, duplication of an individual across data sources was probable. Deduplication consisted of a four-phase process whereby duplicate individuals were limited to one entry. During Phase 1 of deduplication, 28.7% of the raw data were identified as exact duplicates (i.e., exact matches for any cases with more than 10/18 variables), with 6.2% of remaining cases unable to be identified as duplicates during Phases 2, 3 and 4, due to missing key variables (i.e., at least one of the following variables were absent: breed (free text), sex (M/F/unknown), date of birth (DOB; MM/YYYY), first three characters of dog name (common, not pedigree name).

Phase 1: ***Remove exact duplicates.***
- Calculate percentage data available for each variable.
- Calculate column completeness for each row (number of NAs per row).
- Set missing threshold (= 10/18 variables), and match duplicates if missingness is less than or equal to threshold. Otherwise, keep rows (with more missing data).

Phase 2:
- Match DOB (month/year), breed, name, sex, and microchip - status excluded.
- Then, match DOB (month/year), breed, name, sex, and microchip - status included i.e., should be left with one status now.
- Check status of matches and keep 'dead'.

Phase 3:
- Find rows which have multiple microchips (split string for those with multiple microchips).
- Find all candidate rows that correspond to the alternative microchips.
- Merge candidate rows if DOB (month/year), breed, name, sex, microchip, and status are all equivalent.
- If matching records are all ‘alive’ or ‘dead’, deduplicate down to a single record.
- If matching records has both ‘alive’ and ‘dead’ records, keep one ‘alive’ row and one ‘dead row.
- We then match again, merging candidate rows if DOB (month/year), breed, name, sex, and microchip are all equivalent (i.e., excludes status).
- This pairs the dead + alive row, and we set the ‘alive’ in the pair to ‘dead’. Thus, all alive + dead pairs are now dead + dead.
- Finally, merge candidate rows if DOB (month/year), breed, name, sex, microchip, and status are all equivalent, thus deduplicating dead + dead pairs.

Phase 4:
- Subset data to those with no microchip numbers.
- Merge candidate rows if DOB (month/year), breed, name, sex, postcode area, and status are all equivalent.
- If matching records are all ‘alive’ or ‘dead’, deduplicate down to a single record.
- If matching records has both ‘alive’ and ‘dead’ records, keep one ‘alive’ row and one ‘dead row.
- We then match again, merging candidate rows DOB (month/year), breed, name, sex, and postcode area are all equivalent (i.e., excludes status).
- This pairs the dead + alive row, and we set the ‘alive’ in the pair to ‘dead’. Thus, all alive + dead pairs are now dead + dead.
- Finally, merge candidate rows if DOB (month/year), breed, name, sex, postcode area, and status are all equivalent, thus deduplicating dead + dead pairs.
- Only duplicates left at this stage, are one copy with chip and one without chip (copies might have different STATUS).
- Deduplicate, based on DOB (month/year), breed, name, sex, postcode area, and status. This will merge rows with and without chip that have a matching STATUS.

Deduplicated data were then subset to rows where the following variables were complete: crossbred (Y/N); status (alive/dead) and sex (M/F). Due to the expansive nature of the dataset, and the absence of a mandate to report a dog's death, data were limited to dogs aged ≤ 18.3 years, i.e., the age at which 95% of the UK's pure and crossbred pet dog population were found to be deceased[24]. This measure was taken to ensure that the data only included dogs that were alive in 2019, thereby preventing artificial inflation of the resulting population estimate caused by the inadvertent inclusion of deceased dogs.
